# Small Disulfide Proteins with Antifungal Impact: NMR Experimental Structures as Compared to Models of Alphafold Versions

**DOI:** 10.3390/ijms26031247

**Published:** 2025-01-31

**Authors:** Jiawei Gai, Márk File, Réka Erdei, András Czajlik, Florentine Marx, László Galgóczy, Györgyi Váradi, Gyula Batta

**Affiliations:** 1 Department of Organic Chemistry, Faculty of Science and Technology, University of Debrecen, Egyetem tér 1, H-4032 Debrecen, Hungary; gai.jiawei@science.unideb.hu (J.G.); file.mark@science.unideb.hu (M.F.); erdei.reka@science.unideb.hu (R.E.); czajlik.andras@semmelweis.hu (A.C.); 2 Institute of Molecular Biology, Biocenter, Medical University of Innsbruck, Innrain 80-82, A-6020 Innsbruck, Austria; florentine.marx@i-med.ac.at; 3 Department of Biotechnology and Microbiology, Faculty of Science and Informatics, University of Szeged, Közép fasor 52, H-6726 Szeged, Hungary; galgoczi@bio.u-szeged.hu; 4 Department of Medical Chemistry, Albert Szent-Györgyi Medical School, University of Szeged, Dóm tér 8, H-6720 Szeged, Hungary; varadi.gyorgyi@med.u-szeged.hu

**Keywords:** antifungal proteins, AlphaFold, NMR, disulfide proteins, mini-protein

## Abstract

In response to the growth of emerging resistance to conventional antifungal drugs, antifungal proteins (AFPs) of filamentous Ascomycetes origin have been discovered in recent years. Understanding the structure of AFPs is crucial for elucidating their antifungal mechanisms and developing new therapeutic agents. While nuclear magnetic resonance (NMR) has proven effective in determining the structures of small proteins, some AFP structures remain unresolved, necessitating the use of alternative prediction methods. Through bioinformatics analysis and heatmaps of amino acid sequence identity and similarity matrix, we categorized AFPs into three major classes and six subcategories, revealing structural and bioactivity differences. We employed AlphaFold (AF) to predict the 3D structures of six different AFPs, with predictions compared to NMR-derived structures. The results demonstrated a high degree of consistency between AF and NMR structures, with AF excelling in structural quality assessment and accurately capturing complex disulfide bond patterns. Both AF2 and AF3 models outperform the NMR model in overall structural quality and coherence, with AF3 showing the best performance. However, the limitations of AF should be considered, including its reduced accuracy in predicting multi-metal ion complexes, suboptimal performance in highly flexible or disordered regions, and its inability to account for multiple conformers, as it generates only a single dominant structure. Moreover, while AF3 accurately predicts all disulfide bond patterns, AF2 falls short in this regard. This study verifies the reliability of AF in the structural prediction of cysteine-rich AFPs while highlighting these constraints, offering important support for the rational design of new protein-based antifungal drugs.

## 1. Introduction

Fungi, encompassing 2-11 million species [1], play multifaceted roles across various fields, including natural ecosystems, agriculture, industry, and medicine. Among them, certain species are pathogenic to plants, animals, and humans, posing significant threats to human health, food security, and agricultural productivity, with substantial safety risks and economic losses [2,3,4]. Currently, only three primary classes of antifungal agents are used to treat invasive fungal infections: polyenes (e.g., amphotericin B), azoles (e.g., fluconazole), and echinocandins (e.g., caspofungin) [5]. However, the rise in drug resistance has led to a growing number of fatal infections, resulting in approximately 1.5 million deaths annually [6,7]. Consequently, the urgent development of new antifungal agents is critical. AFPs secreted by Eurotiomycetes are among the most promising candidates [8]. Among them, PAF from *Penicillium chrysogenum* Q176 has been extensively studied, with its structure, properties, and biological functions well characterized [9,10,11]. PAF demonstrates excellent thermal stability, pH tolerance, and proteolytic resistance [12], indicative of robust structural and functional adaptability. Importantly, PAF has shown no cytotoxicity in both in vitro and in vivo studies [13,14,15], underscoring its safety profile as a potential therapeutic agent. Furthermore, as a biofungicide, PAF effectively inhibits the growth of plant pathogenic fungi, offering an eco-friendly solution for disease control, while also showing unique promise in antifungal therapeutics [16].

In addition to PAF, *P. chrysogenum* Q176 secretes PAFB and PAFC, while *Neosartorya* (*Aspergillus*) *fischeri* NRRL 181 produces NFAP and NFAP2, and *Aspergillus giganteus* secretes AFPg (to avoid confusion with the previously defined abbreviation “AFPs”, which stands for antifungal proteins, we refer to AFPg as the *A. giganteus* antifungal protein). These proteins are also promising antifungal drug candidates [17]. Despite sharing positively charged, cysteine-rich surfaces, these AFPs exhibit distinct structural, bioactive, and mechanistic properties. In terms of stability, PAFB [18], AFPg [19], and NFAP [20] demonstrate high stability under extreme pH and proteolytic conditions, similar to PAF. However, PAFC shows slightly lower tolerance to metal ions and reduced proteolytic stability [21]. Proteins like NFAP2 have been shown to exhibit good thermal stability due to their folded tertiary structure and disulfide bond stabilization [22]. Stability impacts their antifungal activity, with research suggesting that differences in the antifungal spectra and mechanisms of PAF, PAFB, NFAP, and AFPg are due to the presence and activity of host interaction molecules regulating uptake, signaling, and response to stress induced by the proteins [18], which ultimately determines the susceptibility of fungal species [23,24,25]. Furthermore, the surface charge and distribution of these proteins play a crucial role in PAF’s functionality, which further explains the differences in antifungal activity [11].

In terms of antifungal activity against filamentous fungi, PAF, PAFB [26], and PAFC [27] exhibit strong antifungal properties. These proteins enter fungal cells via endocytosis without disrupting the plasma membrane, accumulating in the cytoplasm and inducing the production of intracellular reactive oxygen species (ROS), ultimately leading to cell death [18,28]. In contrast, NFAP disrupts the cell wall organization of *Aspergillus nidulans* by breaking down chitin fibers [23], entering cells via passive transport rather than endocytosis [17]. AFPg, on the other hand, exerts its effects by disrupting the cell membrane, inhibiting chitin synthesis at the tips of *Magnaporthe grisea* hyphae, causing membrane permeabilization, and accumulating in the fungal nucleus to interact with nucleic acids, ultimately inducing cell death [22,29]. In terms of antifungal activity against yeast, NFAP2 stands out from other AFPs due to its remarkable antifungal effect on clinically relevant *Candida* species (including *Candida albicans* and other non-albicans strains) [30], rapidly disrupting plasma membrane structure and effectively killing yeast cells within minutes [22]. In contrast, AFPg and NFAP show no yeast-inhibitory activity [18]. PAFB demonstrates higher inhibitory potency against several non-albicans *Candida* species, particularly *Candida glabrata*, while PAF shows better efficacy against *Candida parapsilosis* [8,18]. Both proteins exhibit concentration- and time-dependent cell death, indicating a more complex mechanism than rapid killing (e.g., pore formation) [11,31]. PAFC not only inhibits plant pathogenic fungi (e.g., *Botrytis cinerea*) [32], but also exerts growth inhibition on certain *Candida* species, with similar inhibitory concentrations as PAF and PAFB [27]. It causes cell damage similar to NFAP2 [33] but requires internalization and cytoplasmic localization before plasma membrane permeabilization occurs, directing the action to intracellular targets. This mechanism is similar to those of PAF, PAFB [11,18], and NFAP [20] in their antifungal effects against filamentous fungi. The complex and distinct yet interconnected mechanisms of AFPs, such as binding, internalization, intracellular distribution, and the induction of apoptosis, are crucial for improving efficacy, specificity, and identifying potential fungal targets in drug design. Despite significant advancements in AFP research in recent years, numerous practical challenges remain. Achieving high yields of specific proteins from source organisms has long been difficult, which is why heterologous expression is widely employed. However, even with heterologous expression, obtaining large quantities of highly purified protein samples remains a considerable challenge. A precise understanding of protein structures is vital for elucidating these mechanisms. Although the tertiary structures of these AFPs have been thoroughly studied using NMR [20,32,34,35,36], the number of available AFP structures remains limited. (The structures of PAFg, PAFC, and NFAP2 each have only one available structure, PDB ID: 1AFP, 6TRM, 8RP9). 

In recent years, advances in attention-based machine learning models [37] and sequence covariance-based contact prediction methods [38,39], alongside the rapidly expanding genome sequence and experimental protein structure databases [40,41], have provided the foundation for deep learning applications in protein structure prediction [42]. AlphaFold2 (AF2), released by DeepMind in 2021, achieved remarkable success at Critical Assessment of Structure Prediction-14th edition (CASP14), significantly advancing protein structure prediction accuracy and attracting widespread scientific attention. On 9 October 2024, David Baker and his colleagues were awarded the Nobel Prize in Chemistry for their contributions to computational protein design and structure prediction. AF2′s backbone prediction accuracy (Cα root mean square deviation (RMSD) at r.m.s.d.95) reached a median of 0.96 Å, far outperforming the runner-up method, which scored 2.8 Å. For all-atom structure prediction, AF2 achieved 1.5 Å r.m.s.d.95, in contrast to the best competing method’s 3.5 Å [43]. AF2′s side-chain predictions are exceptionally precise, outperforming template-based methods even with strong templates [44]. AF2 has demonstrated experimental-level accuracy, with a database of 365,198 protein models now available to the scientific community [45]. Impressively, the vast majority of AF2 predictions achieve a global distance test total score (GDT_TS) above 80, with an average score of 92.4 (theoretical maximum of 100). Only five predictions scored below 70, mostly involving complex chains or NMR data. AF2′s performance for complexes is limited, as it was not designed for modeling conformational changes during complex formation. Its lower accuracy for NMR structures is more intriguing [46]. NMR structures are typically small, single-chain proteins and should be easier to predict. One explanation is that poor-quality NMR data might make AF2′s predictions more reliable. However, AF2 may struggle with NMR data because it was trained on crystallographic structures, differing from the solution conditions of NMR [47]. This observation is of particular interest to our research, which focuses on determining the NMR structures of AFPs from filamentous fungi belonging to the class of Eurotiomycetes. AFPs are small cysteine-rich mini-proteins with cationic properties that can inhibit the growth of fungi, bacteria, and even viruses. The correct disulfide bonding patterns in AFPs are critical for their stability and functional integrity [9,10,11,12,34]. This raises critical questions: Can AF2 predict AFP structures with NMR-level precision? Which approach provides better performance, particularly in capturing the disulfide bonding patterns? The answers to them remain unknown.

Building on the immense success of AF2 in protein structure prediction, researchers have begun to explore more complex biomolecular systems. While AF2 accurately predicts protein structures and their interactions, its application scope remains relatively limited, focusing primarily on individual proteins. To address this limitation, AlphaFold 3 (AF3) has been developed [48]. Building on AF2′s foundation, AF3 introduces new architectural modules and generative models to expand its predictive range to include intricate molecular systems, such as protein–ligand, protein–nucleic acid, and protein–protein complexes, with significant improvements in complex structure prediction [49,50]. AF3 also refines the process for multiple sequence alignment by reducing model complexity and replacing the “evoformer” module with a simplified “pairformer” module, which retains only pairwise representations as the basis for subsequent structure prediction. Additionally, AF3 integrates a diffusion module [51] that operates on atomic coordinates, improving local and global structural prediction accuracy through multiscale denoising. Its generative diffusion mechanism further ensures accurate geometric predictions even in regions with local uncertainty. However, a critical limitation of the current AF models remains: they typically predict static structures rather than capturing the dynamic behavior of biomolecular systems in solution. Even with multiple random seeds in the diffusion head or network, they cannot simulate the ensemble of structures in solution [46,48,52]. Given that the AFPs we studied with NMR exhibit intrinsic dynamics, a key question remains: can AF3, like AF2, accurately predict the 3D structures of AFPs? Finding the answer to this is central to our work. This paper aims to compare NMR and AF predictions, analyze the structural characteristics of AFPs, and improve structural information on AFPs.

## 2. Results

### 2.1. Bioinformatics Analysis of the Primary Structure of AFPs

**Figure 1:**
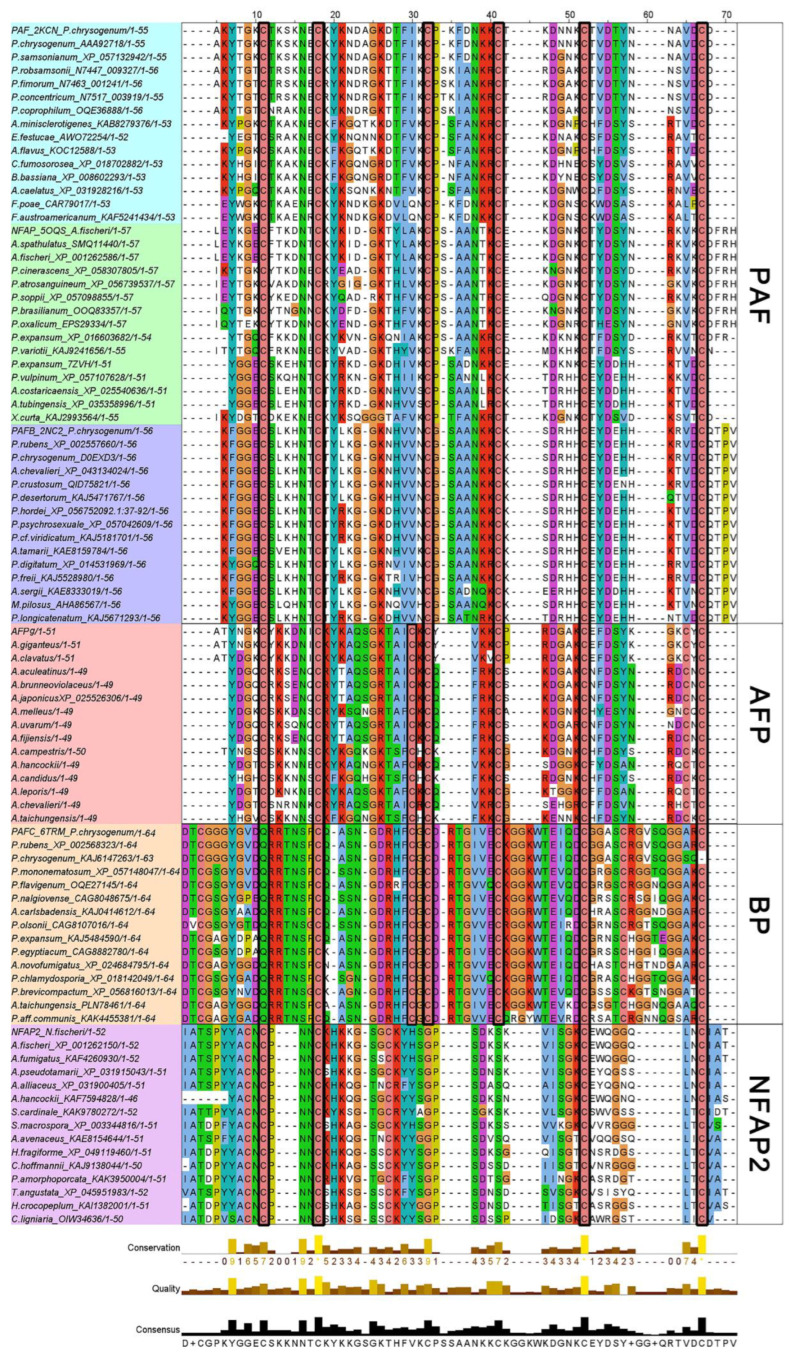
Multiple sequence alignment of AFPs’ amino acids. The alignment displays six AFPs (from top to bottom: PAF, NFAP, PAFB, AFPg, PAFC, and NFAP2) categorized into four groups based on prior phylogenetic clades: I-PAF (including PAF, PAFB, and NFAP), II-AFP (AFPg), III-BP (bubble protein, the class that includes PAFC due to their similarities), and IV-NFAP2 [55]. The conserved cysteines are highlighted using black rectangles. Below the alignment, metrics such as conservation, quality, and consensus are shown, representing the degree of amino acid conservation, sequence alignment quality, and consensus symbols, respectively. Amino acid sequences were downloaded from NCBI database after protein–protein BLAST searching, aligned using BioEdit, and analyzed and grouped with JalView. ClustalX scheme was used to indicate the conserved residues.

This study focuses on six AFPs: PAF, PAFB, and PAFC from *P. chrysogenum* Q176 (although referred to as ‘*P. chrysogenum*’ in this study to align with previous publications, it is now known that strains traditionally identified as *P. chrysogenum* are taxonomically classified as ‘*P. rubens*’ [53]); NFAP and NFAP2 from *N.* (*A.*) *fischeri* NRRL 181 (this study utilized the preliminary structure of NFAP2 [54]); and AFPg from A. giganteus. Phylogenetic analysis has classified AFPs into four major clades: Class I, which includes PAF (including PAF, PAFB, and NFAP); Class II, including AFPg; Class III, including BP (bubble protein, the class that includes PAFC due to their similarities); and Class IV, represented by NFAP2 [55]. In the NCBI’s protein–protein BLAST results, amino acid sequences from 15 different species representing six AFP types were selected for alignment based on prior phylogenetic clades (Figure 1). Proteins PAF, NFAP, and PAFB exhibited high homology within their respective subcategories. In Class I, six cysteine residues (marked by black rectangles) were highly conserved, which play a critical role in disulfide bond formation. These bonds are essential for maintaining protein structural stability and biological activity [9,10,11,12]. Class II AFPg shares a similarity with Class I PAF; among its eight cysteine residues, six align with highly conserved cysteines in Class I AFPs. Class I AFPs are characterized by a high content of basic, positively charged residues (K and R), which make up approximately 20% of the sequence; Class II AFPg, with shorter sequence lengths, exhibits an even higher proportion of basic amino acids (~25%), which may be linked to their antifungal activity. In contrast, Classes III and IV AFPs also exhibit high cysteine conservation but have a lower proportion of basic residues, around 12% and 7%, respectively. In six different AFPs, the N-terminus and C-terminus of the sequences contain one and two absolutely conserved cysteines. Across all 90 analyzed sequences, cysteine residues are highly conserved, underscoring their importance in AFPs’ primary structure. 

**Figure 2:**
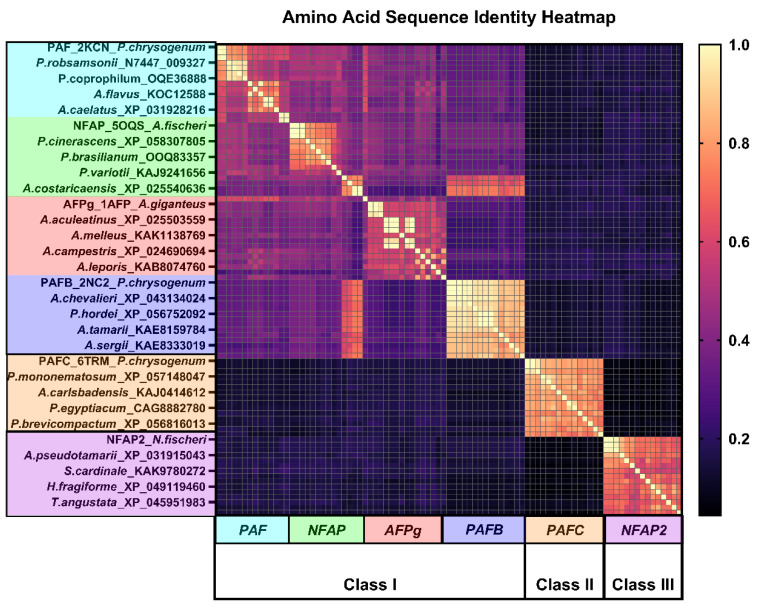
Heatmap of AFPs’ amino acid sequence identity. A light-colored large block along the diagonal represents Class I, while two smaller, brighter blocks in the bottom-right corner correspond to Classes II and III. The sequences are divided into six groups from top to bottom and left to right: PAF, NFAP, PAFB, AFPg, PAFC, and NFAP2, arranged in the same order as the sequence alignment. Each small square represents a numerical value, with the color gradient indicating the value intensity. (The sequence identity matrix was calculated using BioEdit, and the heatmap was generated with GraphPad.)

**Figure 3:**
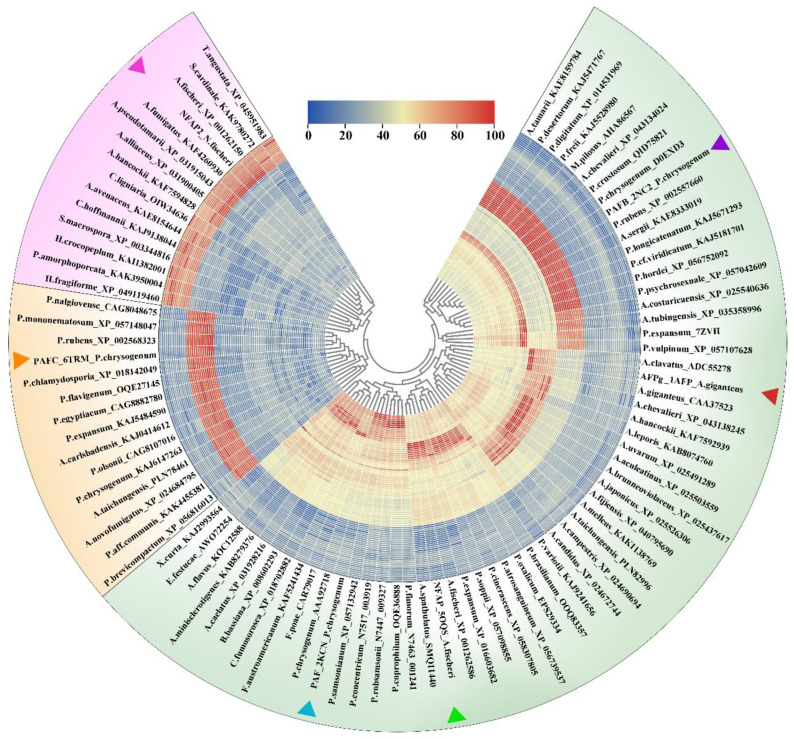
Amino acid sequence similarity cluster tree and heatmap of AFPs. The fan-shaped regions are arranged from inner to outer layers in the same order as the sequence alignment. Each small fan-shaped region represents a numerical value. The value is expressed as a percentage, ranging from 0 to 100. Classification is based on similarity: Class I (pink), Class II (orange-yellow), and Class III (green). The three sectors show low similarity to each other. The light yellow region represents Class I, where the similarity between subgroups causes some grouping shifts in the amino acid sequences, resulting in not every red region containing 15 sequences. (Generated using TBtools [56].)

The amino acid sequence identity heatmap derived from the 90 selected sequences (Figure 2) clearly delineates three major classes. A large, light-colored square is visible along the diagonal, corresponding to Class I, while two smaller, bright squares in the lower-right corner represent Classes II and III, respectively. The protein sequences of the PAFB, PAFC, and NFAP2 subcategories exhibit high consistency, showing three bright block regions, whereas sequence consistency in PAF, NFAP, and AFPg fluctuates, with a certain level of consistency observed among the four proteins subcategories in Class I. The inter-subcategory consistency values range from 0.3 to 0.5 (consensus matrix data available in Data S1). In the protein similarity dendrogram and heatmap (Figure 3), the 90 × 90 sequence similarity matrix reveals six red regions, representing the six subcategories. Each of the six categories of AFPs shows high conservation within their own group. The similarity heatmap further demonstrates that AFPs are divided into three major classes with substantial differences among them. Both Classes II and III consist of 15 original sequences, with no migration to other groups. In Class I, the AFPg subcategory sequences have not migrated to other subcategories and show relatively high conservation; this further highlights the unique position of AFPg within Class I. However, NFAP subcategory sequences have shifted toward the PAF and PAFB subcategories, changing their quantity, with some sequences in the NFAP group showing high similarity to PAFB, forming a small red sector. The overall sector for Class I appears yellow, reflecting similarity among the subcategories within Class I, with similarity values fluctuating around 50%; at the same time, it confirms the rationality of our classification (sequence similarity matrix data available in Data S2).

The classification presented through sequence conservation and similarity heatmaps, though differing from previous phylogenetic categorizations [55], is not without explanation. Phylogenetic trees, such as those based on maximum-likelihood (ML) methods, are constructed using amino acid sequence alignments and evolutionary models to infer relationships that emphasize shared ancestry and evolutionary history. The heatmap analysis reveals that PAF and AFPg share 30–50% sequence identity and exhibit approximately 50% similarity, classifying them within the same class. Previous sequence analyses confirmed their shared γ-core motif (the γ-motif, located in the N-terminus of PAF, is a GXCX_3-9_C structure associated with antifungal activity) [55], while phylogenetic studies placed them on distinct evolutionary branches. This suggests that these proteins likely share a common ancestral origin but diverged over evolutionary time to adapt to different ecological or functional demands. Such divergence, despite distinct origins, reflects convergent evolution, whereby functional similarities (e.g., antifungal activity) are maintained due to shared selective pressures. This suggests that despite originating from different lineages, they may have evolved similar structural and functional traits to adapt to analogous ecological or functional demands. This finding underscores the complementary nature of phylogenetic analysis and sequence similarity studies when investigating protein origins and functions. Conversely, PAFC and NFAP2 are clearly distinct from PAF and AFPg in both phylogenetic trees and heatmaps. Their unique features are underscored by their low sequence similarity, as visualized by predominantly blue regions on the similarity heatmap. These observations highlight the structural and functional divergence of PAFC and NFAP2 from the other AFPs. Although the AFPg family forms a distinct cluster in phylogenetic analysis, this study does not delve into the evolutionary history of its biological functions. Instead, it focuses on analyzing protein structures, including three structural levels. Considering the high sequence identity and similarity of AFPg to the PAF family in the primary structure, including subsequent analyses of secondary and tertiary structures (Table 1), and the unique primary structures of PAFC and NFAP2, AFPs are classified into three classes based on sequence similarity, core secondary structure, and overall folding patterns, i.e., based on structure characteristics rather than phylogenetic evolution: Class I: PAF (PAF, PAFB, NFAP, and AFPg, with AFPg considered a unique member within this class), Class II: BP (PAFC), and Class III: NFAP2. It is important to note that this study aims to perform a structural clustering analysis of AFPs rather than redefining their classification. This categorization not only enhances our understanding of the functional adaptations among family members but also provides new perspectives for further structural and functional studies.

### 2.2. AlphaFold2 Prediction of the PAF Structural Model

**Figure 4:**
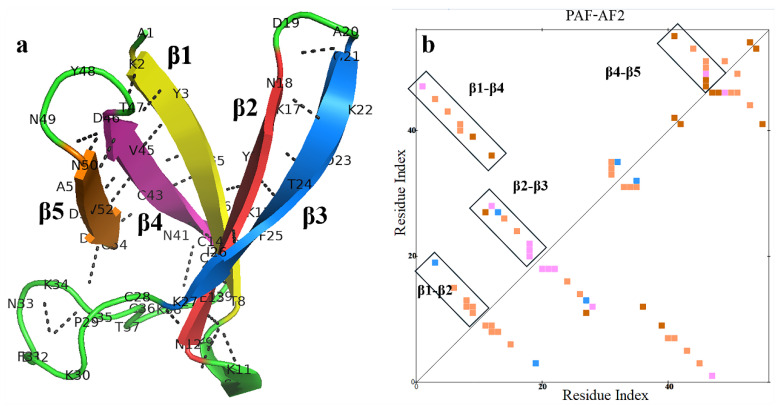
Hydrogen bond interaction distribution in the AF2 structure of PAF. (**a**) Distribution of backbone hydrogen bonds in PAF. (**b**) Internal hydrogen bond distribution within PAF. Hydrogen bonds are categorized based on their formation sites: main chain to main chain (orange), main chain to side chain (red), side chain to side chain (blue), multiple (pink). The colors are used to distinguish different categories of hydrogen bond interactions, and the calculations were performed using Discovery Studio (DS).

In November 2020, the results of the 14th Critical Assessment of Structure Prediction (CASP14) competition showed that the AI-based AF2 developed by DeepMind outperformed all other methods [43,57]. AF2 has achieved experimental-level accuracy in protein structure prediction, particularly for small molecules [43]. As small cationic mini-proteins (typically characterized by an amino acid length of 50–100 residues [58] and a molecular weight of <10 kDa [59]), AFPs were predicted in 3D using AF2 in the template-free mode. The AF2 prediction for PAF was compared with the NMR structure of PAF (optimized structure, Protein Data Bank [PDB] ID: 2MHV). The comparison revealed that the predicted structure exhibited very high confidence, with interface-placed distance-dependent test (IPDDT) deep blue, and was highly consistent with the NMR structure in terms of the five anti-parallel β-strands. However, there were some geometric and spatial orientation differences in the unstructured regions. The AF2 structure of PAF displayed a longer β-chain than the NMR structure (Table 1), with an α-helix formed between β1 and β2 by Lys9, Ser10, and Lys11. The hydrogen bonds formed by the main chain of the amino acids (Thr8-Lys11, Thr8-Asn12, Thr8-Glu13, Lys9-Asn12, and Lys6-Lys15) help to stabilize the α-helix and β-topology. The hydrogen bond distribution shows four linear regions along one side of the diagonal, stabilizing the β-topology between the β-strands (β1-β2, β2-β3, β1-β4, and β4-β5), thus maintaining the structural integrity of the protein’s β-fold (Figure 4a,b). 

**Figure 5:**
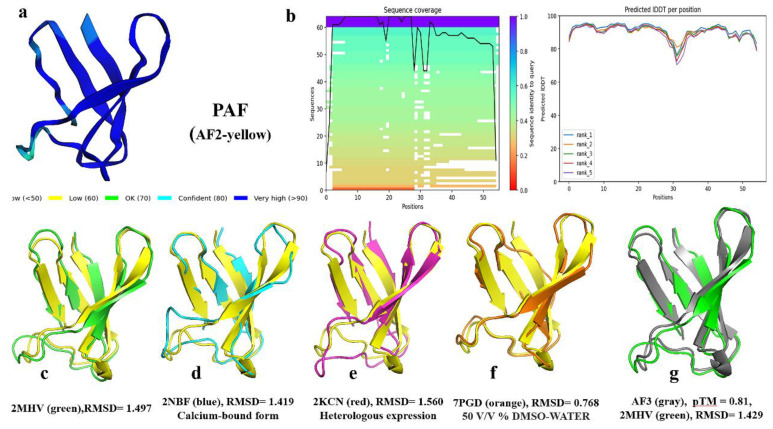
Comparison of the AF2-predicted structure with the NMR structures of PAF. (**a**) AF2-predicted confidence scores. (**b**) Predicted sequence coverage and residue scores. (**c**–**f**) Comparison of the AF2 model with the NMR structure in different solvents. (**g**) The AF3-predicted structure.

In the presence of different solvents, the β-topology of the NMR structure of PAF remains relatively unchanged, with notable differences concentrated in the coil region between residues K27 and N40 (Figure 4a and Figure 5c–f). When the solvent is DMSOd_6_-H_2_O (50 *v*/*v*%), the NMR structure exhibits the closest similarity to the AF2-predicted structure (Figure 5f), with an RMSD of 0.768. In contrast, the RMSD values for other solvents fluctuate around 1.5. Overall, the sequence alignment coverage is high, with most amino acid positions exhibiting predicted confidence scores (IPDDT) around 90. However, both AF2 and NMR structures show reduced confidence in predicting the coil regions (around 80), particularly in areas with lower sequence coverage. This observation highlights a correlation between lower prediction scores and reduced sequence coverage (Figure 5a,b). A surface-exposed loop is located at position D19 of PAF, and previous studies utilized site-directed mutagenesis to substitute Asp at position 19 with Ser (PAF^D19S^) to investigate its potential role in activity or binding. Structural analysis indicated that the overall 3D solution structure of PAF^D19S^ is nearly identical to that of PAF. However, PAF^D19S^ exhibits slightly increased dynamics and significant differences in surface charge distribution compared to PAF [11]. In AF2 predictions of PAF^D19S^, the substitution at position D19 leads to a reduction in amino acid coverage in this region, while the prediction confidence remains unchanged. The structure at D19 is represented by the same IPDTT blue coloring as in PAF, indicating similar prediction reliability. The AF2-predicted structure of PAF^D19S^ aligns more closely with the NMR structure obtained in a DMSO–water solvent, showing an RMSD of 1.093. This is slightly lower in similarity compared to the standard NMR structure, which has an RMSD of 2.888 (Figure S1). Additionally, the predicted surface electrostatic potential of the structure shows a shift from negative to neutral at position 19 (Figure S2), consistent with the results obtained through NMR.

**Figure 6:**
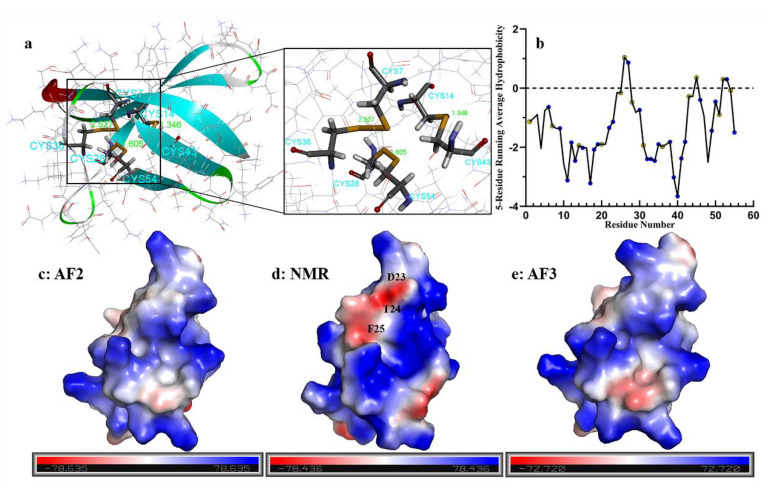
Disulfide bonds, hydrophobicity, and surface electrostatic potential of the PAF structure. (**a**) The AF2 model displays the correct disulfide bond pattern ‘*abcabc*; 7-36, 14-43, 28-54.’ (**b**) Hydrophobicity of amino acid residues in PAF. Blue represents hydrophilic residues; yellow represents hydrophobic residues. The running average is calculated using a sliding window of five residues (including the residue at position i and its two preceding and two succeeding residues): H_i_ = (H_i-2_ + H_i-1_ + H + H_i+1_ + H_i+2_)/5i. (**c**–**e**) Surface electrostatic potential of the AF2, NMR, and AF3 structures, respectively.

Disulfide bonds play a crucial role in stabilizing the PAF structure [9,10,11,12]. The AF2 results for PAF indicate a disulfide bond pattern of “*abcabc*; 7-36, 14-43, 28-54” (Figure 6a), which is fully consistent with previous studies on PAF [12]. Hydrophobicity analysis using a five-residue sliding average [60] (Figure 6b) reveals that PAF is mostly hydrophilic. The sequence contains thirteen positively charged hydrophilic lysine (K) residues (23.6%), seven negatively charged hydrophilic aspartic acid (D) residues (12.7%), and seven neutral hydrophilic asparagine (N) residues (12.7%). The average sliding values for hydrophilic residues are as follows: K11: -3.12, K17: -3.22, D39: -3.02, and N40: -3.66, while the highest sliding average for hydrophobic residues is observed at I26: 1.04. A comparison of the surface electrostatic potential energies among the three models, including AF3, reveals subtle conformational differences, resulting in variations in surface charge distribution. However, all three models exhibit a predominantly positively charged surface with basic residues. A notable difference is found in the third β-strand, where residues D23, T24, and F25 in the NMR structure (PDB ID: 2MHV) are exposed on the surface (Figure 6c–e), while in the AF2 and AF3 models, these residues are buried within the protein and do not present a negative charge, indicating deeper conformational differences between the NMR and AF structures.

### 2.3. AlphaFold3 Prediction of PAF–Metal Ion Complex Structure Models

Recently, Google DeepMind released AF3, which represents an improvement and optimization over AF2. AF3 not only enhances protein structure prediction but also enables the prediction of protein interactions with atoms, facilitating the study of complexes and their interactions [48]. Despite some initial criticism in the academic community due to AF3’s closed-source nature and usage limitations, it has demonstrated substantial advances. For PAF, the AF3-predicted structure has a pTM score of 0.81 (Figure 5g), with an RMSD of 0.316 when compared to AF2. The RMSDs between AF3 and various reference structures are as follows: 2MHV = 1.429, 2KCN = 1.329, 2NBF = 1.440, and 7PGD = 0.864. Overall, AF3 shows similar performance to AF2 in terms of prediction confidence, structural accuracy, and disulfide bonding patterns, further improving the similarity to the NMR structure.

**Figure 7:**
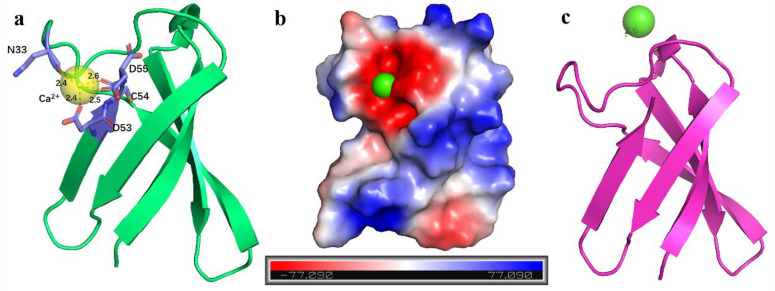
AF3 interaction predictions. (**a**) Ca^2+^-PAF complex, showing interactions between Ca^2+^ and the residues N33, D53, C54, and D55 of PAF. (**b**) Binding of Ca^2+^ to the anionic surface region of PAF. (**c**) Prediction of Ca^2+^ interactions with the PAF^D53S-D55S^ mutant, where Ca^2+^ interacts only with the solvent.

In addition to predicting the PAF structure, we also utilized AF3 to investigate interactions with metal ions. We simulated PAF complexes with varying numbers of Ca^2+^, Mg^2+^, and Na^+^ ions (one to four ions) to explore the structural interactions between PAF and these metal ions. The results (Table 2) revealed that while the structure prediction scores for all complexes remained high, the reliability and accuracy of docking decreased as the number of metal ions increased, showing a negative correlation. This suggests that PAF may bind to specific regions with a smaller number of metal ions, possibly due to spatial constraints. The Ca^2+^ ion was predicted to bind to a negatively charged surface region at the C-terminus of PAF, interacting with the oxygen atoms of residues N33, D53, C54, and D55 (Figure 7a,b). However, no distinct specificity was observed in the interactions, as all Ca^2+^, Mg^2+^, and Na^+^ complexes with PAF showed good binding scores. Additionally, we predicted complexes of PAF and its mutant variants with two metal ions. Although the binding scores for all experimental groups were high, the two metal ions in each group interacted with N33, D53, C54, and D55, showing no priority or competition in the interaction between these ions and PAF. In a previous study, we found that PAF and its mutant PAF^D19S^ are sensitive to Ca^2+^, affecting its antifungal activity in a concentration-dependent manner [11]. While the Ca^2+^ binding to PAF is relatively weak, it is specific. The primary Ca^2+^ binding site in PAF is located at the C-terminus, which was supported by molecular dynamics (MD) simulations. We confirmed that Asp53 and Asp55, along with the C-terminal carboxyl group, form the preferred Ca^2+^ binding site in PAF [61]. Furthermore, we predicted the complex of Ca^2+^ with the PAF^D53S-D55S^ mutant (Figure 7c). In this mutant, Ca^2+^ failed to interact with the protein and only interacted with the solvent (water), confirming the importance of the D residues at the C-terminus for Ca^2+^ binding. Previous studies have shown that mutating D53 and D55 to serine does not significantly affect the activity of PAF [61].

### 2.4. MolProbity Structural Evaluation Analysis

We compared the structural quality of the NMR structure, the AF2 model, and the AF3 model using MolProbity and recorded key metrics of PAF, including the MolProbity score, the atomic clash score, the side-chain rotamer outlier percentage, and the Ramachandran-plot analysis. The NMR structure was evaluated by averaging the results of 20 structures, while the AF models were assessed by averaging the top three predicted quality structures (Table 3). The MolProbity score of the NMR structure is 1.98, slightly higher than the AF2 model at 1.86 but significantly higher than that of the AF3 model at 0.673, indicating that the AF3 structure is more ideal compared to the other two. The Clashscore of the NMR structure is 1.29, much lower than the AF2 model’s 24.11, suggesting fewer atomic clashes in the NMR structure, whereas the AF2 model has more atomic clashes. The AF3 model’s Clashscore is 1.563, which is comparable to the NMR structure. In the NMR structure, 35.3% of the rotamers are poor rotamers, while both AF2 and AF3 models have 0%. The NMR structure has 43.2% favored rotamers, which is significantly lower than the 100% in both AF2 and AF3 models. The NMR structure contains 1.796% Ramachandran outliers, while the AF2 model has no outliers. In contrast, 97.92% of the NMR structure’s Ramachandran-favored residues are present, which is slightly lower than the 99.73% in the AF2 model and 99.3% in the AF3 model. The Ramachandran distribution Z-score for the NMR structure is -1.88 ± 1.04, while for AF2, it is 0.72 ± 0.95, and for AF3, it is 1.32 ± 1.03. This indicates that the NMR structure has a slightly abnormal distribution of torsion angles, while the AF2 model is closer to the ideal value. All models performed well in the Cβ deviation and cis-prolines metrics, with a score of zero. The NMR structure exhibited some minor anomalies with the Cα-based backbone local assessment method (CaBLAM) and in Cα geometry outliers, which were absent in the AF2 model (see Table S1 for criteria). In summary, the overall quality assessment shows that the AF3 model has the lowest MolProbity score, indicating the highest overall structural quality. The Clashscore of the NMR and AF3 models is lower, suggesting fewer atomic clashes compared to the AF2 model, which has more. The side-chain conformations in the AF2 and AF3 models are highly reasonable, whereas the NMR model shows poorer performance in this aspect. The Ramachandran-plot analysis of the AF2 and AF3 models is excellent (Figure S3a–c), while the NMR model has some outliers. In terms of structural validity, the AF3 model outperforms all others, followed by the AF2 model, while the NMR model has some structural issues. This indicates that both AF2 and AF3 models exhibit superior overall structural quality and reasonableness compared to the NMR model, with the AF3 model being the best.

### 2.5. Structural Analysis of Other AFPs

For other subcategories of Class I AFPs, AF structures exhibit longer β-strands and shorter β-turns compared to NMR structures. The β-topology formed by five β-strands is highly conserved, with two β-sheets following the β-strand patterns: β1-β2-β3 and β1-β4-β5, which are typical features of the Class I AFPs (Table 1, Figure S4). For example, the NMR structure of PAFB (Figures S4 and S5c) shows five short β-strands: Gly4-Cys6 (β1), Thr12-Leu16 (β2), Asn21-Asn25 (β3), His40-Asp44 (β4), and Arg49-Asp51 (β5), with a β-barrel topology that is relatively less pronounced, dominated by flexible loop structures. In contrast, the AF structure reveals much longer β-strands: Gly2-Cys7 (β1), Thr11-Leu17 (β2), Asn20-Asn25 (β3), His38-Asp44 (β4), and Arg48-Asp52 (β5), with a more conserved β-barrel topology. Notably, although the pafB gene is highly transcribed, the translated product could not be detected via fermentation in *P. chrysogenum* Q176 [18]. Structural and functional data for PAFB were obtained by studying a recombinant produced from the short-form version of PAFB, sfPAFB (2NC2), with a deletion of the N-terminal Lys1, and MD simulations confirmed favorable interactions between Lys1 and Asp42 [18]. These interactions were also observed in the AF3 prediction, where Lys1 and Asp42 form side-chain hydrogen bonds, stabilizing the β-sheet structure between β1 and β4 (Figure S6). Furthermore, the structural similarity between the NFAP models was as follows: RMSD-AF2: 1.429 and RMSD-AF3: 1.395. The NMR structure of NFAP features five antiparallel β-strands: Tyr3-Cys7 (β1), Cys14-Lys17 (β2), Thr22-Ala25 (β3), Lys41-Asp45 (β4), and Lys50-Asp54 (β5). These β-strands are connected by three small loops (Phe8-Thr13, Ile18-Lys21, and Ser46-Arg49) and one large loop (Lys26-Asn40) to form β-turns [20]. In the AF2 and AF3 models, longer β-strands are observed: from Tyr2 to Cys8 (β1), from Cys12 to Lys18 (β2), from Thr21 to Ala26 (β3), from Lys39 to Asp45 (β4), and from Lys49 to Asp53 (β5). The β-turns in AF2 are shorter, and the directionality of the first small loop’s β-turns is opposite to that seen in the NMR structure. In terms of electrostatic surface potential, AF structures align with the NMR data, showing no large positively charged regions [20]. For the AFPg structure, the NMR structure exhibited more violations during the overall evaluation, while AF3 performed significantly better (Table S2a,b). Regarding MolProbity evaluation, the structural quality of the NMR and AF models follows similar trends as observed with PAF. Both NMR and AF3 models exhibit fewer atomic clashes, while AF2 shows more clashes. The side-chain conformations in both AF2 and AF3 models are highly reasonable, whereas NMR exhibits deficiencies in this area. Ramachandran-plot analysis for both AF2 and AF3 models shows excellent results, while NMR has some deviations in backbone dihedral angles (Figure S7). Overall, AF3 demonstrates the best performance across all metrics, followed by AF2, while the NMR model displays certain structural issues. This indicates that the AF2 and AF3 models can outperform the NMR model in overall structure quality and rationality, with AF3 being the superior model.

**Figure 8:**
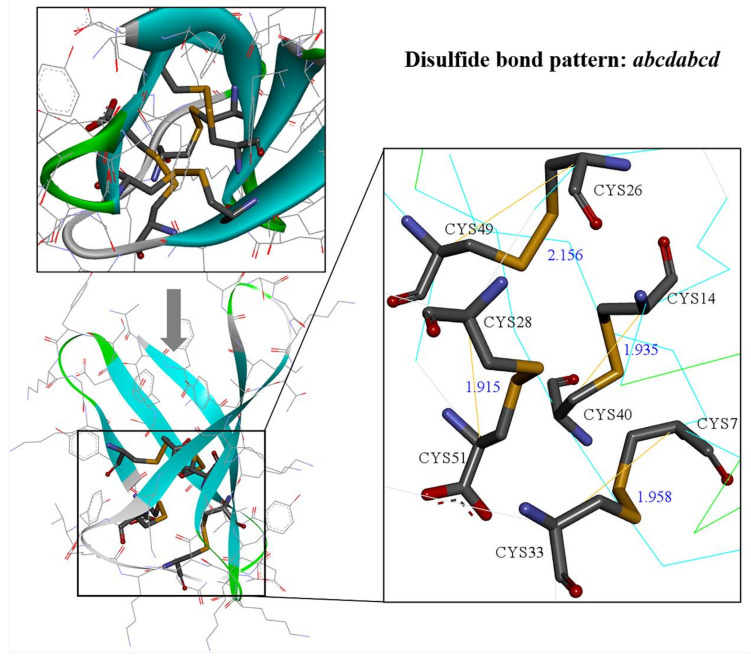
AF3-predicted structure of AFPg. The AF3-predicted structure of AFPg is shown, where four disulfide bonds stabilize the β-barrel topology. The disulfide bond pairing follows the *abcdabcd* pattern with bond pairs at positions 7-33, 14-40, 26-49, and 28-51. This result was not observed in the NMR structure.

Although AF2 has shown remarkable performance in recent years, it is not perfect for AFPs. AF3, released this year (2024), has proven to be even more reliable. The disulfide bonds between cysteine residues play a crucial role in stabilizing the protein structure. These disulfide bonds are highly stable across a wide pH range and at high temperatures, preventing proteolytic degradation [44]. It has been demonstrated that the presence and correct formation of disulfide bonds are vital for both structural integrity and antifungal activity [29,34]. In PAF, PAFB, and NFAP, six cysteine residues form three disulfide bonds in an *abcabc* pattern [9,11,12,62], whereas in AFP and PAFC, four disulfide bonds connect eight cysteines, allowing for multiple possible pairing combinations, resulting in a relatively complex pattern [10,19]. Notably, the C-terminal of AFPg features two cysteine residues separated by an amino acid, complicating the disulfide bond formation. Indeed, AF2 models show suboptimal performance in predicting correct disulfide bond pairings in AFPs, while AF3 accurately predicts all disulfide bond pairings (Table 1). Despite the structural similarities between AF2 and AF3 predictions, AF2 did not correctly predict the disulfide bond pairings for NFAP [20], AFPg [34], or PAFB [18], whereas AF3′s predictions correctly identified the disulfide bonding patterns (Table 1). Interestingly, the NMR results for AFPs show that due to the spatial proximity of C14, C27, C42, and C53 in NFAP, the disulfide bonds formed are twisted, leading to potential geometric distortions. Such irregularities may interfere with the protein’s overall conformation and stability. Previous NMR studies on the solution structure of AFPg also suggested the presence of non-native disulfide bond conformations, yielding conflicting results [19]. AFPg contains eight cysteine residues, complicating the correct pairing of cysteine thiols and resulting in 105 possible pairing combinations. The interlocking *abcdabcd* disulfide bond pattern is considered the most likely for AFPg and has been confirmed by recent chemical synthesis strategies using semi-orthogonal thiol protection groups. These strategies validated the disulfide bonds between cysteines 26 and 49 and 28 and 51 (Table 1), supporting the *abcdabcd* pattern. However, the precise pairing remains unclear [34,62]. In the AF3 predictions, we observe eight cysteines forming four disulfide bonds in an *abcdabcd* pattern: 7-33, 14-40, 26-49, and 28-51, stabilizing the β-barrel topology formed by five antiparallel β-strands (Figure 8), which aligns with experimental hypotheses and provides substantial support for this disulfide bond arrangement. 

**Figure 9:**
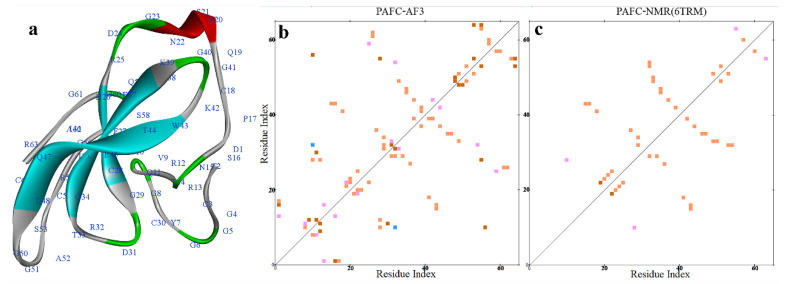
AF3 structure and hydrogen bond interaction distribution of PAFC. (**a**) AF3 structure of PAFC, with secondary structures indicated: sheet (blue), helix (red), and turn (green). (**b**) Distribution of hydrogen bond interactions in the AF3 structure of PAFC. (**c**) Distribution of hydrogen bond interactions in the NMR structure of PAFC (PDB ID: 6TRM). In the AF3 structure of PAFC, the number of hydrogen bond interactions between amino acids is significantly higher than in the NMR structure. Hydrogen bonds are categorized based on their formation sites: main chain to main chain (orange), main chain to side chain (red), side chain to side chain (blue), and multiple (pink).

In addition to producing Class I-category PAF and PAFB, *P. chrysogenum* also co-expresses the Class II-category PAFC (PDB ID: 6TRM) during fermentation [27]. The mature PAFC (64 aa; 6.63 kDa) is a unique example among AFPs, being slightly longer than the mature PAF (55 aa; 6.25 kDa) and PAFB (58 aa; 6.55 kDa) [10,18]. PAFC contains two putative levomeric γ-core motifs (CX_3-9_CXG). One is located at the center of PAFC (CDRTGIVECKG) and is highly conserved among the BP cluster of ascomycetous antimicrobial peptides (AMPs), while the second, shorter motif with lower homology resides near the C-terminus (CGGASCRG) [27,63]. This common γ-core structural motif, characterized by the consensus sequences GXCX_3-9_C (right-handed conformation) or CX_3-9_CXG/CX_3-9_GXC (left-handed conformation), is widely found in cysteine-stabilized small peptides across various biological kingdoms and is potentially associated with antimicrobial activity. [63,64]. Previous NMR results indicated that PAFC has a well-folded structure, with the C-terminal β-chain forming two anti-parallel β-sheets: the first sheet consists of three β-strands (β1, His26–Cys28; β2, Gly34–Lys39; β3, Lys42–Asp48), and the second sheet consists of two β-strands (β4, Arg55–Val57; β5, Gly60–Arg63). An α-helix is located between Ala20–Asp24. Although the second β-sheet is more irregular, experimental data suggest possible hydrogen bonds in this region that help stabilize the structure [32]. In the AF predictions, AF2 performed poorly with an RMSD of 3.129, failing to predict the correct disulfide bond pattern (Table 1, Figure S5d). This may be attributed to the dynamic nature of PAFC, as previous studies have shown that proteins with dynamic or conformational equilibrium can yield less accurate AF2 predictions compared to NMR [65,66]. The AF3-predicted structure, however, shows a high similarity to the NMR structure (RMSD = 0.868). The AF3 model only includes the first sheet with three β-strands (β1, 26–28; β2, 34–38; β3, 43–48) (Figure 9), while the two short β-strands at the C-terminal are predicted to be irregularly coiled and connected by a turn formed by Ser58 and Gln59. The α-helix is slightly shorter, spanning Ala20–Asn22, and the Gly23–Asp24 region is predicted as a turn. However, the AF2 model includes the C-terminal β-strands β4 (Arg55–Val57) and β5 (Gly61–Arg63) (Figure S4). Both NMR and AF3 structures of PAFC show significant unordered coil content at the N-terminal, with AF3 utilizing more turns to alter the coil direction and maintain structural integrity. The structure also includes numerous possible hydrogen bonds, with only a few stabilizing interactions between β-strands, while most are between coils and β-strands (Figure 9). The AF3 model predicts four correctly paired disulfide bonds following the *abcabdcd* pattern: 3–30, 18–38, 28–54, 49–64. The eight cysteines are surrounded by hydrophilic amino acids (Asp1, 31, 48; Gln19, 47; Glu37) and hydrophilic, positively charged amino acids (His26; Arg25, 32, 55, 63; Lys39). Interestingly, the average hydrophobicity of the five surrounding cysteine residues in AF3 is maintained in a relatively neutral range (−1 to 1), unlike in Class I-category AFPs such as PAF, NFAP, and PAFB, where the cysteines are located in more hydrophilic regions. Eight cysteines of AFPg exhibit a broader range of hydrophobicity. This may be closely related to the unique structural characteristics and catalytic functions of AFPs (Figure S9, marked dots). While AF models excel in predicting disulfide bond connectivity and structural accuracy, they exhibit notable limitations. According to our previous NMR studies, PAFC is a dynamic protein, particularly displaying significant high-dynamic behavior in two γ-core regions between residues 30–40 and 49–56 [55,67]. The average NMR S^2^ order parameter was measured as 0.76 ± 0.11, which is slightly lower compared to PAF (S^2^ = 0.81 ± 0.05) and NFAP (S^2^ = 0.82 ± 0.09), indicating that PAFC has higher flexibility on the ps–ns timescale [12,20,32]. However, neither the AF2 nor AF3 models can capture this dynamic nature, highlighting their limitations in addressing the dynamics of proteins, despite their outstanding performance in static structure prediction. For Class III NFAP2, although the NMR structure is preliminary and was not structurally evaluated, its disulfide pattern has been confirmed. Due to the absence of trypsin and chymotrypsin cleavage sites in the NFAP2 sequence, traditional mass spectrometry-based disulfide mapping methods are not applicable [54]. Previous computational predictions based on the initial NMR solution structure suggested two potential disulfide bond isomers. These were chemically synthesized and analyzed using reverse-phase high-performance liquid chromatography, circular dichroism, and NMR spectroscopy. Combined with antifungal assays, the natural disulfide bonding pattern (*abbcac*: 9-40, 11-15, 23-49) was confirmed [54]. Both AF2 and AF3 predicted the correct disulfide pattern, with a structural similarity of approximately 1.2 to the preliminary NMR data.

## 3. Discussion

In recent years, we have focused our research on antifungal proteins, which are cysteine-rich, cationic mini-proteins. Despite sharing these core characteristics, these AFPs differ significantly in origin, structural features, biological activity ranges, mechanisms of action, and efficiency. While NMR spectroscopy has been highly effective in determining the structures of small proteins, some AFP structures may be difficult to solve. High-quality protein samples are critical for NMR structural determination and bioactivity assays. For instance, during the overexpression of PAFB [18] and AFPg [34], variations in the N-terminus may occur, influencing protein stability and folding, which can complicate NMR analysis that requires stable, uniform structures [18]. Additionally, protein heterogeneity, uneven distribution in solution, and low concentrations can significantly affect NMR data quality, making structural analysis more challenging. Disulfide bond isomerism (e.g., for AFPg [19,34]) leads to multiple bond connectivity patterns within protein samples, making it difficult to resolve specific disulfide linkages. To determine these linkages, chemical synthesis is often employed (e.g., for NFAP2 [54]) to determine these linkages, though subtle differences, such as post-translational modifications or distinct folding environments, may introduce additional uncertainties. Disulfide bond isomerism can also disrupt local protein folding, causing signal instability or reduced resolution in NMR measurements [34]. Furthermore, protein dynamics can interfere with the detection of the nuclear Overhauser effect (NOE), a process critical for resolving disulfide bonds using NMR [32,34]. Therefore, relying solely on NMR is often insufficient to fully elucidate the disulfide connectivity of AFPs. Complementary techniques, such as MS, are essential to validate disulfide bond patterns. By integrating data from multiple approaches, more reliable and comprehensive structural insights can be achieved.

To compare the NMR-resolved structures with those predicted using AF, we conducted bioinformatics analyses, 3D structural comparisons, and complex structure predictions, integrating these results into a comprehensive evaluation. We used heatmap visualizations to analyze sequence consistency and similarity matrices, classifying the AFPs into three major classes and six subcategories (Figure 2 and Figure 3; Table 1). Unlike traditional phylogenetic tree classifications based on evolutionary history and tracing ancestral relationships, our approach emphasizes clustering by sequence and structural identity, providing a fresh perspective. The sequence consistency matrix (Data S1 and S2) quantifies conservation by scoring residue alignment across multiple sequences, illustrating the degree of conservation at specific positions. Meanwhile, the similarity matrix accounts for amino acid properties and calculates pairwise similarity scores, reflecting sequence alignment similarities. The similarity matrix corroborated the classification into three classes and six subcategories, showing intra-subclass similarities within Class I, while Classes II and III demonstrated absolute conservation and distinctiveness. Disulfide bonds emerged as a critical factor for AFPs stability [9,10,11,12,34]. Our analyses revealed that cysteine residues are highly conserved across 90 sequences grouped into six clusters with high intra-cluster similarity. Conversely, although cationic properties are essential for AFPs’ biological activity, no specific basic amino acids were universally conserved. However, a very high overall abundance of basic residues was consistently observed across all AFP sequences, underscoring their importance in antifungal function (Figure 1).

Our analysis showed that the predictive accuracy of AlphaFold methods for AFP structures is comparable with NMR, with AF3 often surpassing NMR in certain metrics (Table 3). The AF3 model achieved the highest overall structural quality, with fewer atomic clashes than the NMR and AF2 models (Table 3 and Table S2). For side-chain conformations, AF models consistently outperformed NMR, with AF3 achieving nearly ideal side-chain positioning, where over 99% were in favored conformations. The evaluation of backbone dihedral angles, Ramachandran-plot outliers, and Cβ deviations showed that AF3′s conformations were most reasonable, with no residues in outlier regions. In contrast, the AF2 and NMR structures exhibited a few outliers (Figures S3, S7 and S8). Overall, the AF2 and AF3 structures exceeded NMR in structural quality, with AF3 achieving the best results.

For the secondary structure of AFPs, AF2 generally provides accurate β-topology predictions. However, it yields relatively lower pLDDT scores for certain coil or multi-coil regions. For example, the coil between β3 and β4 in PAFB and disordered coils in PAFC exhibit lower confidence in predictions. The pTM scores of AF3 ranged from 0.74 to 0.87, with values above 0.8 indicating high-quality predictions (Figure 5 and Figures S1 and S5). Compared to NMR structures, AF-predicted structures typically feature longer β-strands, with more hydrogen bonds designed between them to maintain the stability of β-sheet structures, turn regions, and certain disordered coils (Figure 9 and Figure S6). However, such designs may be less suitable for dynamic proteins, as the enforced stabilization could limit their inherent flexibility and dynamic conformational changes. Notably, AF3 correctly predicted disulfide bond pairings (Table 3), unlike AF2, which could accelerate experimental validation of pairing patterns and reduce time, cost, and effort in the lab. Remarkably, AF3 even resolved the disulfide bond pairing patterns of AFPg (Figure 8), which have remained unresolved to date. A detailed analysis of PAF structures confirmed high pLDDT and pTM scores for both AF2 and AF3 models, with the predicted structure closely matching PAF in DMSO–water solvent (Figure 5). Using AF3, we simulated protein–metal ion complexes (Table 2, Figure 7), observing high pTM scores (>0.8) and reasonable structures for complexes with Ca^2+^, Mg^2+^, and Na^+^ ions. Although NMR experimental data indicated PAF sensitivity to Ca^2+^, AF3 did not reveal marked binding preferences, possibly due to the spatial arrangements of metal ions and differences in electrostatic interactions, leading to varying binding preferences and interactions. Overall, we still consider AF an outstanding tool for protein structure prediction. While AF mostly outperforms NMR in predicting overall structural quality, dihedral angle accuracy, and disulfide bond pairing, it has limitations in capturing protein states across various solvents, dynamic structural flexibility, and conformational changes—areas where NMR retains a relative advantage. Additionally, although AF3 excels in predicting hydrogen bond stability, β-sheet construction, and certain disordered regions, there remains room for improvement in predicting more complex ligand-binding structures and multi-metal ion complexes. In conclusion, AF3 has demonstrated remarkable efficacy in accurately predicting all disulfide bond linkages in AFPs with ease. However, significant limitations persist. AF models cannot observe diverse protein conformations under different solvent conditions or monitor AFPs’ folding states under varying physicochemical conditions (e.g., temperature, pressure, pH, and denaturants). Additionally, certain aspects of protein dynamics, such as local flexibility in PAFC [32], NFAP2 [54], and other AFPs, remain inaccessible to a single AF model. Thus, while AF shows exceptional promise, we maintain that it cannot entirely replace NMR.

In future studies, we could further explore the complementarity between AF, especially the AF3 model, and NMR structural analysis. Tejero R et al. utilized AF-predicted structural models to accelerate the determination of the overall protein backbone, providing a valuable reference for NMR experiments [46]. AF predictions not only optimized NMR constraints, such as the selection of NOE and dihedral angles, thereby reducing ambiguities in structural analysis, but also significantly enhanced structural accuracy. Moreover, for dynamic or disordered regions, AF offered plausible conformational hypotheses, effectively complementing experimental data. Notably, in the verification of complex structures like disulfide bond pairings, AF predictions provided critical support, further improving the reliability and precision of NMR-based structural elucidation. Looking ahead, combining AF with other experimental methods could further enhance predictive accuracy. For instance, using initial structures of AF as a reference for NMR analysis may accelerate the preliminary model-building and validation process; conversely, NMR data could guide AF in optimizing prediction algorithms or refining model training to simulate protein structures more accurately across different solvent environments. Integrating wet-lab experiments with AF promises deeper insights into the structure, function, and interactions of small proteins like AFPs at reduced experimental cost and improved predictive accuracy, facilitating more targeted structure–function research.

## 4. Materials and Methods

The Analysis of Protein Primary Structure: Sequence information was obtained from NCBI-Blast [https://www.ncbi.nlm.nih.gov/] (accessed on 1 August 2024) and downloaded using pro-pro. Sequence alignment was performed using BioEdit 5.0.9, followed by grouping analysis with JalView 10.0.7 and sequence coloring based on ClustalX. The sequence identity heatmap was generated through the sequence identity matrix function in BioEdit 5.0.9 and visualized using GraphPad Prism 10.1.2. The similarity heatmap was created using TBtools-II v2.142.

Structure acquisition: AFP structures were predicted through AI tools using AlphaFold models: AlphaFold2 [https://alphafold.ebi.ac.uk/] (accessed on 18 July 2024) and AlphaFold3 https://blog.Google/technology/ai/google-deepmind-isomorphic-alphafold-3-ai-model/] (accessed on 25 September 2024). NMR structure data for AFPs were obtained from the RCSB Protein Data Bank [https://www.rcsb.org] (accessed on 16 May 2024).

Structure Evaluation: The NMR and AF structure models were assessed using the MolProbity tool [http://molprobity.biochem.duke.edu] (accessed on 30 September 2024).

Structure Analysis: Structural analysis of the proteins was performed using PyMOL 3.0.0 and Discovery Studio 4.5.0.

## 5. Conclusions

In this study, we conducted a comprehensive analysis of six AFPs derived from different sources, focusing on their structural characteristics and prediction models. Our results highlight that while NMR has been effective in determining the structures of small proteins, certain AFP structures remain unresolved due to sample heterogeneity, the complexity of disulfide bond formation, and dynamic structural features. In contrast, AF, particularly the AF3 model, demonstrates significant advantages in predicting AFP structures with high accuracy, outperforming NMR in aspects such as disulfide bond pairing, backbone conformation, and side-chain orientation. Our comparison between NMR and AF structures reveals that AF3 not only provides high-quality structural predictions but also excels in predicting key structural elements, such as hydrogen bond stability and β-sheet formation. The disulfide bond pairing predicted using AF3 closely matches experimental results, showcasing its potential to aid in structural elucidation.

However, despite AF’s impressive accuracy, NMR still maintains its irreplaceable value, particularly for studying protein dynamics, complex ligand-binding interactions, and in the presence of multiple conformations. Future research should focus on integrating AF predictions with NMR data to enhance structural accuracy, particularly in dynamic and ligand-binding contexts. This complementary approach will provide a more complete and reliable understanding of protein structures.

In conclusion, AF, especially the AF3 model, proves to be a powerful tool for protein structure prediction, though there is still room for improvement in its prediction of dynamic behavior and complex ligand binding. By combining AF with experimental methods, we can achieve a more accurate and cost-effective approach to studying the structure, function, and interactions of AFPs and other small proteins, advancing our knowledge in these areas.

## Figures and Tables

**Table 1:** Structural information of AFPs. The table summarizes the structural details of AFPs, including their source, differences between the AF2-predicted structure and the NMR structure, disulfide bond formation patterns, and whether the disulfide bonds are correctly paired. The PDB IDs for the structures are as follows: 2MHV, 5OQS, 1AFP, 2NC2, 6TRM, and 8RP9.

	PAF	NFAP	AFPg	PAFB	PAFC	NFAP2
**Organism**	*Penicillium* *chrysogenum* Q176	*Neosartorya (Aspergillus) fischeri* NRRL 181	*Aspergillus giganteus*	*Penicillium* *chrysogenum* Q176	*Penicillium* *chrysogenum* Q176	*Neosartorya (Aspergillus) fischeri* NRRL 181
**Aa len/Mw**	55Aa/6.26kDa	57Aa/6.64kDa	51 Aa/5.82kDa	56Aa/6.32kDa	64Aa/6.64kDa	52Aa/5.57kDa
**NMR**	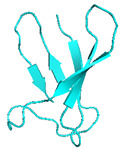	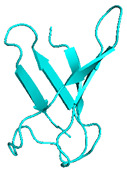	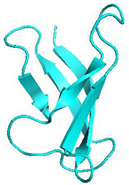	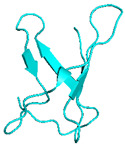	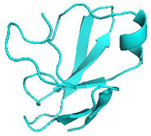	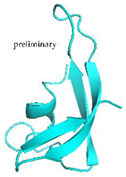
**AF2**	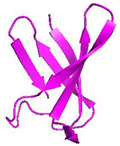	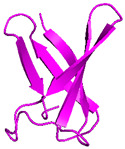	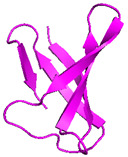	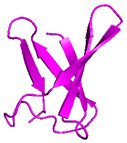	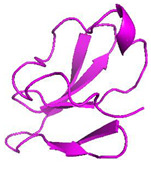	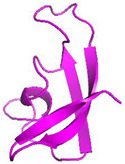
**AF3**	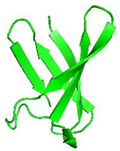	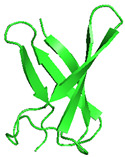	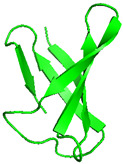	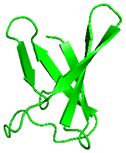	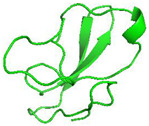	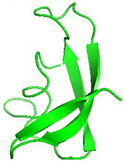
**RMSD to NMR structure**	AF2(1.497); AF3(1.429)	AF2(1.429); AF3(1.395)	AF2(1.004); AF3(0.865)	AF2(1.795); AF3(1.648)	AF2(3.129); AF3(0.868)	AF2(1.244); AF3(1.257)
**Disulfide bond pattern**	*abcabc*: 7-36, 14-43, 28-54	*abcabc*: 7-35, 14-42, 27-53	*abcdabcd*: 26-49, 28-51, (NMR); 7-33, 14-40, 26-49, 28-51 (AF3)	*abcabc*: 6-34, 13-41, 26-52	*abcabdcd*: 3-30, 18-38, 28-54, 49-64	*abbcac*: 9-40, 11-15, 23-49
**Correctness of disulfide bonds**	AF2(Y); AF3(Y)	AF2(N); AF3(Y)	AF2(N); AF3(Y)	AF2(N); AF3(Y)	AF2(N); AF3(Y)	AF2(Y); AF3(Y)

**Table 2:** Docking reliability and structural accuracy scores of PAF and PAF mutants with Ca^2+^, Mg^2+^, and Na^+^ in complexes predicted using AF3. The predicted template modeling (pTM) and interface-predicted template modeling (ipTM) scores are derived from the template modeling (TM) score, which assesses structural accuracy. A pTM score >0.5 suggests that the overall fold may align with the true structure, while ipTM evaluates subunit positioning. ipTM > 0.8 indicates high confidence, <0.6 suggests failure, and 0.6–0.8 represents uncertain predictions [48].

Ions Num	Mg^2+^		2Mg^2+^		3Mg^2+^		4Mg^2+^	
**Score**	**ipTM**	**pTM**	**ipTM**	**pTM**	**ipTM**	**pTM**	**ipTM**	**pTM**
**PAF**	0.78	0.85	0.7	0.85	0.58	0.84	0.55	0.84
**PAF^D19S^**	0.79	0.85	0.71	0.85	0.57	0.84	0.53	0.84
**Ions Num**	**Na^+^**		**2Na^+^**		**3Na^+^**		**4Na^+^**	
**Score**	**ipTM**	**pTM**	**ipTM**	**pTM**	**ipTM**	**pTM**	**ipTM**	**pTM**
**PAF**	0.8	0.85	0.75	0.86	0.69	0.85	0.65	0.85
**PAF^D19S^**	0.8	0.85	0.75	0.85	0.69	0.85	0.65	0.85
**Ions Num**	**Ca^2+^**		**2Ca^2+^**		**3Ca^2+^**		**4Ca^2+^**	
**Score**	**ipTM**	**pTM**	**ipTM**	**pTM**	**ipTM**	**pTM**	**ipTM**	**pTM**
**PAF**	0.74	0.84	0.65	0.85	0.61	0.84	0.57	0.84
**PAF^D19S^**	0.77	0.84	0.69	0.85	0.66	0.85	0.55	0.84
**Ions**	**Ca^2+^ and Mg^2+^**		**Ca^2+^ and Na^+^**		**Na^+^ and Mg^2+^**			
**Score**	**ipTM**	**pTM**	**ipTM**	**pTM**	**ipTM**	**pTM**		
**PAF**	0.78	0.85	0.78	0.85	0.81	0.86		
**PAF^D19S^**	0.78	0.85	0.78	0.85	0.81	0.86		

**Table 3:** MolProbity evaluation of the NMR, AF2, and AF3 structures.

Metric	NMR- PAF	NMR- NFAP	NMR- PAFB	NMR- PAFC	NMR- AFPg	AF2- PAF	AF2- NFAP	AF2- PAFB	AF2- PAFC	AF2- AFPg	AF2- NFAP2	AF3- PAF	AF3- NFAP	AF3- PAFB	AF3- PAFC	AF3- AFPg	AF3- NFAP2
**MolProbity Score**	1.98	3.19	3.52	2.58	4.44	1.86	2.62	2.99	3.49	2.18	1.75	0.673	1.70	1.32	0.99	1.31	1.31
**Clashscore**	1.29	8.84	17.06	3.46	89.22	24.11	33.38	41.82	47.21	39.79	11.01	1.563	13.62	5.88	2.31	4.59	3.53
**Poor rotamers (%)**	35.3	33.43	34.18	21.80	36.34	0	4.57	3.4	5.67	0.78	2.33	0	0.65	0	0	0.78	0.78
**Favored rotamers (%)**	43.2	45.39	40.31	63.72	37.03	100	92.16	92.52	88.65	99.22	93.80	100	99.35	99.32	100	98.45	99.22
**Ramachandran** **Outliers (%)**	1.796	6.36	2.78	1.61	10.87	0	0	5.56	21.50	1.36	0	0	0	0	0	0	0
**Ramachandran** **favored (%)**	97.92	89.18	86.23	92.83	68.52	99.73	96.97	90.12	71.51	97,96	98	99.3	99.39	100	100	100	98.67
**Rama distribution** **Z-score**	−1.88 ±1.04	−3.36 ±0.82	−3.57 ±0.95	−1.70 ±0.79	−5.86 ±1.01	0.72 ±0.95	0.34± 1.03	−1.11 ±1.04	−4.62 ±0.70	−0.85 ±1.06	−1.35 ±1.02	1.32 ±1.03	0.89 ±1.03	1.14 ±1.04	0.56 ±1.05	0.35 ±1.22	−0.73 ±1.1
**Cβ deviations >0.25Å(%)**	0	0	0	0	0	0	0	0	0	0	0	0	0	0	0	0	0
**Bad bonds (%)**	0	0	0	0	0	2.82	2.92	2.47	4.92	3.11	3.64	0	0.14	0	0	0	0
**Bad angles (%)**	0	0	0	0	0	0.8	0.91	1.46	4.28	0.75	0.88	0	0.16	00	0	0	0
**Cis Prolines (Per Chain)**	0/1	0/1	0/1	0/1	0/1	0/1	0/1	0/1	0/1	0/1	1/3	0/1	0/1	0/1	0/1	0/1	1/3
**CaBLAM outliers (%)**	3.81	4.09	3.80	2.86	13.83	0	0	1.90	6.1	0.7	1.4	0	0	0	2.23	0	2.77
**CA Geometry outliers (%)**	1.96	0	0.10	1.67	1.60	0	0	0.64	2.22	0	4.17	0	0	0	0	0	3.47
**Chiral volume outliers**	0	0	0	0	0	0	0	0	0	0	0	0	0	0	0	0	0
**Waters with clashes (%)**	0	0	0	0	0	0	0	0	0	0	0	0	0	0	0	0	0

## Data Availability

The data presented in this study are available in Supplementary Materials.

## Supplementary Materials

The following supporting information can be downloaded at: https://www.mdpi.com/article/10.3390/ijms26031247/s1, Figures S1 and S5: Comparison of AF2 predicted structure with NMR structure of AFPs. Figure S2. Surface electrostatic potential of AF2 structures of PAF and PAF^D19S^. Figures S3, S7 and S8. Ramachadran Analysis of AFPs. Figure S4. Predicted Structures of AFPs from AF Models.; Figure S6. AF3 structure and hydrogen bond interaction distribution of NFAP, AFPg and PAFB. Figure S9. Hydrophilicity of the AFPs AF3 Structure. Table S1: Evaluation Standards for MolProbity Parameters. Table S2. MolProbity Evaluation of NMR and AF Structures of AFPs.
