# Supplementary material for: Small Disulfide Proteins with Antifungal Impact: NMR Experimental Structures as Compared to Models of Alphafold Versions
Source: Int J Mol Sci. 2025 Jan 31;26(3):1247. doi: 10.3390/ijms26031247 (PMC11818080; doi:10.3390/ijms26031247)
Supplement: Supplementary file 1 [file ijms-26-01247-s001.zip › Figure S3a. NMR-PAF-2kcn.pdf]

MolProbity Ramachandran analysis

2kcnH.pdb, all models

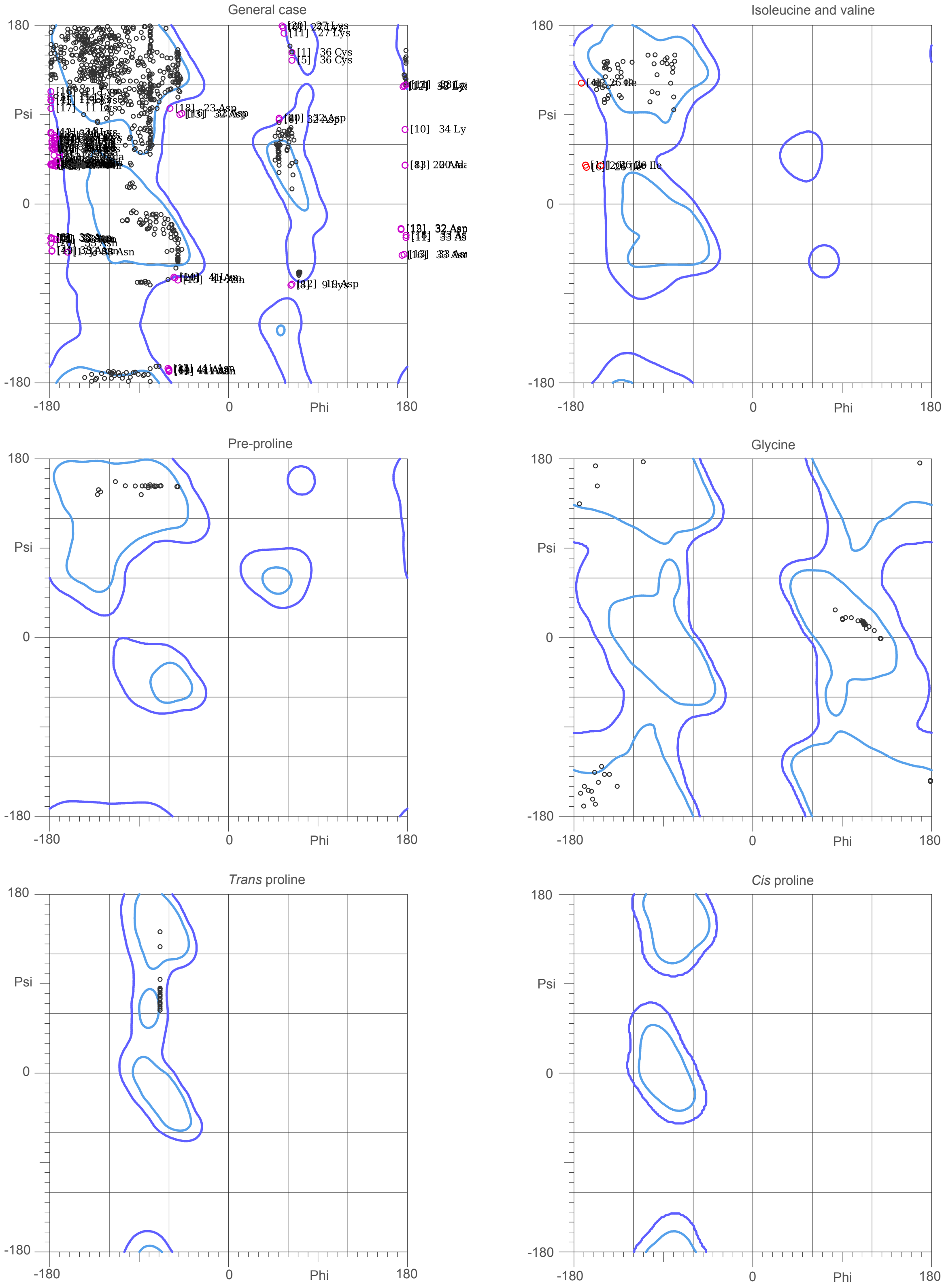

72.4% (767/1060) of all residues were in favored (98%) regions.
92.2% (977/1060) of all residues were in allowed (>99.8%) regions.
This list is truncated; use the MolProbity multi-chart.html for complete list.
There were 83 outliers (phi, psi):

[1] 20 Ala (-174.0, 44.8)
[1] 34 Lys (-179.4, 71.2)
[1] 36 Cys (64.0, 153.3)
[2] 20 Ala (-170.2, 52.1)
[2] 33 Asn (-177.0, -35.9)
[2] 34 Lys (-175.7, 58.0)
[3] 20 Ala (-175.3, 42.3)
[3] 33 Asn (-179.9, -34.7)
[3] 34 Lys (-175.6, 60.3)
[4] 11 Lys (-179.7, 105.3)
[4] 20 Ala (-177.1, 39.9)
[4] 26 Ile (-173.9, 122.5)
[4] 33 Asn (-178.4, -47.4)
[4] 41 Asn (-60.8, -167.2)
[5] 11 Lys (-179.4, 108.7)
[5] 20 Ala (-173.8, 40.9)
[5] 33 Asn (-179.4, -34.8)
[5] 34 Lys (-174.8, 56.2)
[5] 36 Cys (64.5, 145.0)
[6] 20 Ala (-178.4, 40.0)
[6] 26 Ile (-169.0, 37.8)
[6] 27 Lys (55.1, 178.2)
[6] 32 Asp (51.5, 85.5)
[6] 33 Asn (-179.9, -34.9)
[6] 34 Lys (-175.3, 67.8)
[6] 41 Asn (-60.9, -168.4)
[7] 20 Ala (-175.7, 40.0)
[7] 33 Asn (-178.4, -35.5)
[7] 34 Lys (-175.5, 58.6)
[8] 9 Lys (63.9, -82.7)
[8] 20 Ala (178.6, 39.9)
[8] 33 Asn (-174.5, -36.6)
[8] 34 Lys (-176.3, 64.9)
[9] 20 Ala (-176.4, 49.3)
[9] 34 Lys (-177.0, 55.0)
[10] 20 Ala (-178.2, 61.0)
[10] 34 Lys (178.2, 75.5)
[11] 20 Ala (-171.5, 41.5)
[11] 26 Ile (-169.8, 39.7)
[11] 27 Lys (56.6, 172.5)
[11] 33 Asn (179.8, -34.7)
[11] 38 Lys (179.0, 120.9)
[12] 19 Asp (64.1, -81.7)
[12] 20 Ala (-170.8, 47.8)
[12] 32 Asp (174.7, -25.9)
[12] 34 Lys (-179.6, 72.7)
[12] 37 Thr (-174.8, 38.9)
[12] 38 Lys (176.9, 118.0)
[13] 20 Ala (178.9, 39.9)
[13] 32 Asp (-49.1, 90.8)
[13] 33 Asn (177.6, -51.6)
[13] 41 Asn (-61.1, -166.8)
[14] 9 Lys (-55.1, -74.7)
[14] 20 Ala (-179.2, 39.9)
[14] 34 Lys (-175.6, 64.1)
[14] 41 Asn (-60.2, -169.5)
[15] 11 Lys (-179.5, 105.0)
[15] 20 Ala (-178.2, 40.1)
[15] 32 Asp (174.9, -26.2)
[15] 34 Lys (-176.0, 69.8)
[15] 41 Asn (-51.4, -77.4)
[16] 11 Lys (-179.9, 113.3)
[16] 20 Ala (-175.5, 41.3)
[16] 32 Asp (-47.0, 91.3)

MolProbity Ramachandran analysis

2kcnH.pdb, model 1

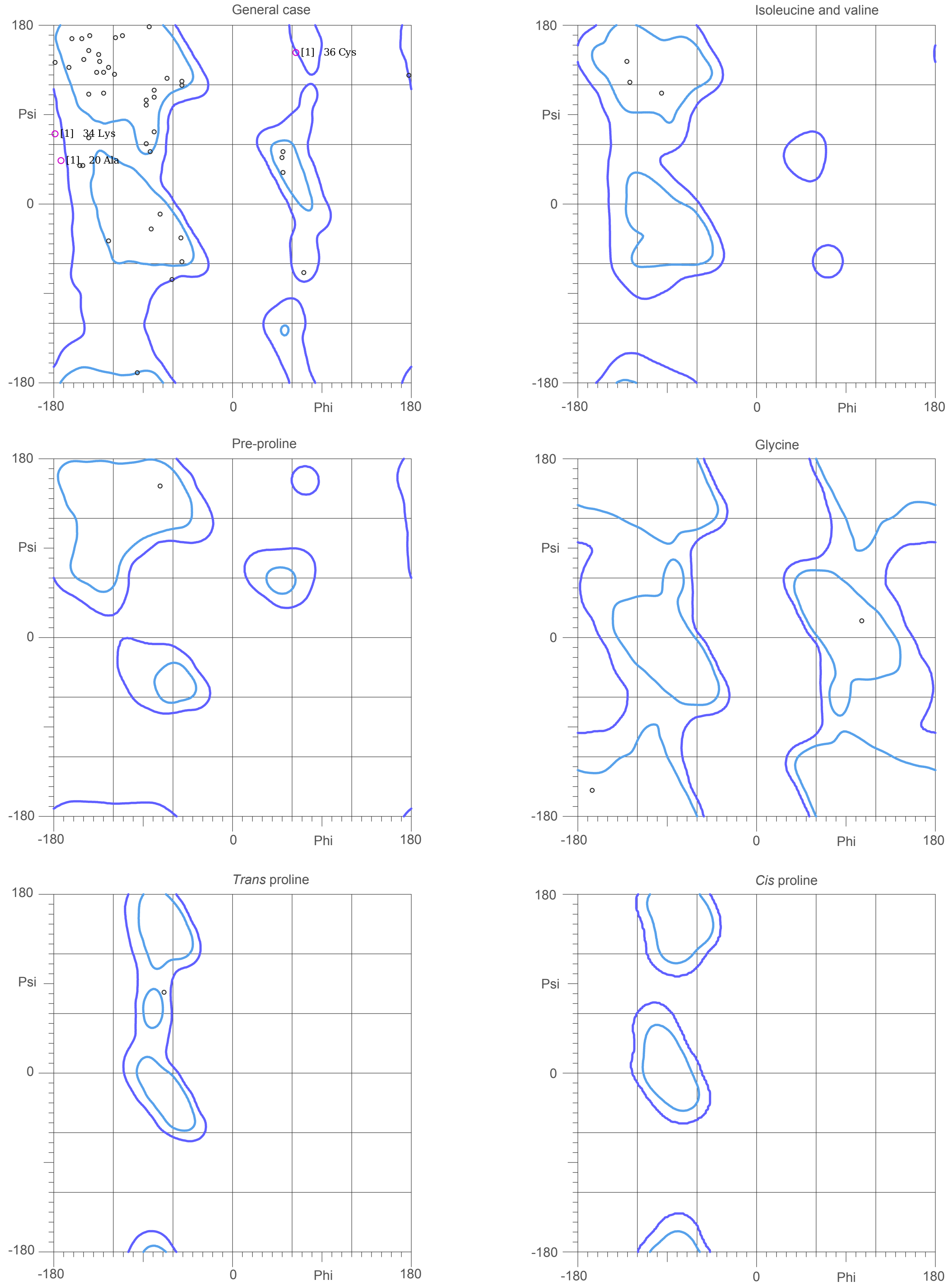

79.2% (42/53) of all residues were in favored (98%) regions.
94.3% (50/53) of all residues were in allowed (>99.8%) regions.

There were 3 outliers (phi, psi):
[1] 20 Ala (-174.0, 44.8)
[1] 34 Lys (-179.4, 71.2)
[1] 36 Cys (64.0, 153.3)

MolProbity Ramachandran analysis

2kcnH.pdb, model 2

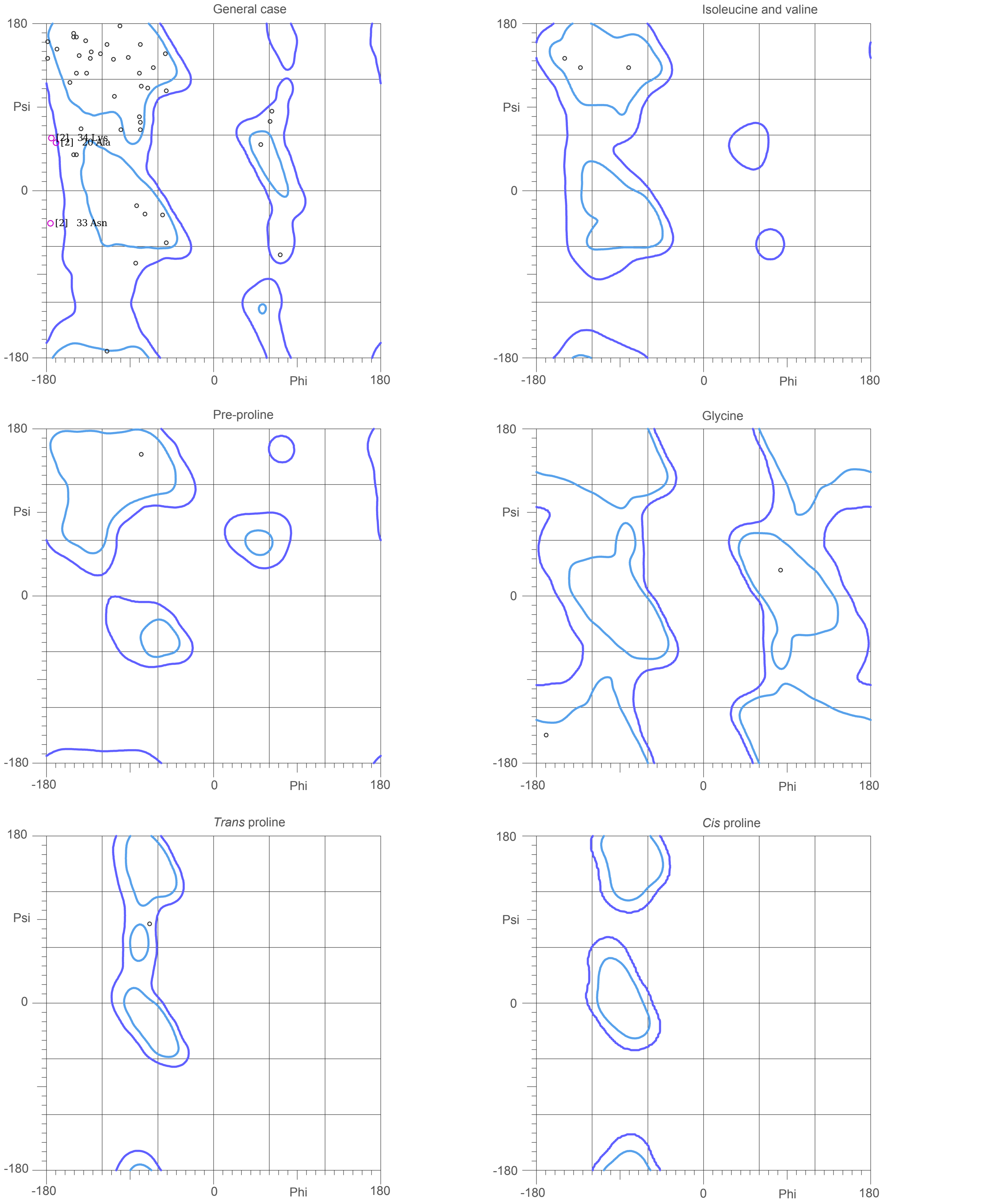

71.7% (38/53) of all residues were in favored (98%) regions.
94.3% (50/53) of all residues were in allowed (>99.8%) regions.

There were 3 outliers (phi, psi):
[2] 20 Ala (-170.2, 52.1)
[2] 33 Asn (-177.0, -35.9)
[2] 34 Lys (-175.7, 58.0)

MolProbity Ramachandran analysis

2kcnH.pdb, model 3

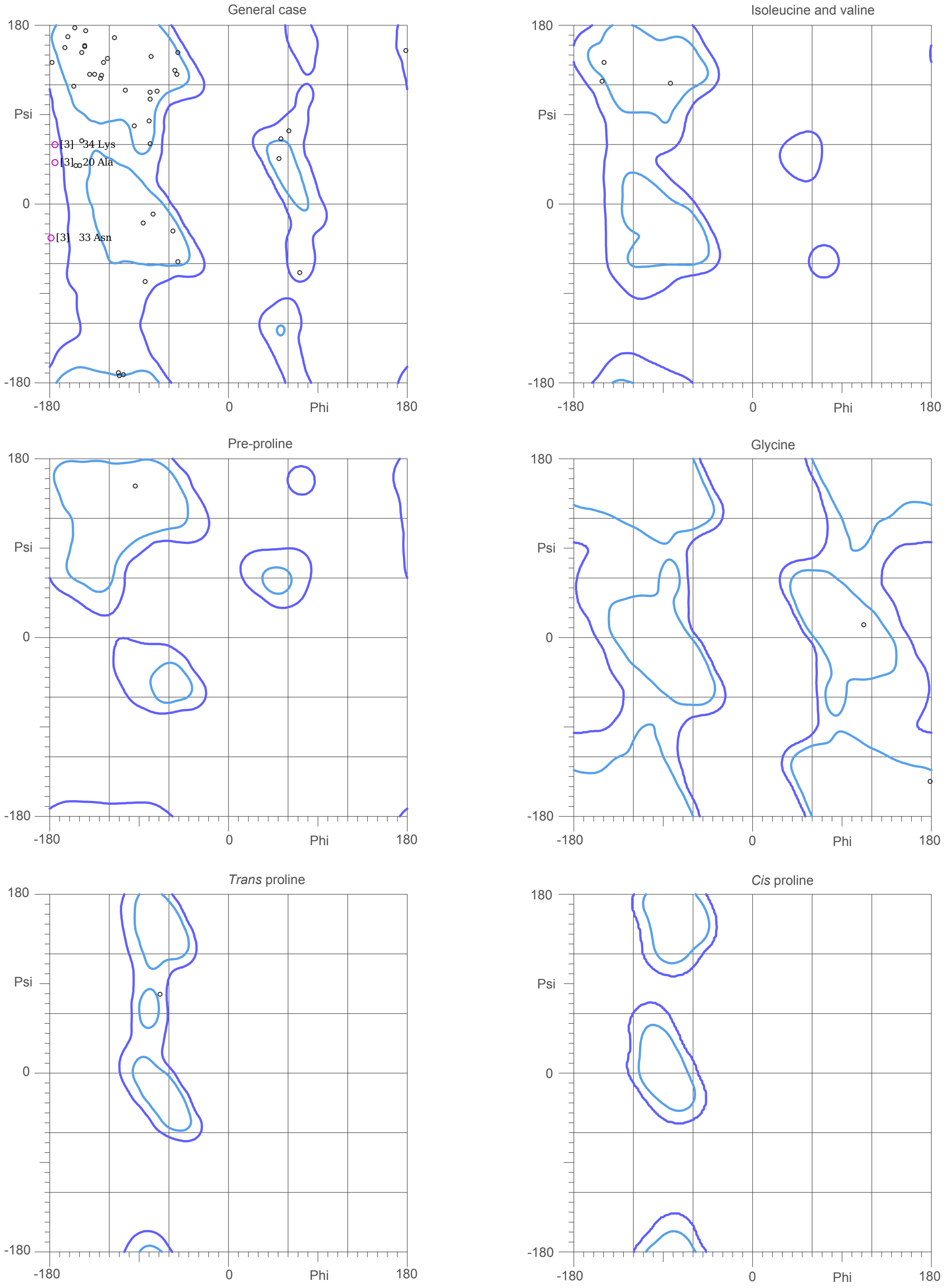

71.7% (38/53) of all residues were in favored (98%) regions.
94.3% (50/53) of all residues were in allowed (>99.8%) regions.

There were 3 outliers (phi, psi):
[3] 20 Ala (-175.3, 42.3)
[3] 33 Asn (-179.9, -34.7)
[3] 34 Lys (-175.6, 60.3)

MolProbity Ramachandran analysis

2kcnH.pdb, model 4

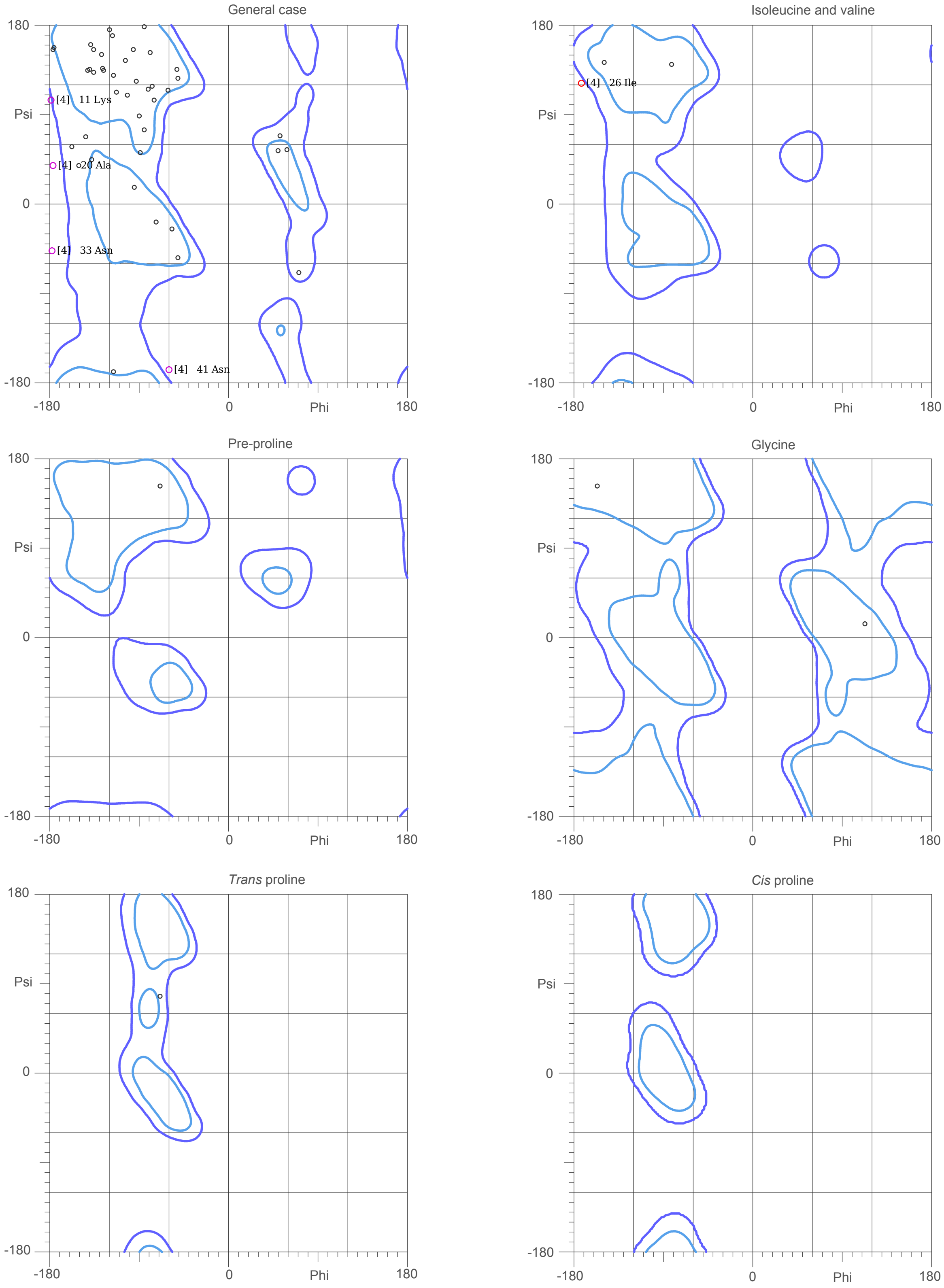

73.6% (39/53) of all residues were in favored (98%) regions.
90.6% (48/53) of all residues were in allowed (>99.8%) regions.

There were 5 outliers (phi, psi):
[4] 11 Lys (-179.7, 105.3)
[4] 20 Ala (-177.1, 39.9)
[4] 26 Ile (-173.9, 122.5)
[4] 33 Asn (-178.4, -47.4)
[4] 41 Asn (-60.8, -167.2)

MolProbity Ramachandran analysis

2kcnH.pdb, model 5

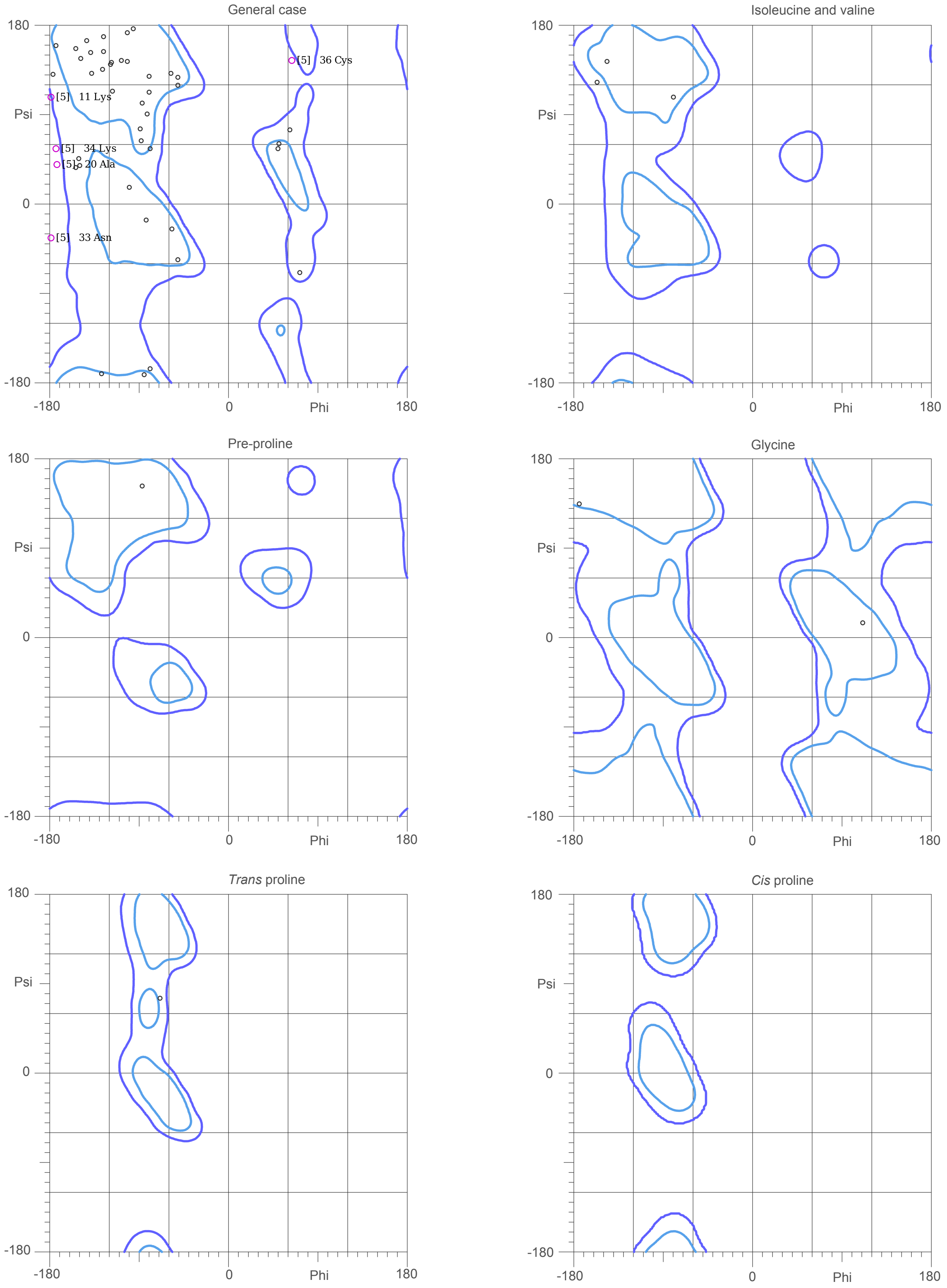

73.6% (39/53) of all residues were in favored (98%) regions.
90.6% (48/53) of all residues were in allowed (>99.8%) regions.

There were 5 outliers (phi, psi):
[5] 11 Lys (-179.4, 108.7)
[5] 20 Ala (-173.8, 40.9)
[5] 33 Asn (-179.4, -34.8)
[5] 34 Lys (-174.8, 56.2)
[5] 36 Cys (64.5, 145.0)

MolProbity Ramachandran analysis

2kcnH.pdb, model 6

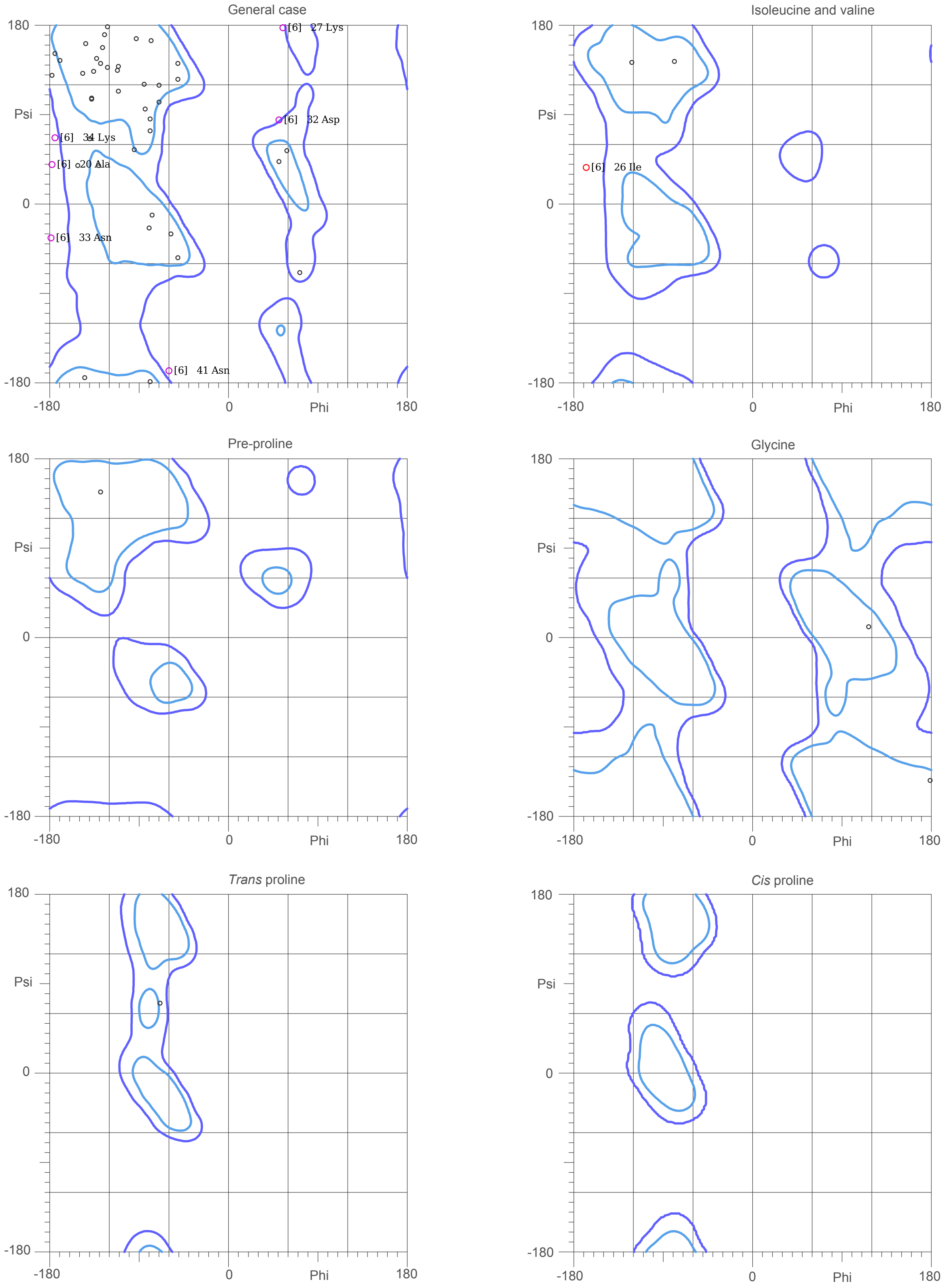

73.6% (39/53) of all residues were in favored (98%) regions.
86.8% (46/53) of all residues were in allowed (>99.8%) regions.

There were 7 outliers (phi, psi):
[6] 20 Ala (-178.4, 40.0)
[6] 26 Ile (-169.0, 37.8)
[6] 27 Lys (55.1, 178.2)
[6] 32 Asp (51.5, 85.5)
[6] 33 Asn (-179.9, -34.9)
[6] 34 Lys (-175.3, 67.8)
[6] 41 Asn (-60.9, -168.4)

http://kinemage.biochem.duke.edu   Lovell, Davis, et al. Proteins 50:437 (2003)

MolProbity Ramachandran analysis

2kcnH.pdb, model 7

General case

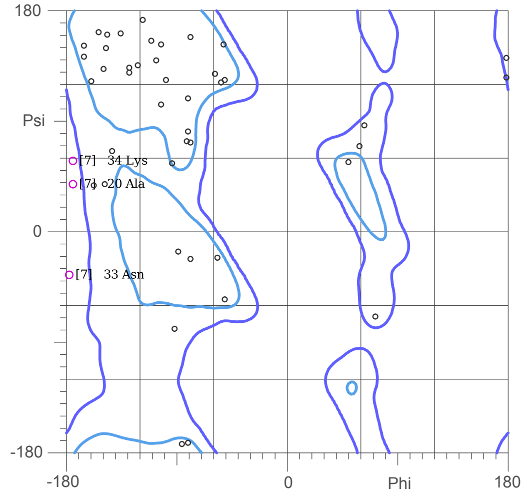

Isoleucine and valine

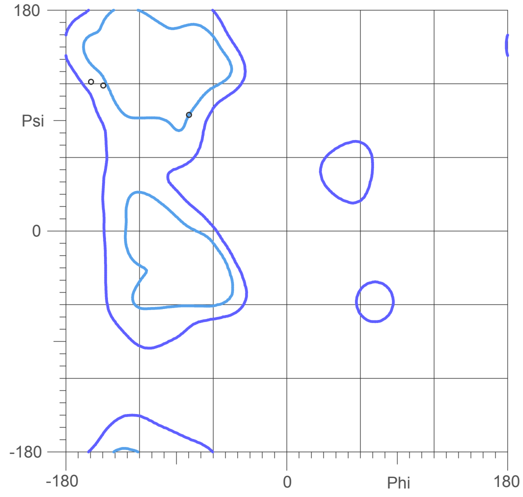

Pre-proline

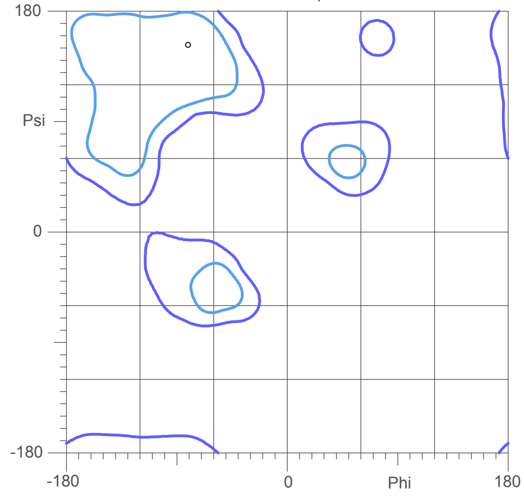

Glycine

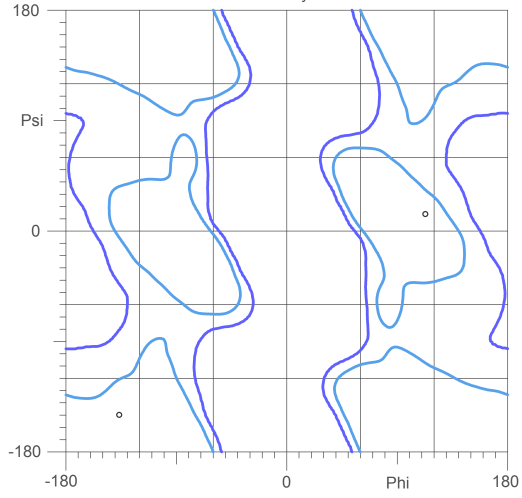

Trans proline

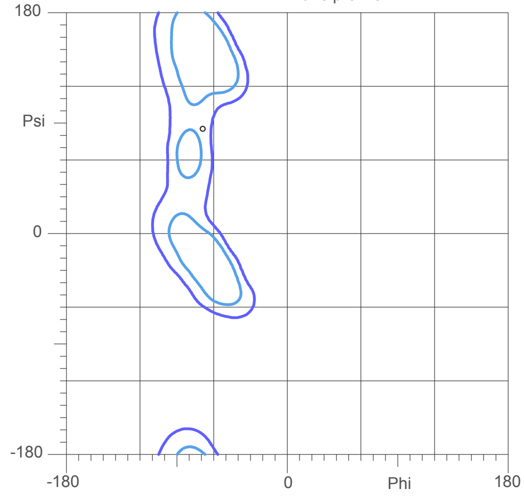

Cis proline

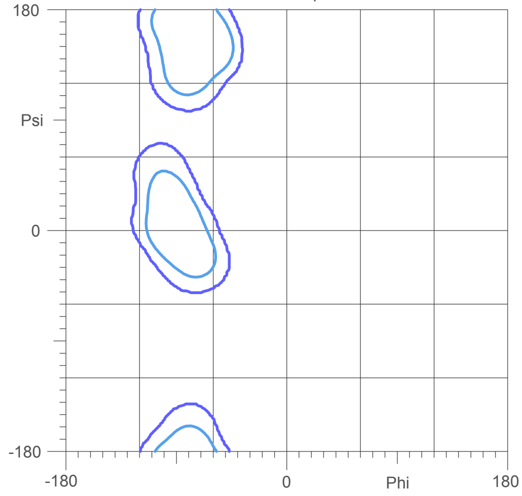

71.7% (38/53) of all residues were in favored (98%) regions.
94.3% (50/53) of all residues were in allowed (>99.8%) regions.

There were 3 outliers (phi, psi):
[7] 20 Ala (-175.7, 40.0)
[7] 33 Asn (-178.4, -35.5)
[7] 34 Lys (-175.5, 58.6)

MolProbity Ramachandran analysis

2kcnH.pdb, model 8

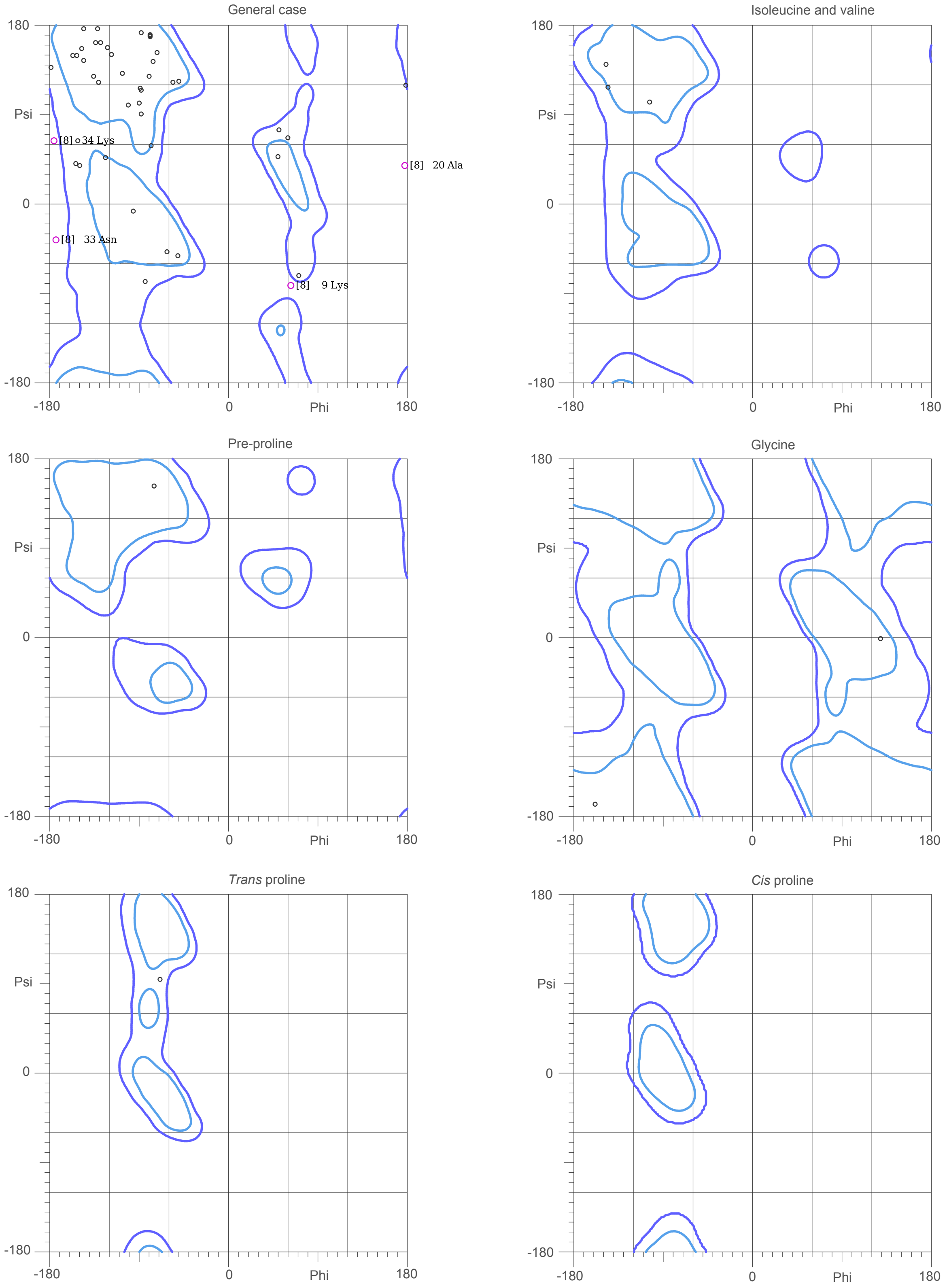

71.7% (38/53) of all residues were in favored (98%) regions.
92.5% (49/53) of all residues were in allowed (>99.8%) regions.

There were 4 outliers (phi, psi):
[8] 9 Lys (63.9, -82.7)
[8] 20 Ala (178.6, 39.9)
[8] 33 Asn (-174.5, -36.6)
[8] 34 Lys (-176.3, 64.9)

MolProbity Ramachandran analysis

2kcnH.pdb, model 9

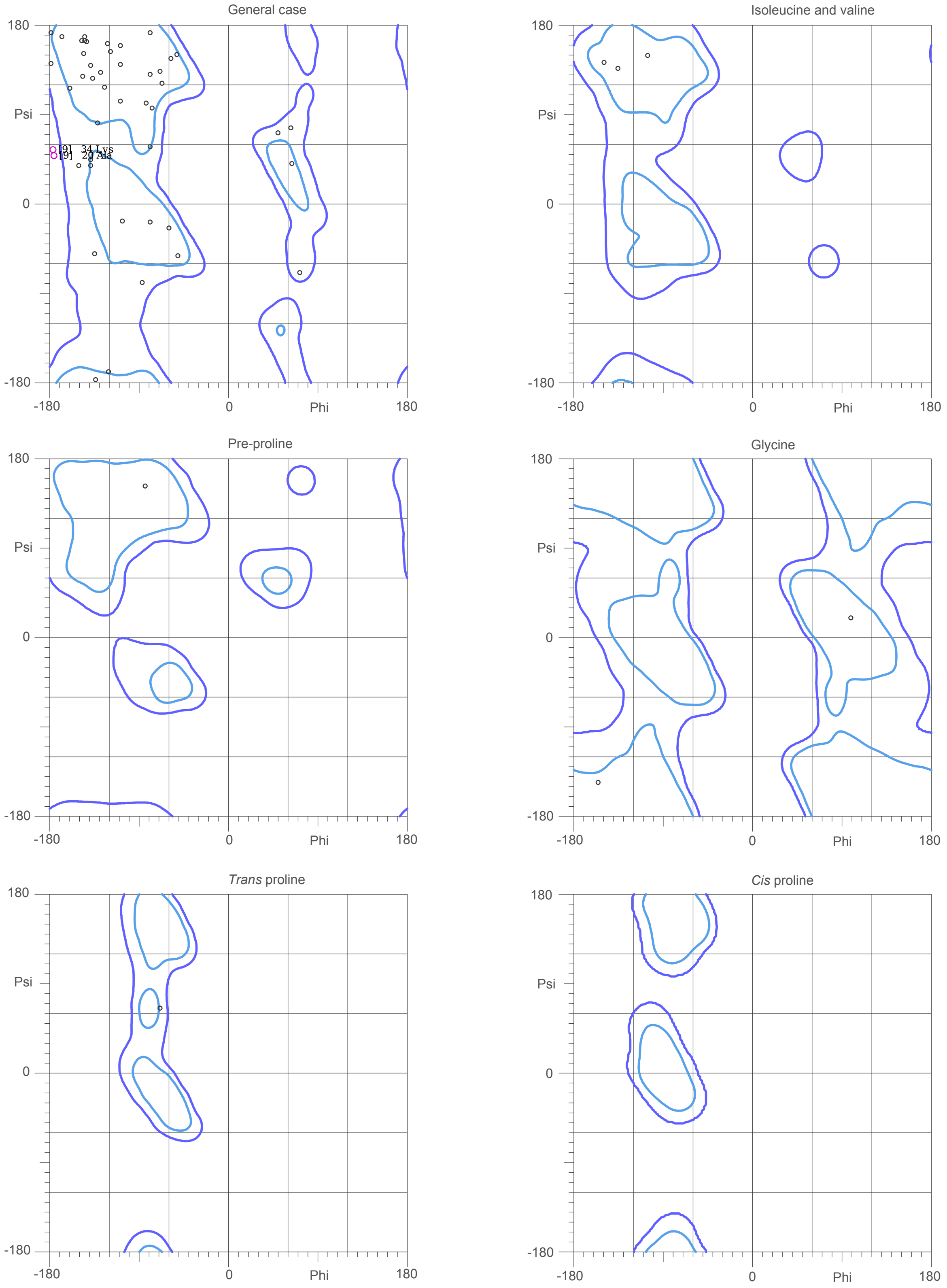

73.6% (39/53) of all residues were in favored (98%) regions.
96.2% (51/53) of all residues were in allowed (>99.8%) regions.

There were 2 outliers (phi, psi):
[9] 20 Ala (-176.4, 49.3)
[9] 34 Lys (-177.0, 55.0)

MolProbity Ramachandran analysis

2kcnH.pdb, model 10

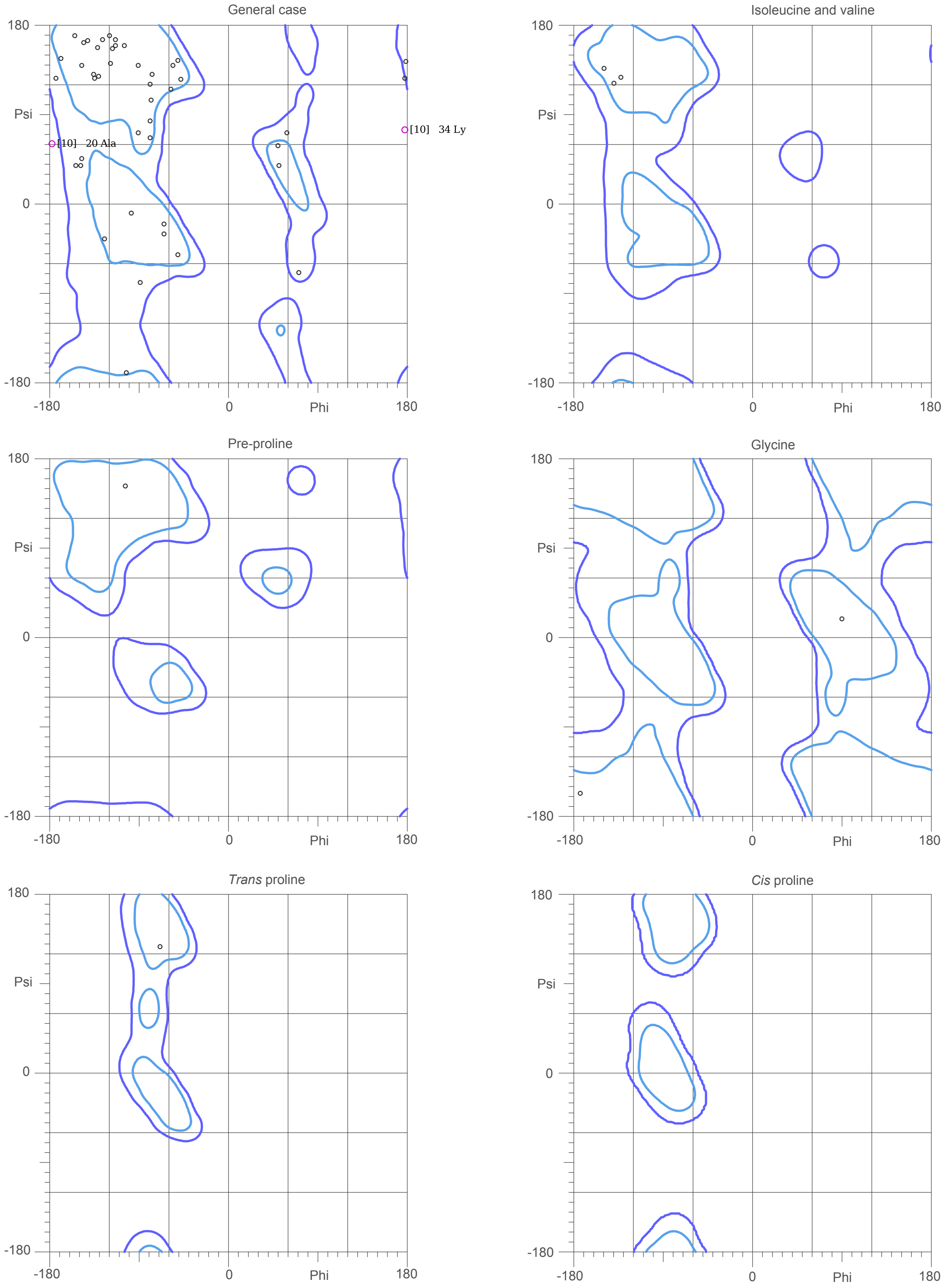

77.4% (41/53) of all residues were in favored (98%) regions.
96.2% (51/53) of all residues were in allowed (>99.8%) regions.

There were 2 outliers (phi, psi):
[10] 20 Ala (-178.2, 61.0)
[10] 34 Lys (178.2, 75.5)

MolProbity Ramachandran analysis

2kcnH.pdb, model 11

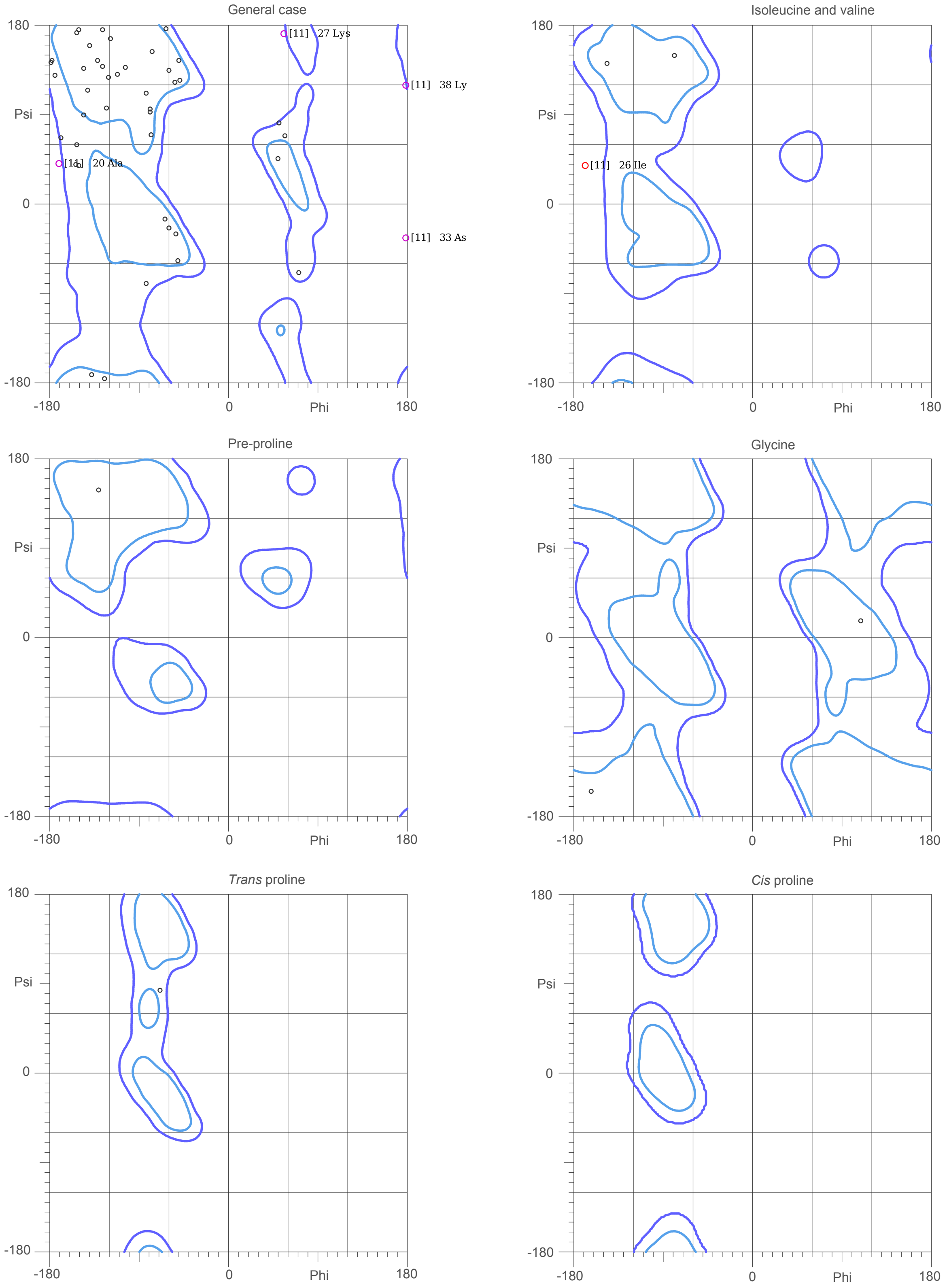

64.2% (34/53) of all residues were in favored (98%) regions.
90.6% (48/53) of all residues were in allowed (>99.8%) regions.

There were 5 outliers (phi, psi):
- [11] 20 Ala (-171.5, 41.5)
- [11] 26 Ile (-169.8, 39.7)
- [11] 27 Lys (56.6, 172.5)
- [11] 33 Asn (179.8, -34.7)
- [11] 38 Lys (179.0, 120.9)

MolProbity Ramachandran analysis

2kcnH.pdb, model 12

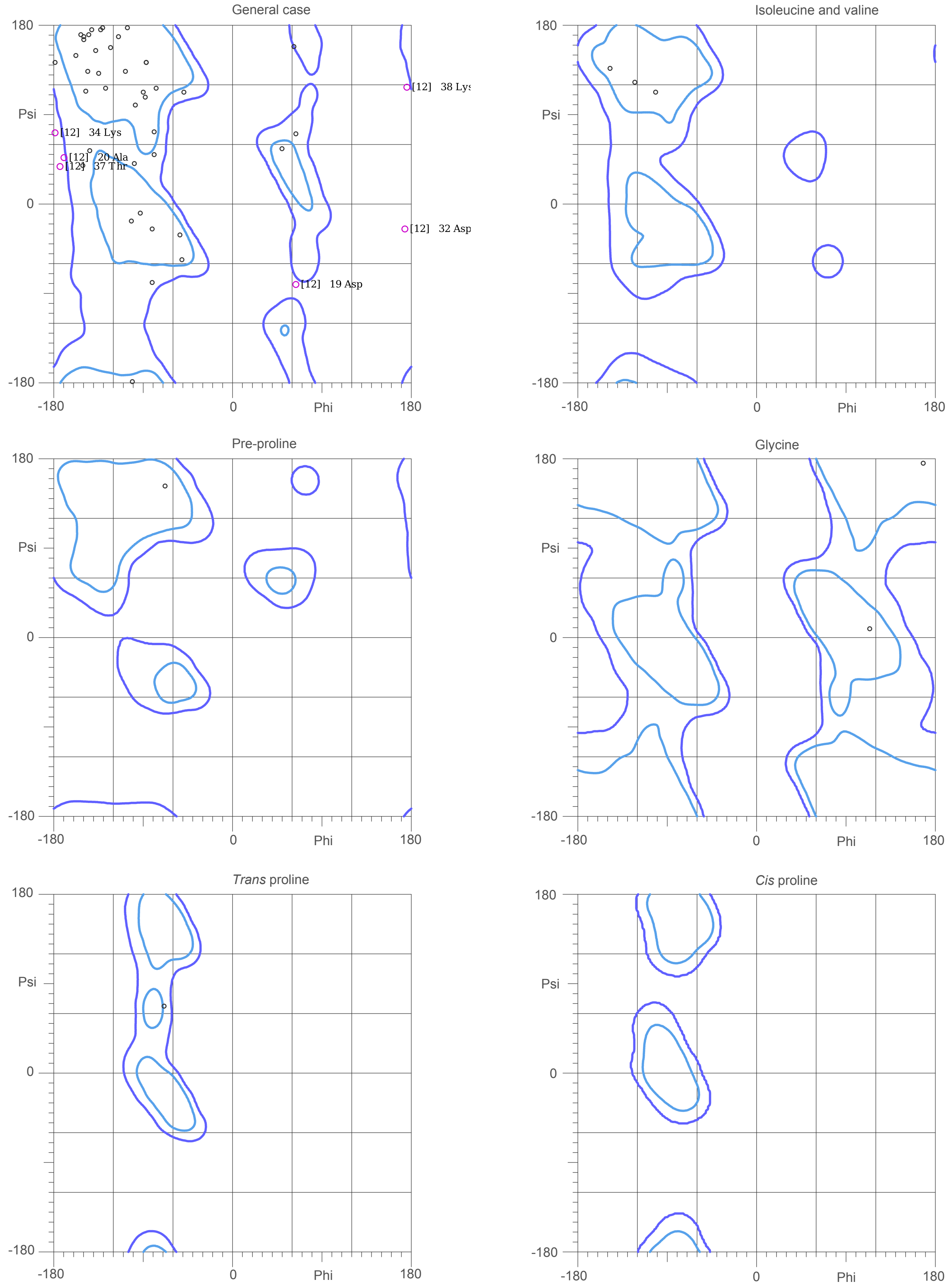

69.8% (37/53) of all residues were in favored (98%) regions.
88.7% (47/53) of all residues were in allowed (>99.8%) regions.

There were 6 outliers (phi, psi):
[12] 19 Asp (64.1, -81.7)
[12] 20 Ala (-170.8, 47.8)
[12] 32 Asp (174.7, -25.9)
[12] 34 Lys (-179.6, 72.7)
[12] 37 Thr (-174.8, 38.9)
[12] 38 Lys (176.9, 118.0)

MolProbity Ramachandran analysis

2kcnH.pdb, model 13

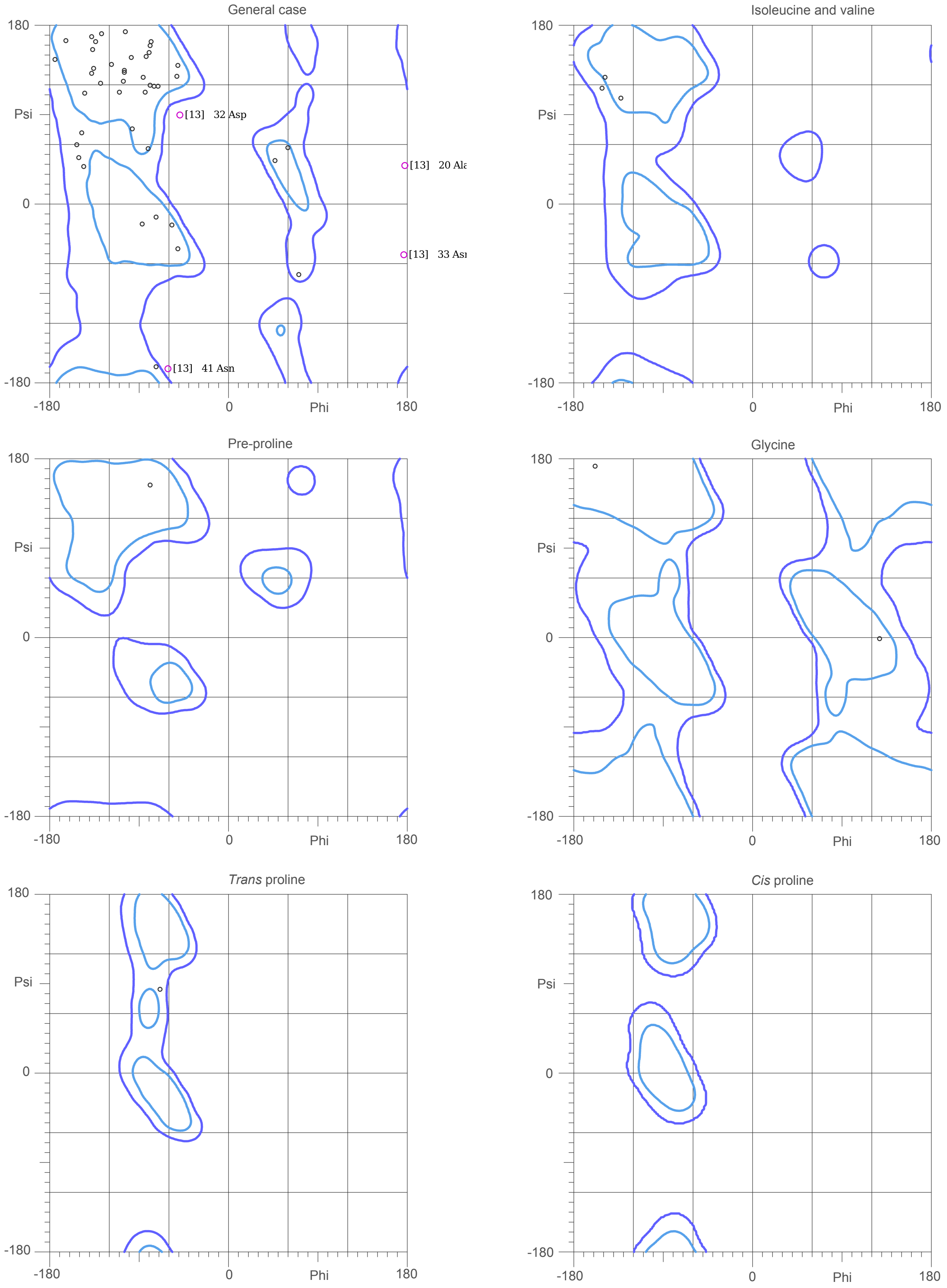

75.5% (40/53) of all residues were in favored (98%) regions.
92.5% (49/53) of all residues were in allowed (>99.8%) regions.

There were 4 outliers (phi, psi):
[13] 20 Ala (178.9, 39.9)
[13] 32 Asp (-49.1, 90.8)
[13] 33 Asn (177.6, -51.6)
[13] 41 Asn (-61.1, -166.8)

MolProbity Ramachandran analysis

2kcnH.pdb, model 14

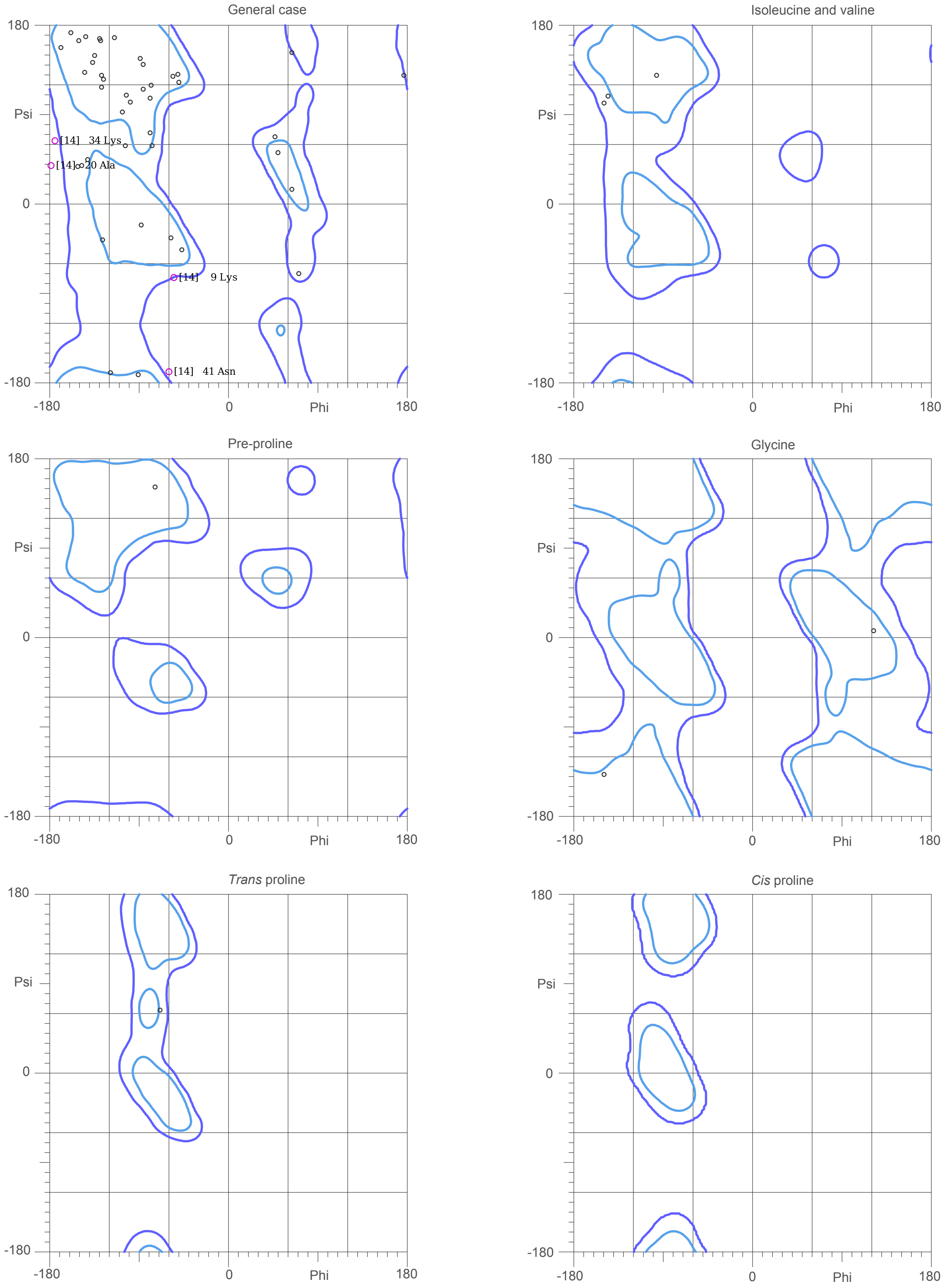

69.8% (37/53) of all residues were in favored (98%) regions.
92.5% (49/53) of all residues were in allowed (>99.8%) regions.

There were 4 outliers (phi, psi):
[14] 9 Lys (-55.1, -74.7)
[14] 20 Ala (-179.2, 39.9)
[14] 34 Lys (-175.6, 64.1)
[14] 41 Asn (-60.2, -169.5)

http://kinemage.biochem.duke.edu

Lovell, Davis, et al. Proteins 50:437 (2003)

MolProbity Ramachandran analysis

2kcnH.pdb, model 15

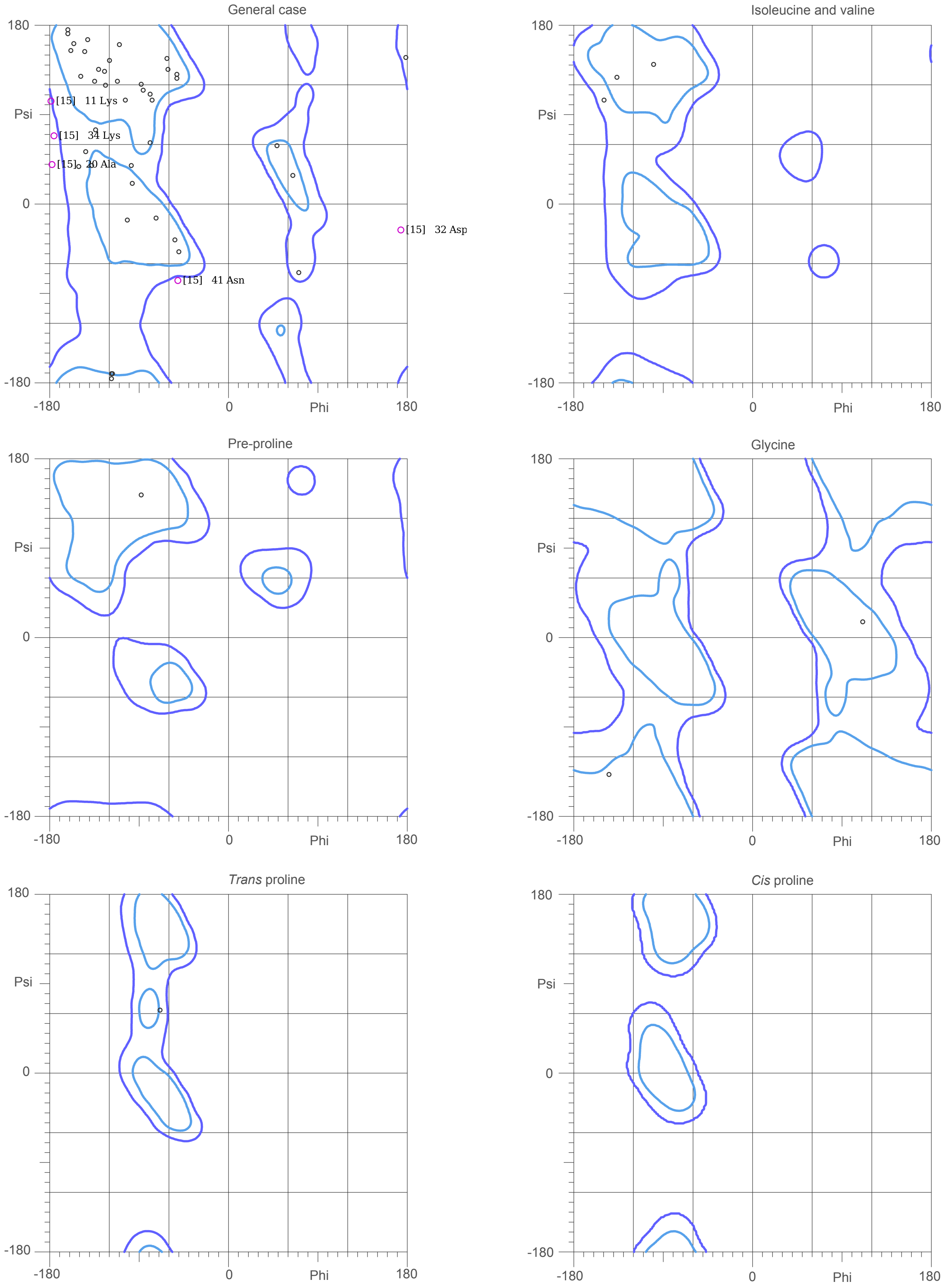

75.5% (40/53) of all residues were in favored (98%) regions.
90.6% (48/53) of all residues were in allowed (>99.8%) regions.

There were 5 outliers (phi, psi):
[15] 11 Lys (-179.5, 105.0)
[15] 20 Ala (-178.2, 40.1)
[15] 32 Asp (174.9, -26.2)
[15] 34 Lys (-176.0, 69.8)
[15] 41 Asn (-51.4, -77.4)

MolProbity Ramachandran analysis

2kcnH.pdb, model 16

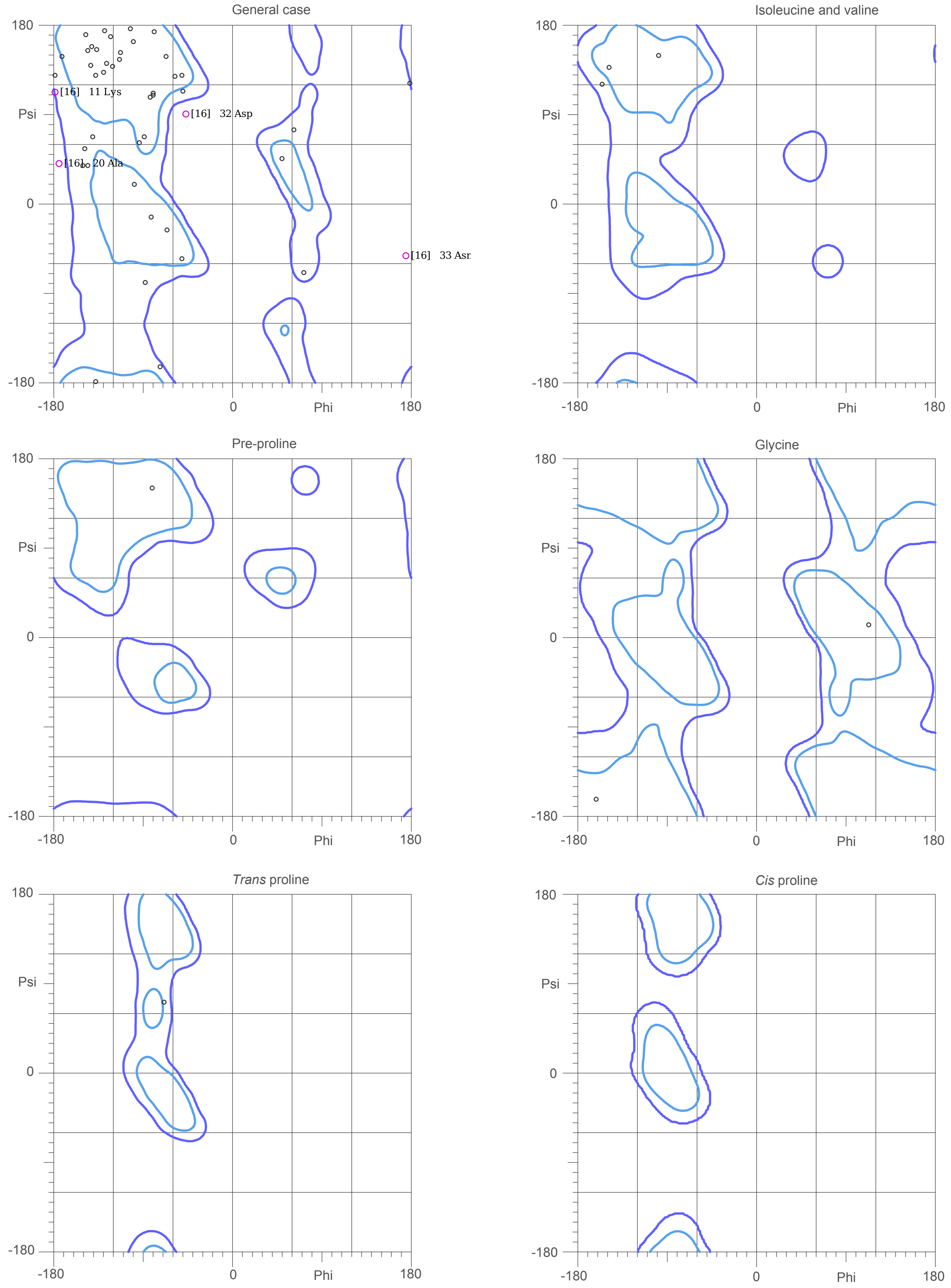

67.9% (36/53) of all residues were in favored (98%) regions.
92.5% (49/53) of all residues were in allowed (>99.8%) regions.

There were 4 outliers (phi, psi):
[16] 11 Lys (-179.9, 113.3)
[16] 20 Ala (-175.5, 41.3)
[16] 32 Asp (-47.0, 91.3)
[16] 33 Asn (175.9, -52.6)

MolProbity Ramachandran analysis

2kcnH.pdb, model 17

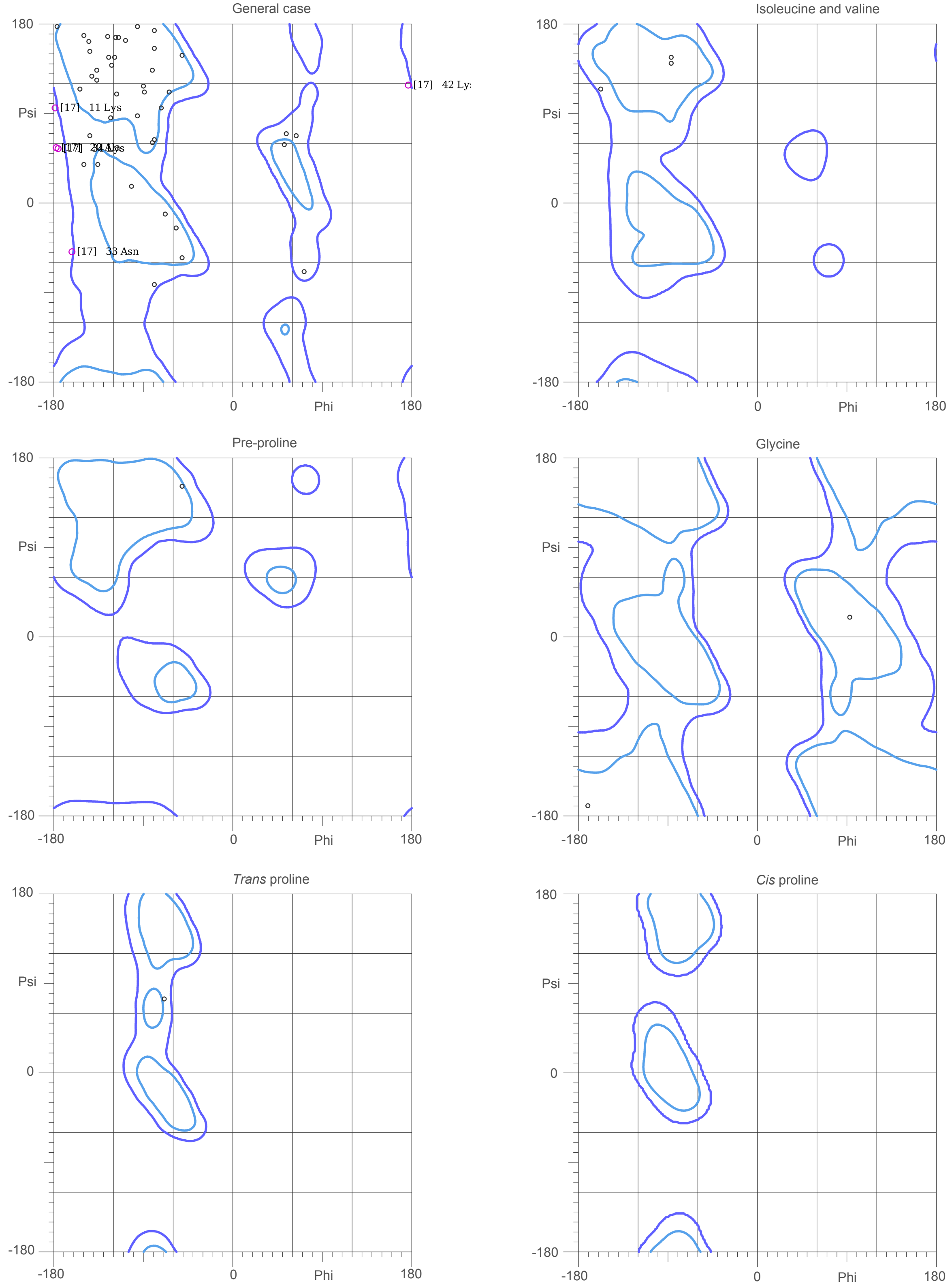

73.6% (39/53) of all residues were in favored (98%) regions.
90.6% (48/53) of all residues were in allowed (>99.8%) regions.

There were 5 outliers (phi, psi):

[17] 11 Lys (-179.5, 96.4)
[17] 20 Ala (-178.5, 56.6)
[17] 33 Asn (-162.3, -49.7)
[17] 34 Lys (-176.9, 55.5)
[17] 42 Lys (177.7, 119.1)

MolProbity Ramachandran analysis

2kcnH.pdb, model 18

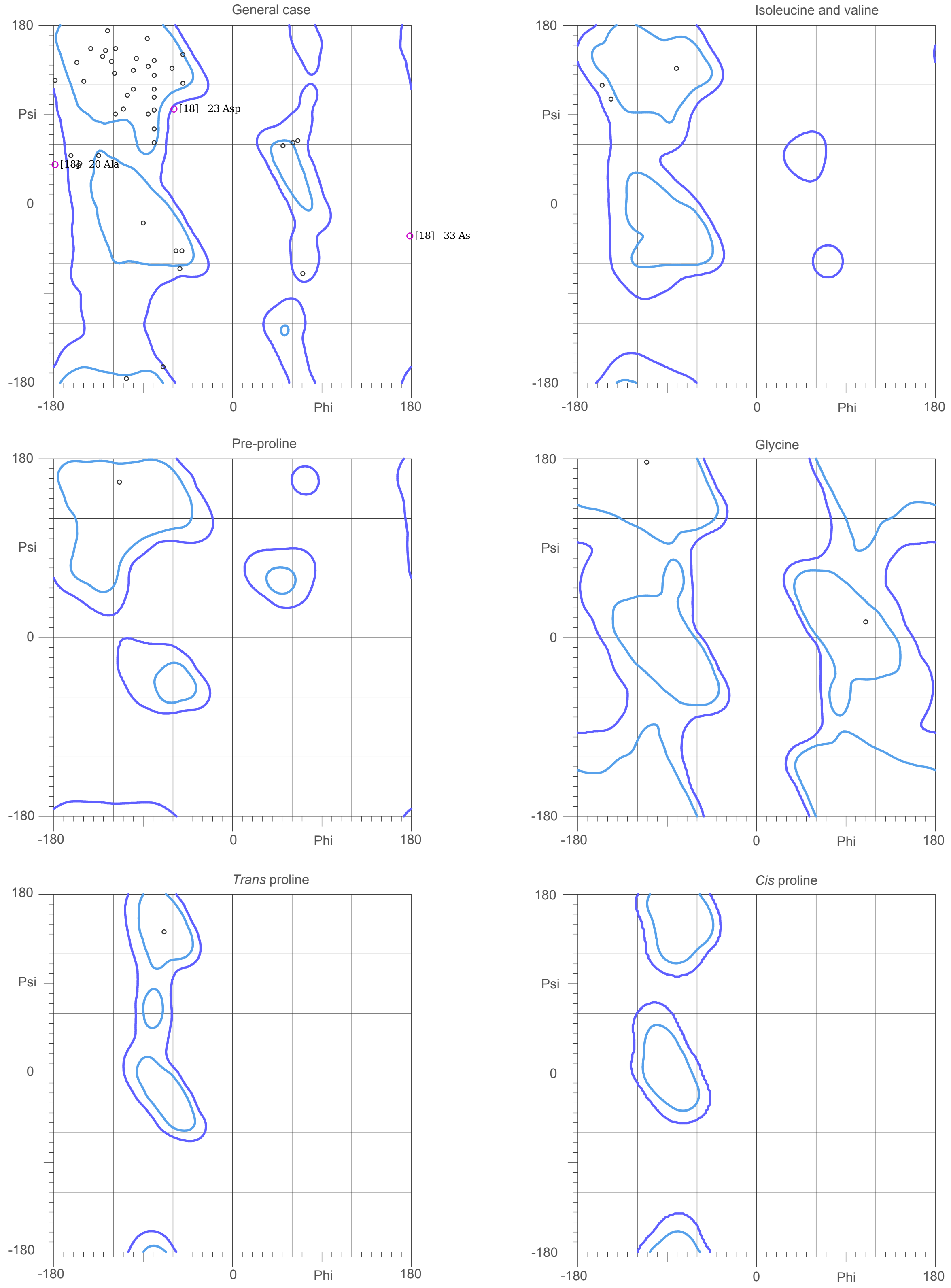

73.6% (39/53) of all residues were in favored (98%) regions.
94.3% (50/53) of all residues were in allowed (>99.8%) regions.

There were 3 outliers (phi, psi):
[18] 20 Ala (-179.4, 41.0)
[18] 23 Asp (-59.9, 97.0)
[18] 33 Asn (179.9, -32.8)

MolProbity Ramachandran analysis

2kcnH.pdb, model 19

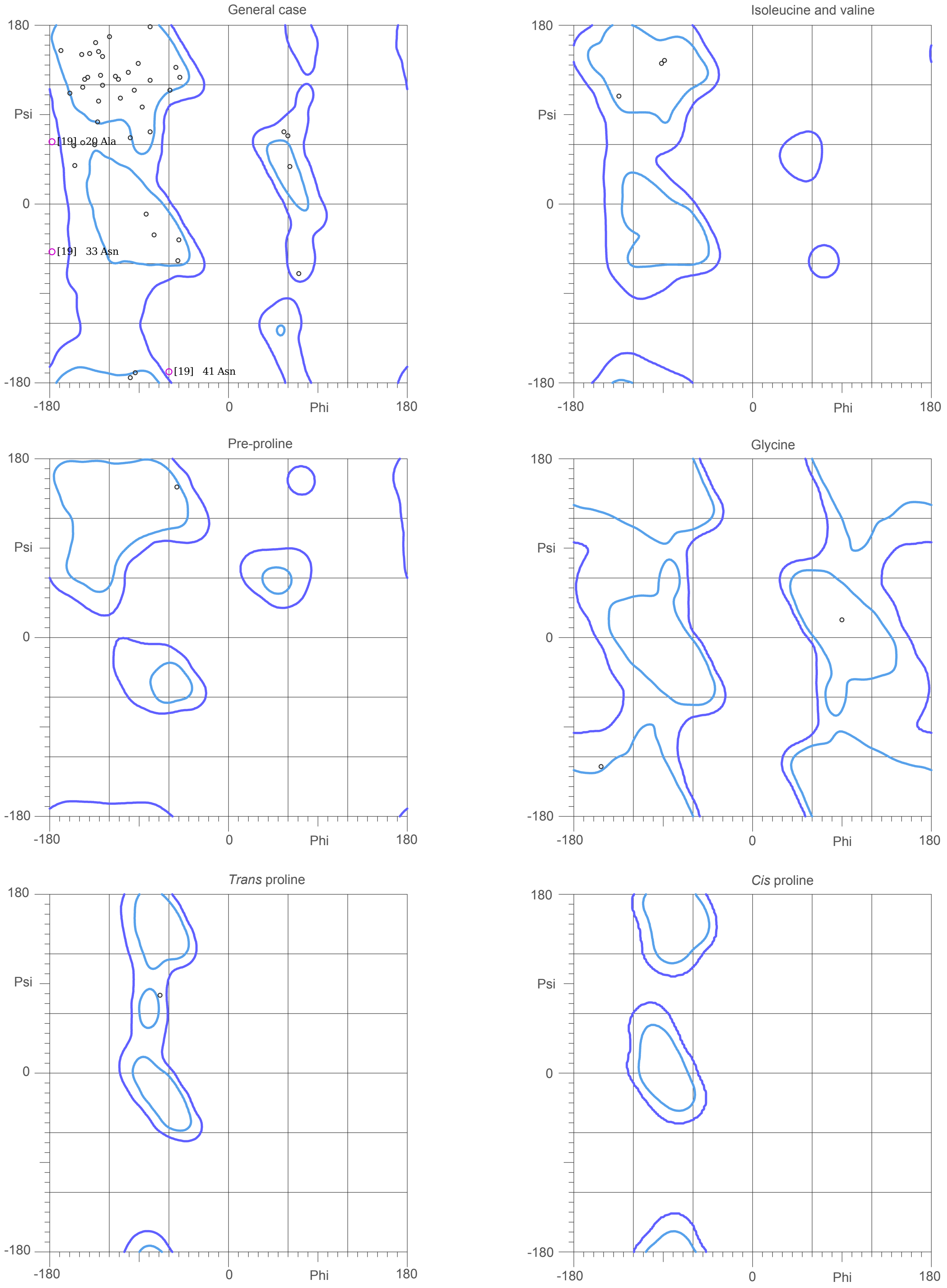

73.6% (39/53) of all residues were in favored (98%) regions.
94.3% (50/53) of all residues were in allowed (>99.8%) regions.

There were 3 outliers (phi, psi):
[19] 20 Ala (-178.9, 63.6)
[19] 33 Asn (-178.4, -48.2)
[19] 41 Asn (-60.3, -169.8)

MolProbity Ramachandran analysis

2kcnH.pdb, model 20

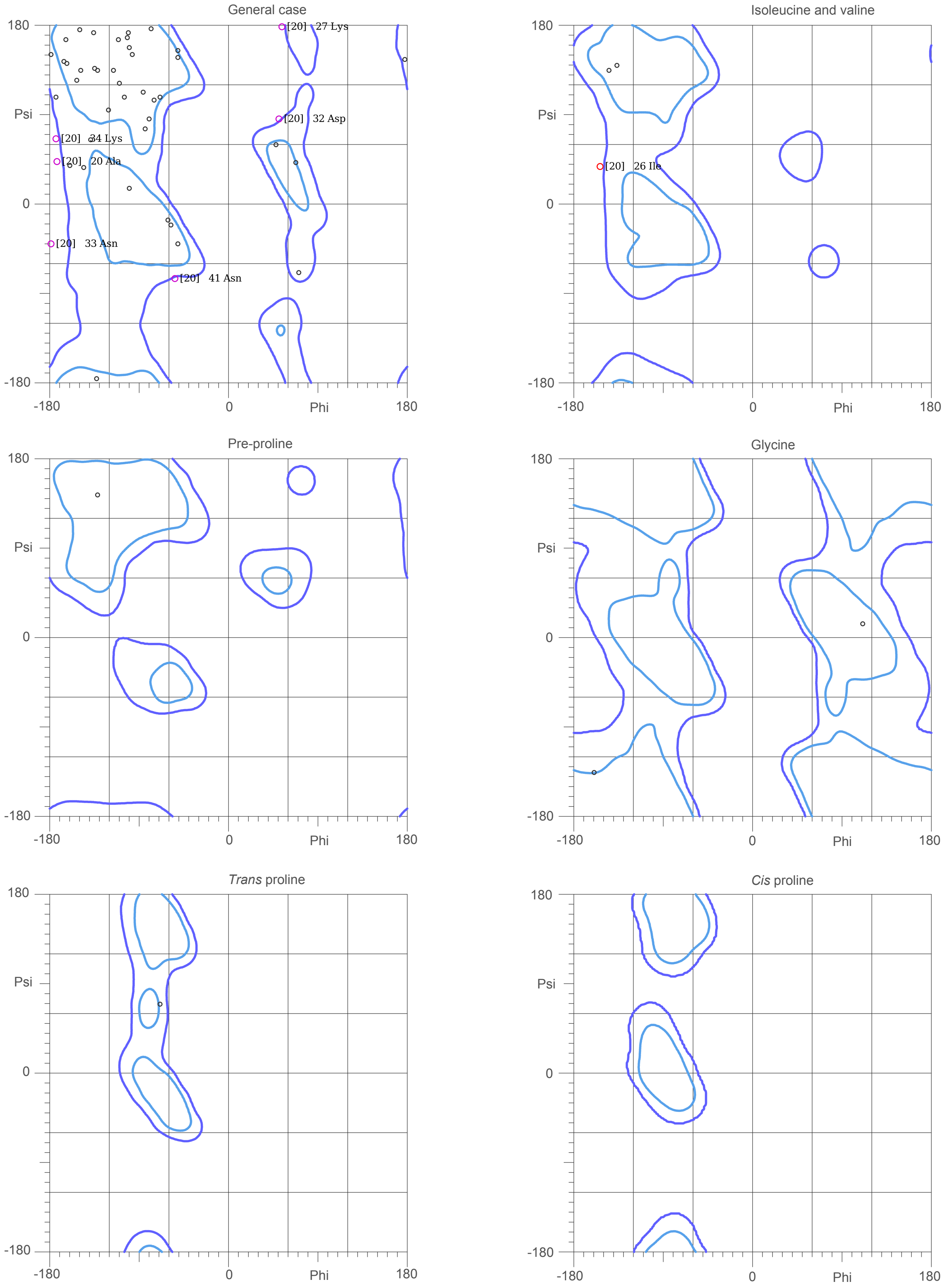

66.0% (35/53) of all residues were in favored (98%) regions.
86.8% (46/53) of all residues were in allowed (>99.8%) regions.

There were 7 outliers (phi, psi):
- [20] 20 Ala (-173.8, 43.8)
- [20] 26 Ile (-154.1, 38.6)
- [20] 27 Lys (54.6, 179.8)
- [20] 32 Asp (51.4, 86.2)
- [20] 33 Asn (-179.3, -40.2)
- [20] 34 Lys (-175.0, 66.5)
- [20] 41 Asn (-54.8, -75.6)

http://kinemage.biochem.duke.edu

Lovell, Davis, et al. Proteins 50:437 (2003)
