# Supplementary material for: Small Disulfide Proteins with Antifungal Impact: NMR Experimental Structures as Compared to Models of Alphafold Versions
Source: Int J Mol Sci. 2025 Jan 31;26(3):1247. doi: 10.3390/ijms26031247 (PMC11818080; doi:10.3390/ijms26031247)
Supplement: Supplementary file 1 [file ijms-26-01247-s001.zip › Figure S3a. NMR-PAF-2mhv.pdf]

MolProbity Ramachandran analysis

2mhvH.pdb, all models

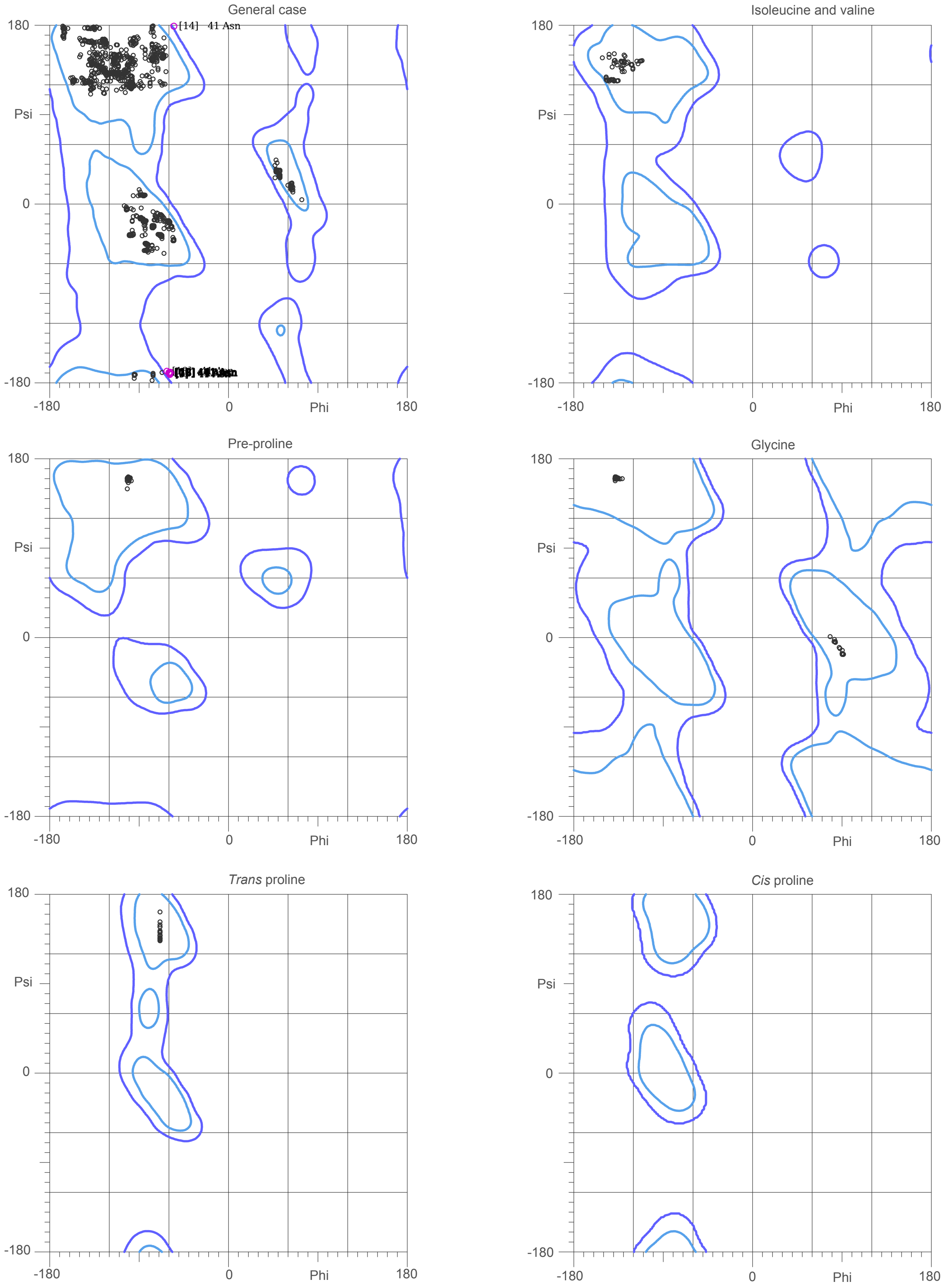

97.9% (1038/1060) of all residues were in favored (98%) regions.
98.2% (1041/1060) of all residues were in allowed (>99.8%) regions.

There were 19 outliers (phi, psi):

[1] 41 Asn (-59.4, -171.1)
[2] 41 Asn (-59.3, -170.9)
[3] 41 Asn (-59.6, -171.4)
[4] 41 Asn (-59.1, -171.0)
[5] 41 Asn (-59.7, -171.2)
[6] 41 Asn (-60.3, -170.9)
[7] 41 Asn (-59.2, -170.9)
[9] 41 Asn (-59.1, -172.5)
[10] 41 Asn (-59.3, -170.7)
[11] 41 Asn (-59.5, -171.3)
[12] 41 Asn (-58.9, -170.2)
[13] 41 Asn (-59.2, -171.3)
[14] 41 Asn (-55.5, 179.0)
[15] 41 Asn (-58.2, -171.3)
[16] 41 Asn (-59.4, -170.7)
[17] 41 Asn (-58.6, -171.9)
[18] 41 Asn (-62.4, -169.5)
[19] 41 Asn (-59.6, -170.3)
[20] 41 Asn (-59.1, -171.4)

MolProbity Ramachandran analysis

2mhvH.pdb, model 1

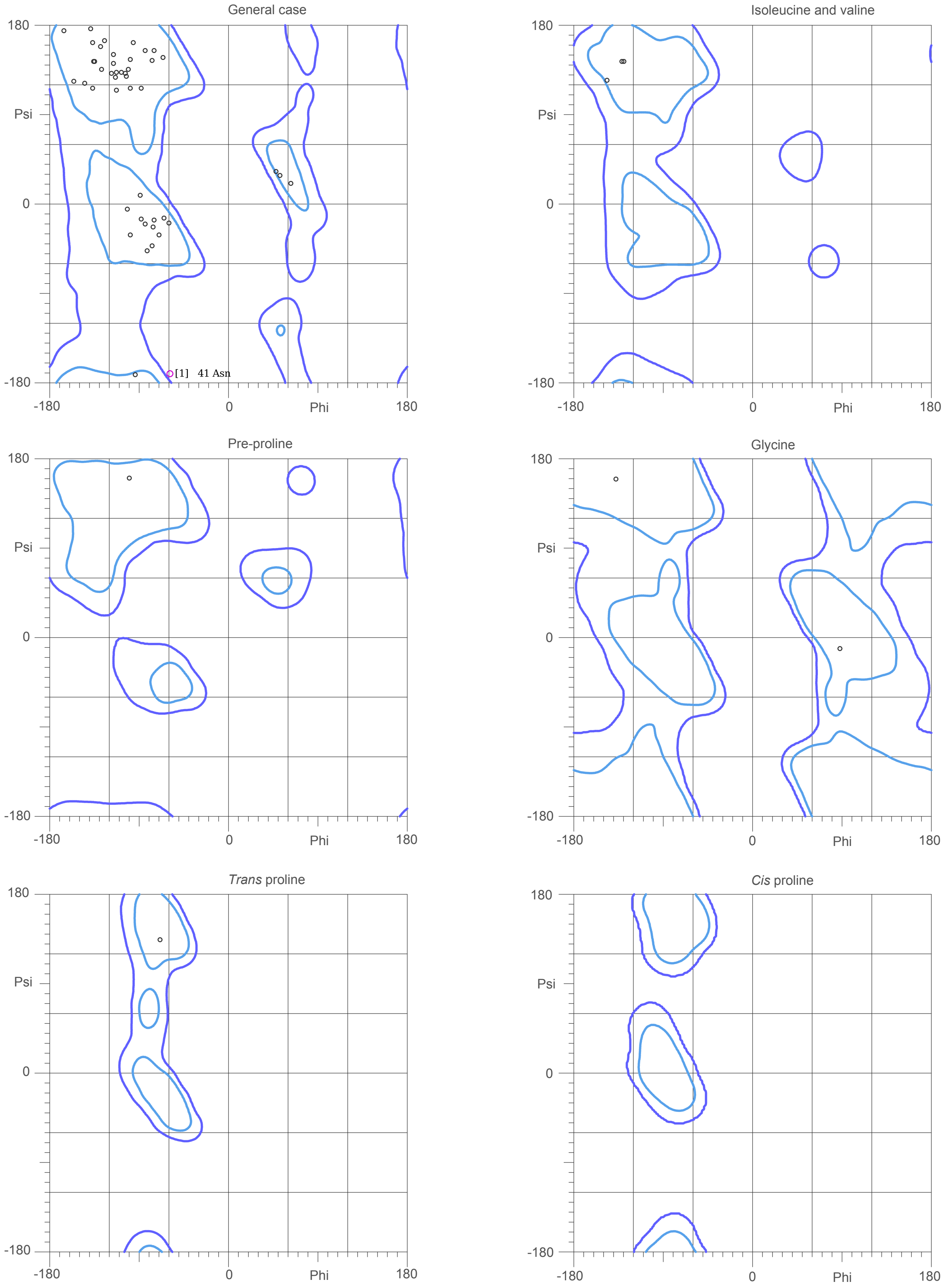

98.1% (52/53) of all residues were in favored (98%) regions.
98.1% (52/53) of all residues were in allowed (>99.8%) regions.

There were 1 outliers (phi, psi):
[1] 41 Asn (-59.4, -171.1)

MolProbity Ramachandran analysis

2mhvH.pdb, model 2

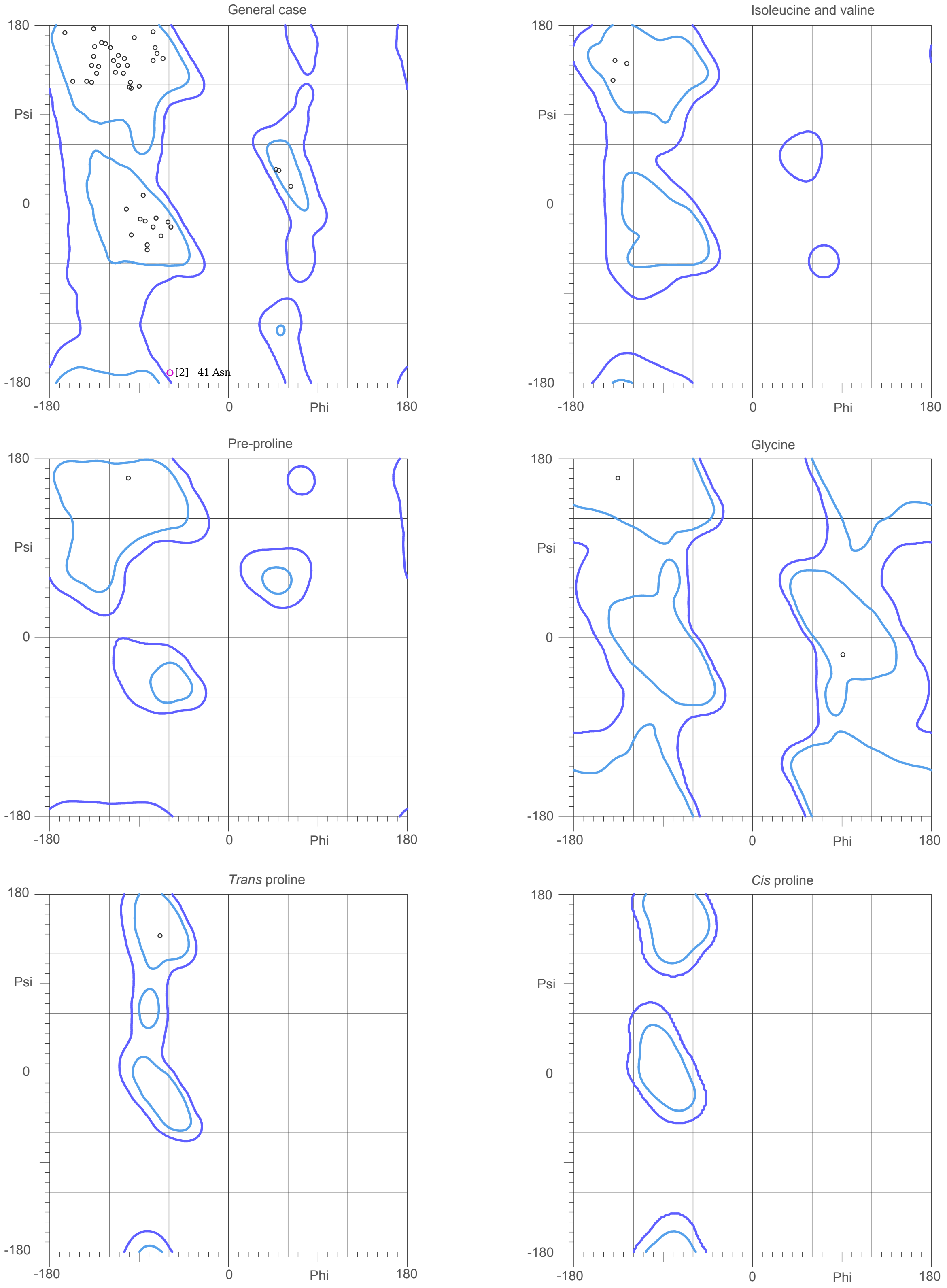

98.1% (52/53) of all residues were in favored (98%) regions.
98.1% (52/53) of all residues were in allowed (>99.8%) regions.

There were 1 outliers (phi, psi):
[2] 41 Asn (-59.3, -170.9)

MolProbity Ramachandran analysis

2mhvH.pdb, model 3

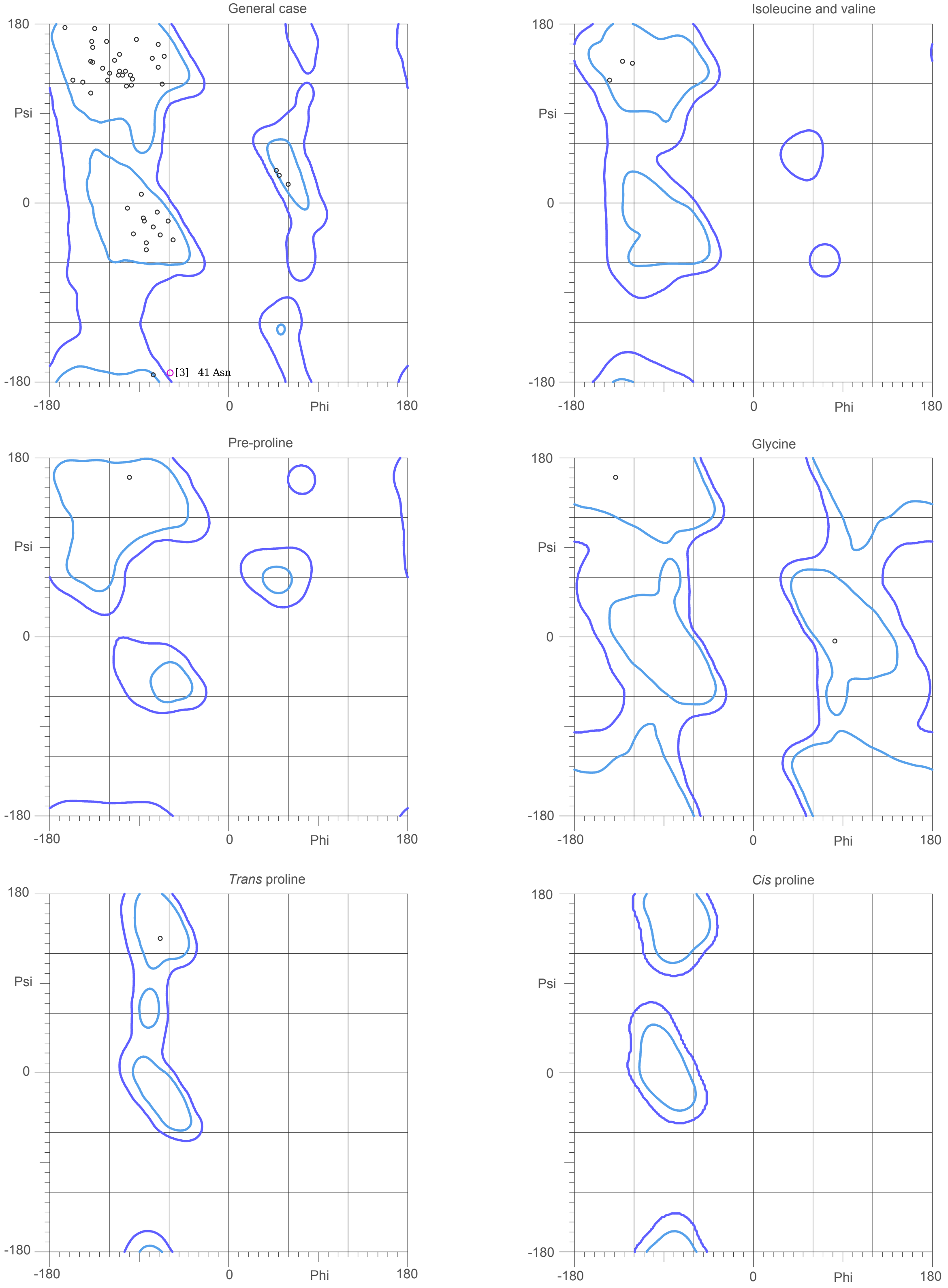

98.1% (52/53) of all residues were in favored (98%) regions.
98.1% (52/53) of all residues were in allowed (>99.8%) regions.

There were 1 outliers (phi, psi):
[3] 41 Asn (-59.6, -171.4)

MolProbity Ramachandran analysis

2mhvH.pdb, model 4

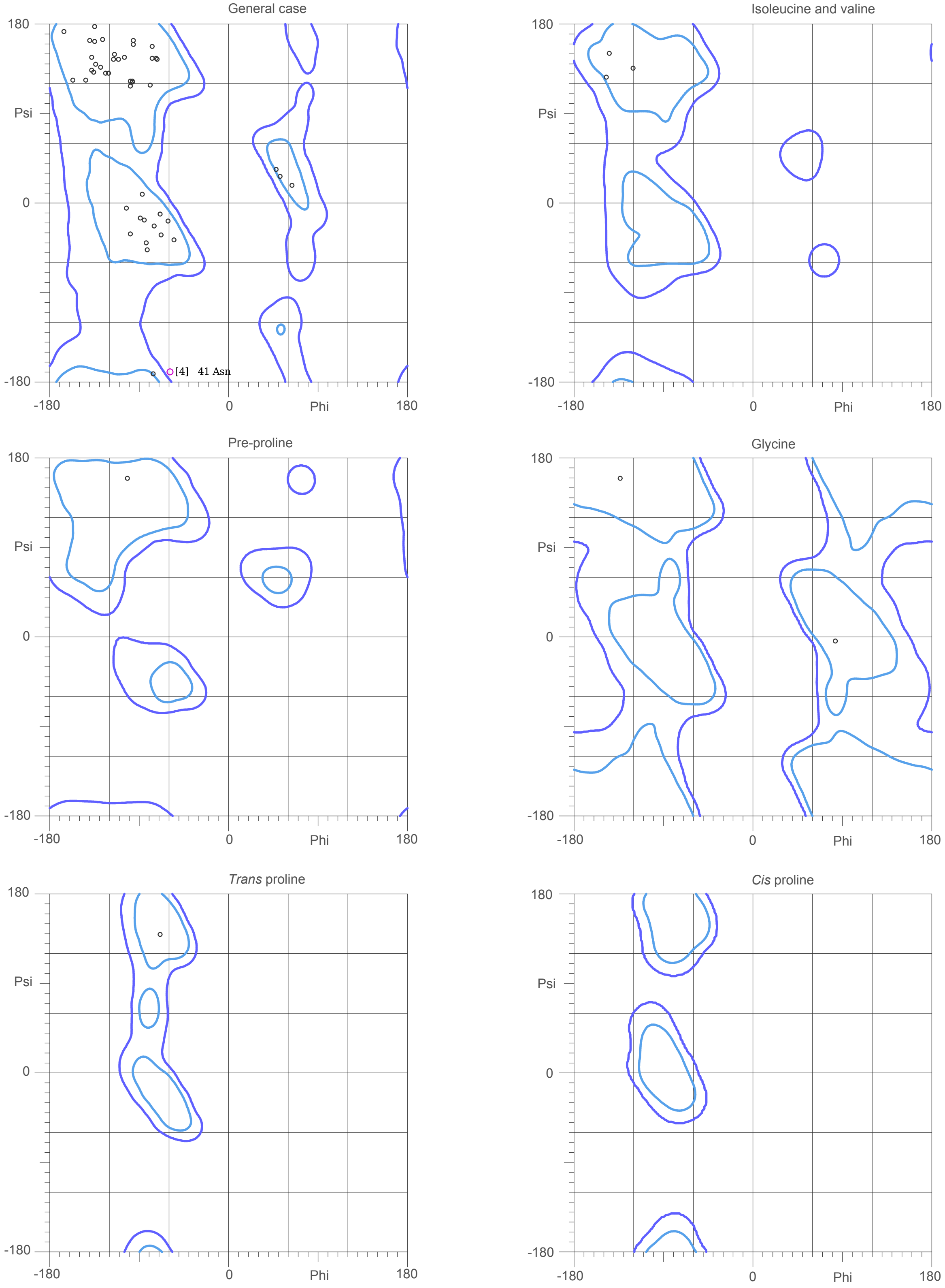

98.1% (52/53) of all residues were in favored (98%) regions.
98.1% (52/53) of all residues were in allowed (>99.8%) regions.

There were 1 outliers (phi, psi):
[4] 41 Asn (-59.1, -171.0)

MolProbity Ramachandran analysis

2mhvH.pdb, model 5

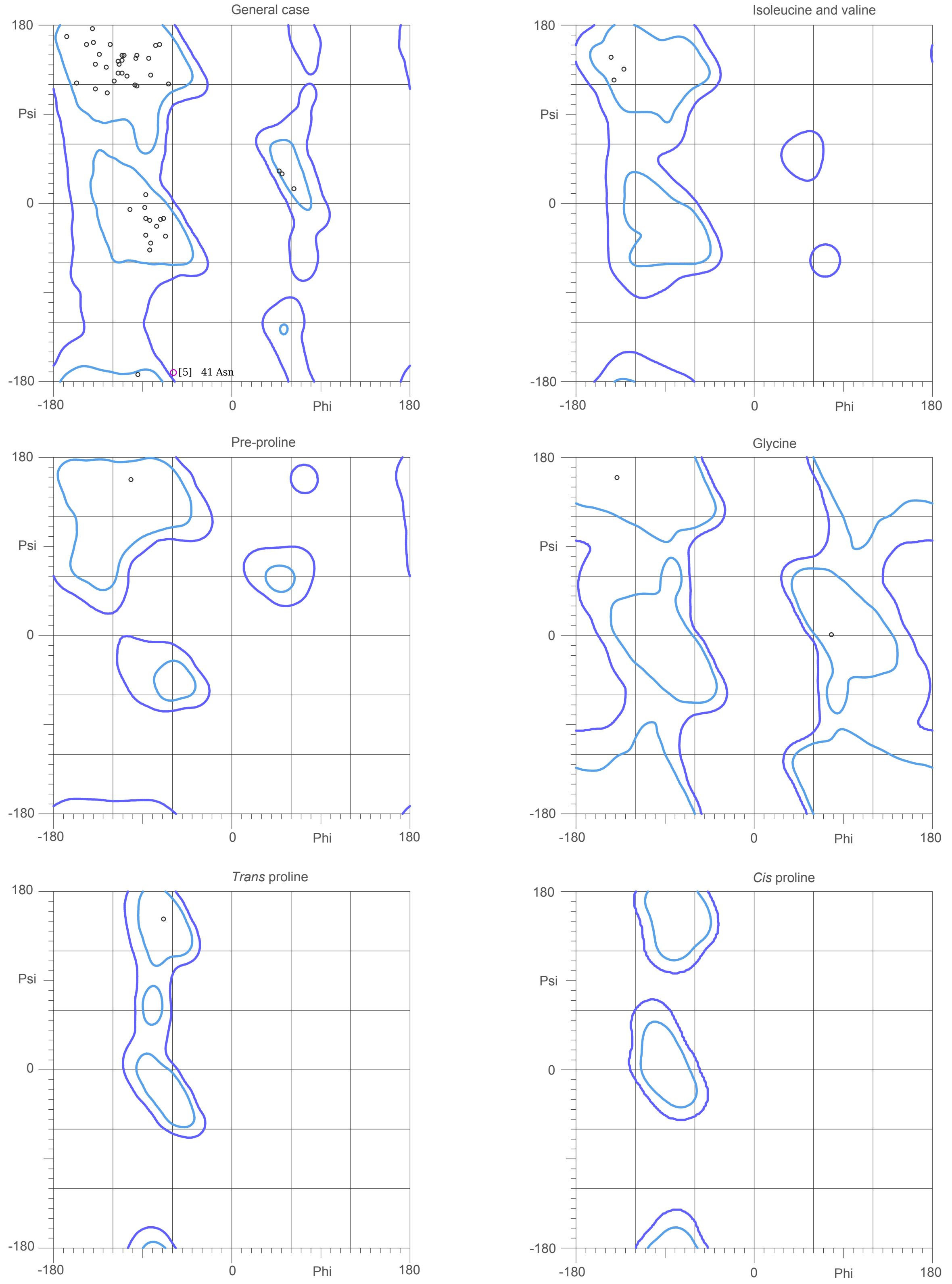

98.1% (52/53) of all residues were in favored (98%) regions.
98.1% (52/53) of all residues were in allowed (>99.8%) regions.

There were 1 outliers (phi, psi):
[5] 41 Asn (-59.7, -171.2)

MolProbity Ramachandran analysis

2mhvH.pdb, model 6

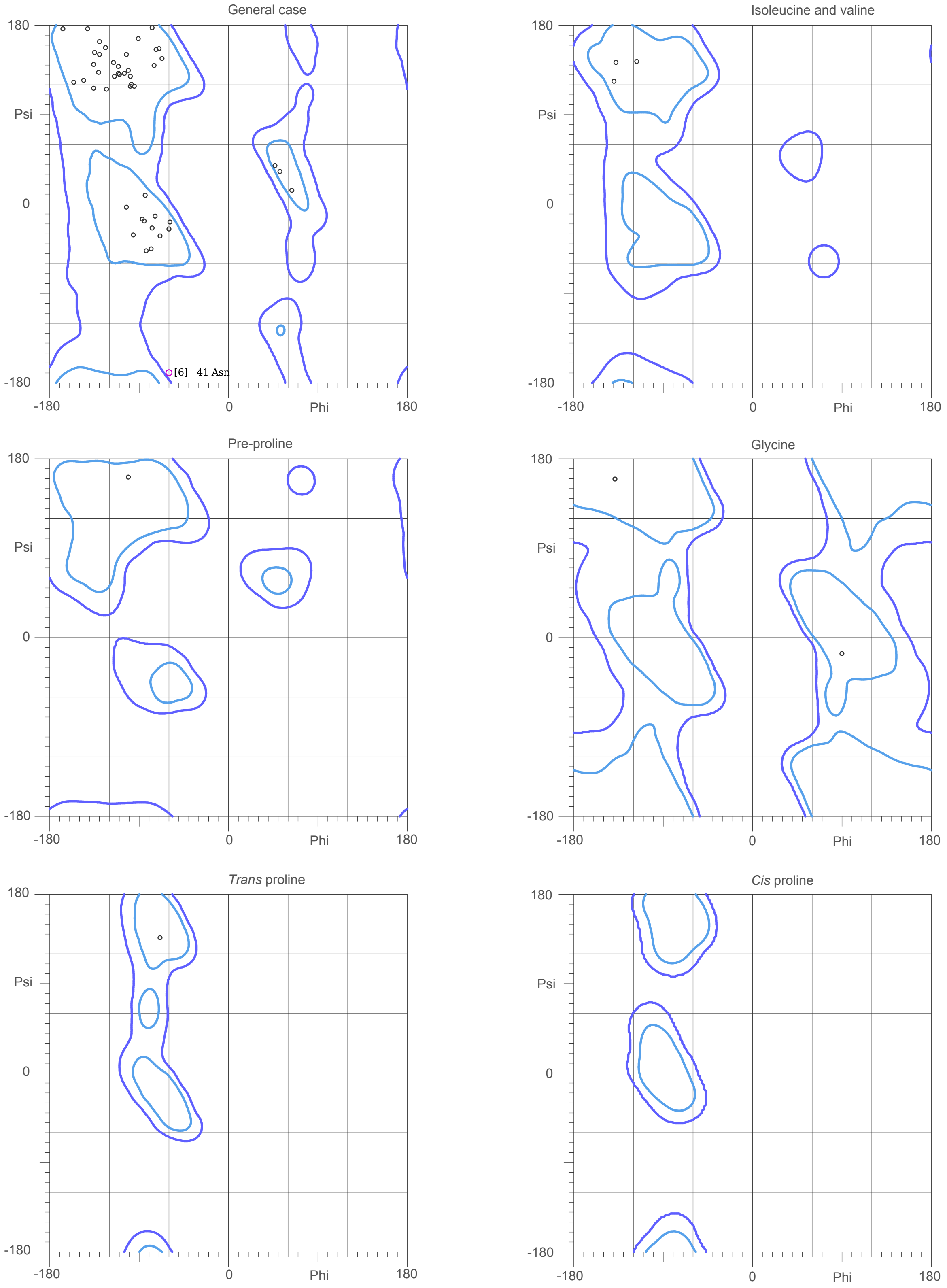

98.1% (52/53) of all residues were in favored (98%) regions.
98.1% (52/53) of all residues were in allowed (>99.8%) regions.

There were 1 outliers (phi, psi):
[6] 41 Asn (-60.3, -170.9)

MolProbity Ramachandran analysis

2mhvH.pdb, model 7

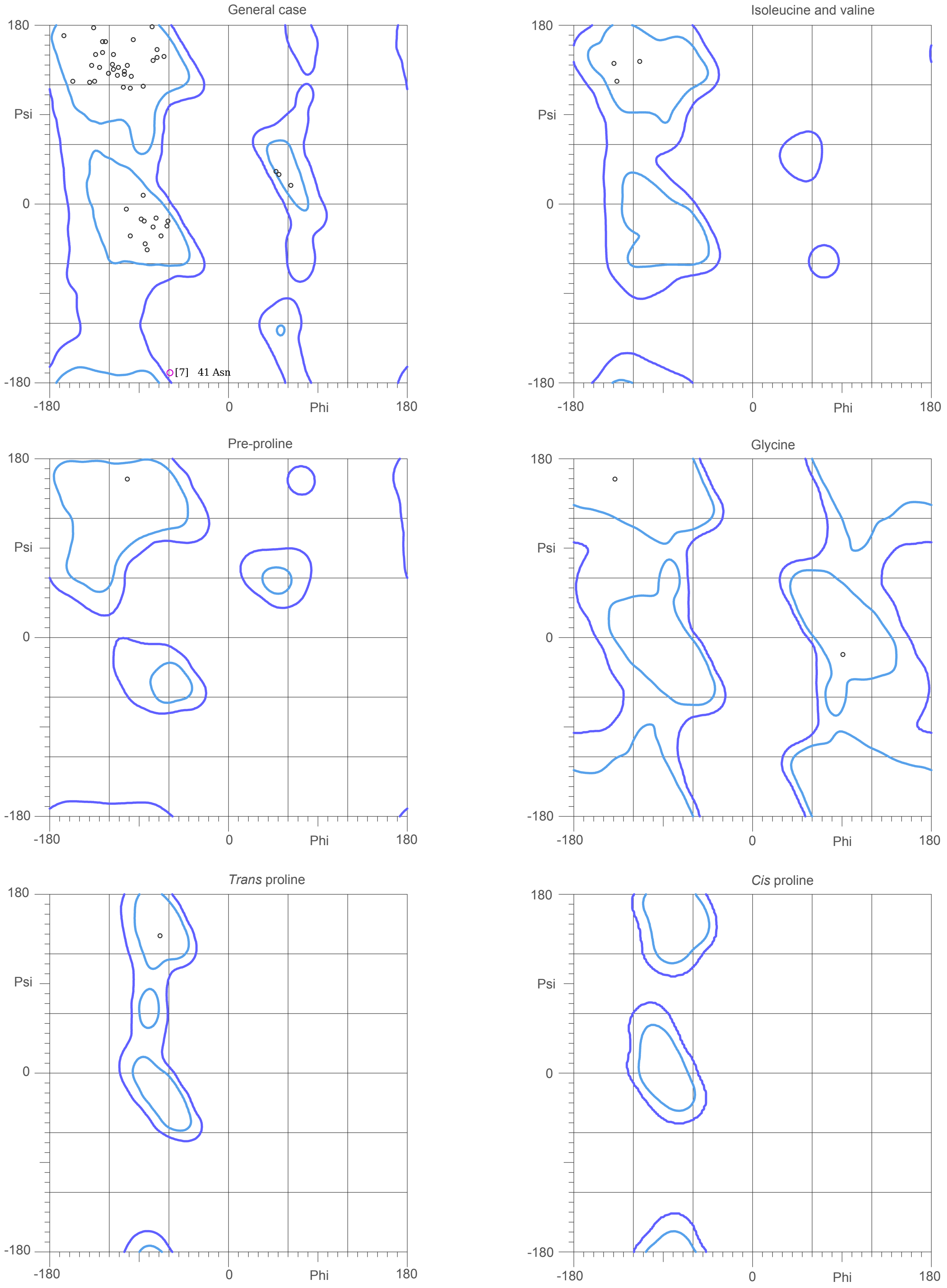

98.1% (52/53) of all residues were in favored (98%) regions.
98.1% (52/53) of all residues were in allowed (>99.8%) regions.

There were 1 outliers (phi, psi):
[7] 41 Asn (-59.2, -170.9)

MolProbity Ramachandran analysis

2mhvH.pdb, model 8

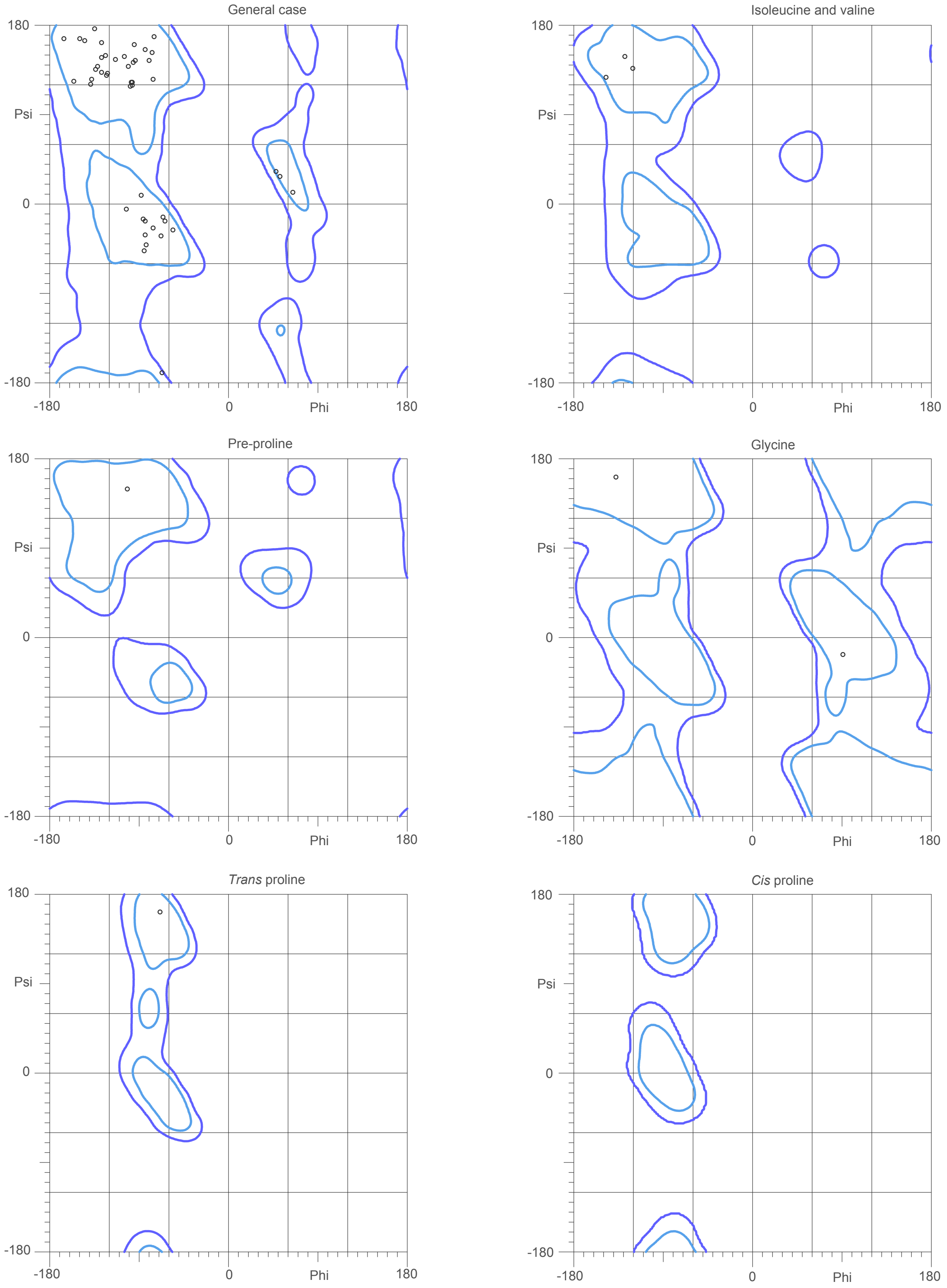

98.1% (52/53) of all residues were in favored (98%) regions.
100.0% (53/53) of all residues were in allowed (>99.8%) regions.

There were no outliers.

MolProbity Ramachandran analysis

2mhvH.pdb, model 9

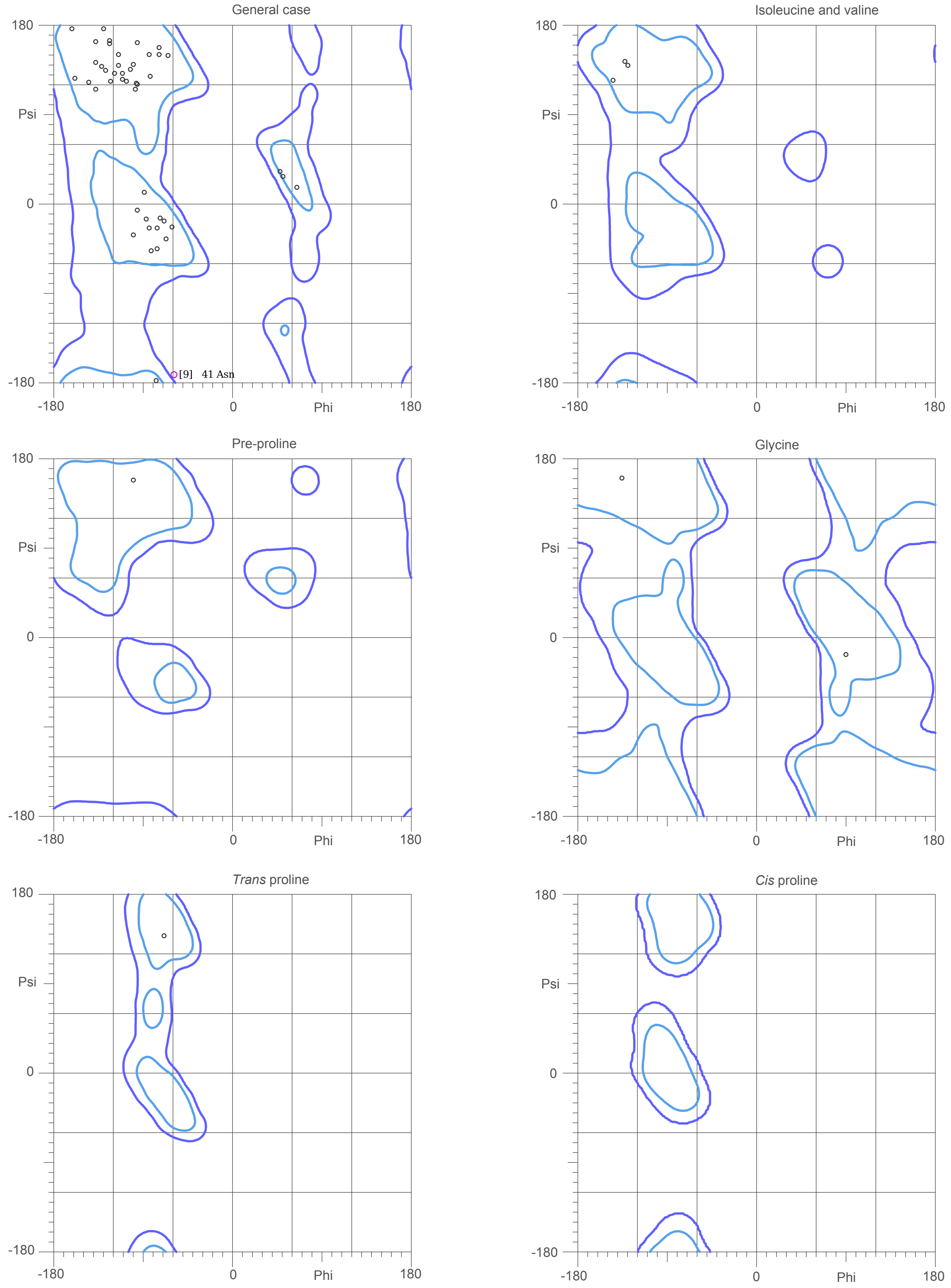

98.1% (52/53) of all residues were in favored (98%) regions.
98.1% (52/53) of all residues were in allowed (>99.8%) regions.

There were 1 outliers (phi, psi):
[9] 41 Asn (-59.1, -172.5)

MolProbity Ramachandran analysis

2mhvH.pdb, model 10

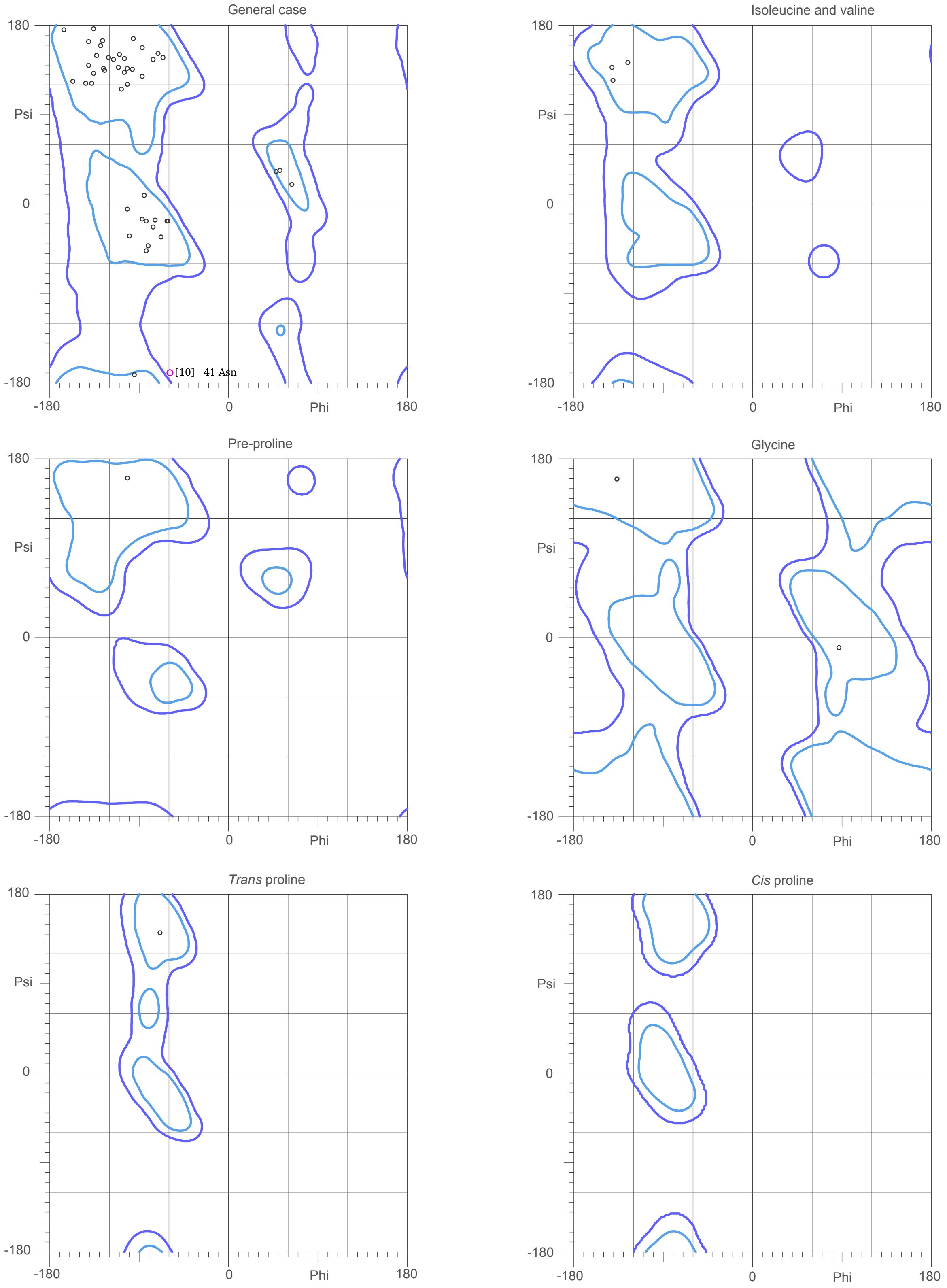

98.1% (52/53) of all residues were in favored (98%) regions.
98.1% (52/53) of all residues were in allowed (>99.8%) regions.

There were 1 outliers (phi, psi):
[10] 41 Asn (-59.3, -170.7)

MolProbity Ramachandran analysis

2mhvH.pdb, model 11

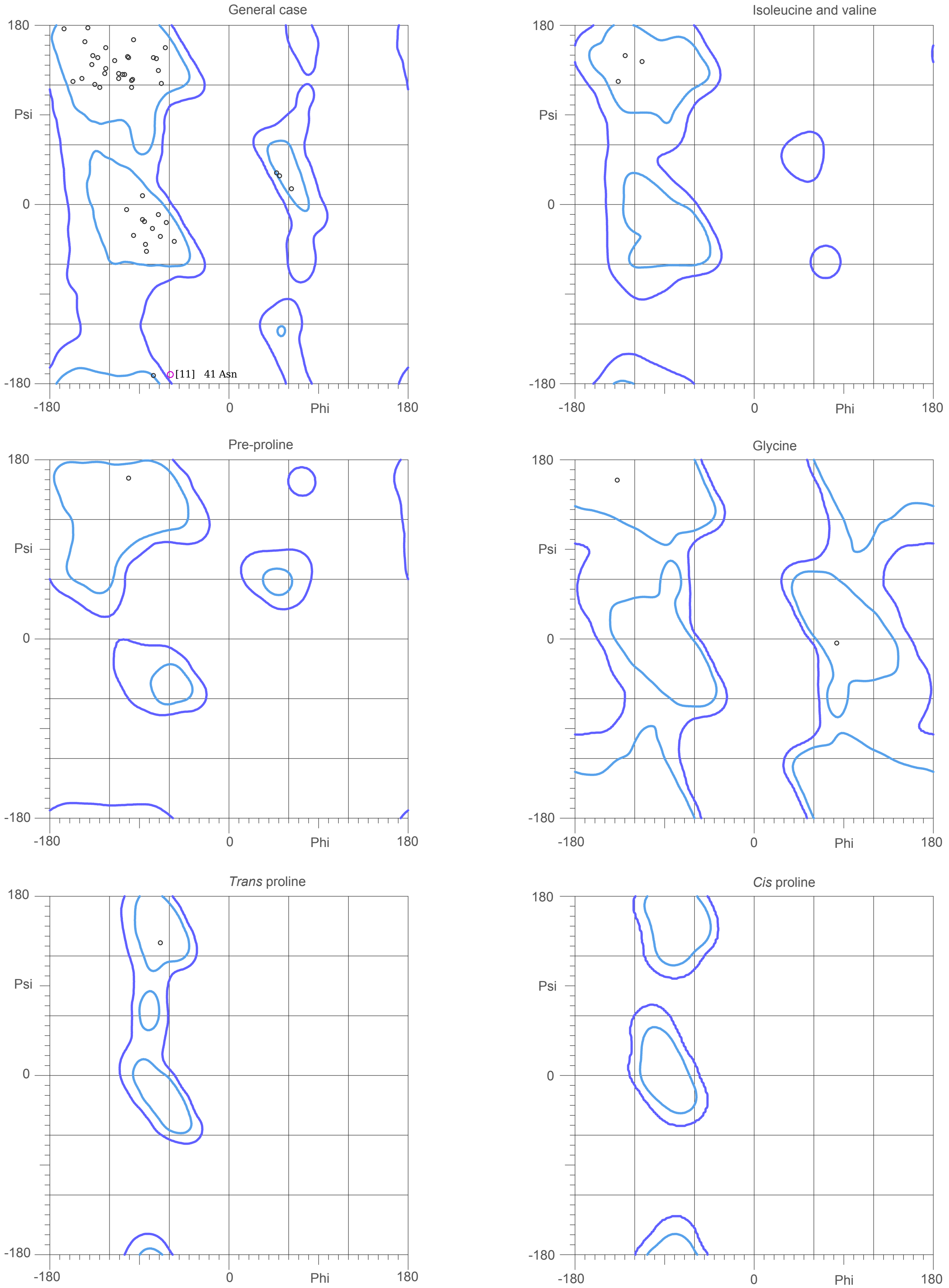

98.1% (52/53) of all residues were in favored (98%) regions.
98.1% (52/53) of all residues were in allowed (>99.8%) regions.

There were 1 outliers (phi, psi):
[11] 41 Asn (-59.5, -171.3)

MolProbity Ramachandran analysis

2mhvH.pdb, model 12

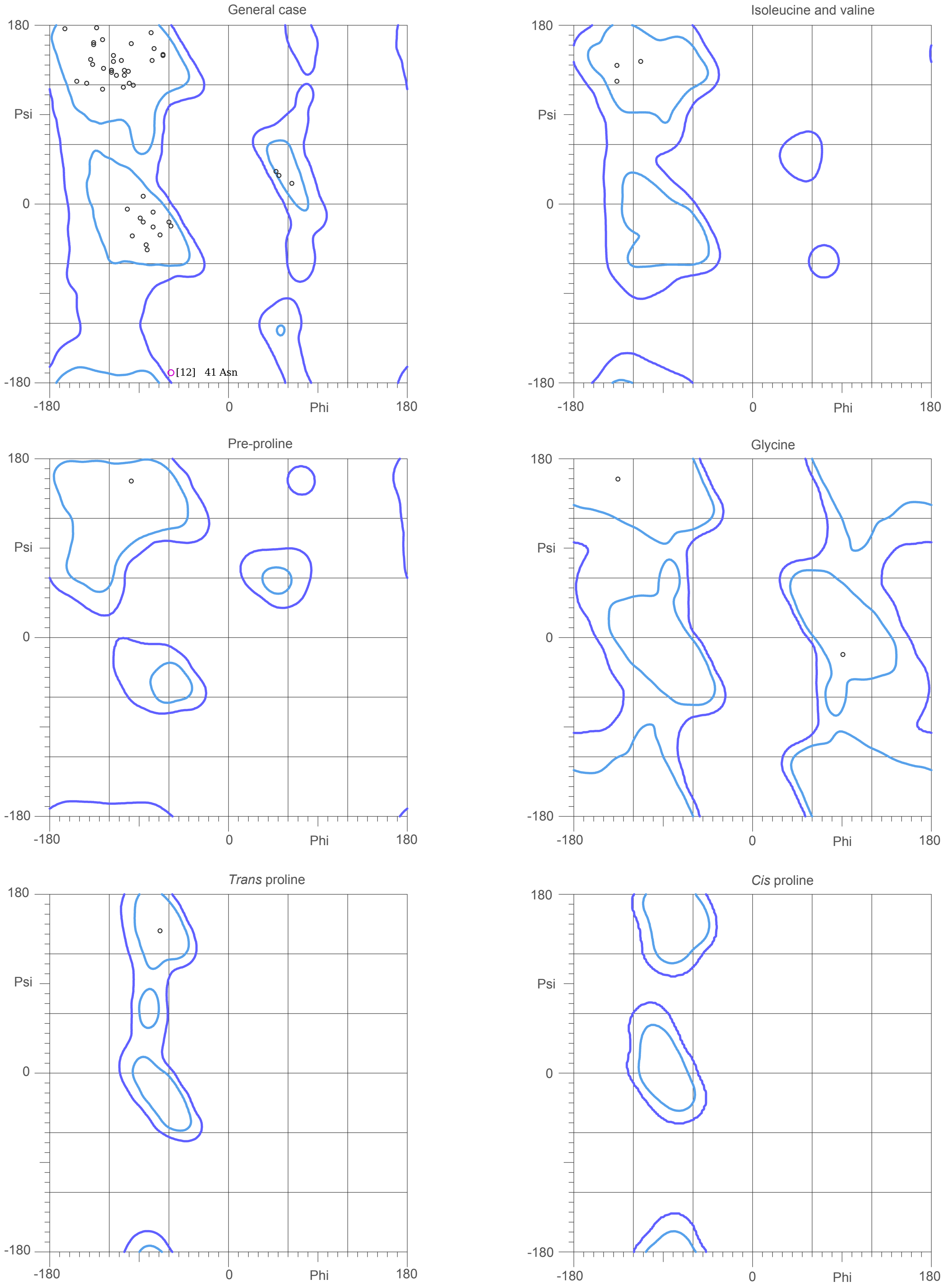

98.1% (52/53) of all residues were in favored (98%) regions.
98.1% (52/53) of all residues were in allowed (>99.8%) regions.

There were 1 outliers (phi, psi):
[12] 41 Asn (-58.9, -170.2)

MolProbity Ramachandran analysis

2mhvH.pdb, model 13

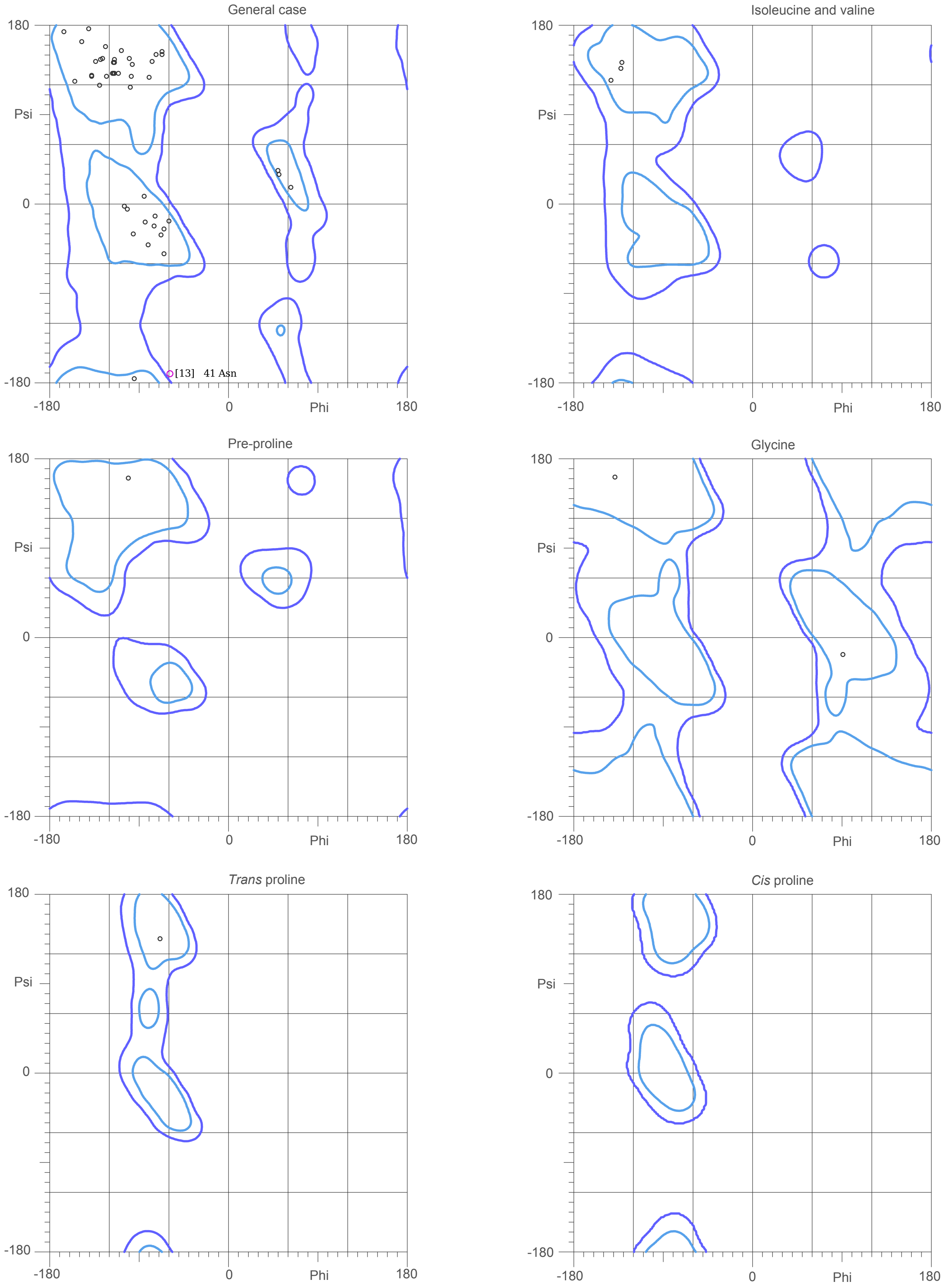

98.1% (52/53) of all residues were in favored (98%) regions.
98.1% (52/53) of all residues were in allowed (>99.8%) regions.

There were 1 outliers (phi, psi):
[13] 41 Asn (-59.2, -171.3)

MolProbity Ramachandran analysis

2mhvH.pdb, model 14

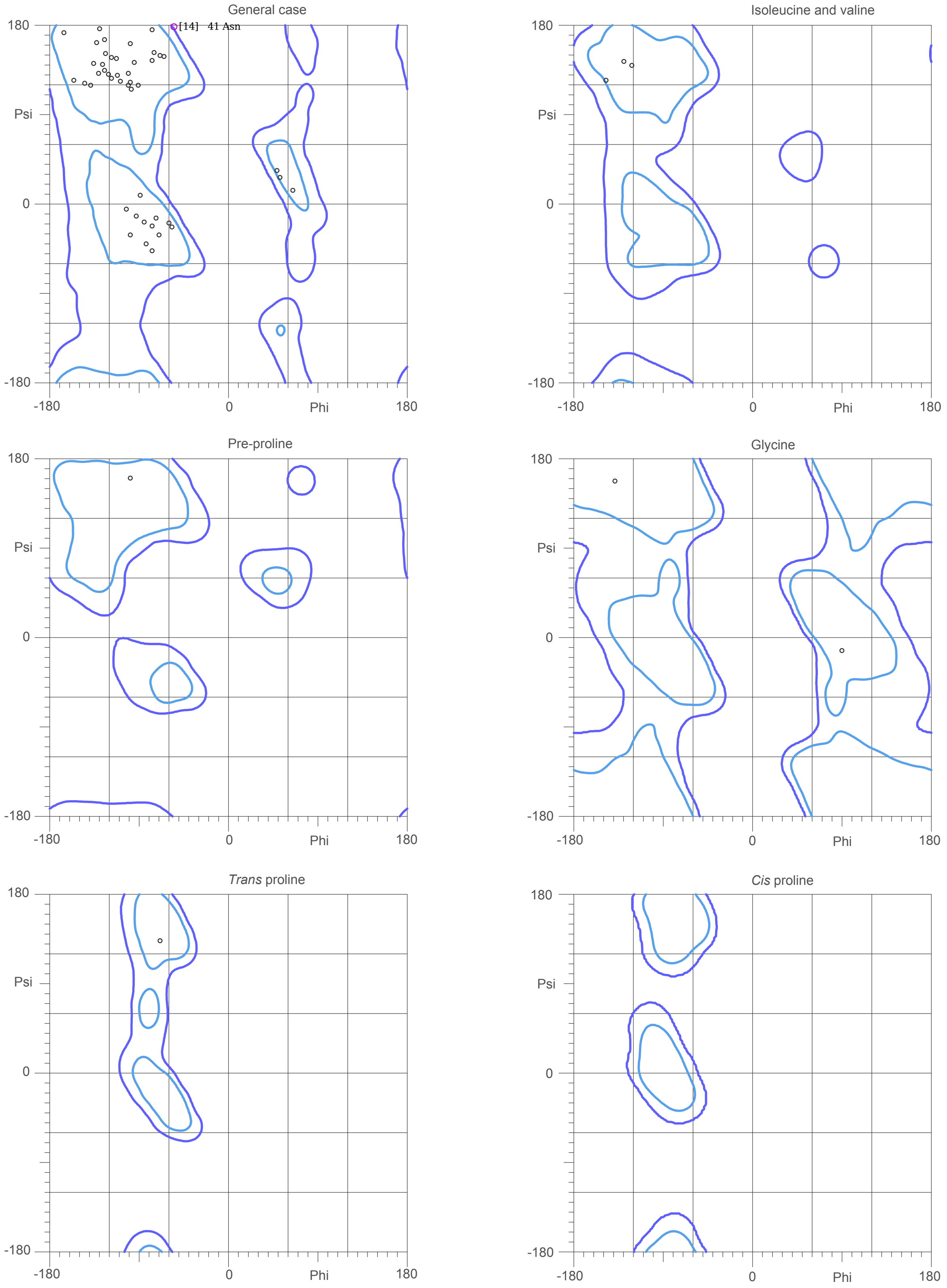

98.1% (52/53) of all residues were in favored (98%) regions.
98.1% (52/53) of all residues were in allowed (>99.8%) regions.

There were 1 outliers (phi, psi):
[14] 41 Asn (-55.5, 179.0)

MolProbity Ramachandran analysis

2mhvH.pdb, model 15

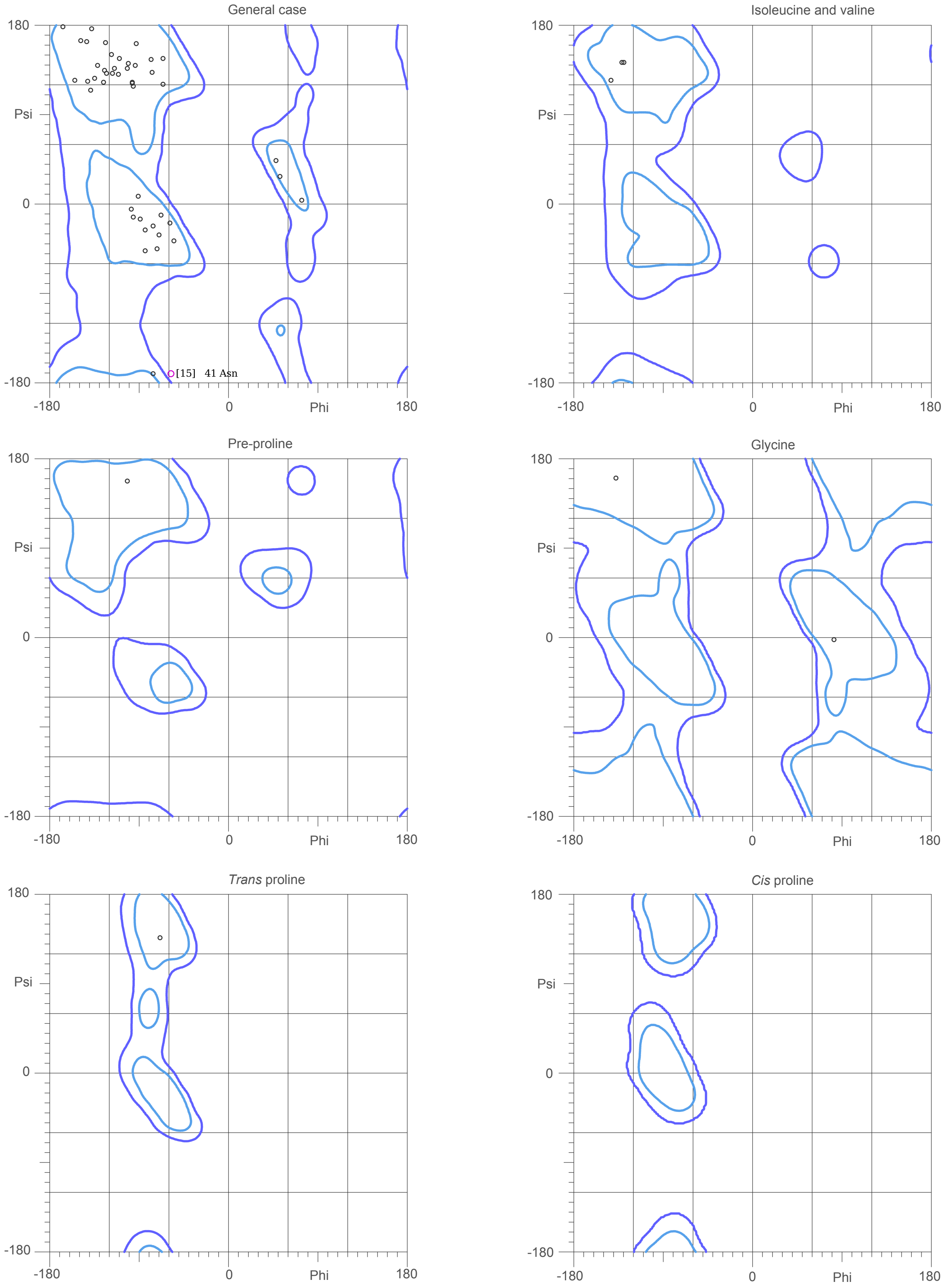

96.2% (51/53) of all residues were in favored (98%) regions.
98.1% (52/53) of all residues were in allowed (>99.8%) regions.

There were 1 outliers (phi, psi):
[15] 41 Asn (-58.2, -171.3)

MolProbity Ramachandran analysis

2mhvH.pdb, model 16

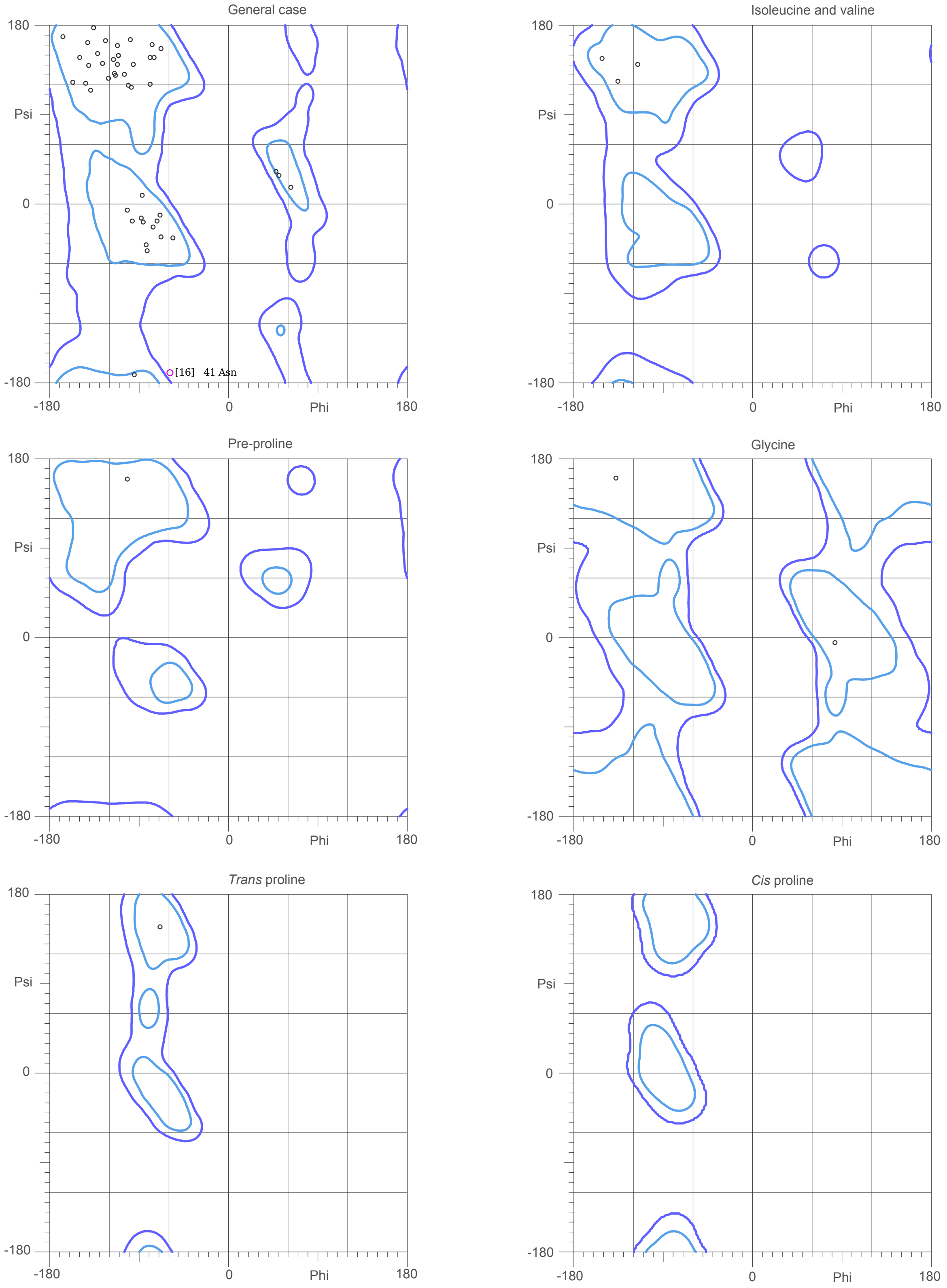

98.1% (52/53) of all residues were in favored (98%) regions.
98.1% (52/53) of all residues were in allowed (>99.8%) regions.

There were 1 outliers (phi, psi):
[16] 41 Asn (-59.4, -170.7)

MolProbity Ramachandran analysis

2mhvH.pdb, model 17

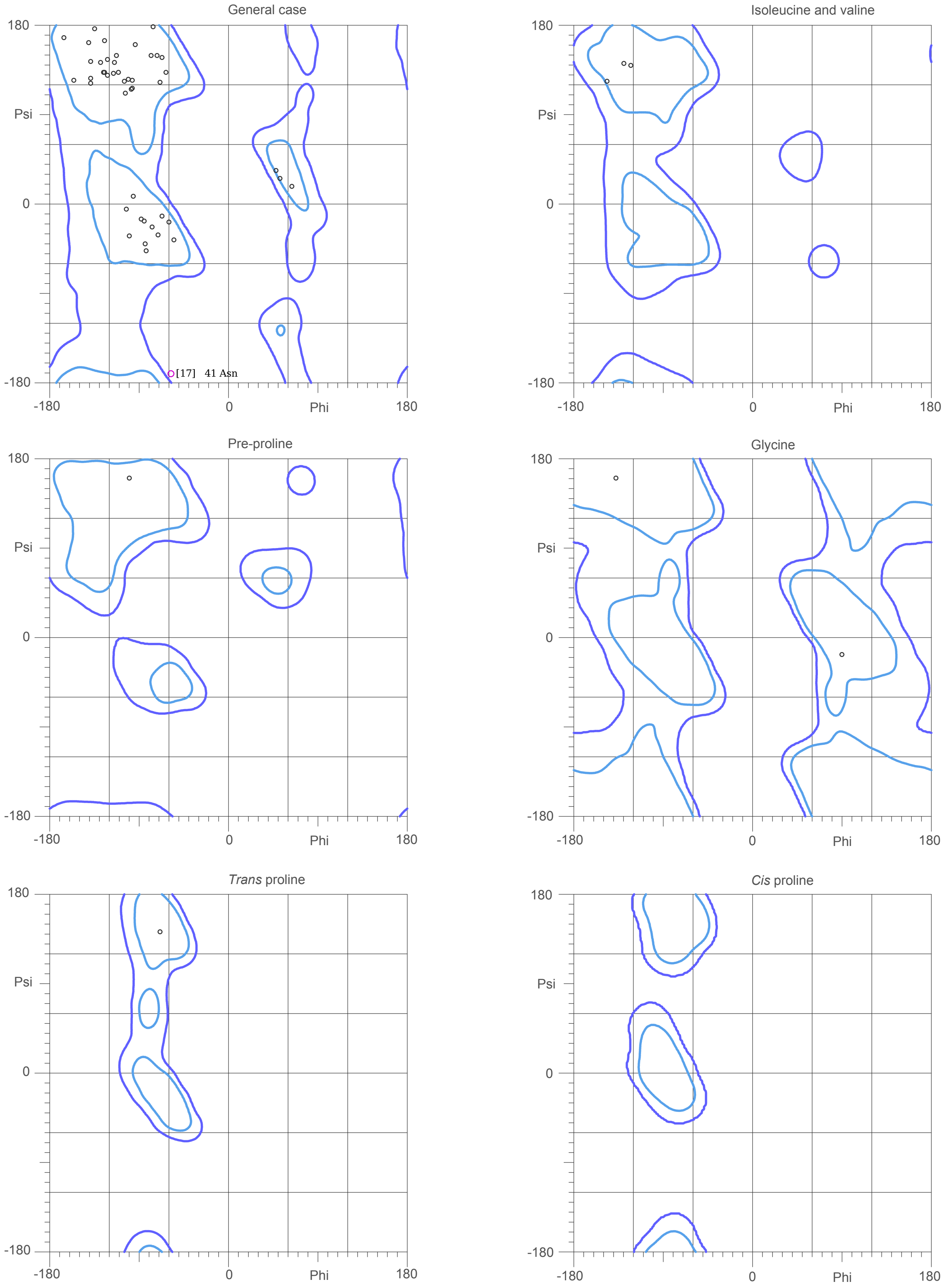

98.1% (52/53) of all residues were in favored (98%) regions.
98.1% (52/53) of all residues were in allowed (>99.8%) regions.

There were 1 outliers (phi, psi):
[17] 41 Asn (-58.6, -171.9)

MolProbity Ramachandran analysis

2mhvH.pdb, model 18

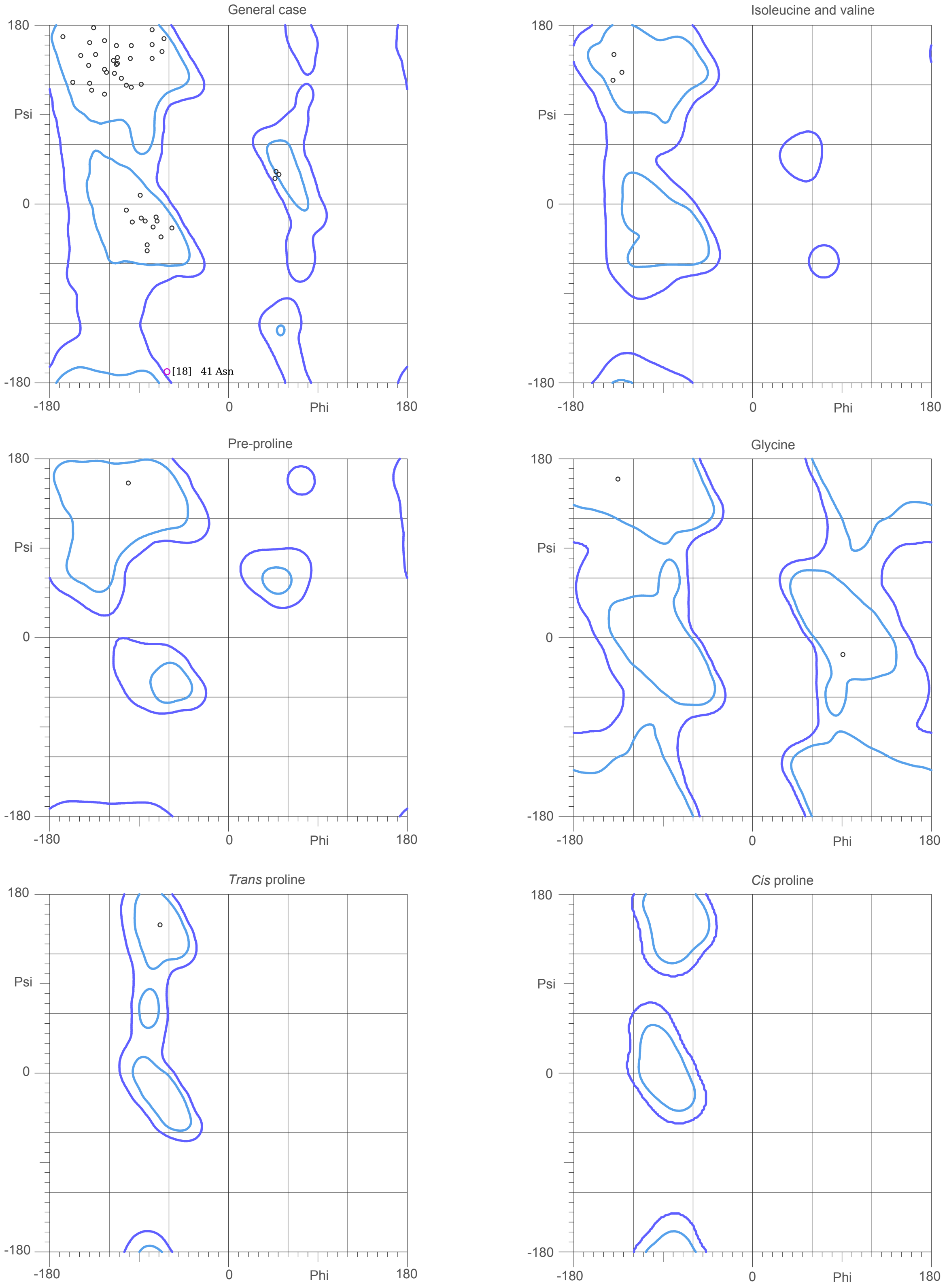

96.2% (51/53) of all residues were in favored (98%) regions.
98.1% (52/53) of all residues were in allowed (>99.8%) regions.

There were 1 outliers (phi, psi):
[18] 41 Asn (-62.4, -169.5)

MolProbity Ramachandran analysis

2mhvH.pdb, model 19

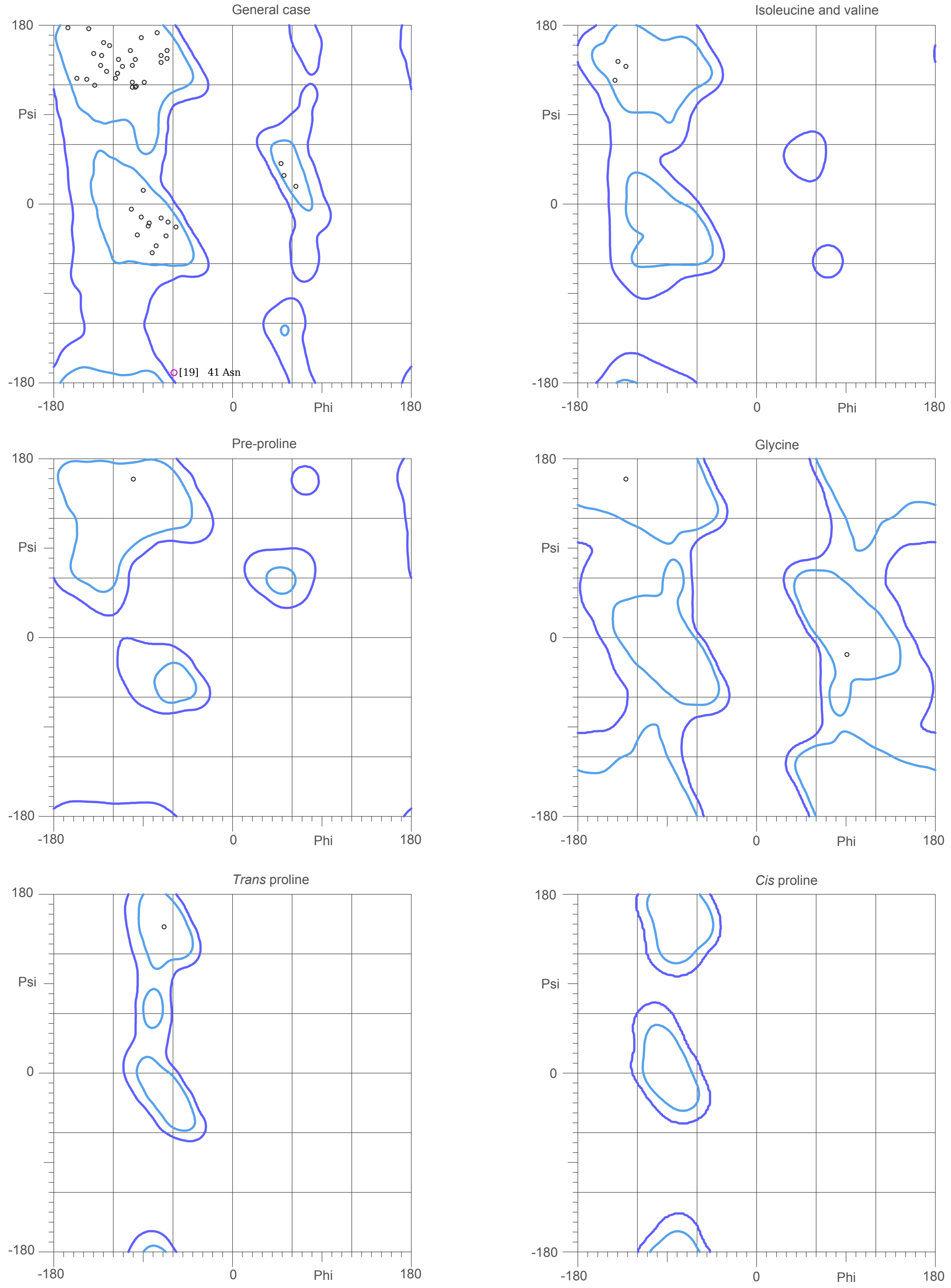

98.1% (52/53) of all residues were in favored (98%) regions.
98.1% (52/53) of all residues were in allowed (>99.8%) regions.

There were 1 outliers (phi, psi):
[19] 41 Asn (-59.6, -170.3)

MolProbity Ramachandran analysis

2mhvH.pdb, model 20

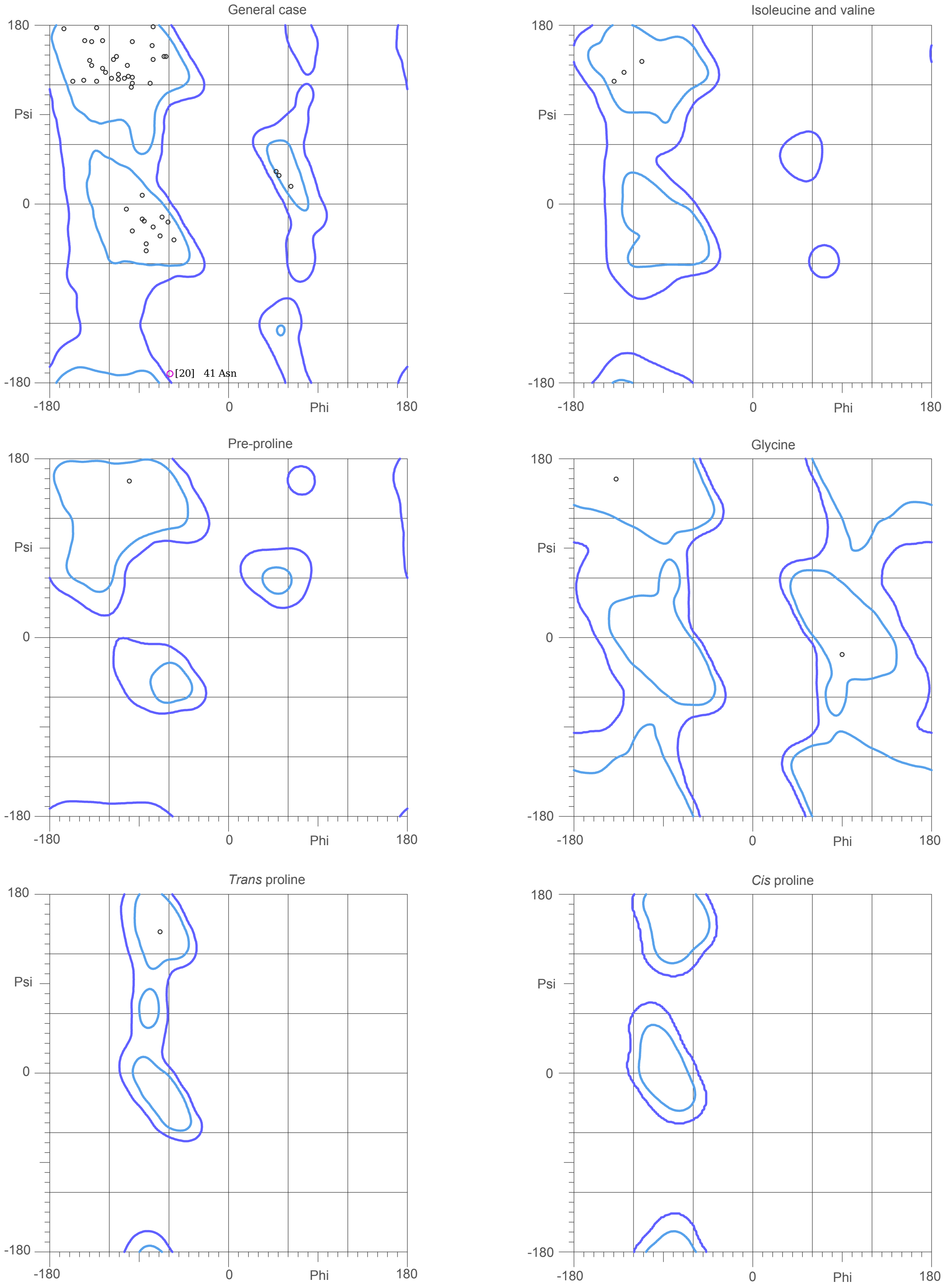

98.1% (52/53) of all residues were in favored (98%) regions.
98.1% (52/53) of all residues were in allowed (>99.8%) regions.

There were 1 outliers (phi, psi):
[20] 41 Asn (-59.1, -171.4)
