# Supplementary material for: Small Disulfide Proteins with Antifungal Impact: NMR Experimental Structures as Compared to Models of Alphafold Versions
Source: Int J Mol Sci. 2025 Jan 31;26(3):1247. doi: 10.3390/ijms26031247 (PMC11818080; doi:10.3390/ijms26031247)
Supplement: Supplementary file 1 [file ijms-26-01247-s001.zip › Figure S3b. AF2-PAF.pdf]

MolProbity Ramachandran analysis

2KCN-1FH.pdb, model 1

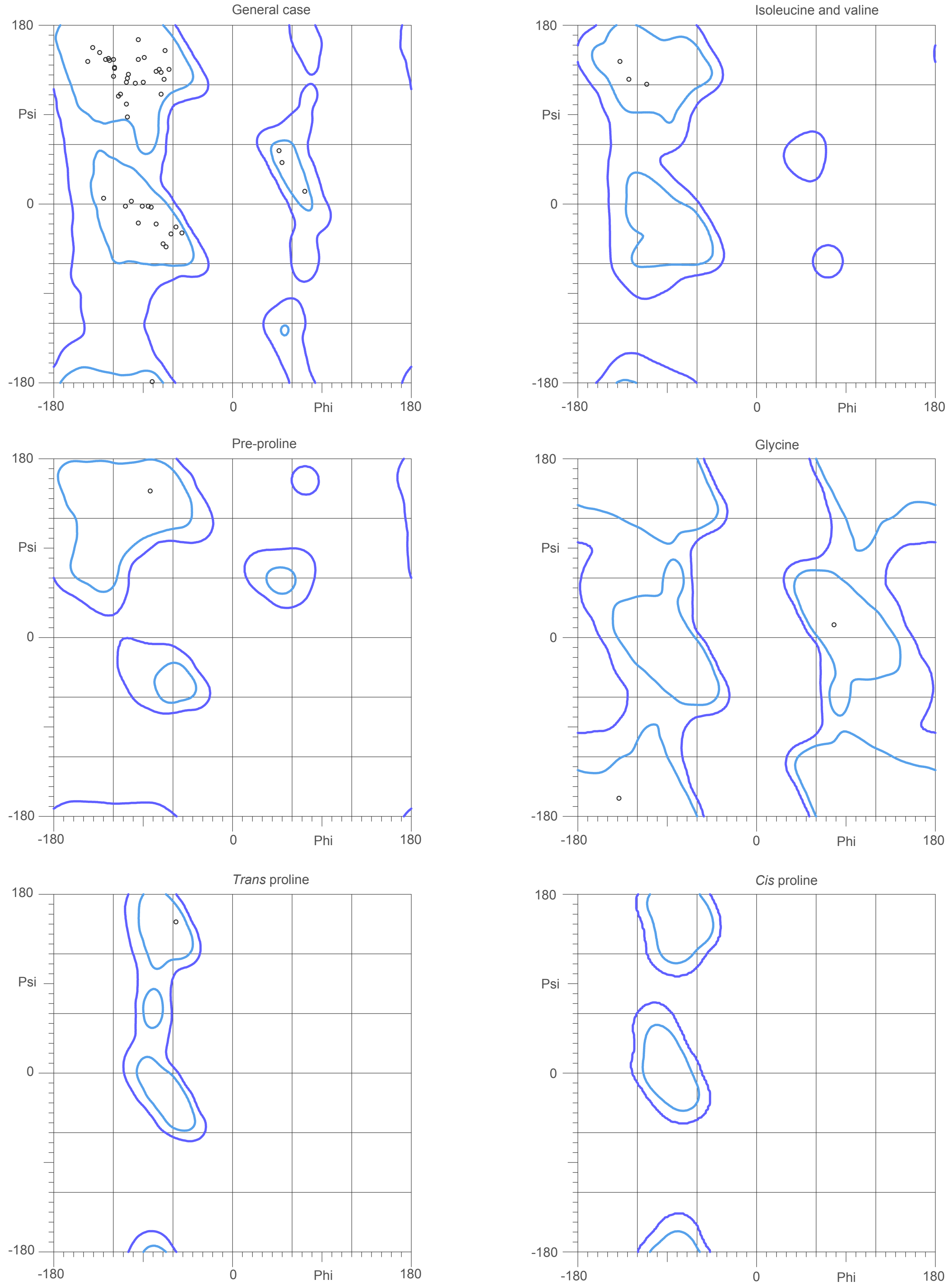

100.0% (53/53) of all residues were in favored (98%) regions.
100.0% (53/53) of all residues were in allowed (>99.8%) regions.

There were no outliers.
