# Supplementary material for: Small Disulfide Proteins with Antifungal Impact: NMR Experimental Structures as Compared to Models of Alphafold Versions
Source: Int J Mol Sci. 2025 Jan 31;26(3):1247. doi: 10.3390/ijms26031247 (PMC11818080; doi:10.3390/ijms26031247)
Supplement: Supplementary file 1 [file ijms-26-01247-s001.zip › Figure S7a. NMR-NFAP-5oqs.pdf]

MolProbity Ramachandran analysis

5oqsH.pdb, all models

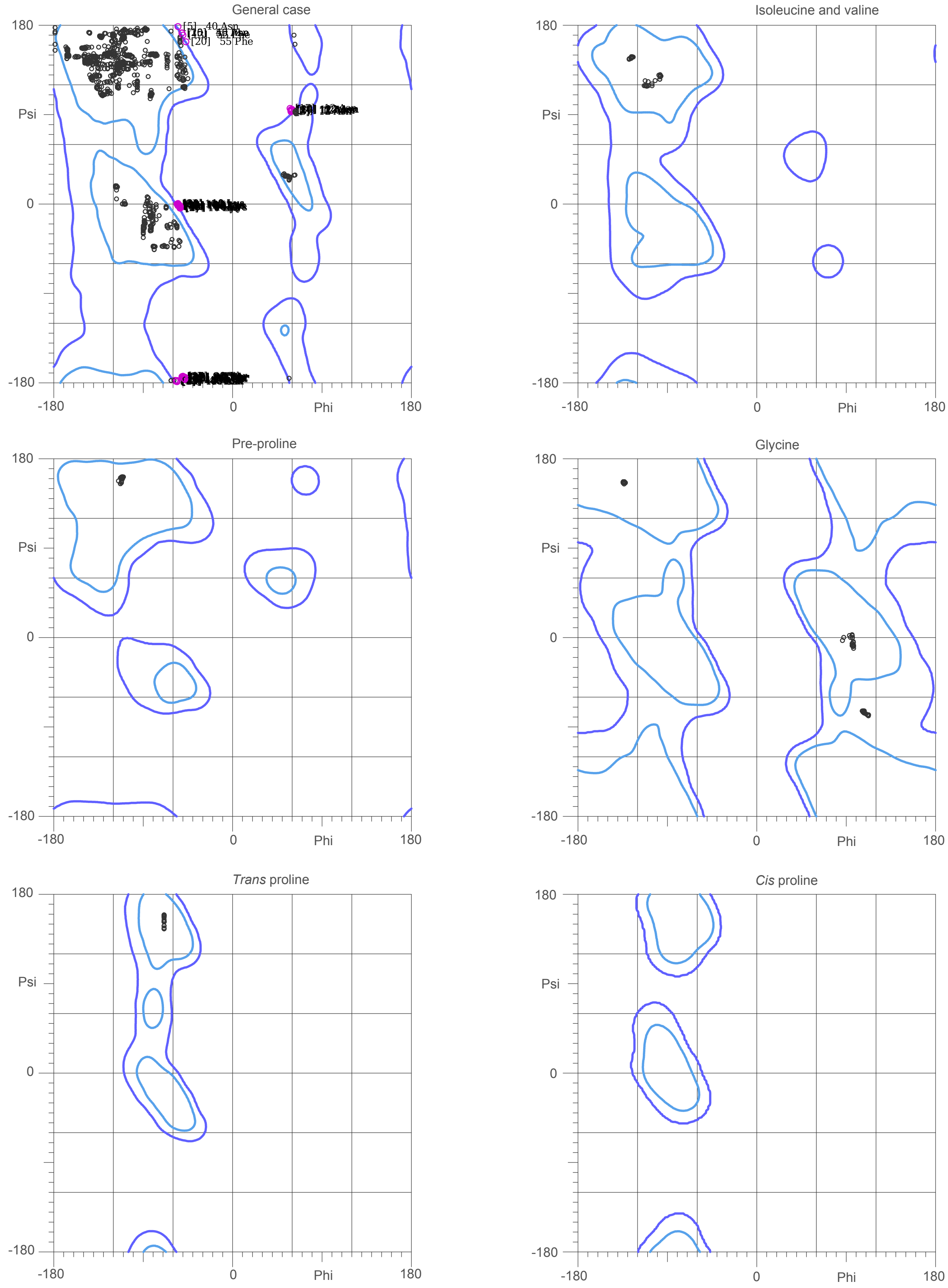

89.2% (981/1100) of all residues were in favored (98%) regions.
93.6% (1030/1100) of all residues were in allowed (>99.8%) regions.
This list is truncated; use the MolProbity multi-chart.html for complete list.
There were 70 outliers (phi, psi):

[1] 9 Thr (-49.0, -175.4)
[1] 10 Lys (-53.8, -3.0)
[1] 12 Asn (59.8, 95.2)
[2] 9 Thr (-51.2, -174.6)
[2] 10 Lys (-53.4, -4.2)
[2] 12 Asn (59.5, 94.7)
[2] 40 Asn (-56.2, -179.4)
[3] 9 Thr (-50.5, -175.1)
[3] 10 Lys (-53.9, -3.4)
[3] 12 Asn (60.7, 95.6)
[3] 40 Asn (-56.3, -179.5)
[4] 9 Thr (-49.7, -176.6)
[4] 10 Lys (-55.7, -1.5)
[4] 40 Asn (-56.4, -179.2)
[5] 9 Thr (-48.7, -177.2)
[5] 10 Lys (-56.1, -0.4)
[5] 40 Asn (-55.5, 179.7)
[6] 9 Thr (-49.8, -176.7)
[6] 10 Lys (-55.6, -1.7)
[6] 40 Asn (-56.7, -178.8)
[7] 9 Thr (-48.9, -176.0)
[7] 10 Lys (-54.4, -2.2)
[7] 12 Asn (62.2, 94.9)
[8] 9 Thr (-50.3, -174.1)
[8] 10 Lys (-53.4, -2.9)
[8] 12 Asn (59.7, 93.6)
[8] 40 Asn (-56.3, -179.7)
[9] 9 Thr (-49.9, -175.7)
[9] 10 Lys (-55.0, -1.9)
[9] 40 Asn (-56.3, -179.3)
[10] 9 Thr (-49.0, -175.3)
[10] 10 Lys (-53.7, -3.0)
[10] 12 Asn (59.6, 95.2)
[10] 40 Asn (-58.2, -178.5)
[11] 9 Thr (-49.3, -176.4)
[11] 10 Lys (-55.6, -0.8)
[11] 40 Asn (-56.2, -179.6)
[12] 9 Thr (-49.7, -175.0)
[12] 10 Lys (-53.5, -3.4)
[12] 12 Asn (60.1, 95.8)
[12] 40 Asn (-56.2, -179.5)
[13] 9 Thr (-48.6, -176.3)
[13] 10 Lys (-55.3, -1.0)
[13] 40 Asn (-57.6, -179.3)
[13] 55 Phe (-50.4, 170.7)
[14] 9 Thr (-48.6, -175.7)
[14] 10 Lys (-53.9, -2.7)
[14] 12 Asn (60.2, 95.1)
[15] 9 Thr (-48.7, -175.7)
[15] 10 Lys (-54.1, -2.7)
[15] 12 Asn (60.8, 95.1)
[15] 40 Asn (-56.7, -179.6)
[15] 55 Phe (-51.5, 172.1)
[16] 9 Thr (-49.8, -176.5)
[16] 10 Lys (-55.3, -1.6)
[16] 40 Asn (-56.3, -179.3)
[17] 9 Thr (-50.0, -174.4)
[17] 10 Lys (-52.5, -4.4)
[17] 12 Asn (58.2, 96.8)
[17] 40 Asn (-56.4, -179.2)
[18] 9 Thr (-49.9, -176.2)
[18] 10 Lys (-55.2, -2.1)
[18] 40 Asn (-56.3, -179.2)
[19] 9 Thr (-50.6, -175.8)

Lovell, Davis, et al. Proteins 50:437 (2003)

MolProbity Ramachandran analysis

5oqsH.pdb, model 1

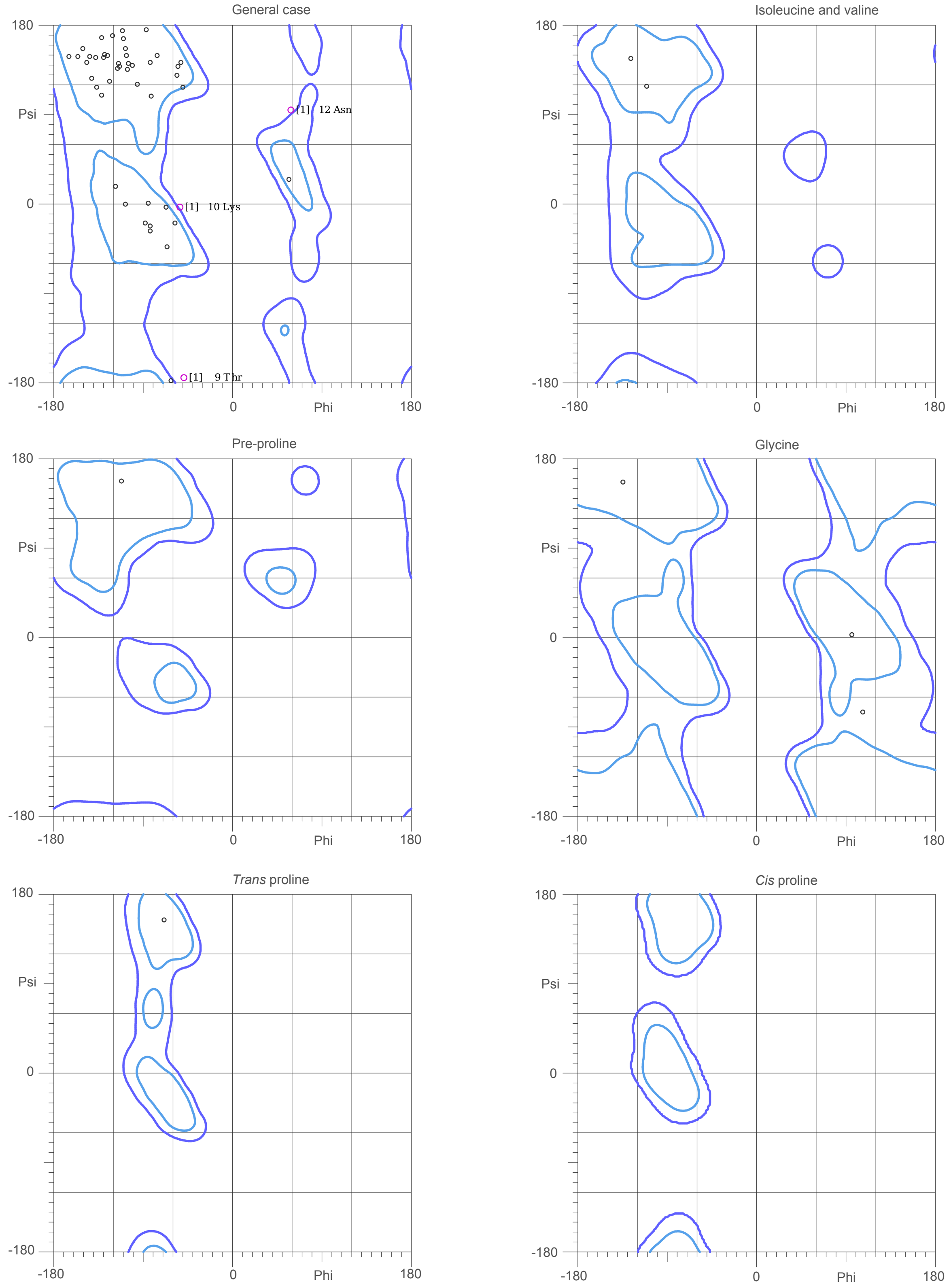

90.9% (50/55) of all residues were in favored (98%) regions.
94.5% (52/55) of all residues were in allowed (>99.8%) regions.

There were 3 outliers (phi, psi):
[1] 9 Thr (-49.0, -175.4)
[1] 10 Lys (-53.8, -3.0)
[1] 12 Asn (59.8, 95.2)

MolProbity Ramachandran analysis

5oqsH.pdb, model 2

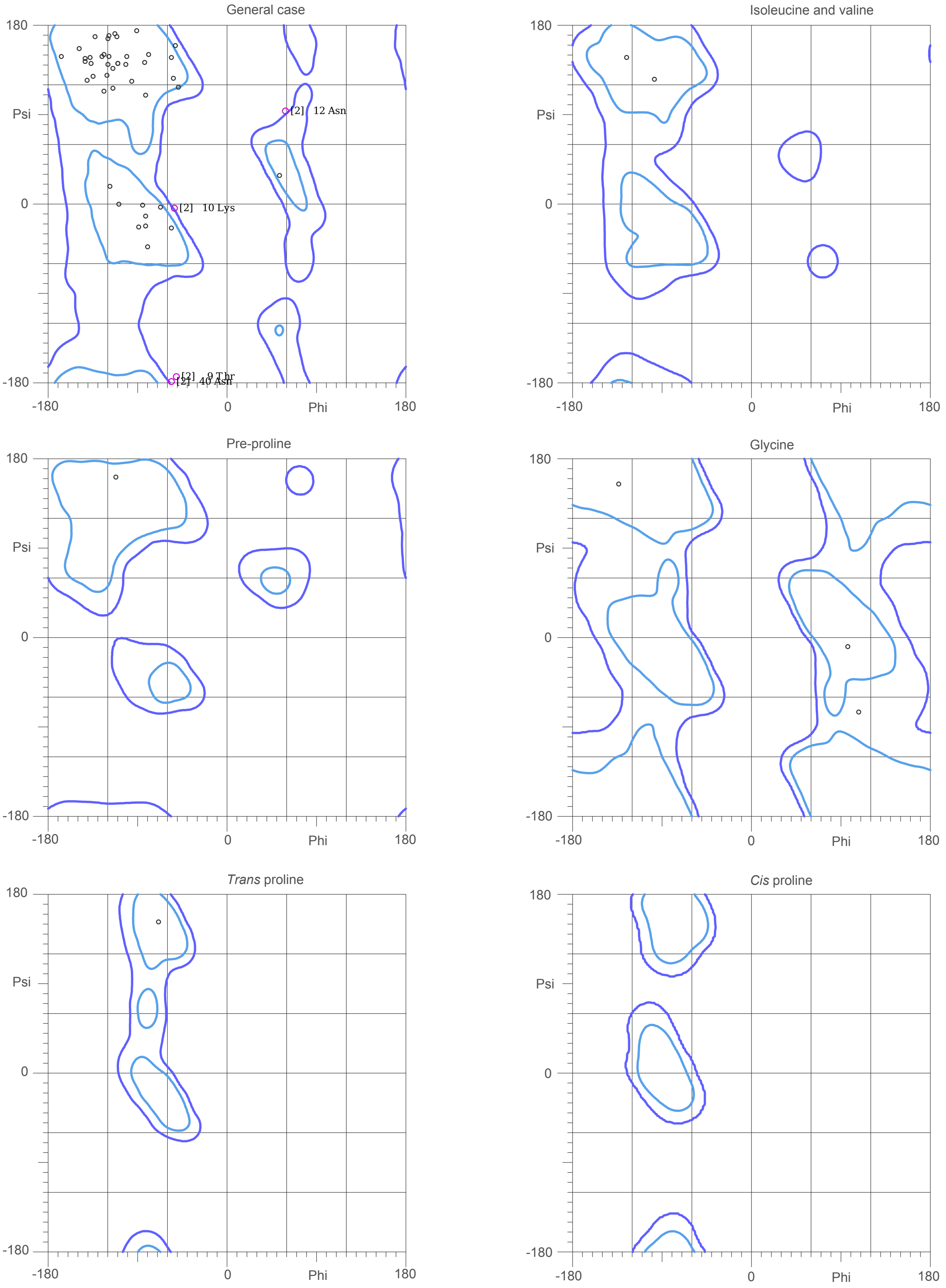

89.1% (49/55) of all residues were in favored (98%) regions.
92.7% (51/55) of all residues were in allowed (>99.8%) regions.

There were 4 outliers (phi, psi):
[2] 9 Thr (-51.2, -174.6)
[2] 10 Lys (-53.4, -4.2)
[2] 12 Asn (59.5, 94.7)
[2] 40 Asn (-56.2, -179.4)

http://kinemage.biochem.duke.edu

Lovell, Davis, et al. Proteins 50:437 (2003)

MolProbity Ramachandran analysis

5oqsH.pdb, model 3

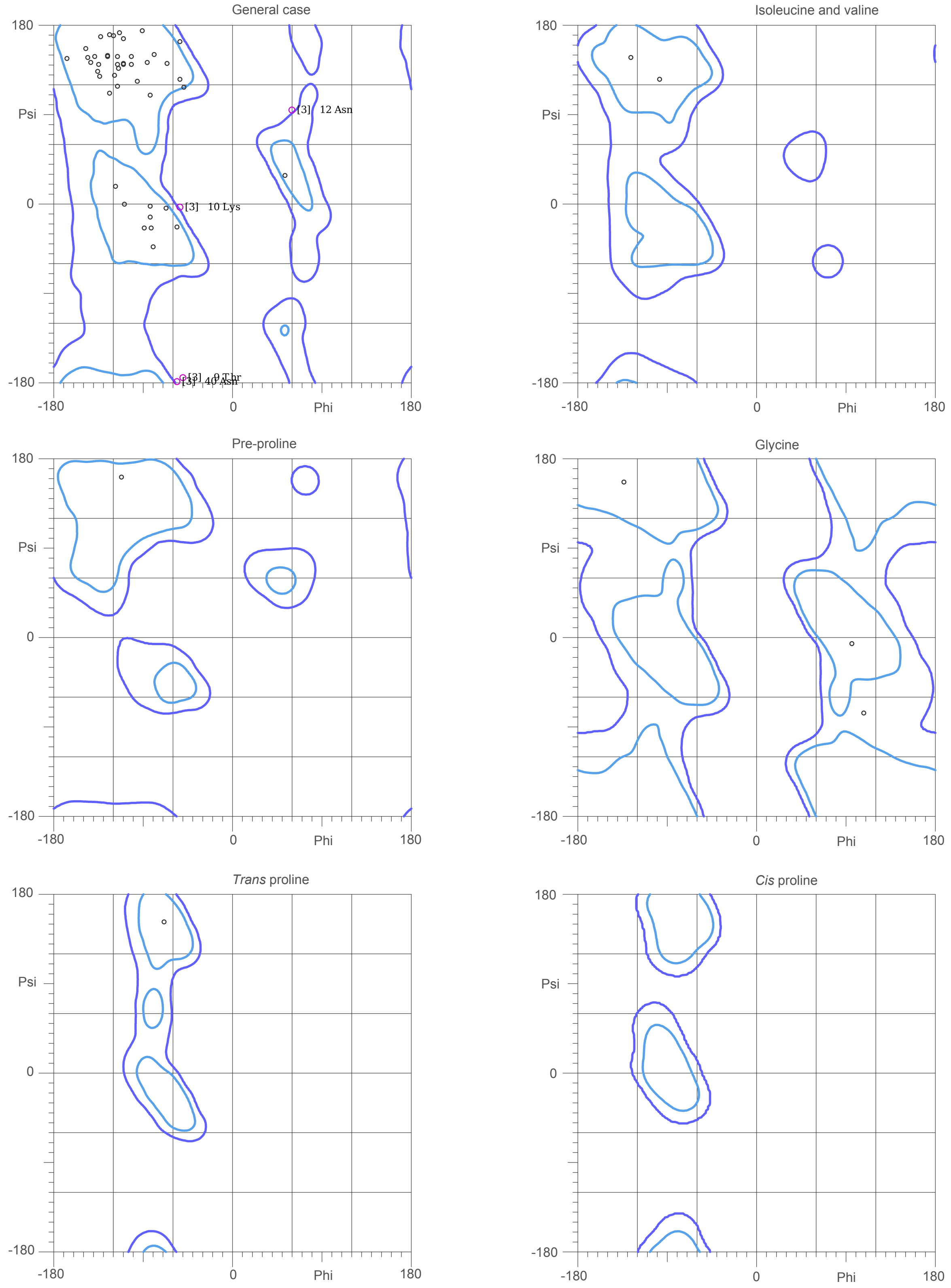

89.1% (49/55) of all residues were in favored (98%) regions.
92.7% (51/55) of all residues were in allowed (>99.8%) regions.

There were 4 outliers (phi, psi):
[3] 9 Thr (-50.5, -175.1)
[3] 10 Lys (-53.9, -3.4)
[3] 12 Asn (60.7, 95.6)
[3] 40 Asn (-56.3, -179.5)

MolProbity Ramachandran analysis

5oqsH.pdb, model 4

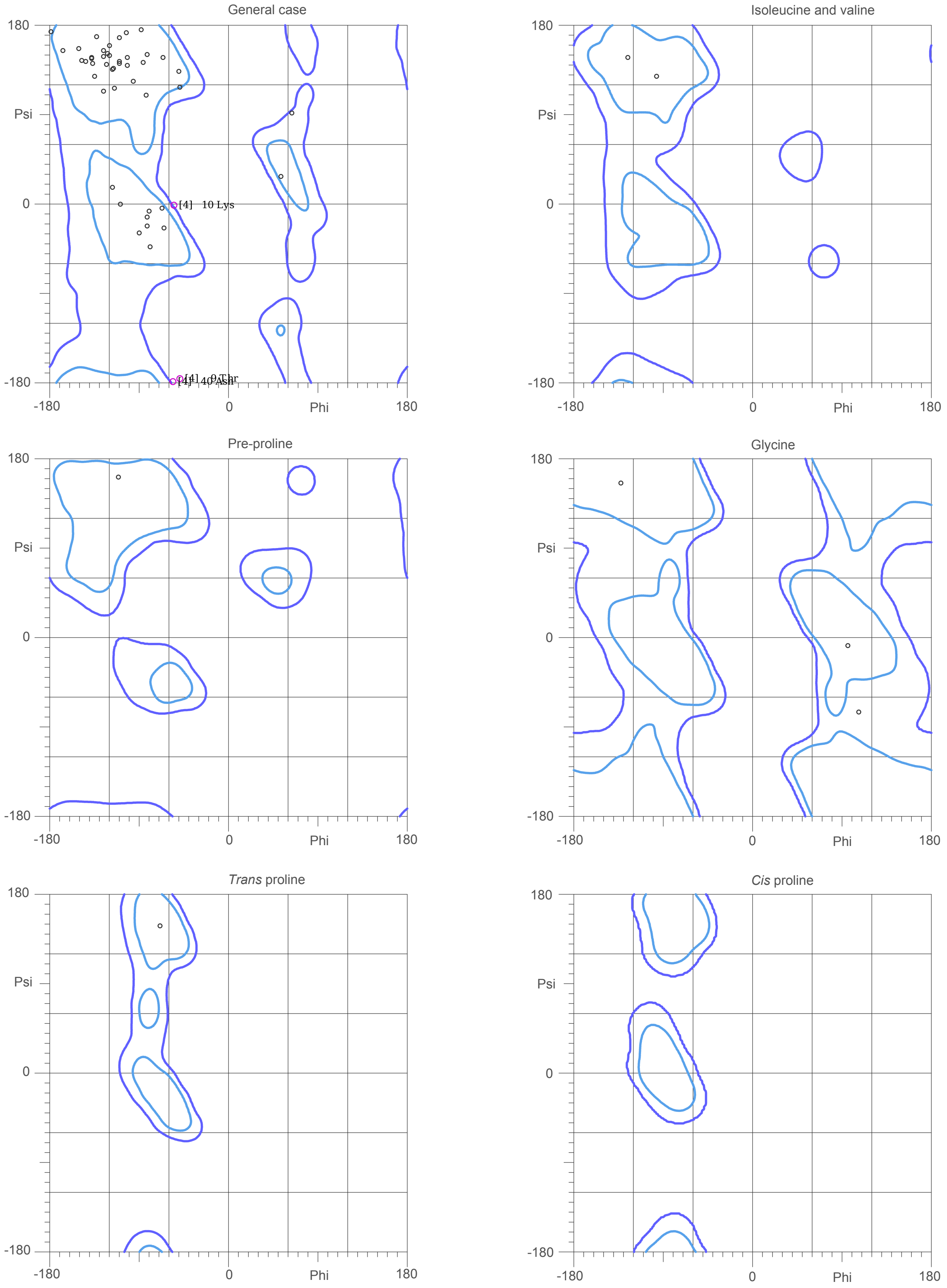

89.1% (49/55) of all residues were in favored (98%) regions.
94.5% (52/55) of all residues were in allowed (>99.8%) regions.

There were 3 outliers (phi, psi):
[4] 9 Thr (-49.7, -176.6)
[4] 10 Lys (-55.7, -1.5)
[4] 40 Asn (-56.4, -179.2)

MolProbity Ramachandran analysis

5oqsH.pdb, model 5

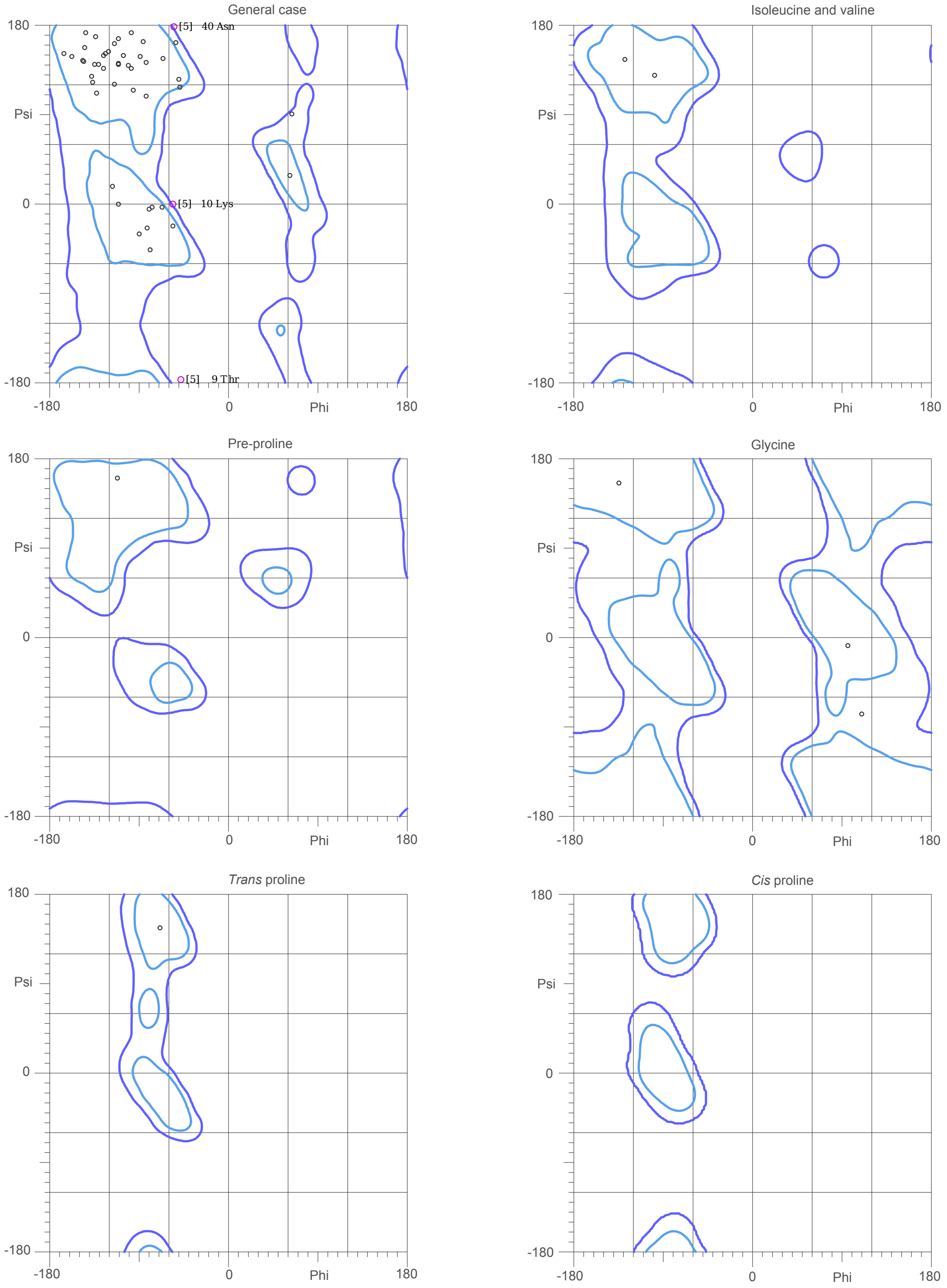

89.1% (49/55) of all residues were in favored (98%) regions.
94.5% (52/55) of all residues were in allowed (>99.8%) regions.

There were 3 outliers (phi, psi):
[5] 9 Thr (-48.7, -177.2)
[5] 10 Lys (-56.1, -0.4)
[5] 40 Asn (-55.5, 179.7)

Lovell, Davis, et al. Proteins 50:437 (2003)

MolProbity Ramachandran analysis

5oqsH.pdb, model 6

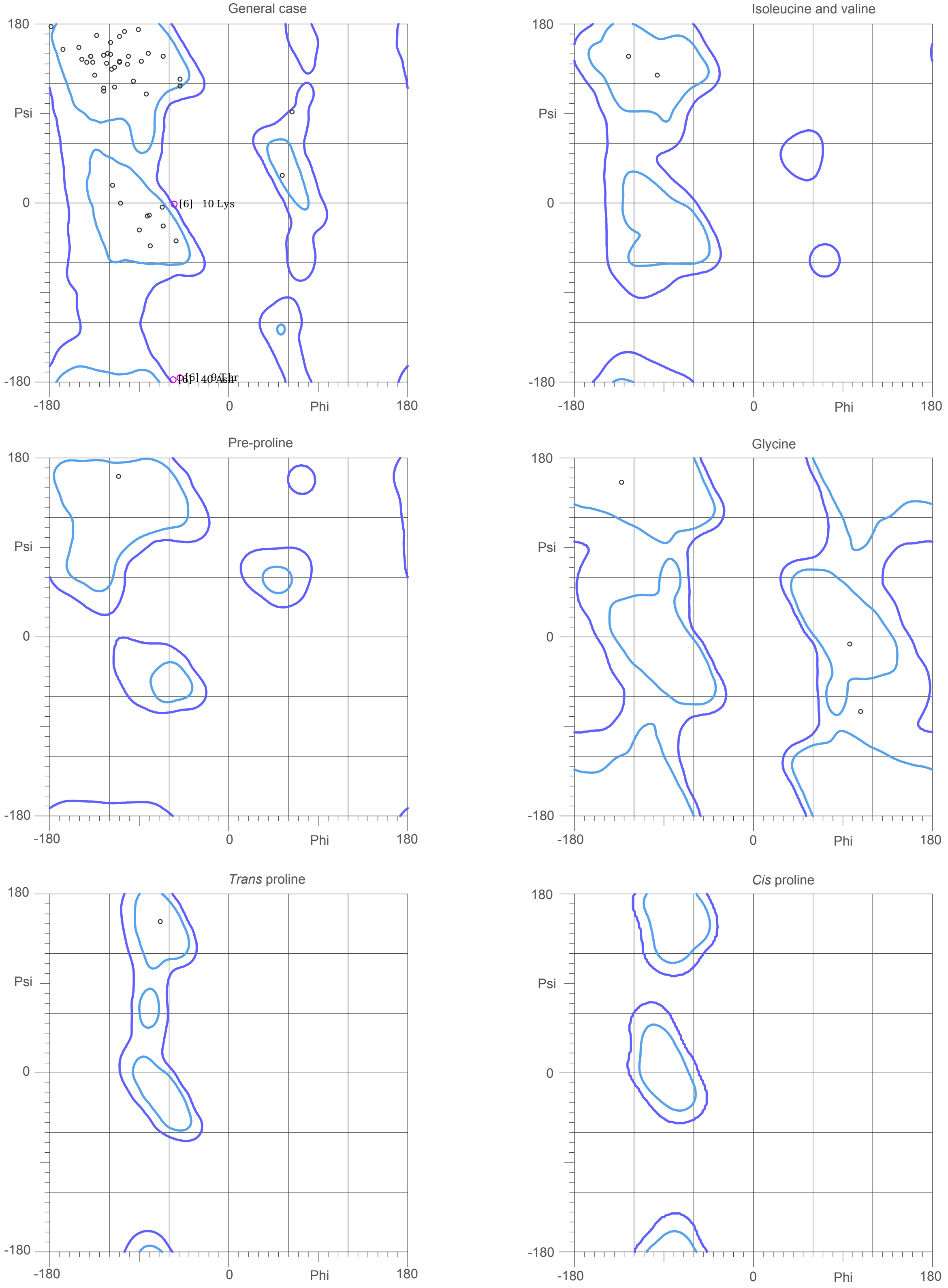

89.1% (49/55) of all residues were in favored (98%) regions.
94.5% (52/55) of all residues were in allowed (>99.8%) regions.

There were 3 outliers (phi, psi):
[6] 9 Thr (-49.8, -176.7)
[6] 10 Lys (-55.6, -1.7)
[6] 40 Asn (-56.7, -178.8)

MolProbity Ramachandran analysis

5oqsH.pdb, model 7

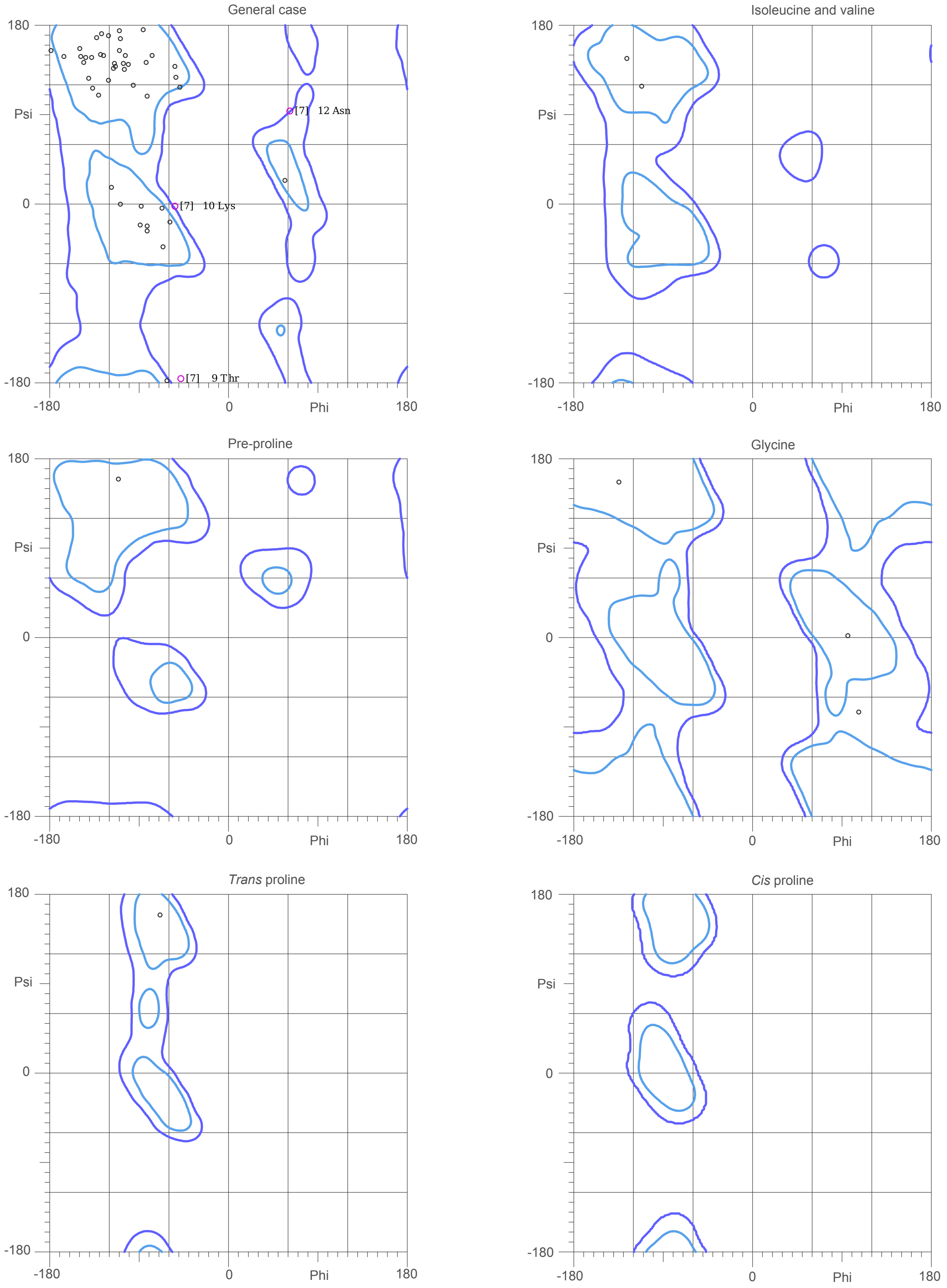

89.1% (49/55) of all residues were in favored (98%) regions.
94.5% (52/55) of all residues were in allowed (>99.8%) regions.

There were 3 outliers (phi, psi):
[7] 9 Thr (-48.9, -176.0)
[7] 10 Lys (-54.4, -2.2)
[7] 12 Asn (62.2, 94.9)

MolProbity Ramachandran analysis

5oqsH.pdb, model 8

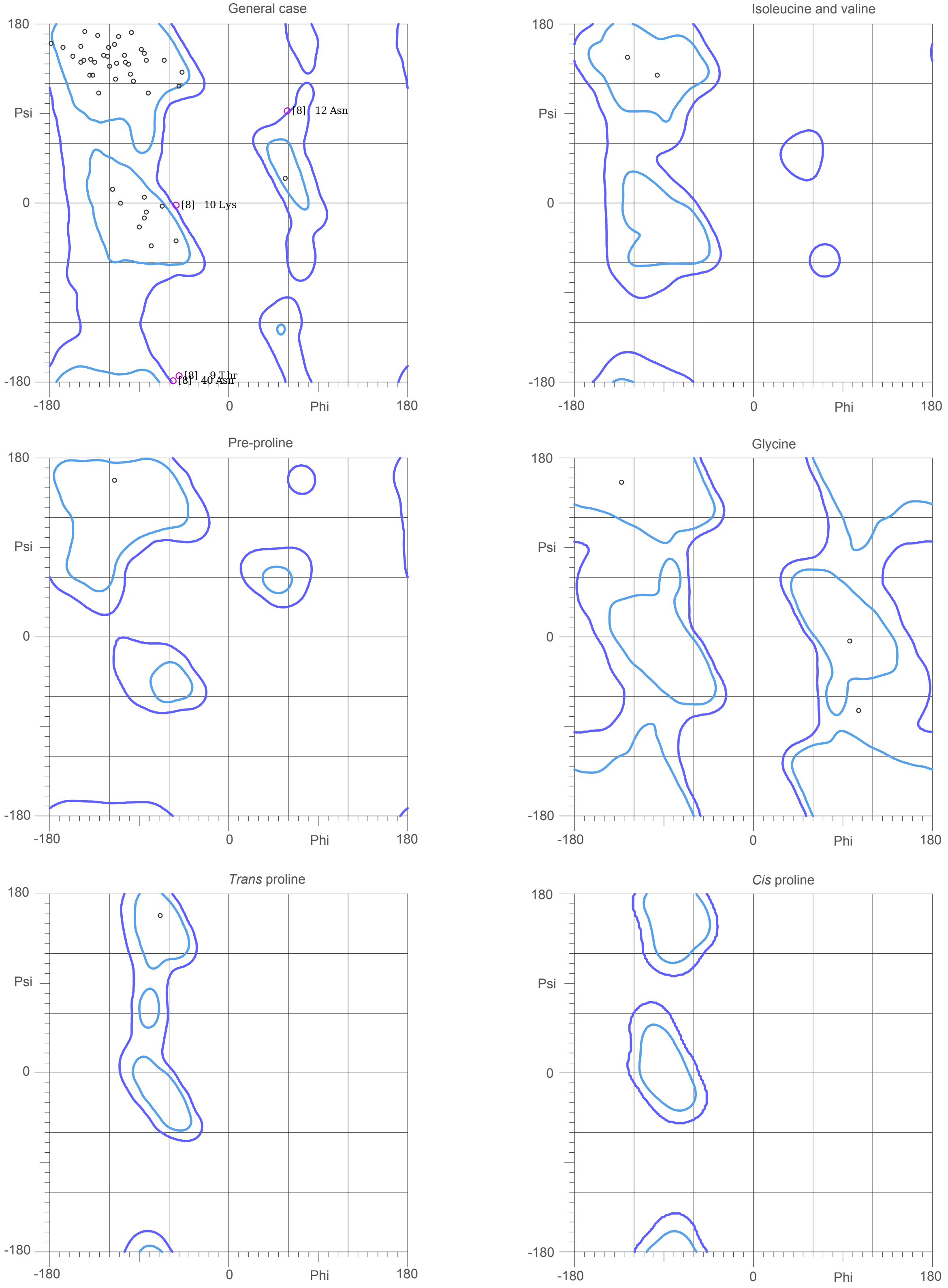

89.1% (49/55) of all residues were in favored (98%) regions.
92.7% (51/55) of all residues were in allowed (>99.8%) regions.

There were 4 outliers (phi, psi):
[8] 9 Thr (-50.3, -174.1)
[8] 10 Lys (-53.4, -2.9)
[8] 12 Asn (59.7, 93.6)
[8] 40 Asn (-56.3, -179.7)

MolProbity Ramachandran analysis

5oqsH.pdb, model 9

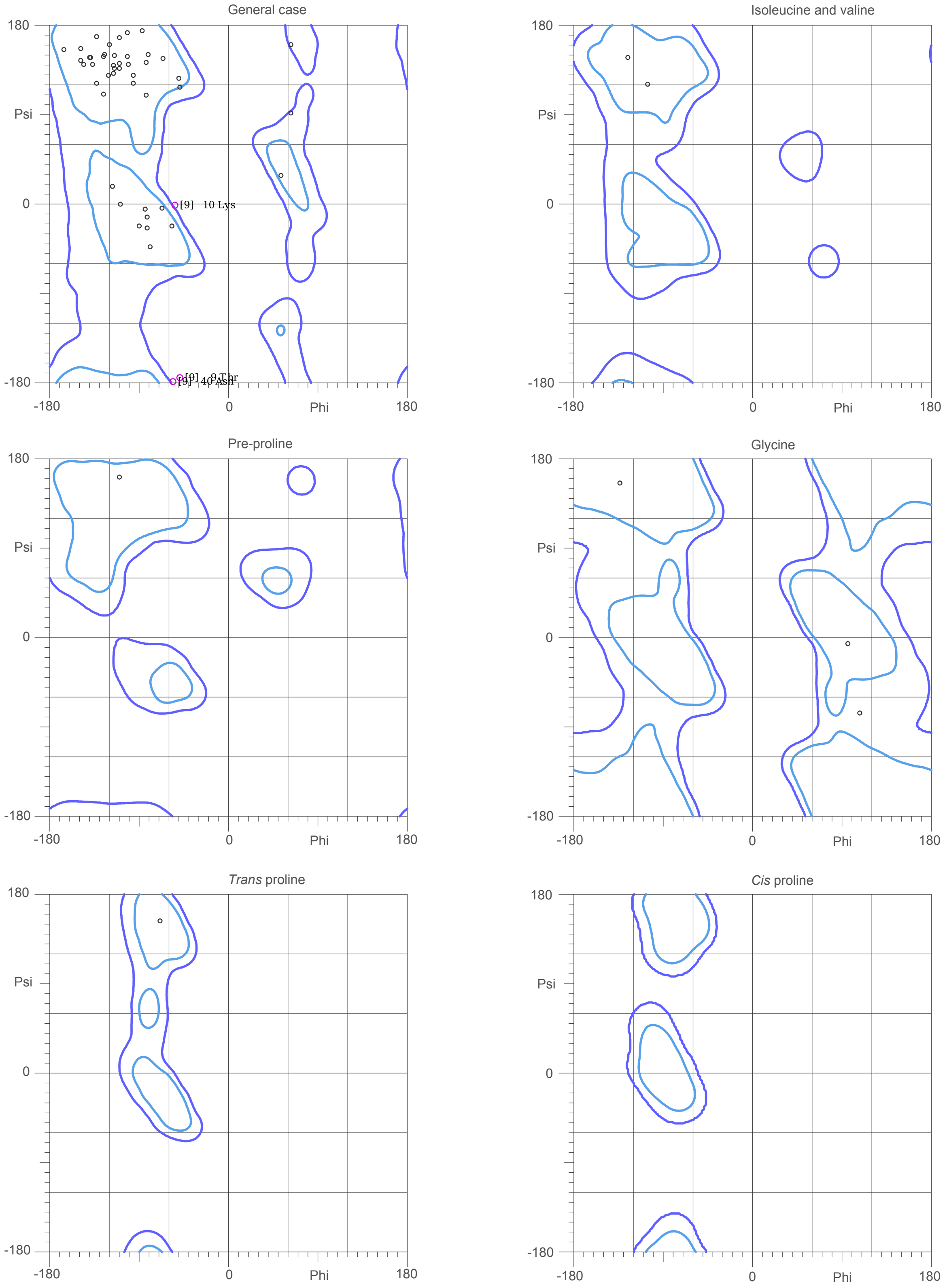

89.1% (49/55) of all residues were in favored (98%) regions.
94.5% (52/55) of all residues were in allowed (>99.8%) regions.

There were 3 outliers (phi, psi):
[9] 9 Thr (-49.9, -175.7)
[9] 10 Lys (-55.0, -1.9)
[9] 40 Asn (-56.3, -179.3)

MolProbity Ramachandran analysis

5oqsH.pdb, model 10

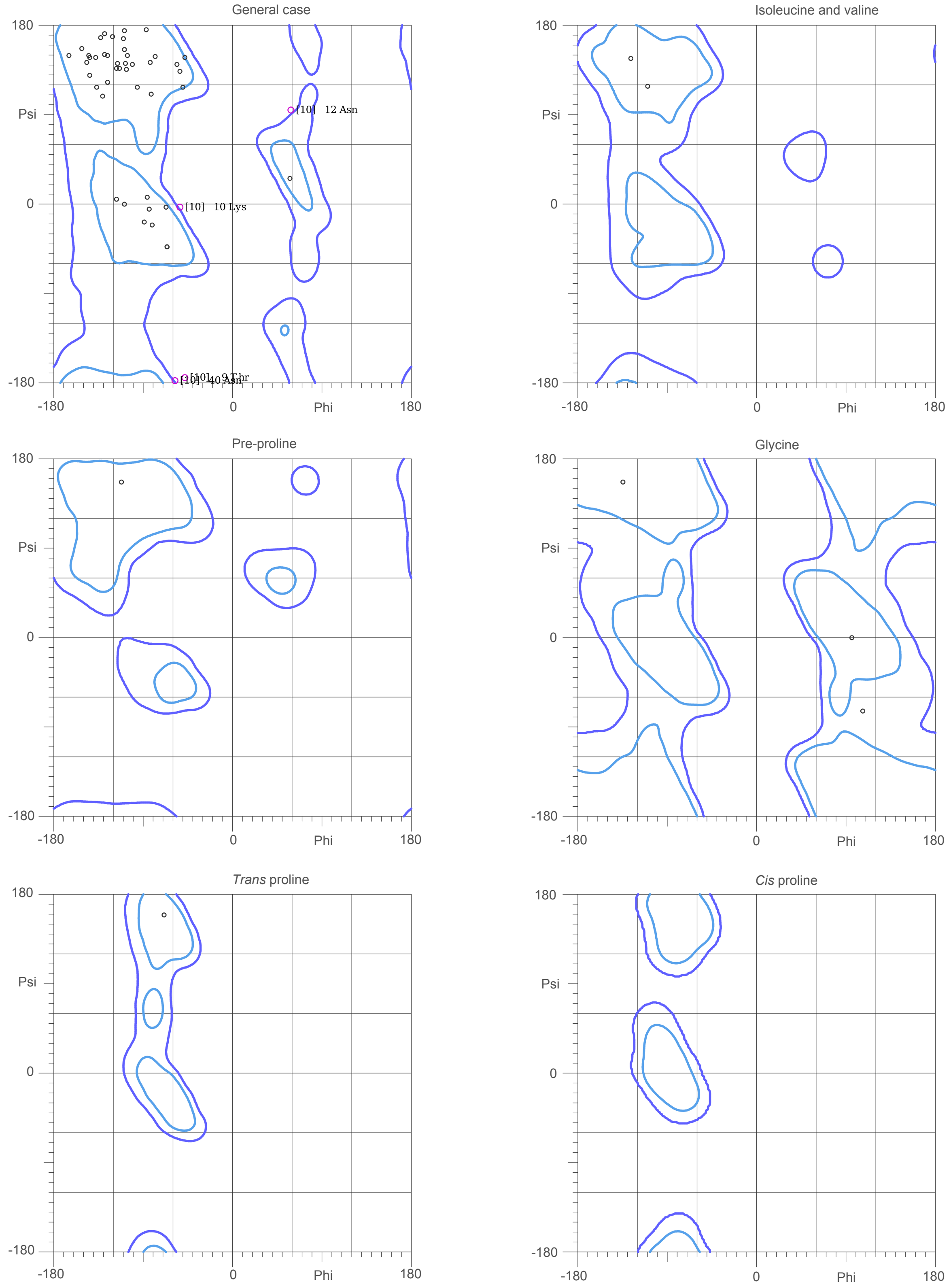

90.9% (50/55) of all residues were in favored (98%) regions.
92.7% (51/55) of all residues were in allowed (>99.8%) regions.

There were 4 outliers (phi, psi):
[10] 9 Thr (-49.0, -175.3)
[10] 10 Lys (-53.7, -3.0)
[10] 12 Asn (59.6, 95.2)
[10] 40 Asn (-58.2, -178.5)

MolProbity Ramachandran analysis

5oqsH.pdb, model 11

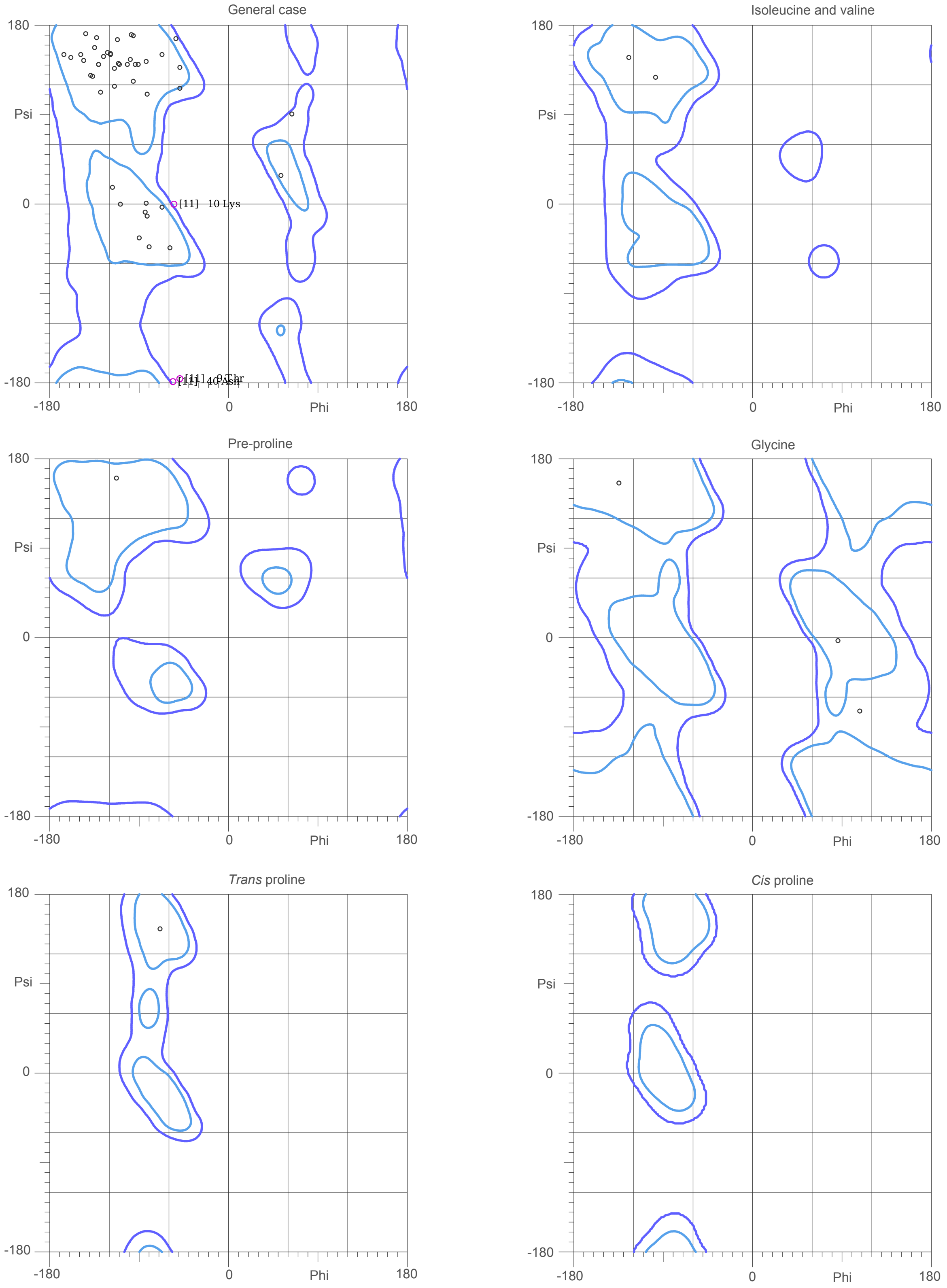

89.1% (49/55) of all residues were in favored (98%) regions.
94.5% (52/55) of all residues were in allowed (>99.8%) regions.

There were 3 outliers (phi, psi):
[11] 9 Thr (-49.3, -176.4)
[11] 10 Lys (-55.6, -0.8)
[11] 40 Asn (-56.2, -179.6)

MolProbity Ramachandran analysis

5oqsH.pdb, model 12

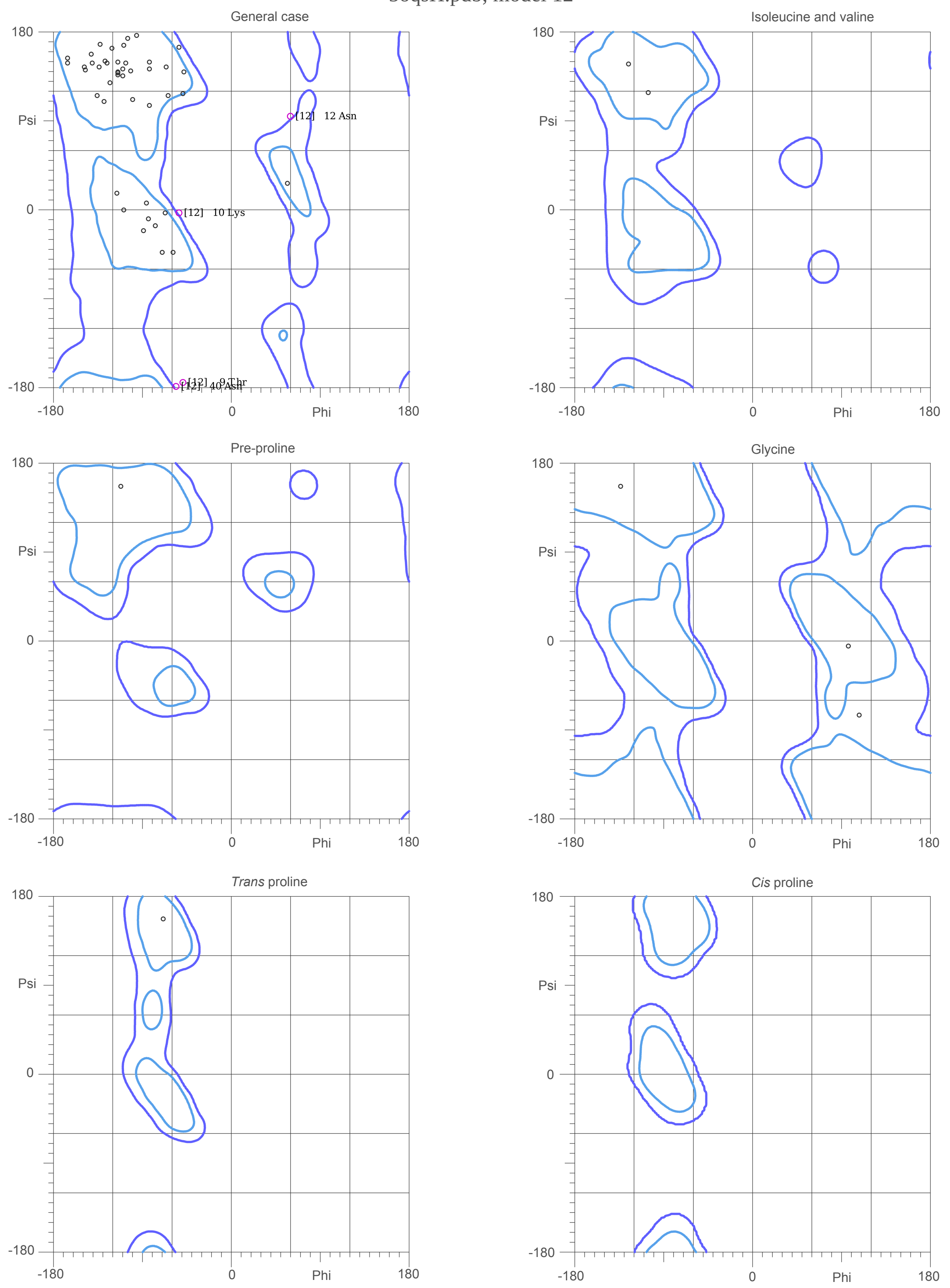

89.1% (49/55) of all residues were in favored (98%) regions.
92.7% (51/55) of all residues were in allowed (>99.8%) regions.

There were 4 outliers (phi, psi):
[12] 9 Thr (-49.7, -175.0)
[12] 10 Lys (-53.5, -3.4)
[12] 12 Asn (60.1, 95.8)
[12] 40 Asn (-56.2, -179.5)

MolProbity Ramachandran analysis

5oqsH.pdb, model 13

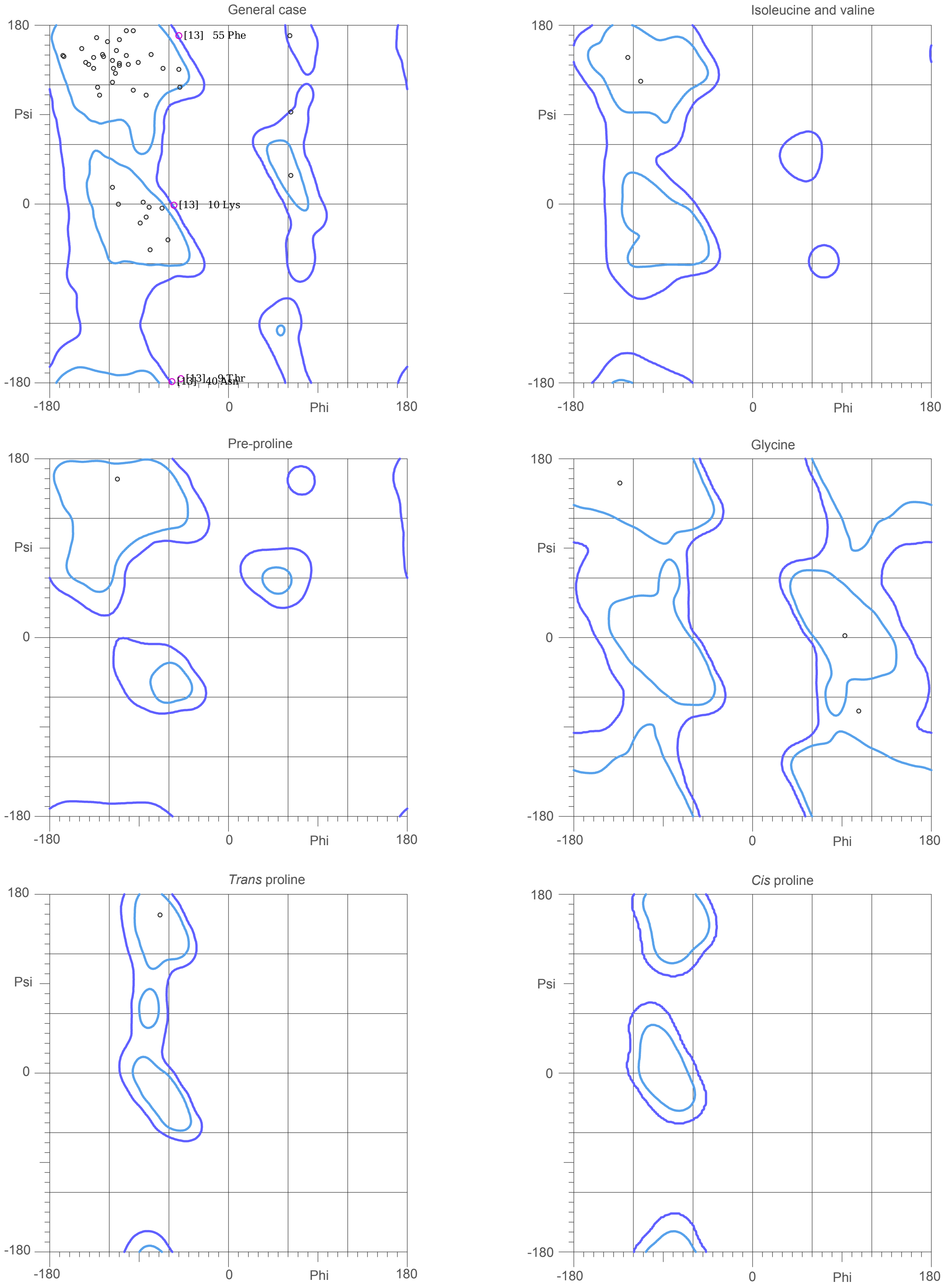

87.3% (48/55) of all residues were in favored (98%) regions.
92.7% (51/55) of all residues were in allowed (>99.8%) regions.

There were 4 outliers (phi, psi):
[13] 9 Thr (-48.6, -176.3)
[13] 10 Lys (-55.3, -1.0)
[13] 40 Asn (-57.6, -179.3)
[13] 55 Phe (-50.4, 170.7)

Lovell, Davis, et al. Proteins 50:437 (2003)

MolProbity Ramachandran analysis

5oqsH.pdb, model 14

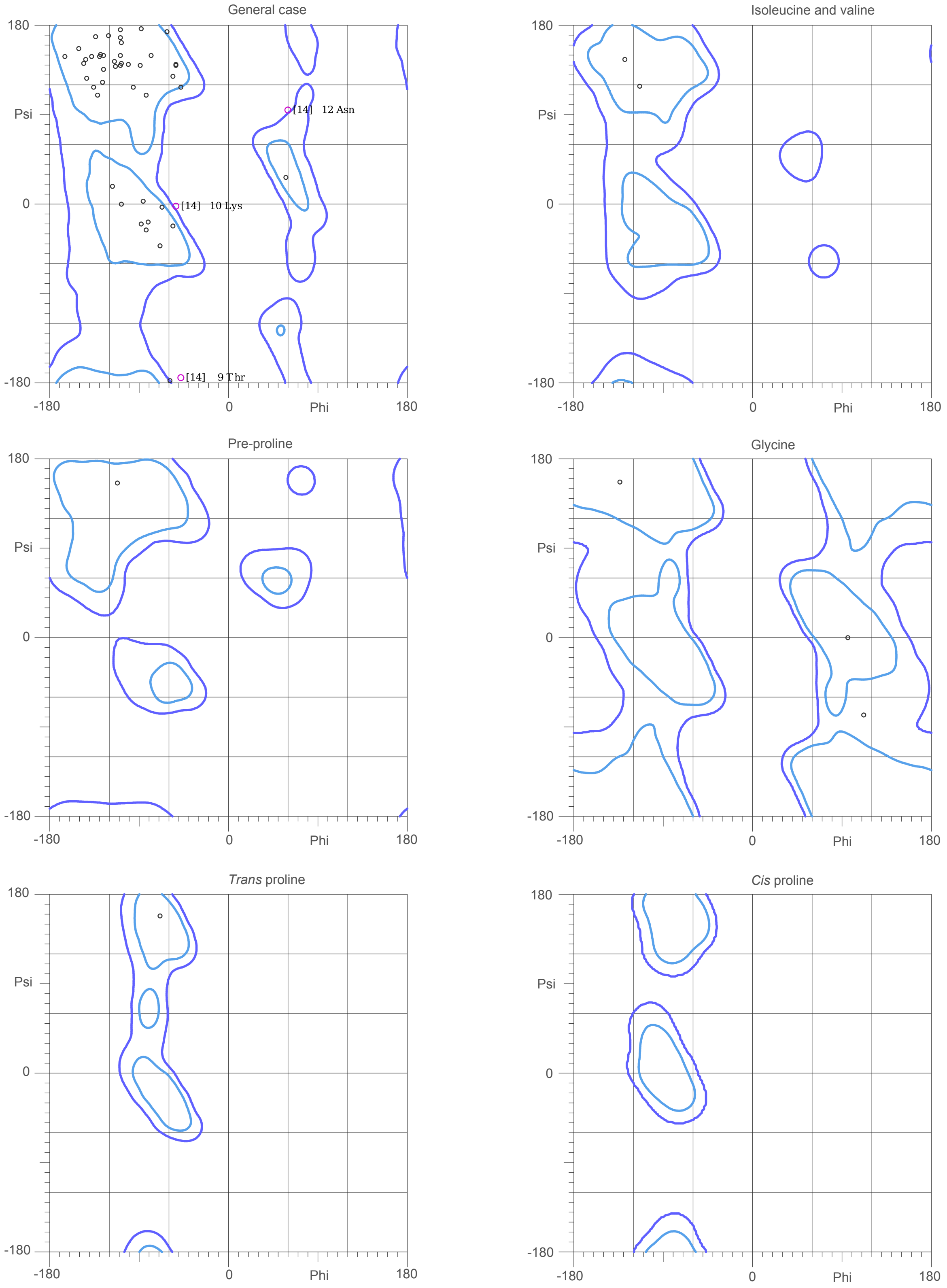

89.1% (49/55) of all residues were in favored (98%) regions.
94.5% (52/55) of all residues were in allowed (>99.8%) regions.

There were 3 outliers (phi, psi):
[14] 9 Thr (-48.6, -175.7)
[14] 10 Lys (-53.9, -2.7)
[14] 12 Asn (60.2, 95.1)

MolProbity Ramachandran analysis

5oqsH.pdb, model 15

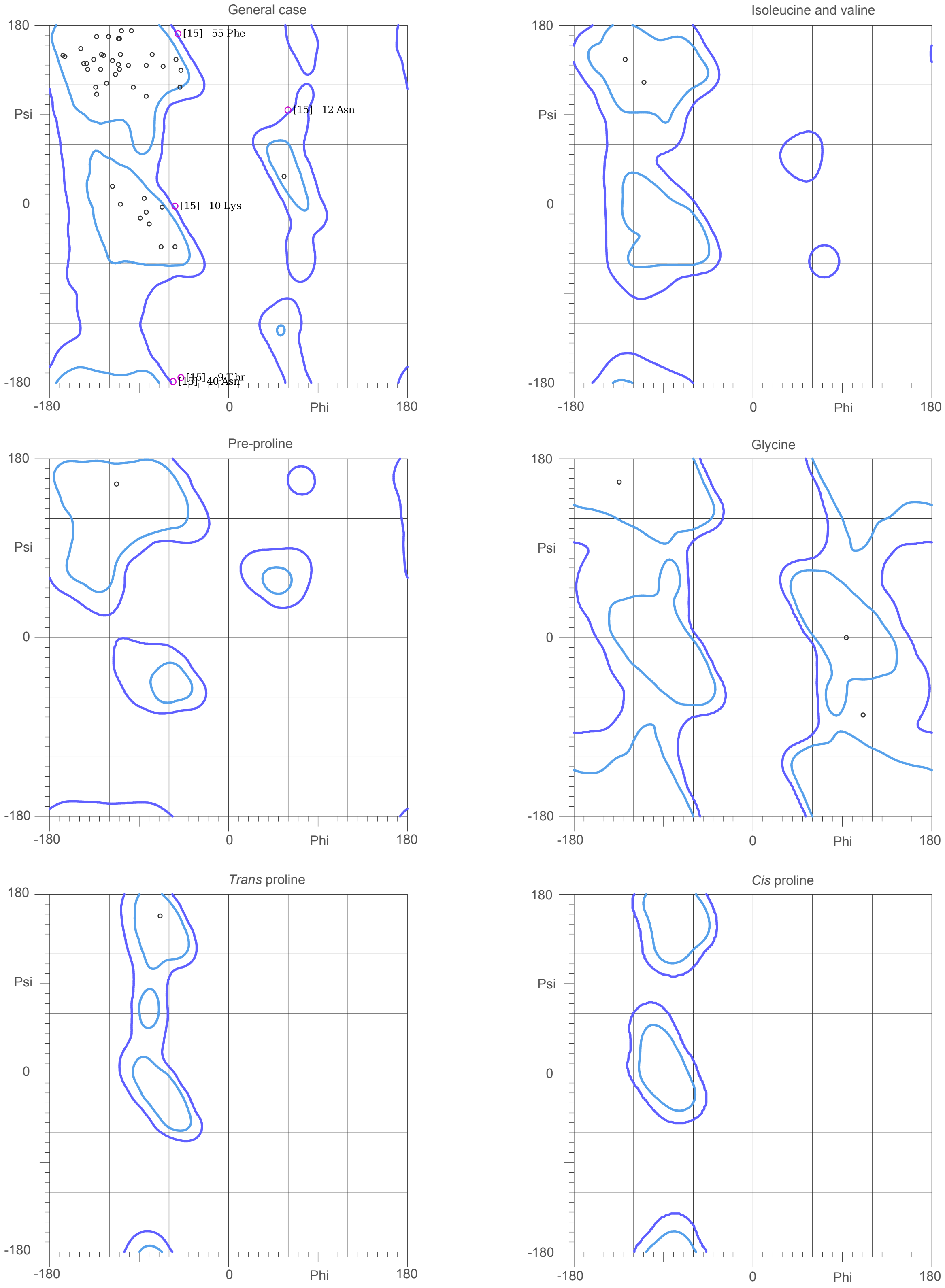

89.1% (49/55) of all residues were in favored (98%) regions.
90.9% (50/55) of all residues were in allowed (>99.8%) regions.

There were 5 outliers (phi, psi):
- [15] 9 Thr (-48.7, -175.7)
- [15] 10 Lys (-54.1, -2.7)
- [15] 12 Asn (60.8, 95.1)
- [15] 40 Asn (-56.7, -179.6)
- [15] 55 Phe (-51.5, 172.1)

http://kinemage.biochem.duke.edu

Lovell, Davis, et al. Proteins 50:437 (2003)

MolProbity Ramachandran analysis

5oqsH.pdb, model 16

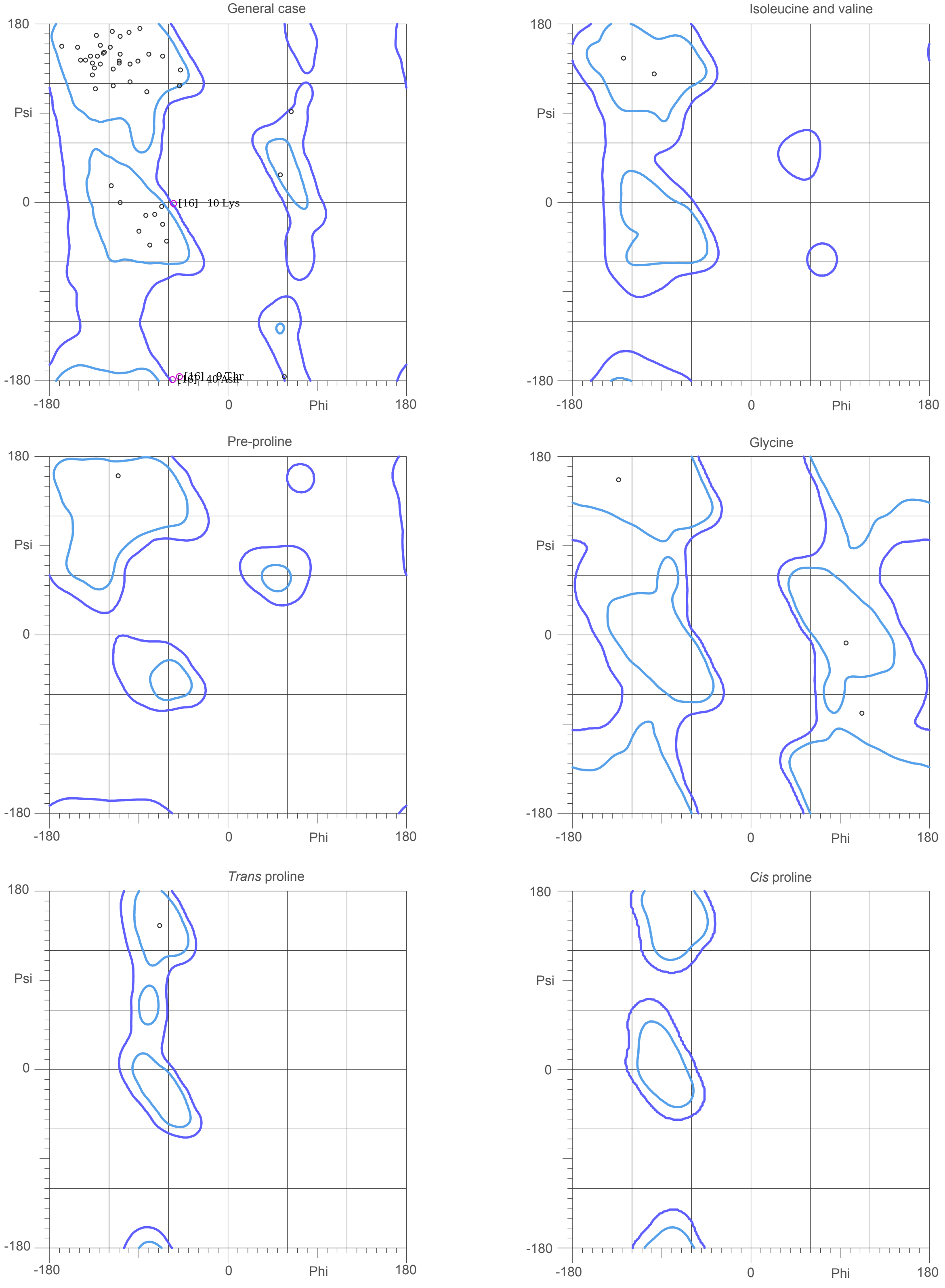

89.1% (49/55) of all residues were in favored (98%) regions.
94.5% (52/55) of all residues were in allowed (>99.8%) regions.

There were 3 outliers (phi, psi):
[16] 9 Thr (-49.8, -176.5)
[16] 10 Lys (-55.3, -1.6)
[16] 40 Asn (-56.3, -179.3)

Lovell, Davis, et al. Proteins 50:437 (2003)

MolProbity Ramachandran analysis

5oqsH.pdb, model 17

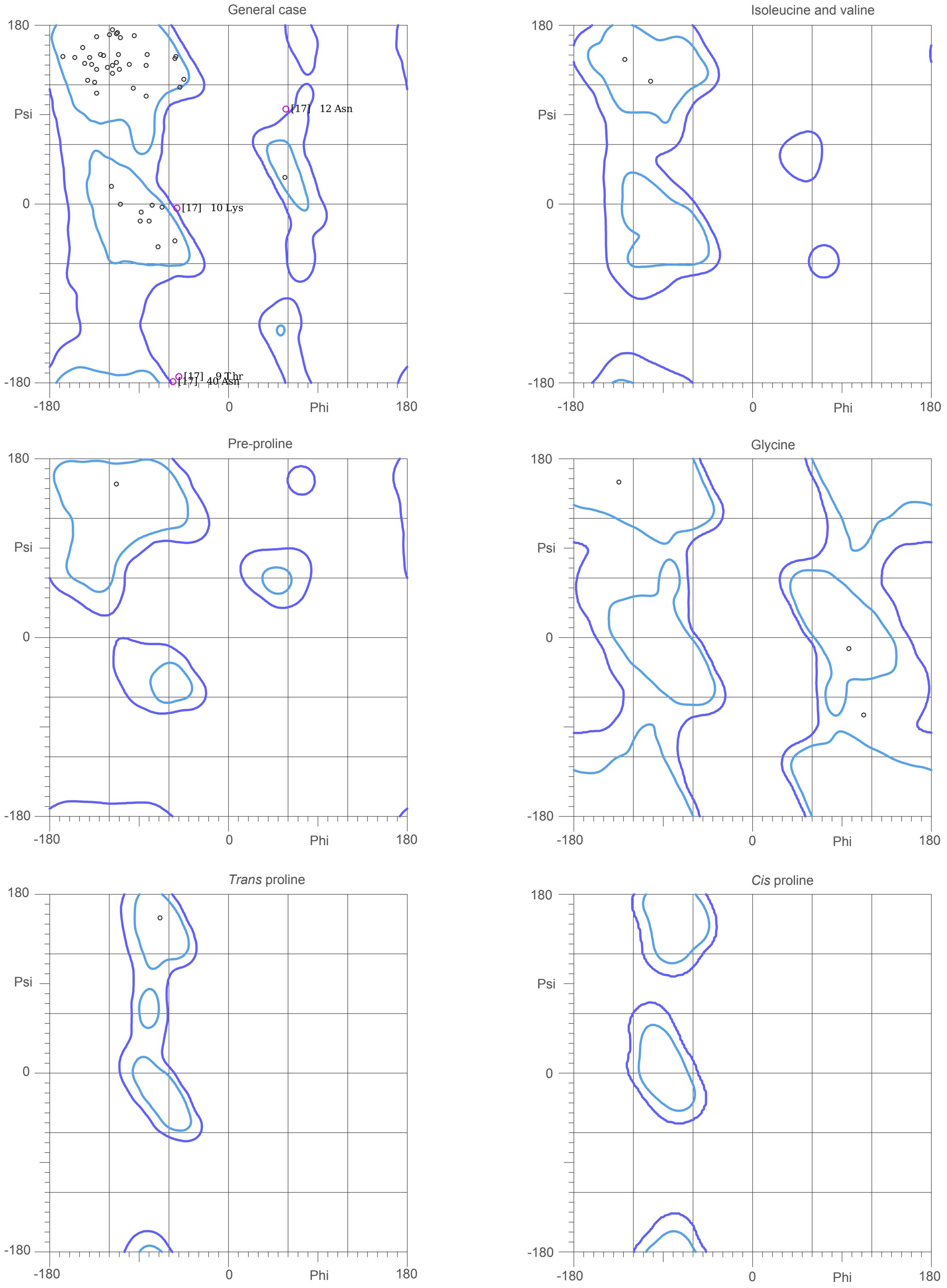

90.9% (50/55) of all residues were in favored (98%) regions.
92.7% (51/55) of all residues were in allowed (>99.8%) regions.

There were 4 outliers (phi, psi):
[17] 9 Thr (-50.0, -174.4)
[17] 10 Lys (-52.5, -4.4)
[17] 12 Asn (58.2, 96.8)
[17] 40 Asn (-56.4, -179.2)

MolProbity Ramachandran analysis

5oqsH.pdb, model 18

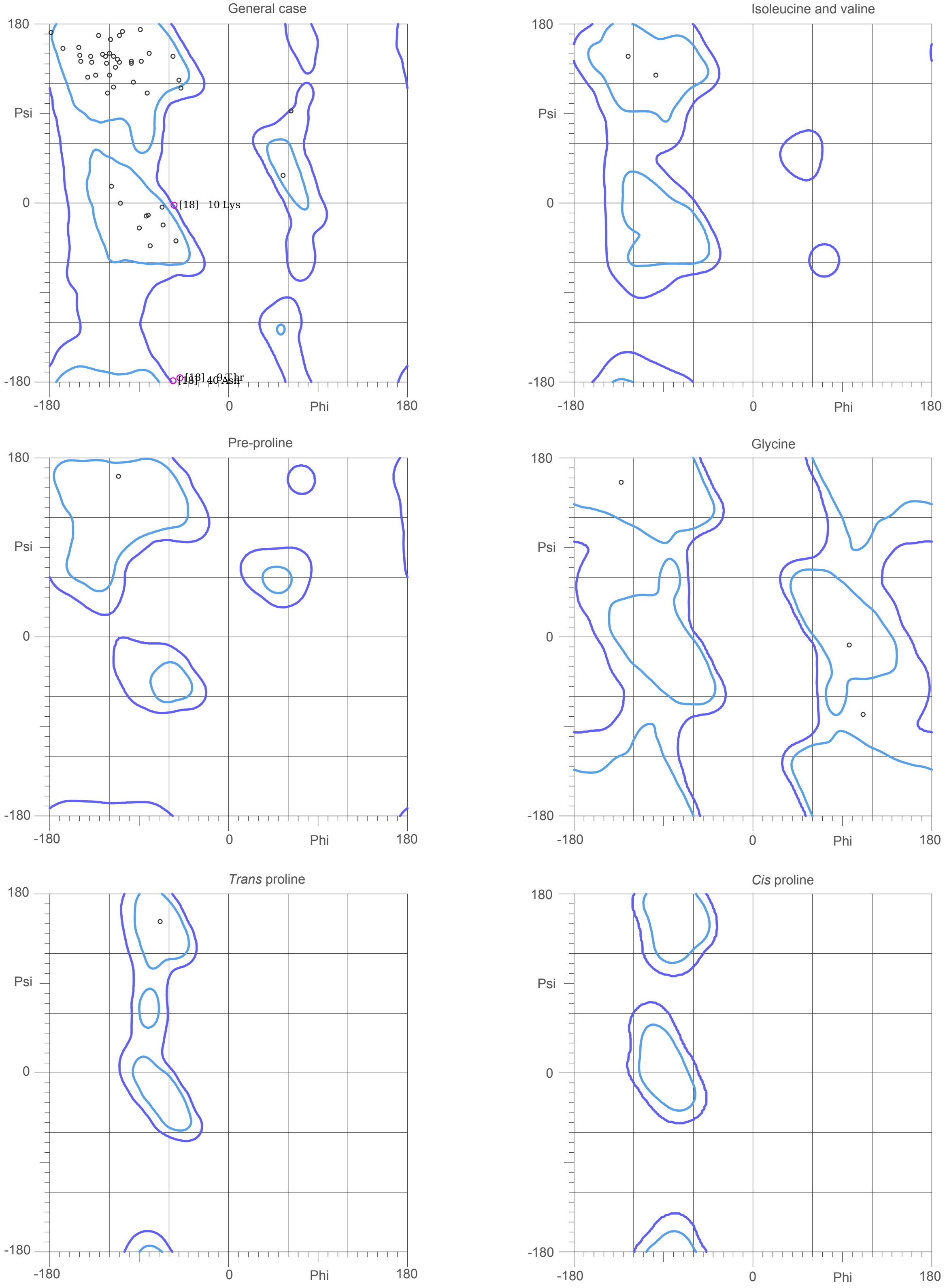

87.3% (48/55) of all residues were in favored (98%) regions.
94.5% (52/55) of all residues were in allowed (>99.8%) regions.

There were 3 outliers (phi, psi):
[18] 9 Thr (-49.9, -176.2)
[18] 10 Lys (-55.2, -2.1)
[18] 40 Asn (-56.3, -179.2)

http://kinemage.biochem.duke.edu

Lovell, Davis, et al. Proteins 50:437 (2003)

MolProbity Ramachandran analysis

5oqsH.pdb, model 19

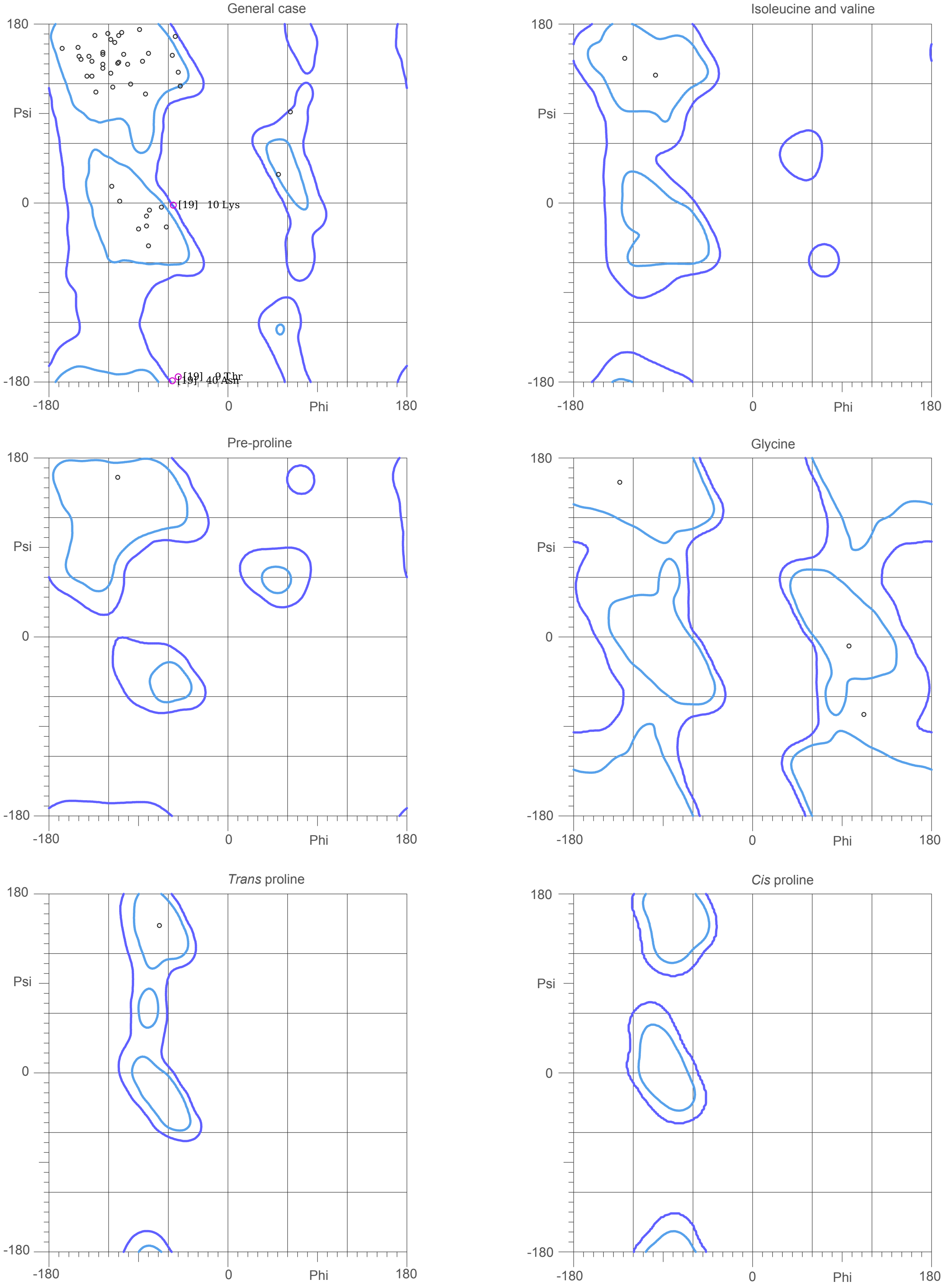

89.1% (49/55) of all residues were in favored (98%) regions.
94.5% (52/55) of all residues were in allowed (>99.8%) regions.

There were 3 outliers (phi, psi):
[19] 9 Thr (-50.6, -175.8)
[19] 10 Lys (-55.0, -2.3)
[19] 40 Asn (-56.0, -179.3)

Lovell, Davis, et al. Proteins 50:437 (2003)

MolProbity Ramachandran analysis

5oqsH.pdb, model 20

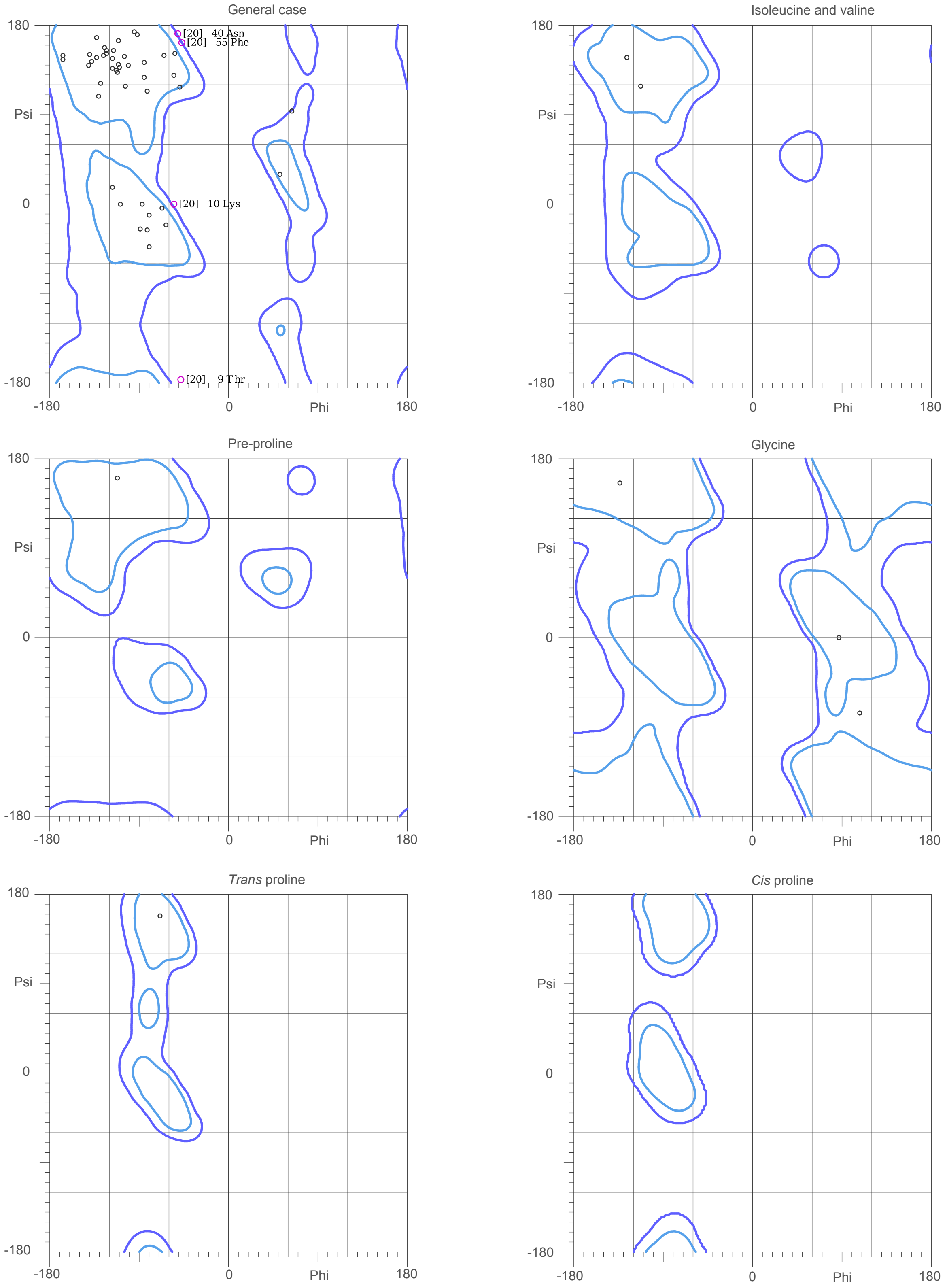

89.1% (49/55) of all residues were in favored (98%) regions.
92.7% (51/55) of all residues were in allowed (>99.8%) regions.

There were 4 outliers (phi, psi):
[20] 9 Thr (-48.1, -177.4)
[20] 10 Lys (-55.8, -0.9)
[20] 40 Asn (-51.2, 172.6)
[20] 55 Phe (-47.0, 163.7)

http://kinemage.biochem.duke.edu

Lovell, Davis, et al. Proteins 50:437 (2003)
