# Supplementary material for: Small Disulfide Proteins with Antifungal Impact: NMR Experimental Structures as Compared to Models of Alphafold Versions
Source: Int J Mol Sci. 2025 Jan 31;26(3):1247. doi: 10.3390/ijms26031247 (PMC11818080; doi:10.3390/ijms26031247)
Supplement: Supplementary file 1 [file ijms-26-01247-s001.zip › Figure S7d. NMR-PAFB.pdf]

MolProbity Ramachandran analysis

2nc2H.pdb, all models

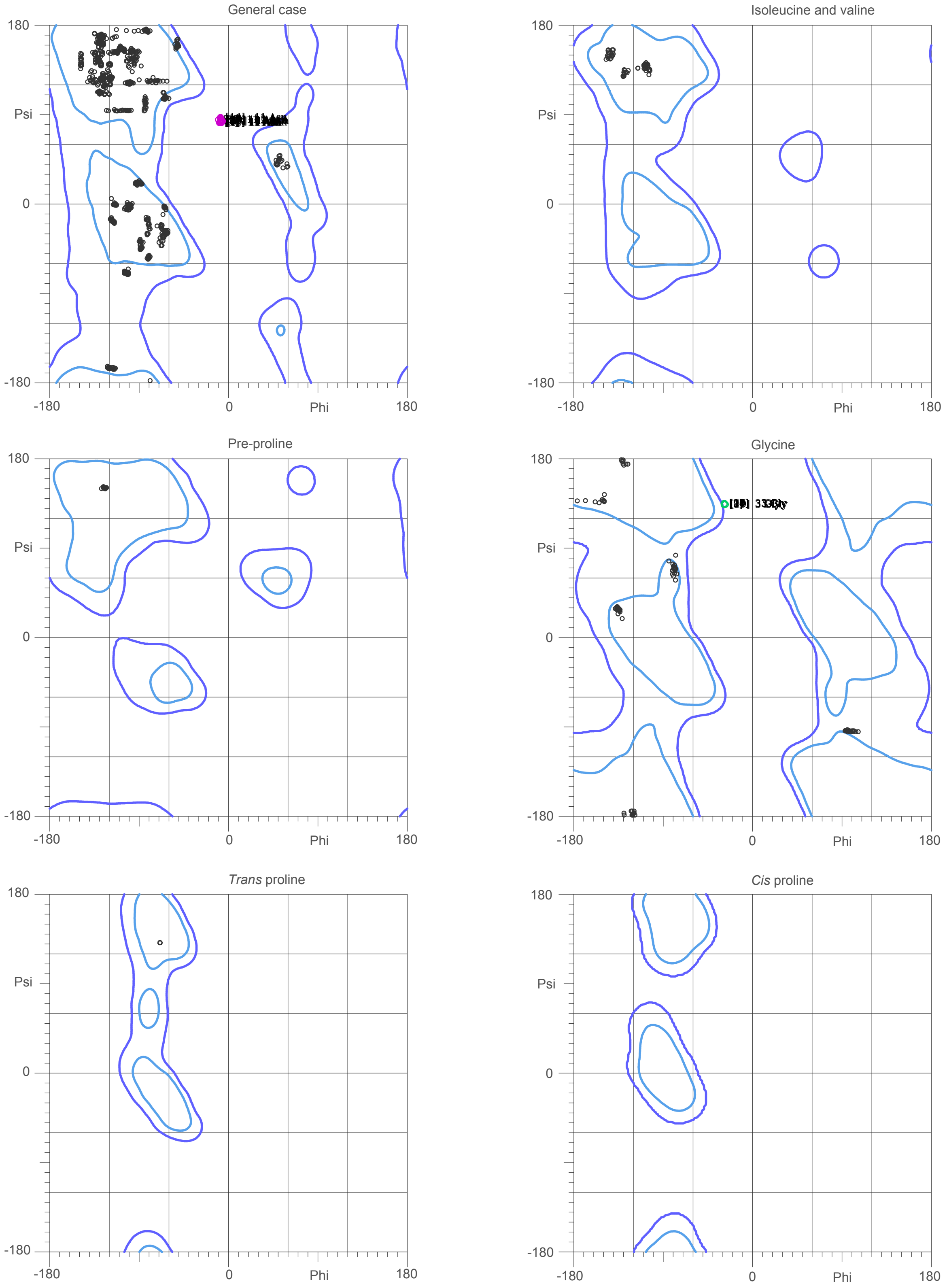

86.3% (932/1080) of all residues were in favored (98%) regions.
97.2% (1050/1080) of all residues were in allowed (>99.8%) regions.

There were 30 outliers (phi, psi):

[1] 11 Asn (-8.2, 83.2)
[2] 11 Asn (-8.0, 84.5)
[3] 11 Asn (-8.0, 85.8)
[4] 11 Asn (-9.0, 82.6)
[5] 11 Asn (-10.1, 85.1)
[6] 11 Asn (-8.6, 83.6)
[7] 3 Gly (-29.6, 135.2)
[7] 11 Asn (-9.0, 82.2)
[8] 3 Gly (-29.8, 134.6)
[8] 11 Asn (-8.5, 84.6)
[9] 3 Gly (-29.4, 135.1)
[9] 11 Asn (-7.8, 83.0)
[10] 3 Gly (-29.2, 135.1)
[10] 11 Asn (-8.0, 85.5)
[11] 3 Gly (-29.4, 135.3)
[11] 11 Asn (-8.3, 84.7)
[12] 11 Asn (-8.6, 83.0)
[13] 11 Asn (-7.7, 84.3)
[14] 3 Gly (-29.6, 135.5)
[14] 11 Asn (-8.9, 87.2)
[15] 3 Gly (-29.3, 135.2)
[15] 11 Asn (-8.3, 85.7)
[16] 11 Asn (-8.6, 83.5)
[17] 3 Gly (-29.2, 135.1)
[17] 11 Asn (-8.4, 84.0)
[18] 11 Asn (-6.9, 83.3)
[19] 3 Gly (-28.6, 134.7)
[19] 11 Asn (-8.4, 86.2)
[20] 3 Gly (-29.5, 135.4)
[20] 11 Asn (-7.2, 84.6)

http://kinemage.biochem.duke.edu

Lovell, Davis, et al. Proteins 50:437 (2003)

MolProbity Ramachandran analysis

2nc2H.pdb, model 1

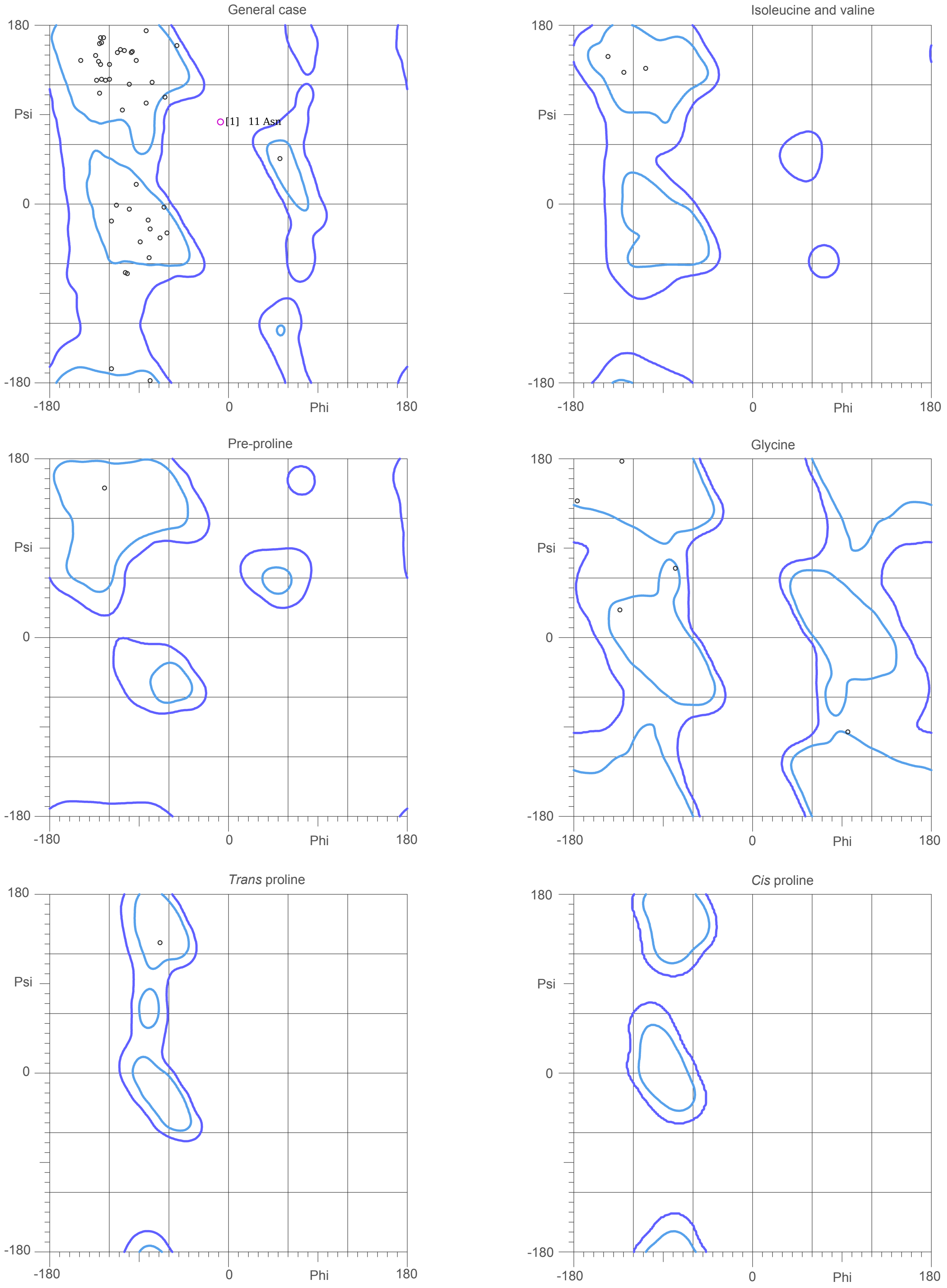

87.0% (47/54) of all residues were in favored (98%) regions.
98.1% (53/54) of all residues were in allowed (>99.8%) regions.

There were 1 outliers (phi, psi):
[1] 11 Asn (-8.2, 83.2)

MolProbity Ramachandran analysis

2nc2H.pdb, model 2

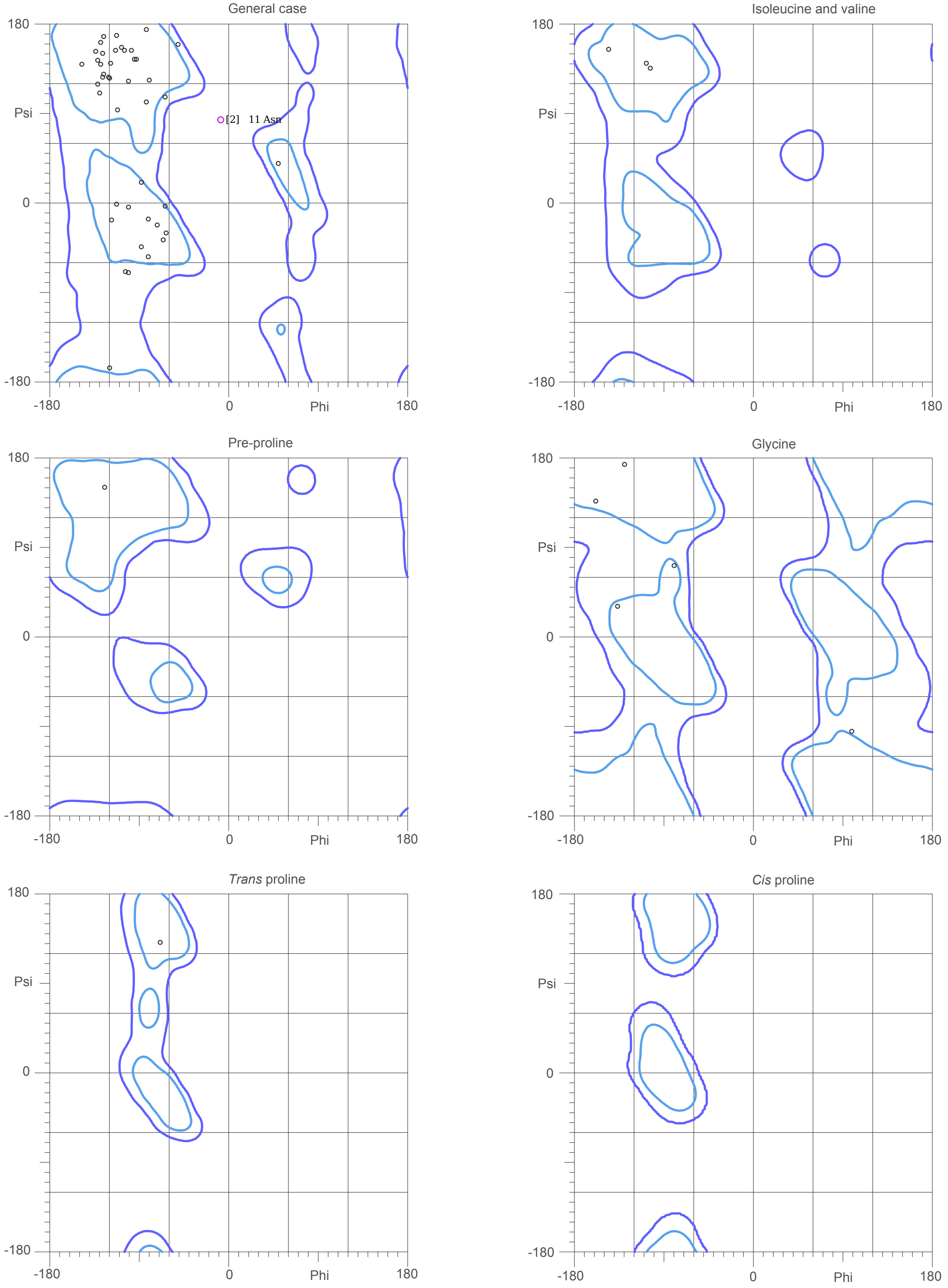

87.0% (47/54) of all residues were in favored (98%) regions.
98.1% (53/54) of all residues were in allowed (>99.8%) regions.

There were 1 outliers (phi, psi):
[2] 11 Asn (-8.0, 84.5)

MolProbity Ramachandran analysis

2nc2H.pdb, model 3

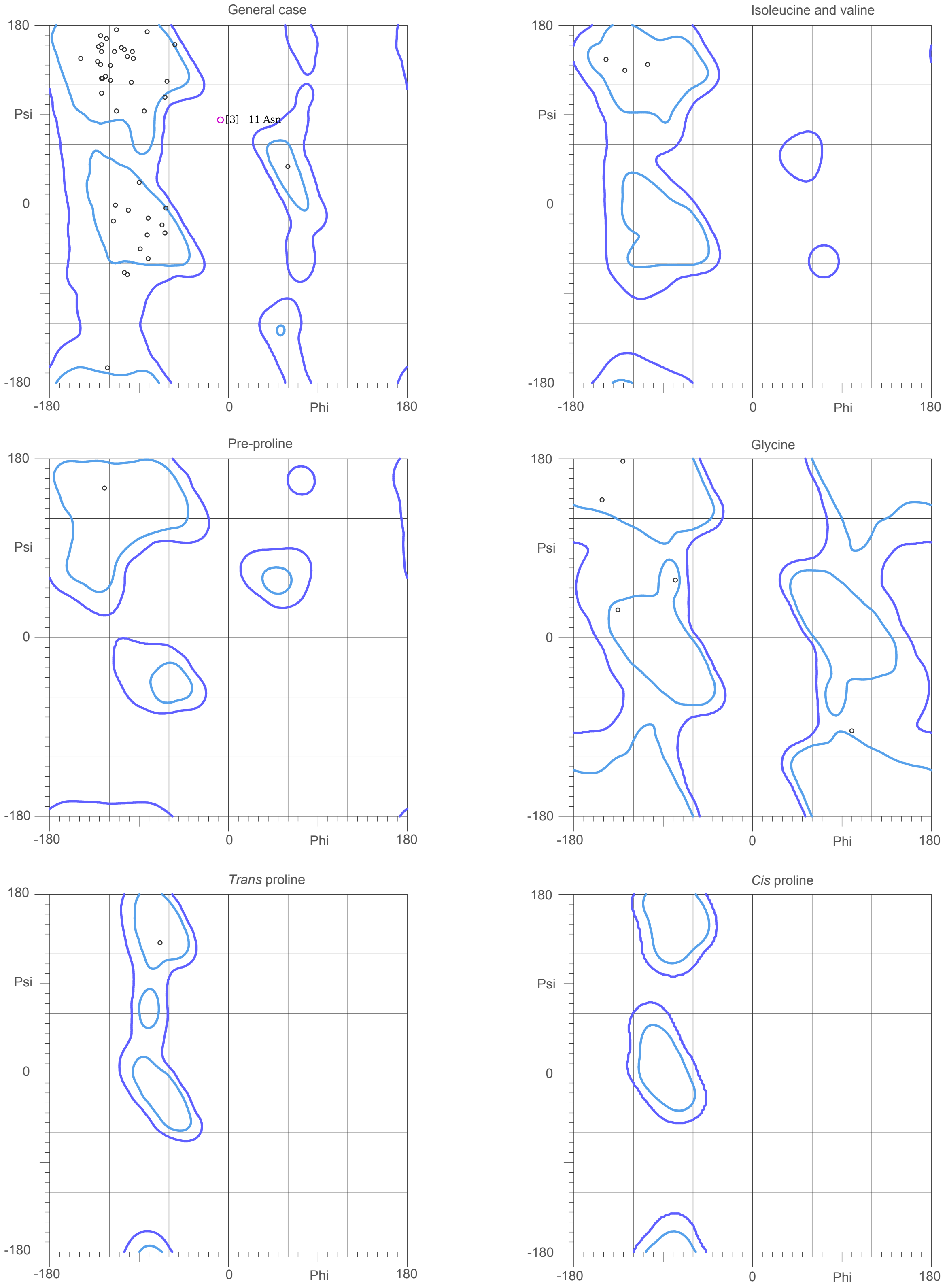

87.0% (47/54) of all residues were in favored (98%) regions.
98.1% (53/54) of all residues were in allowed (>99.8%) regions.

There were 1 outliers (phi, psi):
[3] 11 Asn (-8.0, 85.8)

MolProbity Ramachandran analysis

2nc2H.pdb, model 4

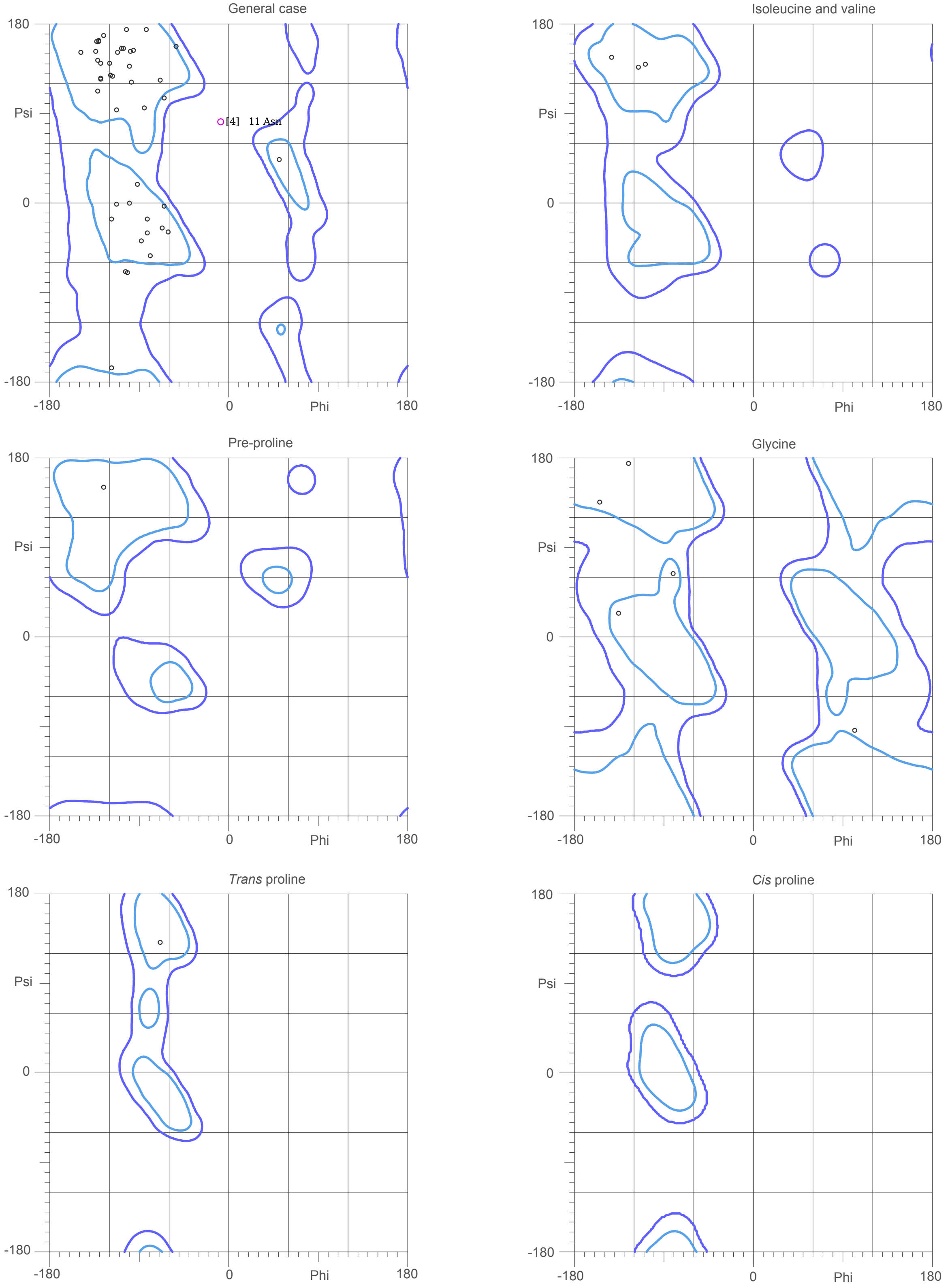

87.0% (47/54) of all residues were in favored (98%) regions.
98.1% (53/54) of all residues were in allowed (>99.8%) regions.

There were 1 outliers (phi, psi):
[4] 11 Asn (-9.0, 82.6)

MolProbity Ramachandran analysis

2nc2H.pdb, model 5

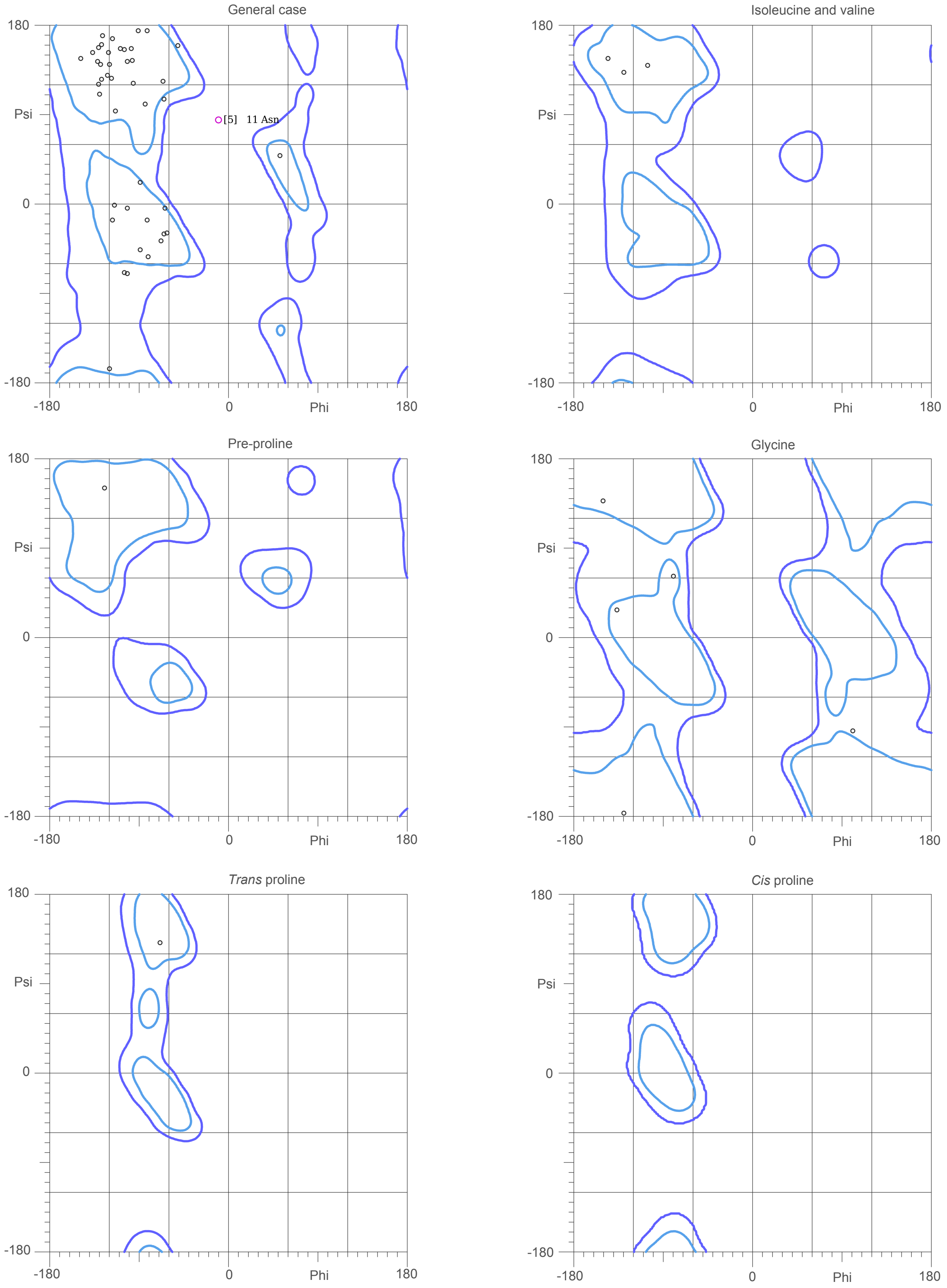

87.0% (47/54) of all residues were in favored (98%) regions.
98.1% (53/54) of all residues were in allowed (>99.8%) regions.

There were 1 outliers (phi, psi):
[5] 11 Asn (-10.1, 85.1)

MolProbity Ramachandran analysis

2nc2H.pdb, model 6

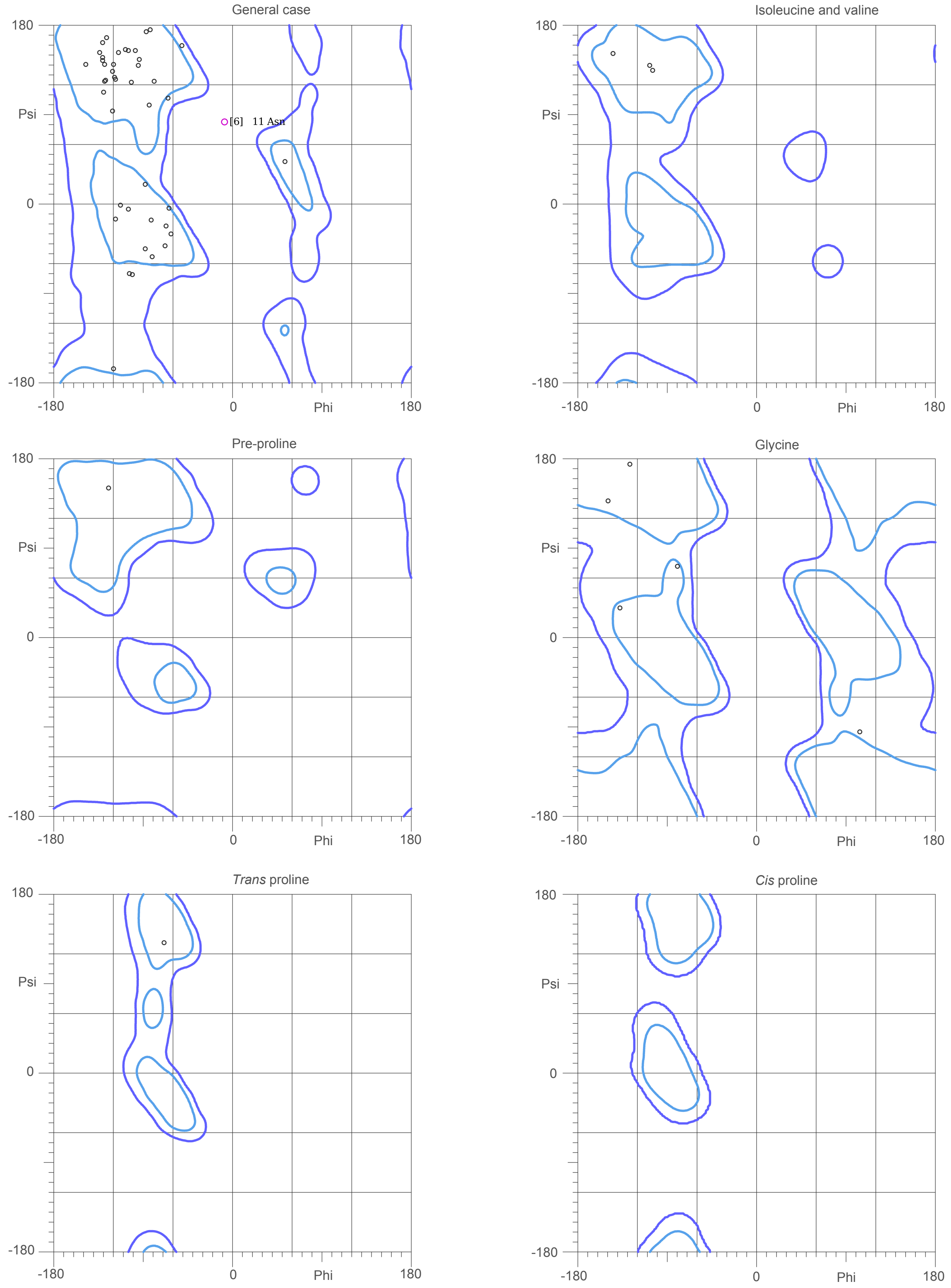

87.0% (47/54) of all residues were in favored (98%) regions.
98.1% (53/54) of all residues were in allowed (>99.8%) regions.

There were 1 outliers (phi, psi):
[6] 11 Asn (-8.6, 83.6)

MolProbity Ramachandran analysis

2nc2H.pdb, model 7

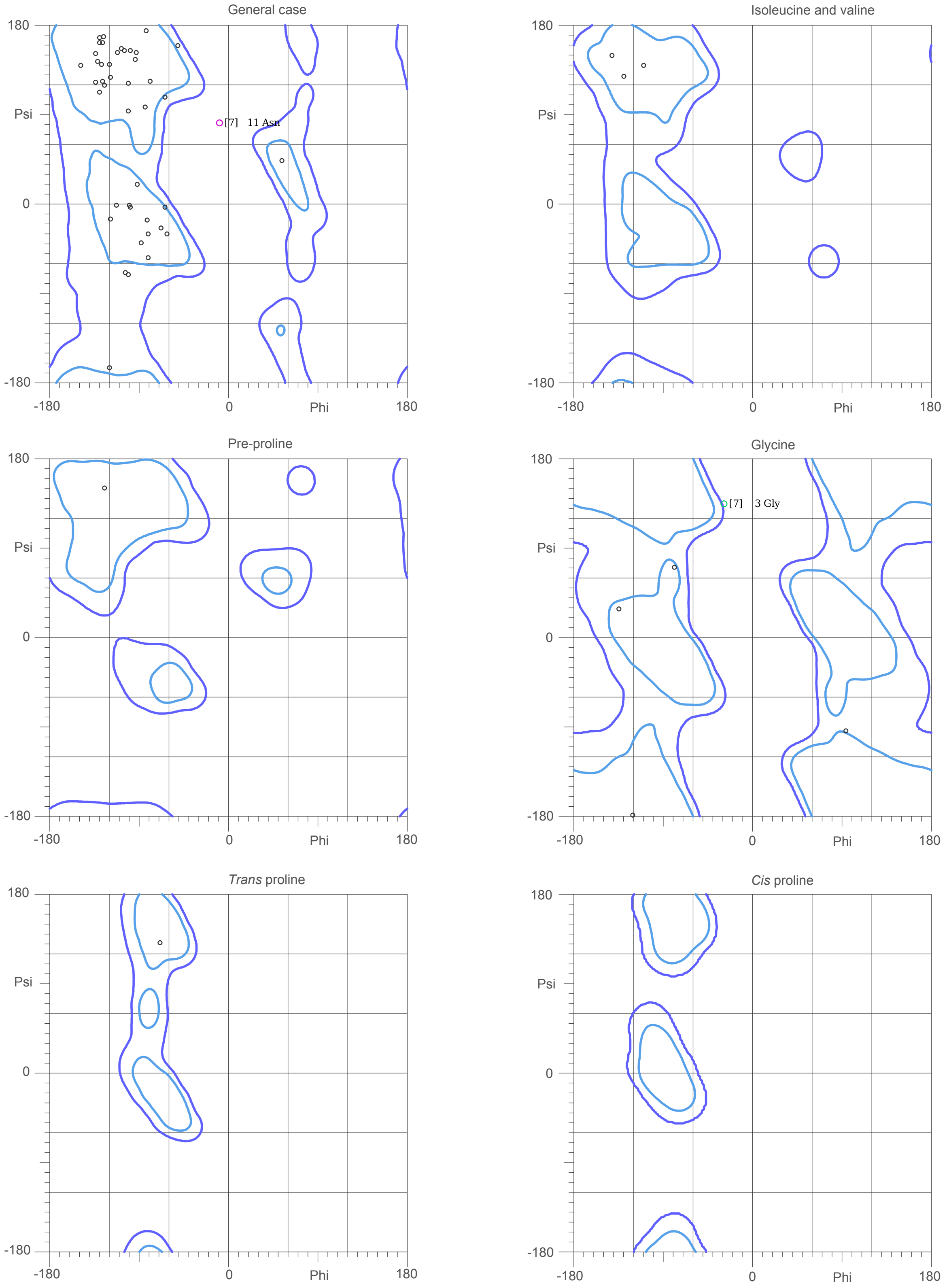

85.2% (46/54) of all residues were in favored (98%) regions.
96.3% (52/54) of all residues were in allowed (>99.8%) regions.

There were 2 outliers (phi, psi):
[7] 3 Gly (-29.6, 135.2)
[7] 11 Asn (-9.0, 82.2)

MolProbity Ramachandran analysis

2nc2H.pdb, model 8

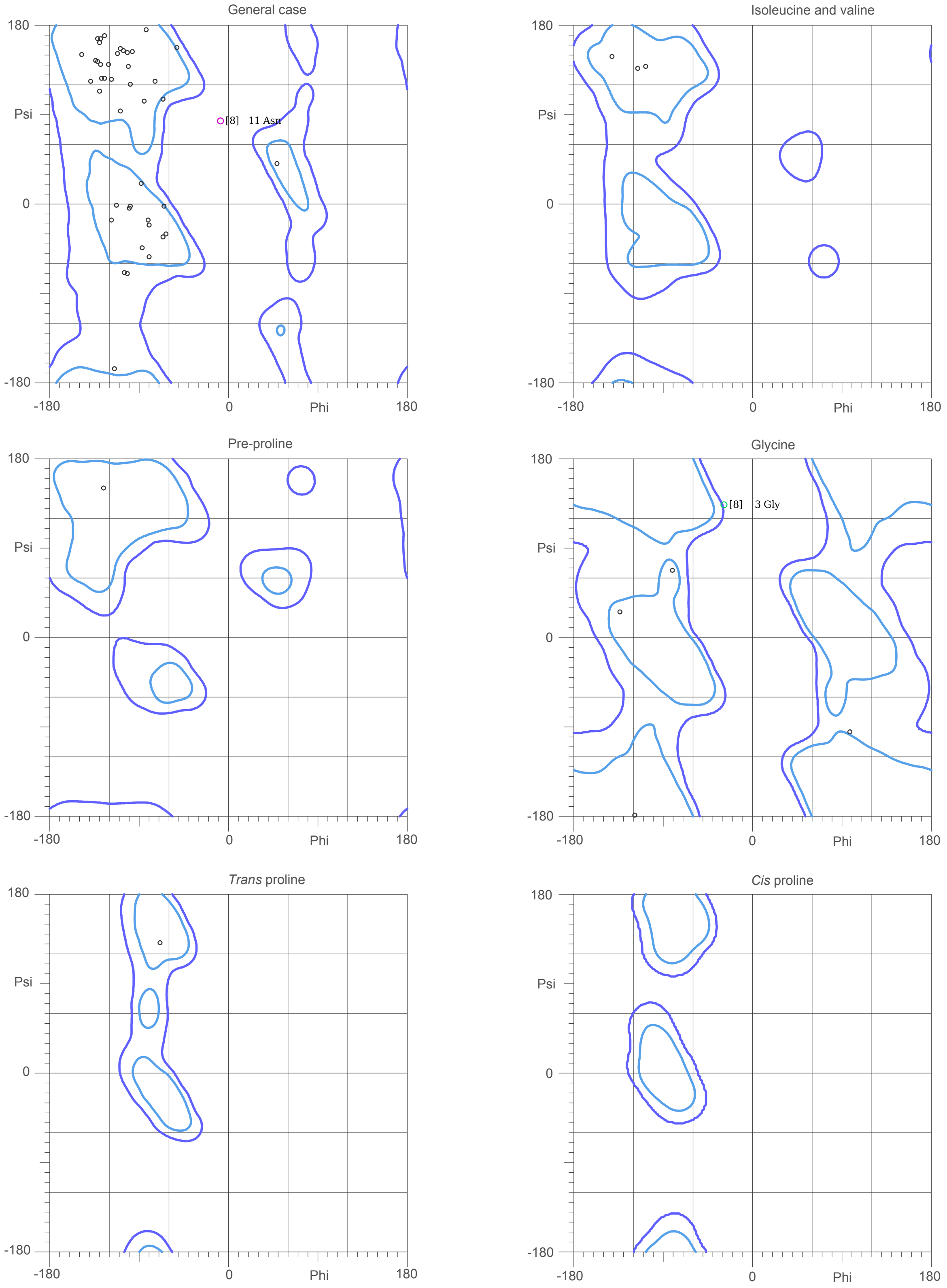

85.2% (46/54) of all residues were in favored (98%) regions.
96.3% (52/54) of all residues were in allowed (>99.8%) regions.

There were 2 outliers (phi, psi):
[8] 3 Gly (-29.8, 134.6)
[8] 11 Asn (-8.5, 84.6)

MolProbity Ramachandran analysis

2nc2H.pdb, model 9

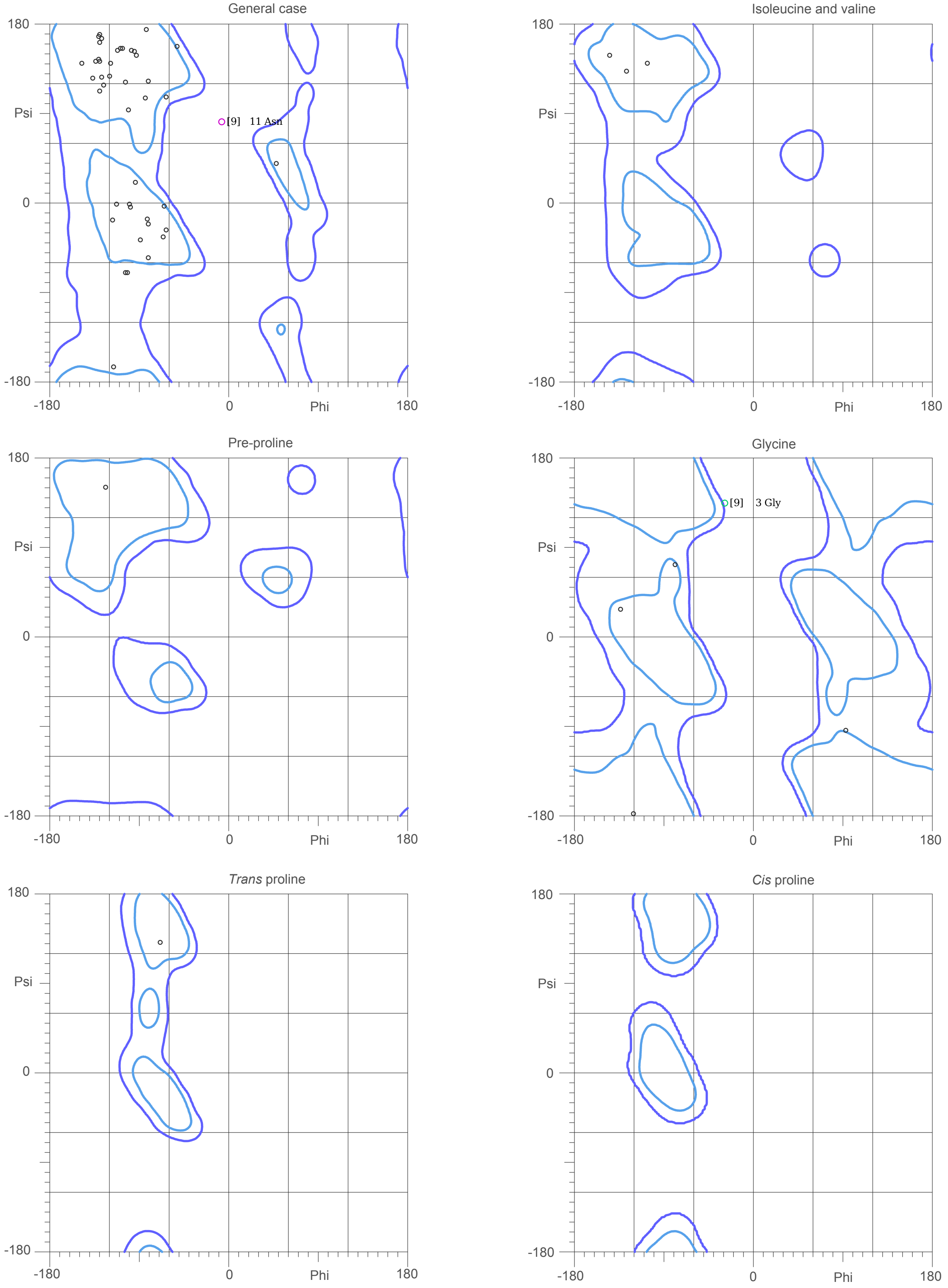

85.2% (46/54) of all residues were in favored (98%) regions.
96.3% (52/54) of all residues were in allowed (>99.8%) regions.

There were 2 outliers (phi, psi):
[9] 3 Gly (-29.4, 135.1)
[9] 11 Asn (-7.8, 83.0)

MolProbity Ramachandran analysis

2nc2H.pdb, model 10

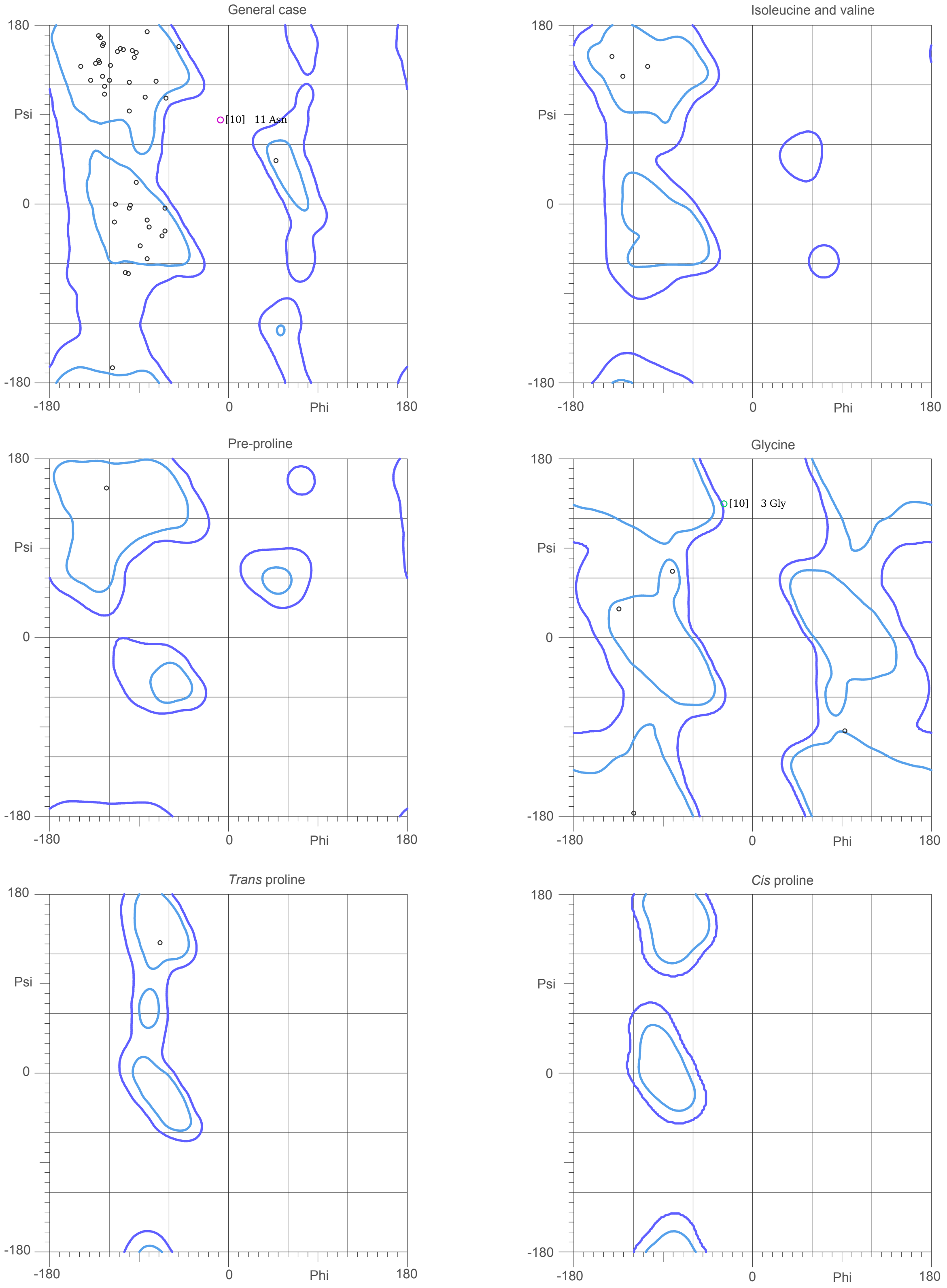

85.2% (46/54) of all residues were in favored (98%) regions.
96.3% (52/54) of all residues were in allowed (>99.8%) regions.

There were 2 outliers (phi, psi):
[10] 3 Gly (-29.2, 135.1)
[10] 11 Asn (-8.0, 85.5)

MolProbity Ramachandran analysis

2nc2H.pdb, model 11

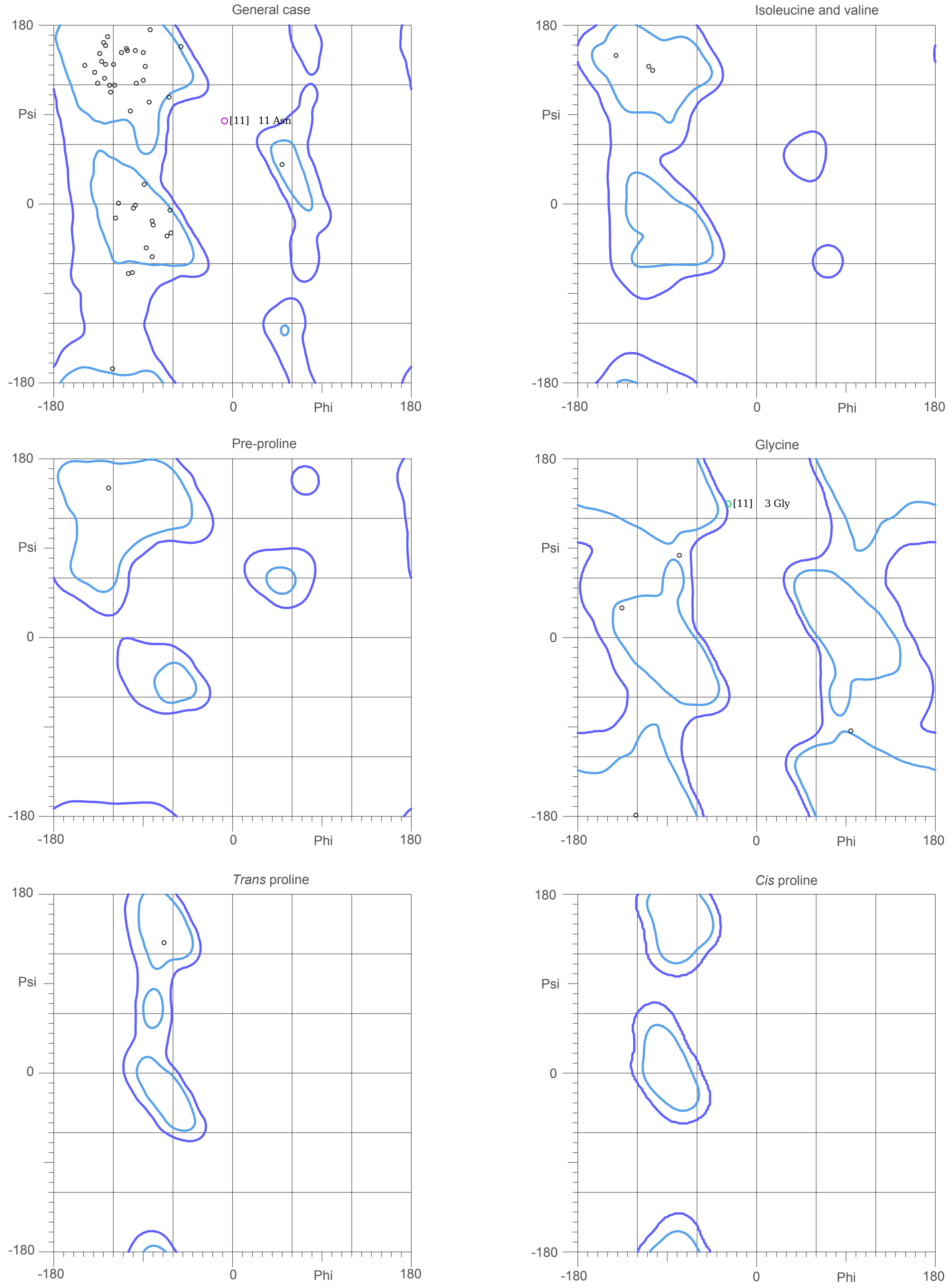

83.3% (45/54) of all residues were in favored (98%) regions.
96.3% (52/54) of all residues were in allowed (>99.8%) regions.

There were 2 outliers (phi, psi):
[11] 3 Gly (-29.4, 135.3)
[11] 11 Asn (-8.3, 84.7)

MolProbity Ramachandran analysis

2nc2H.pdb, model 12

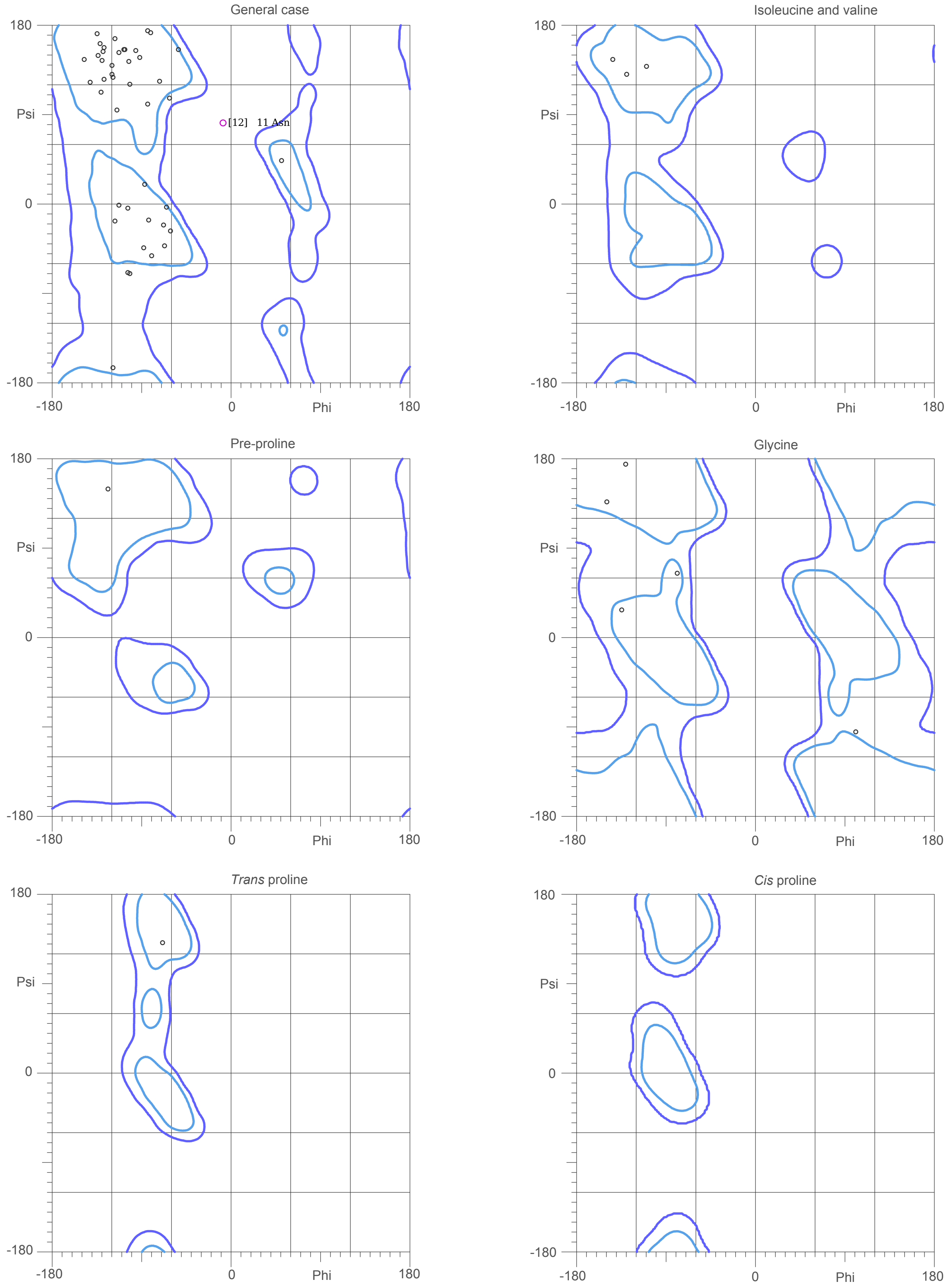

88.9% (48/54) of all residues were in favored (98%) regions.
98.1% (53/54) of all residues were in allowed (>99.8%) regions.

There were 1 outliers (phi, psi):
[12] 11 Asn (-8.6, 83.0)

MolProbity Ramachandran analysis

2nc2H.pdb, model 13

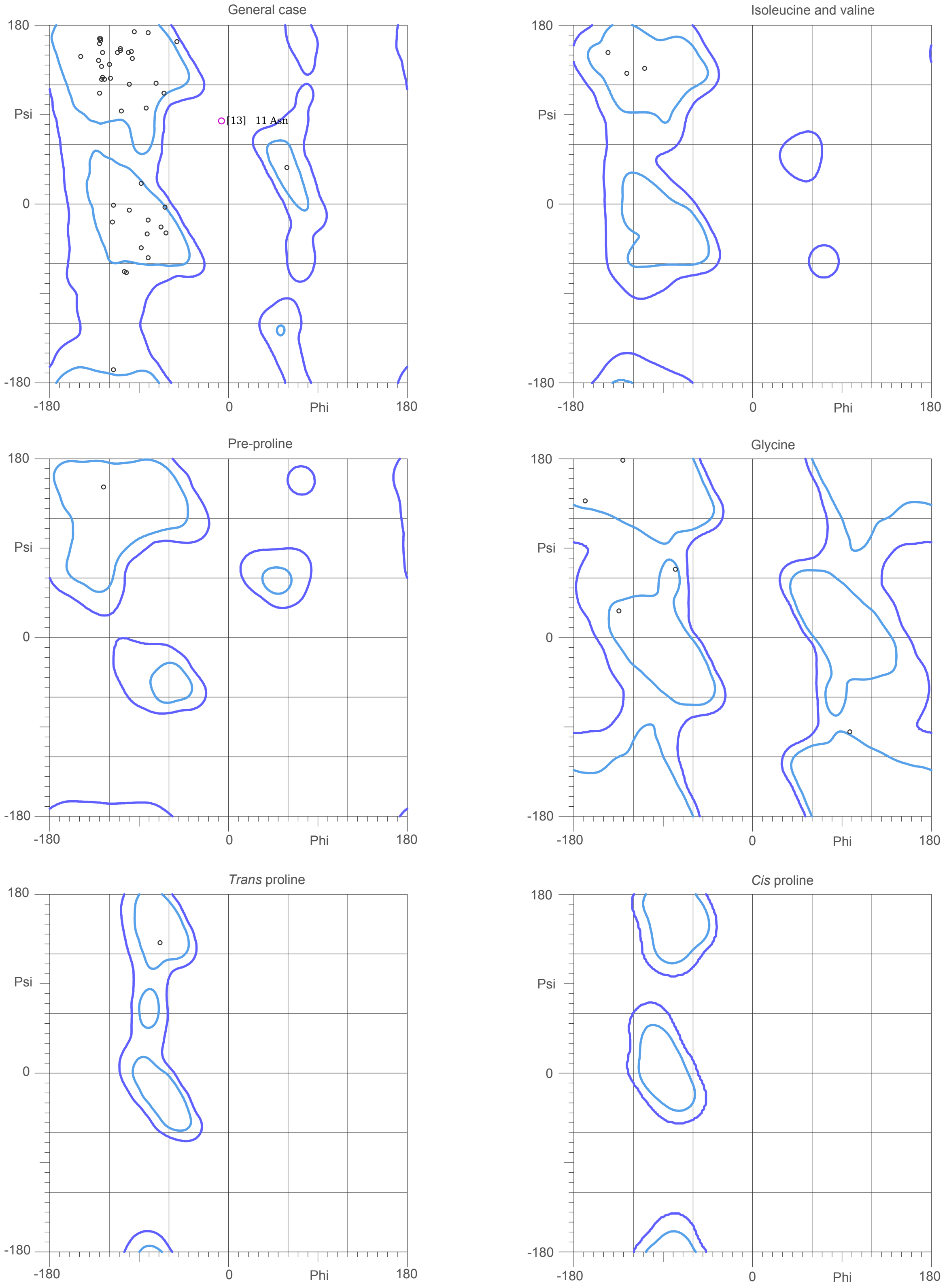

88.9% (48/54) of all residues were in favored (98%) regions.
98.1% (53/54) of all residues were in allowed (>99.8%) regions.

There were 1 outliers (phi, psi):
[13] 11 Asn (-7.7, 84.3)

MolProbity Ramachandran analysis

2nc2H.pdb, model 14

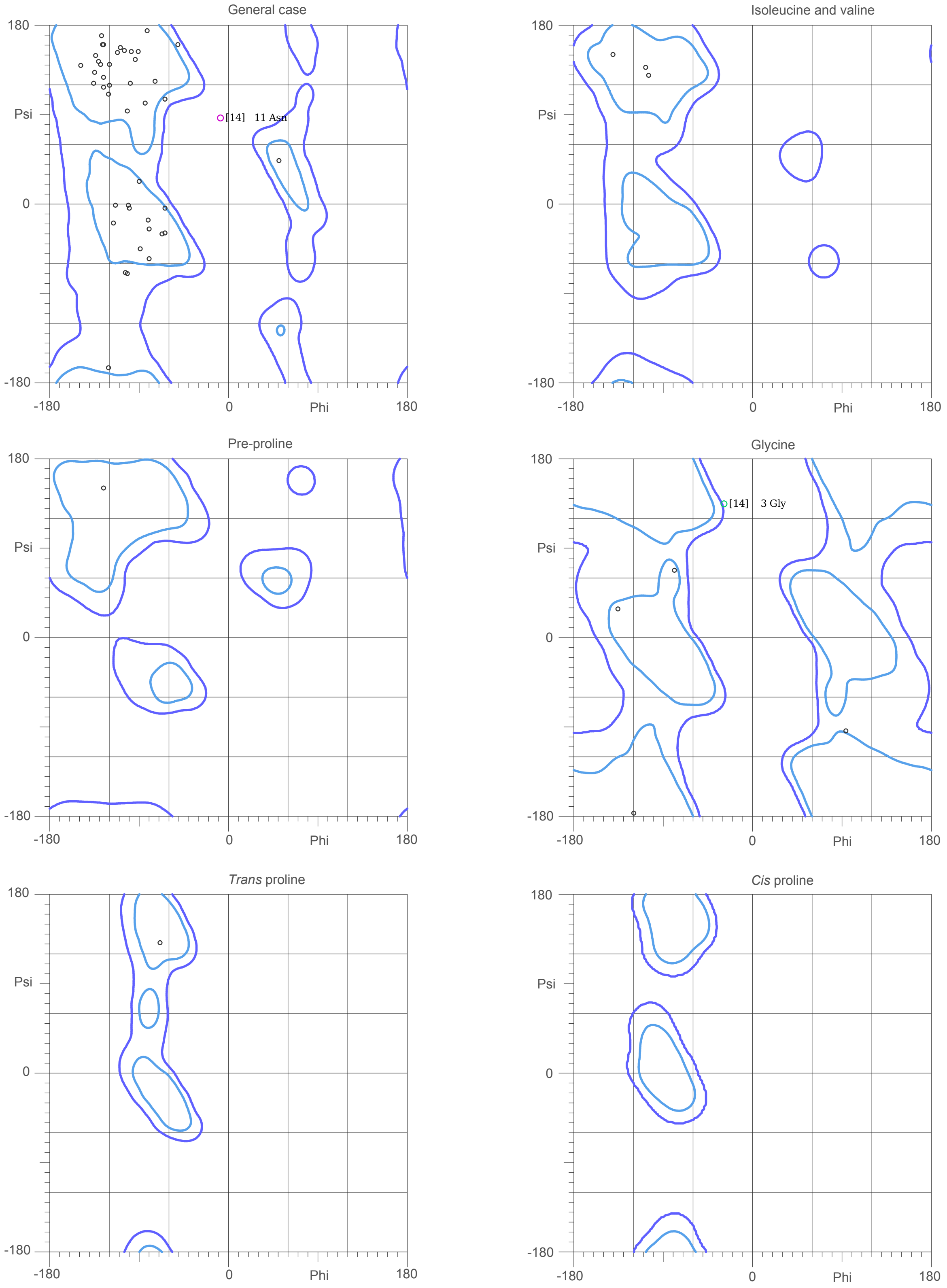

85.2% (46/54) of all residues were in favored (98%) regions.
96.3% (52/54) of all residues were in allowed (>99.8%) regions.

There were 2 outliers (phi, psi):
[14] 3 Gly (-29.6, 135.5)
[14] 11 Asn (-8.9, 87.2)

MolProbity Ramachandran analysis

2nc2H.pdb, model 15

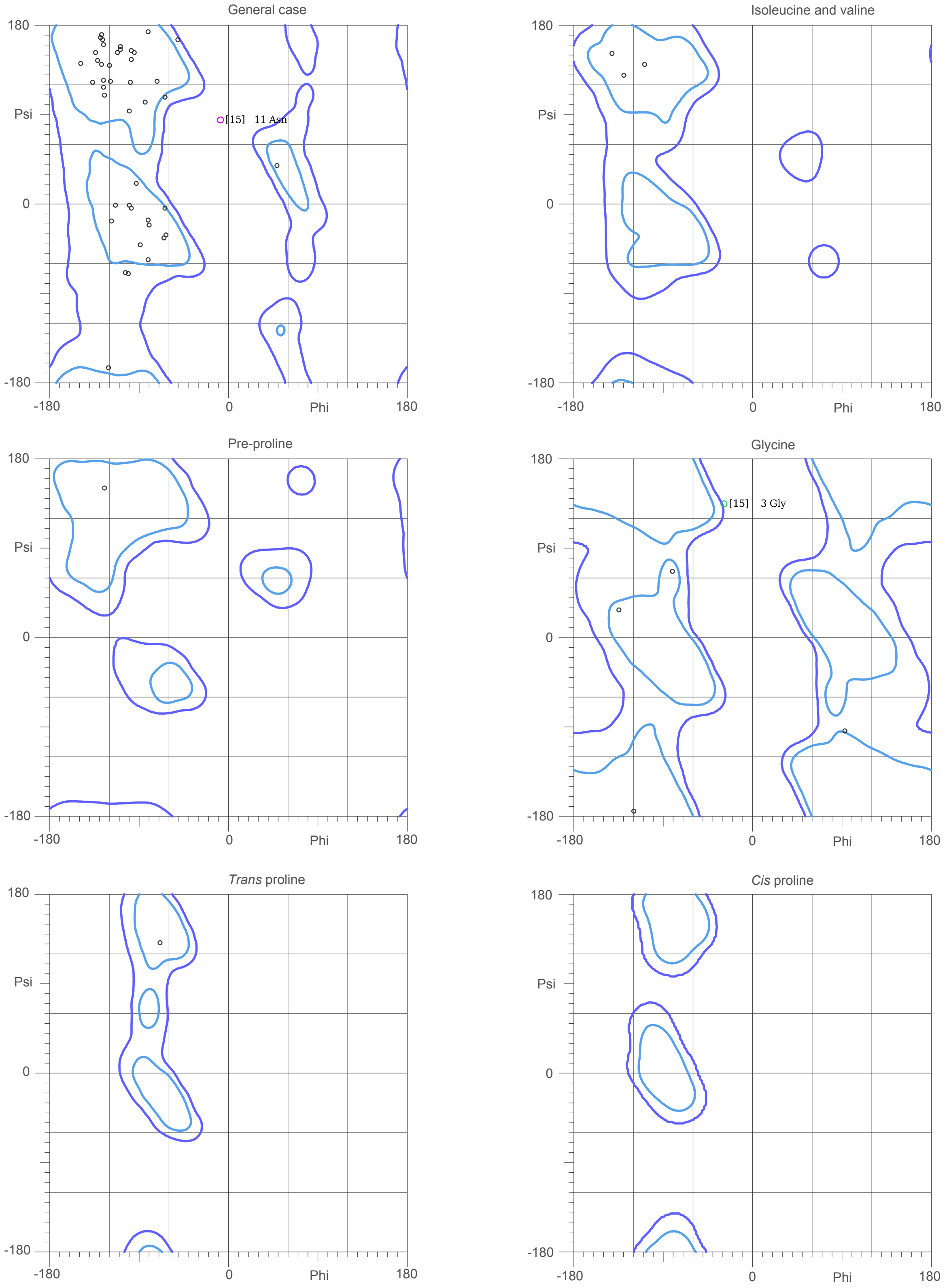

85.2% (46/54) of all residues were in favored (98%) regions.
96.3% (52/54) of all residues were in allowed (>99.8%) regions.

There were 2 outliers (phi, psi):
[15] 3 Gly (-29.3, 135.2)
[15] 11 Asn (-8.3, 85.7)

MolProbity Ramachandran analysis

2nc2H.pdb, model 16

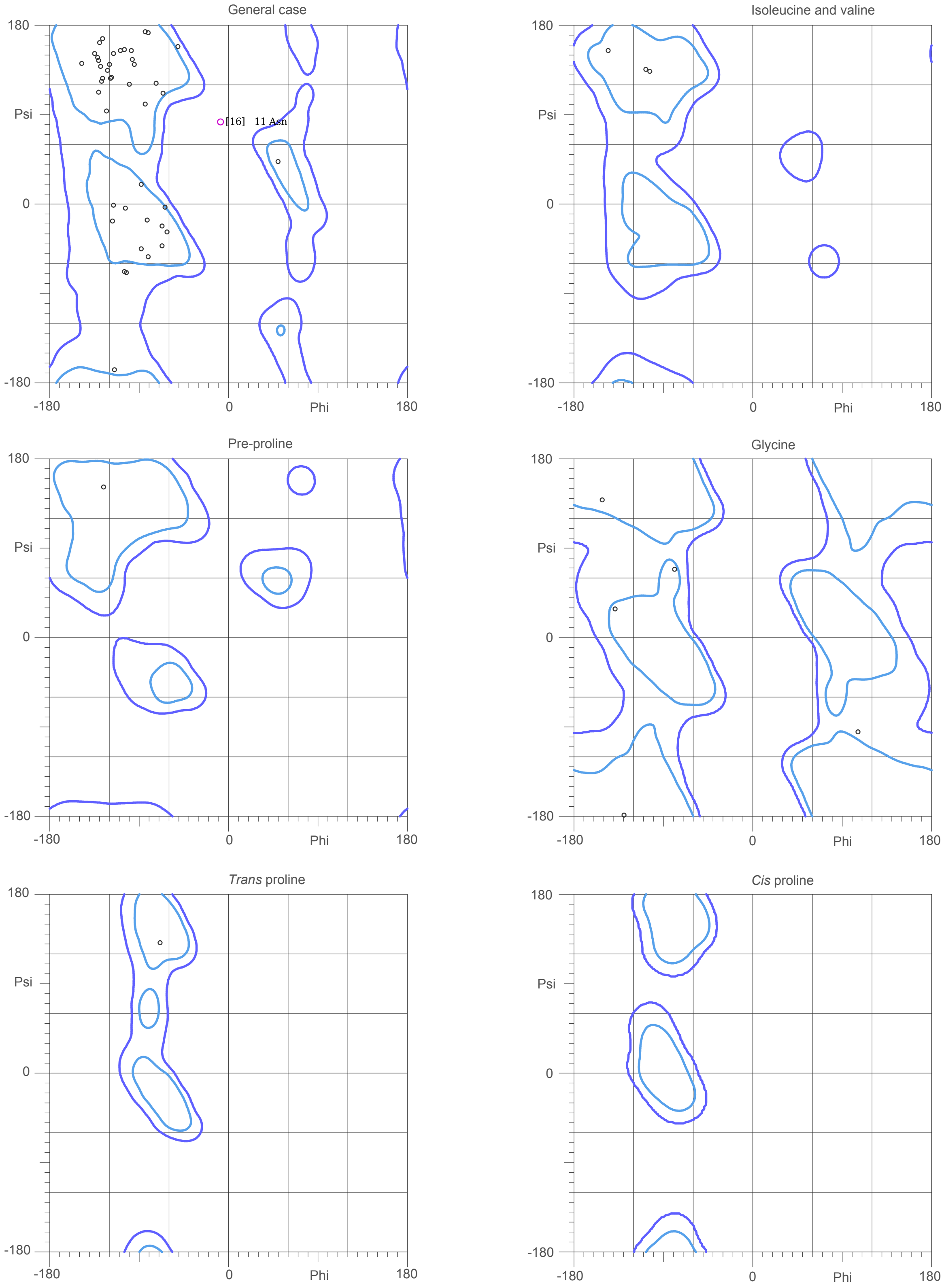

88.9% (48/54) of all residues were in favored (98%) regions.
98.1% (53/54) of all residues were in allowed (>99.8%) regions.

There were 1 outliers (phi, psi):
[16] 11 Asn (-8.6, 83.5)

MolProbity Ramachandran analysis

2nc2H.pdb, model 17

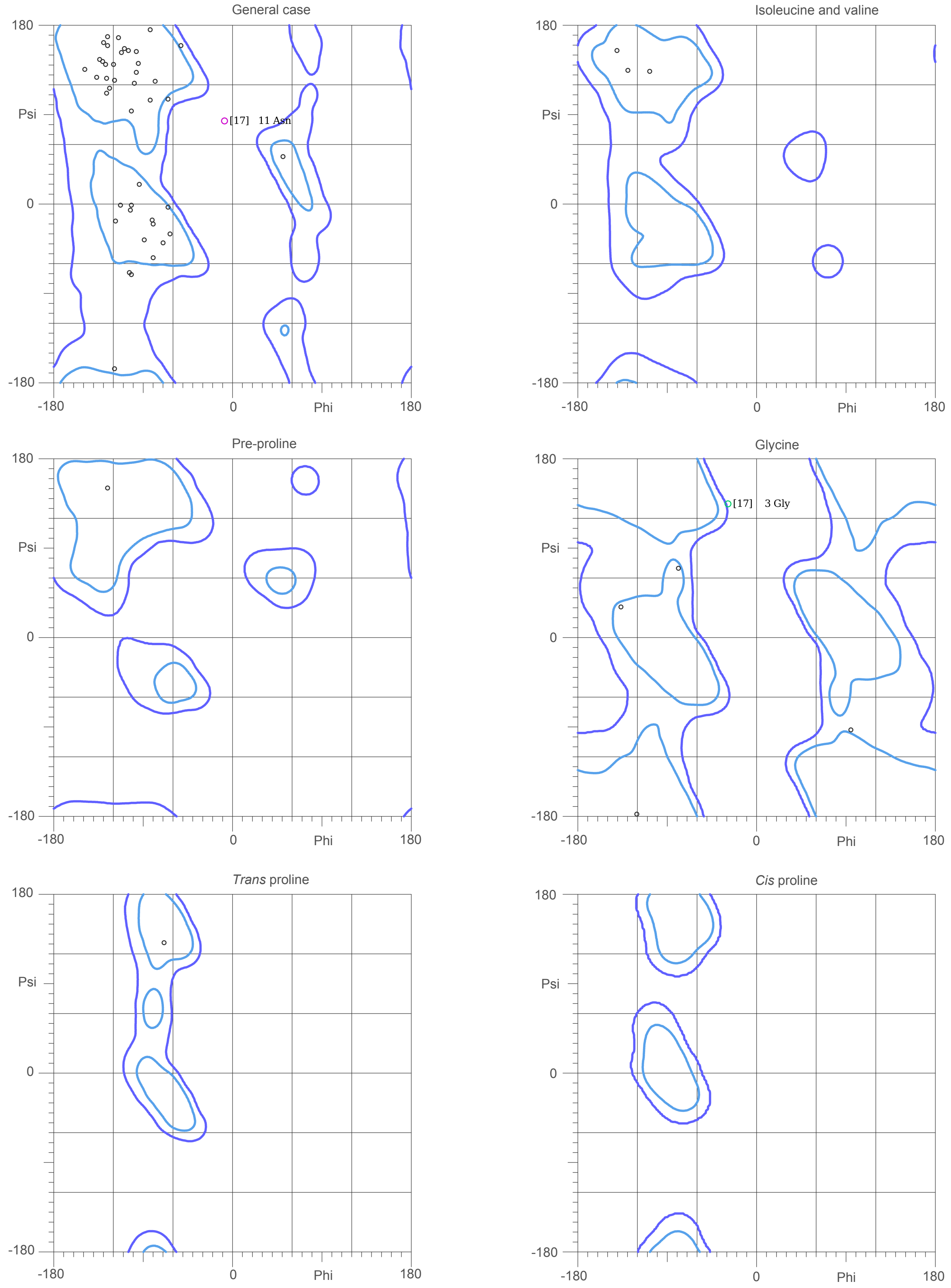

85.2% (46/54) of all residues were in favored (98%) regions.
96.3% (52/54) of all residues were in allowed (>99.8%) regions.

There were 2 outliers (phi, psi):
[17] 3 Gly (-29.2, 135.1)
[17] 11 Asn (-8.4, 84.0)

MolProbity Ramachandran analysis

2nc2H.pdb, model 18

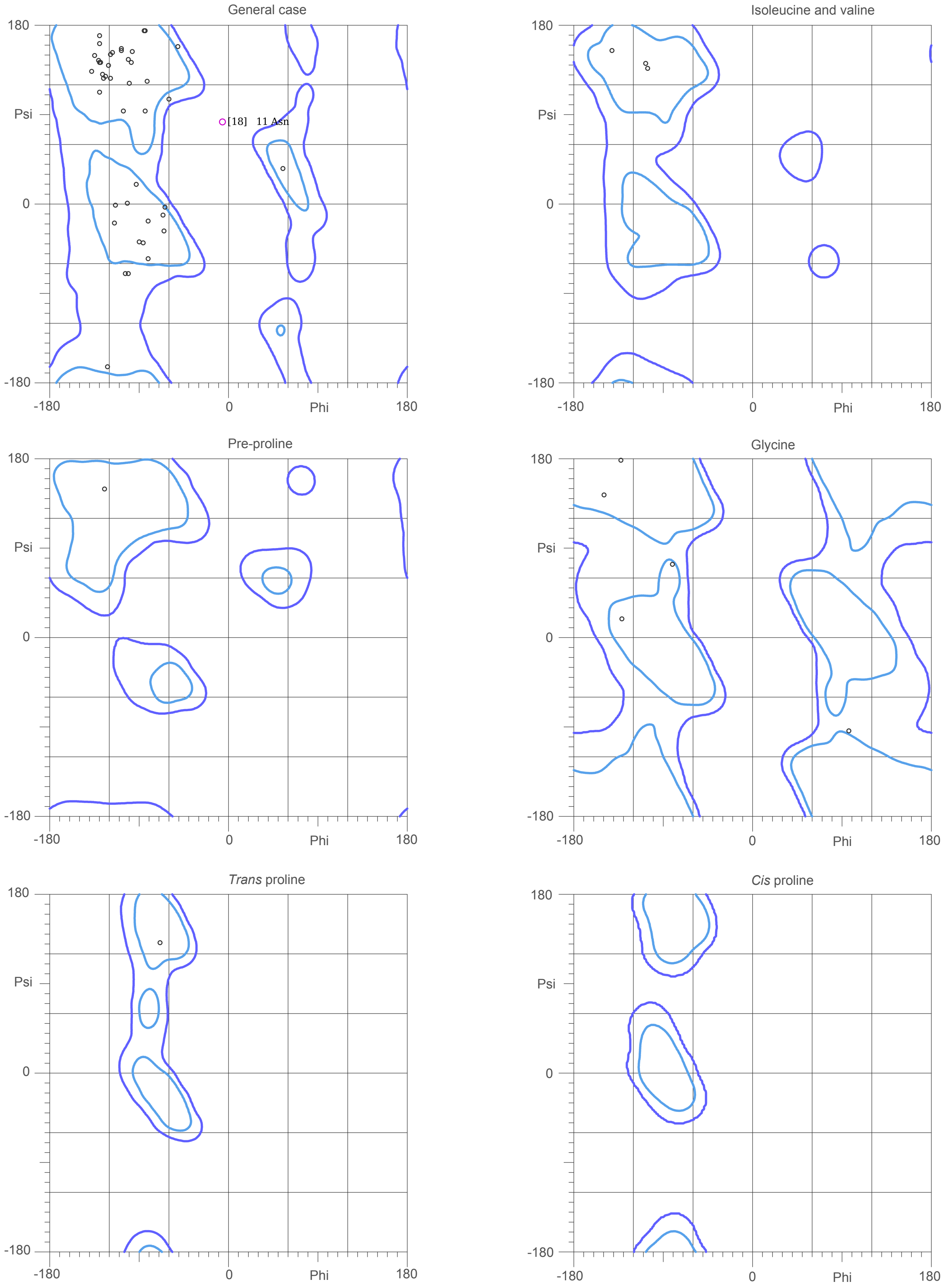

87.0% (47/54) of all residues were in favored (98%) regions.
98.1% (53/54) of all residues were in allowed (>99.8%) regions.

There were 1 outliers (phi, psi):
[18] 11 Asn (-6.9, 83.3)

MolProbity Ramachandran analysis

2nc2H.pdb, model 19

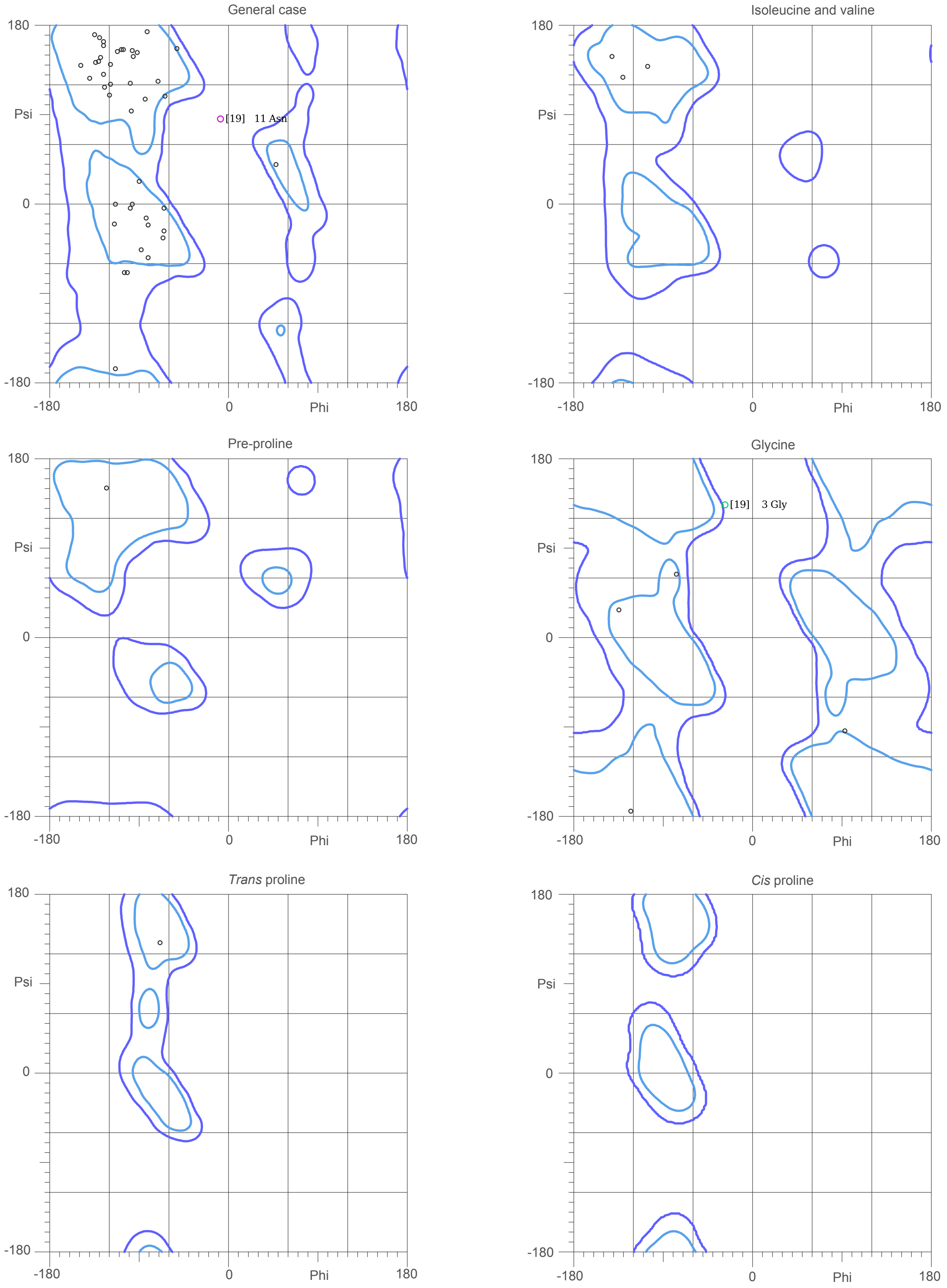

85.2% (46/54) of all residues were in favored (98%) regions.
96.3% (52/54) of all residues were in allowed (>99.8%) regions.

There were 2 outliers (phi, psi):
[19] 3 Gly (-28.6, 134.7)
[19] 11 Asn (-8.4, 86.2)

MolProbity Ramachandran analysis

2nc2H.pdb, model 20

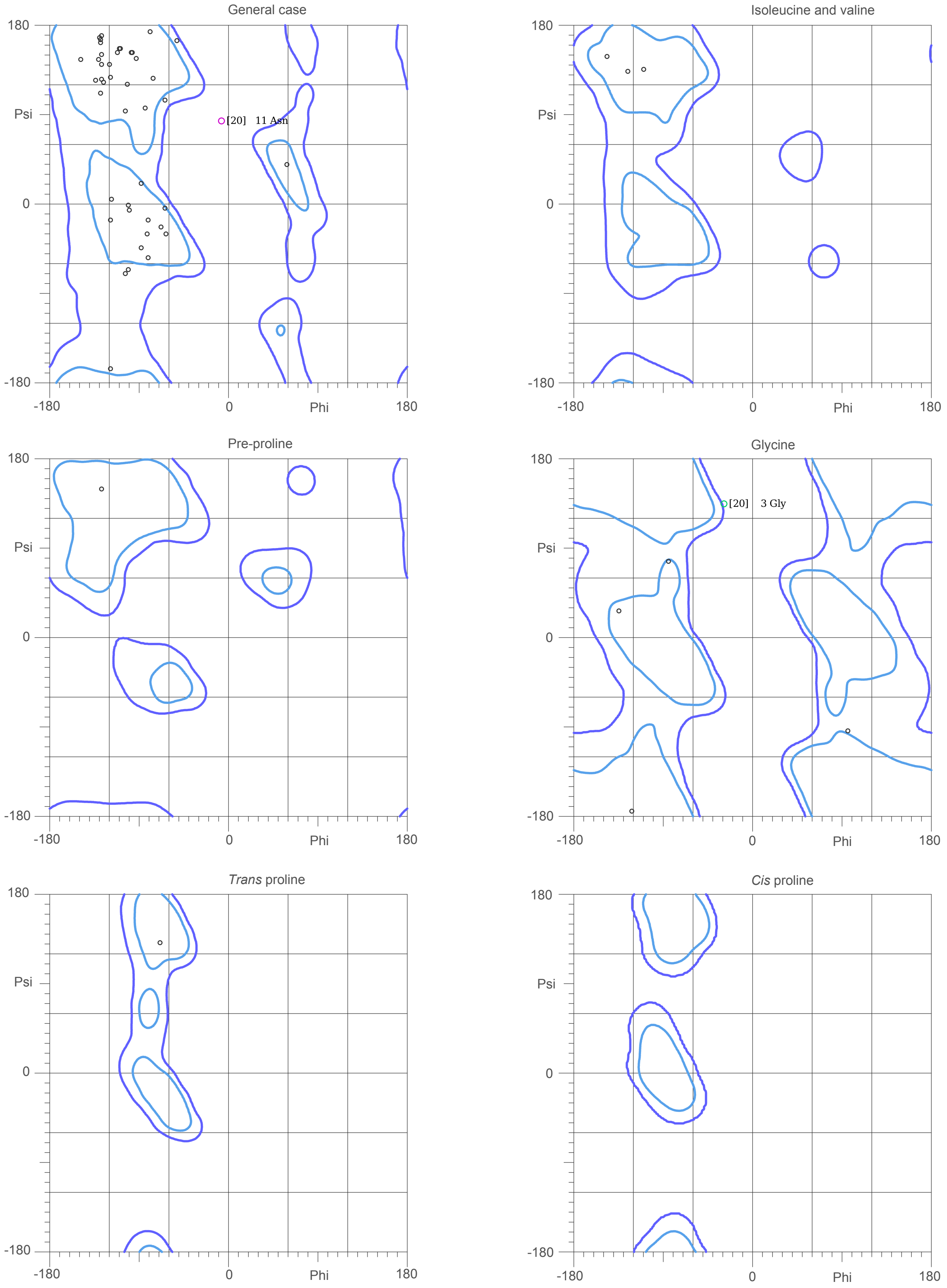

85.2% (46/54) of all residues were in favored (98%) regions.
96.3% (52/54) of all residues were in allowed (>99.8%) regions.

There were 2 outliers (phi, psi):
[20] 3 Gly (-29.5, 135.4)
[20] 11 Asn (-7.2, 84.6)
