# Supplementary material for: Small Disulfide Proteins with Antifungal Impact: NMR Experimental Structures as Compared to Models of Alphafold Versions
Source: Int J Mol Sci. 2025 Jan 31;26(3):1247. doi: 10.3390/ijms26031247 (PMC11818080; doi:10.3390/ijms26031247)
Supplement: Supplementary file 1 [file ijms-26-01247-s001.zip › Figure S7e. AF2-PAFB.pdf]

MolProbity Ramachandran analysis

2NC2-1FH.pdb, model 1

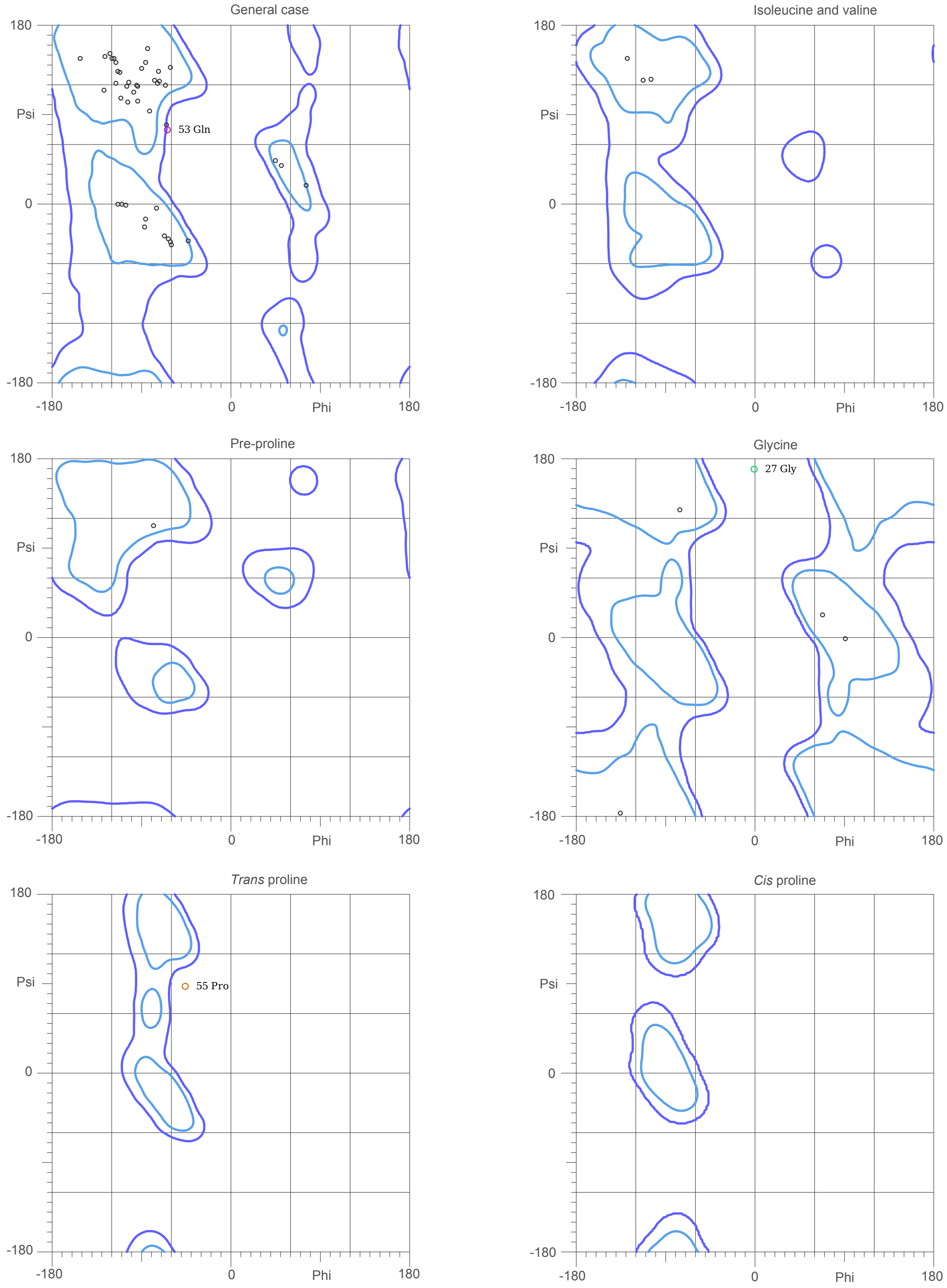

90.7% (49/54) of all residues were in favored (98%) regions.
94.4% (51/54) of all residues were in allowed (>99.8%) regions.

There were 3 outliers (phi, psi):
- 27 Gly (-1.6, 170.0)
- 53 Gln (-64.1, 75.4)
- 55 Pro (-46.9, 88.5)
