# Supplementary material for: Small Disulfide Proteins with Antifungal Impact: NMR Experimental Structures as Compared to Models of Alphafold Versions
Source: Int J Mol Sci. 2025 Jan 31;26(3):1247. doi: 10.3390/ijms26031247 (PMC11818080; doi:10.3390/ijms26031247)
Supplement: Supplementary file 1 [file ijms-26-01247-s001.zip › Figure S7g. NMR-AFPg-1AFP.pdf]

MolProbity Ramachandran analysis

1afp_trimmedH.pdb, all models

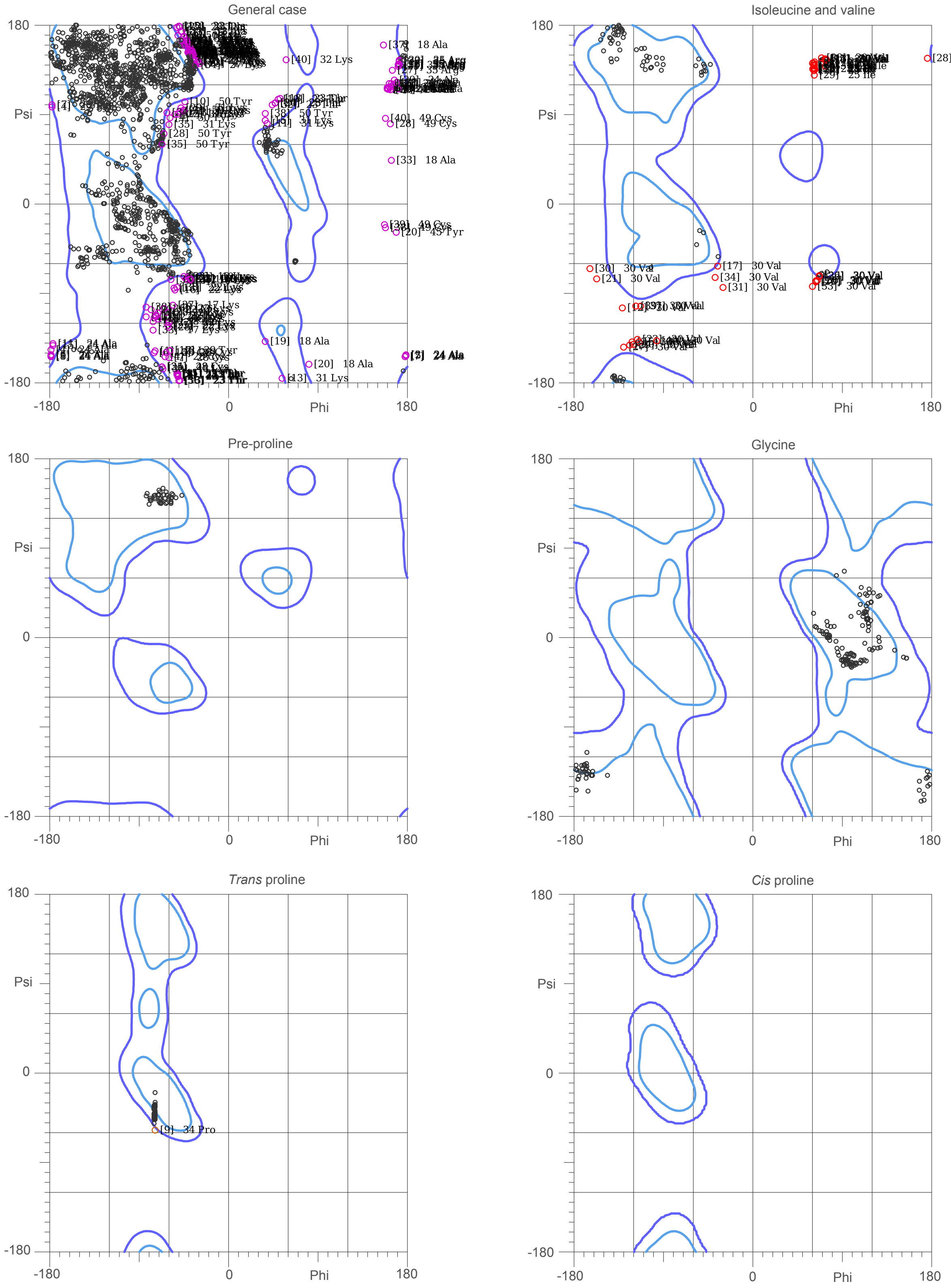

68.5% (1343/1960) of all residues were in favored (98%) regions.
89.1% (1747/1960) of all residues were in allowed (>99.8%) regions.
This list is truncated; use the MolProbity multi-chart.html for complete list.
There were 213 outliers (phi, psi):

[1] 23 Thr (-51.3, -173.0)
[1] 24 Ala (-179.1, -153.8)
[1] 30 Val (74.5, 146.9)
[1] 32 Lys (-40.7, 160.2)
[1] 48 Lys (-74.2, -148.9)
[1] 50 Tyr (-45.9, 95.1)
[2] 23 Thr (-51.6, -174.1)
[2] 24 Ala (179.3, -152.0)
[2] 27 Lys (-37.8, 155.7)
[2] 30 Val (-122.3, -139.4)
[2] 32 Lys (-39.8, 154.2)
[3] 10 Lys (-38.7, -77.2)
[3] 18 Ala (-45.9, 169.7)
[3] 24 Ala (161.4, 117.0)
[3] 25 Ile (60.6, 142.4)
[3] 32 Lys (-39.8, 157.2)
[4] 23 Thr (-51.7, -172.9)
[4] 24 Ala (-179.3, -147.8)
[4] 28 Cys (-65.0, -154.1)
[4] 29 Tyr (-178.2, 98.3)
[4] 50 Tyr (-59.1, 87.5)
[5] 10 Lys (-42.2, -76.2)
[5] 23 Thr (-52.3, -172.5)
[5] 24 Ala (179.1, -153.8)
[5] 27 Lys (-36.7, 154.7)
[5] 30 Val (-115.3, -140.0)
[5] 32 Lys (-41.5, 154.0)
[5] 35 Arg (172.5, 140.8)
[6] 23 Thr (-51.8, -173.1)
[6] 24 Ala (-179.6, -152.2)
[6] 27 Lys (-35.6, 150.9)
[6] 28 Cys (-75.2, -150.8)
[6] 32 Lys (-44.0, 167.1)
[6] 50 Tyr (-51.3, 170.6)
[7] 23 Thr (-51.6, -173.8)
[7] 24 Ala (178.7, -154.7)
[7] 27 Lys (-36.5, 147.6)
[7] 28 Cys (-60.5, -155.7)
[7] 29 Tyr (-178.2, 100.5)
[7] 32 Lys (-40.5, 153.8)
[8] 23 Thr (-51.8, -171.6)
[8] 24 Ala (-179.7, -154.5)
[8] 27 Lys (-33.3, 146.9)
[8] 32 Lys (-39.4, 154.3)
[9] 18 Ala (-45.5, 169.3)
[9] 24 Ala (160.6, 116.9)
[9] 25 Ile (60.8, 136.2)
[9] 32 Lys (-39.1, 156.4)
[9] 34 Pro (-75.0, -57.4)
[10] 22 Lys (-75.9, -115.2)
[10] 28 Cys (-63.6, -150.3)
[10] 32 Lys (-40.8, 153.5)
[10] 50 Tyr (-44.6, 103.0)
[11] 23 Thr (-51.6, -172.4)
[11] 24 Ala (-177.0, -141.7)
[11] 27 Lys (-37.0, 154.9)
[11] 30 Val (73.7, 146.7)
[11] 31 Lys (39.8, 81.6)
[11] 32 Lys (-43.0, 164.2)
[12] 22 Lys (-83.6, -114.6)
[12] 30 Val (-132.1, -106.0)
[12] 32 Lys (-40.1, 154.1)
[13] 32 Lys (-55.8, -85.6)
[13] 23 Thr (-49.9, -178.8)

MolProbity Ramachandran analysis

1afp_trimmedH.pdb, model 1

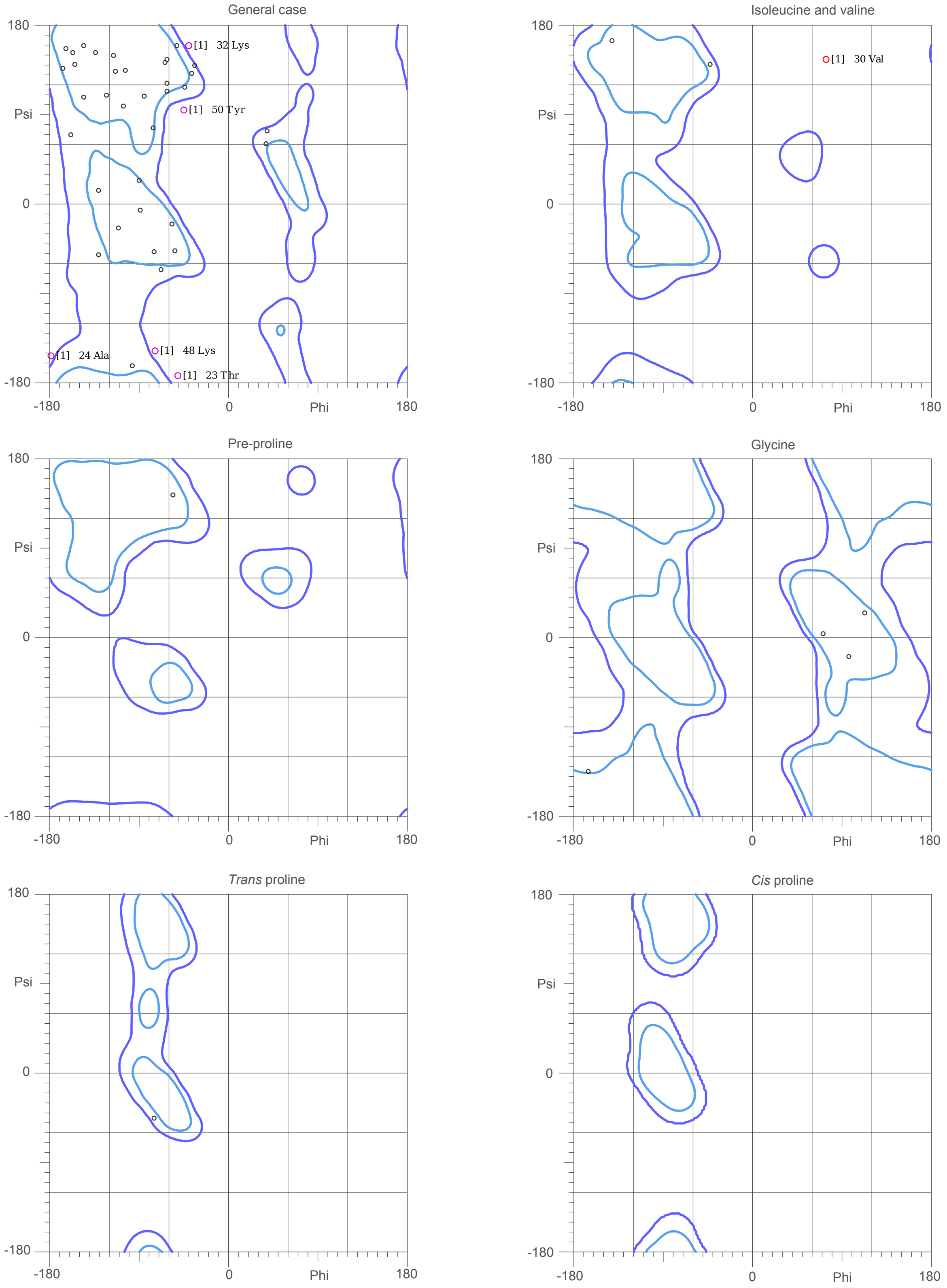

61.2% (30/49) of all residues were in favored (98%) regions.
87.8% (43/49) of all residues were in allowed (>99.8%) regions.

There were 6 outliers (phi, psi):

[1] 23 Thr (-51.3, -173.0)
[1] 24 Ala (-179.1, -153.8)
[1] 30 Val (74.5, 146.9)
[1] 32 Lys (-40.7, 160.2)
[1] 48 Lys (-74.2, -148.9)
[1] 50 Tyr (-45.9, 95.1)

MolProbity Ramachandran analysis

1afp_trimmedH.pdb, model 2

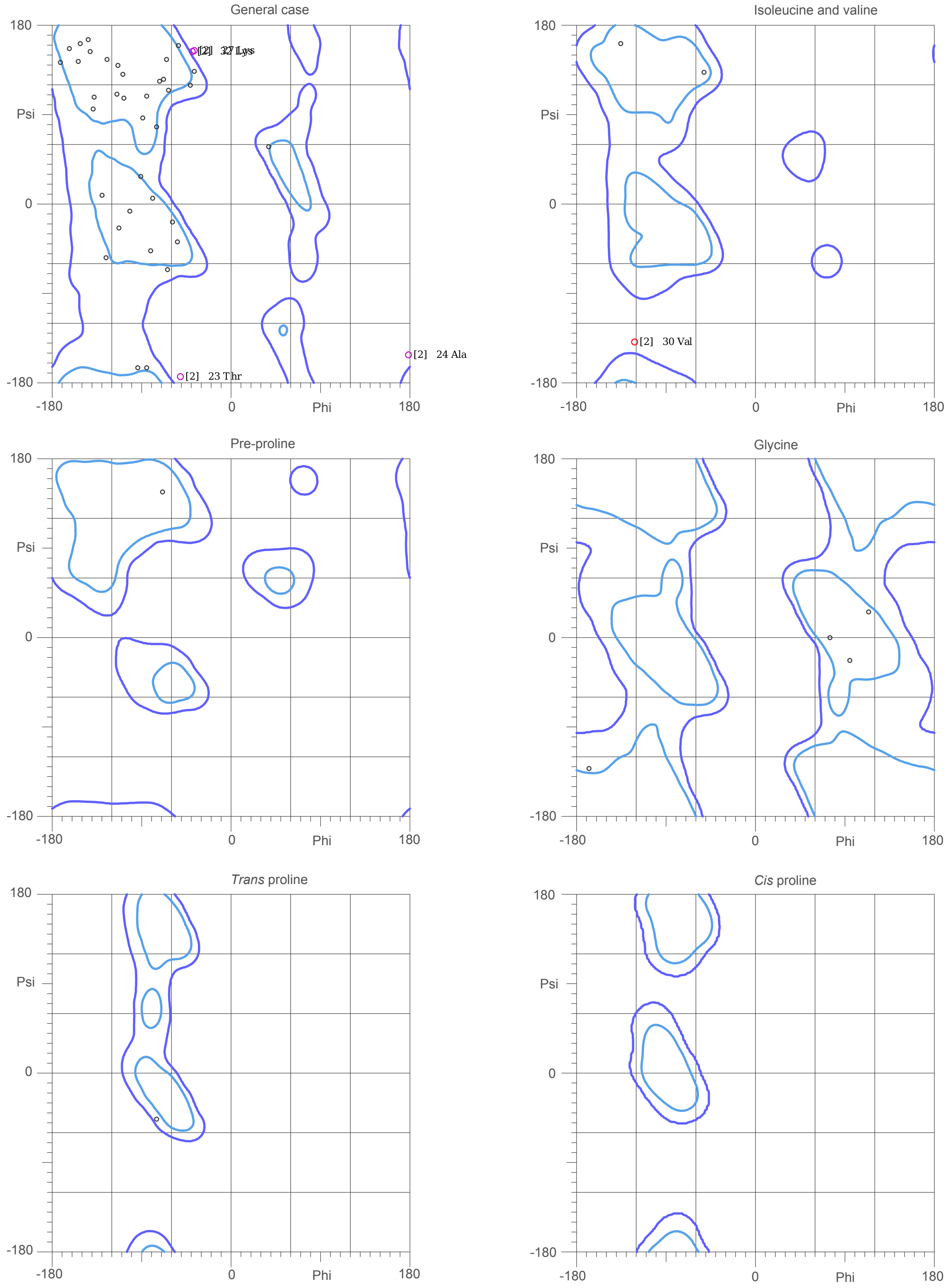

65.3% (32/49) of all residues were in favored (98%) regions.
89.8% (44/49) of all residues were in allowed (>99.8%) regions.

There were 5 outliers (phi, psi):
[2] 23 Thr (-51.6, -174.1)
[2] 24 Ala (179.3, -152.0)
[2] 27 Lys (-37.8, 155.7)
[2] 30 Val (-122.3, -139.4)
[2] 32 Lys (-39.8, 154.2)

MolProbity Ramachandran analysis

1afp_trimmedH.pdb, model 3

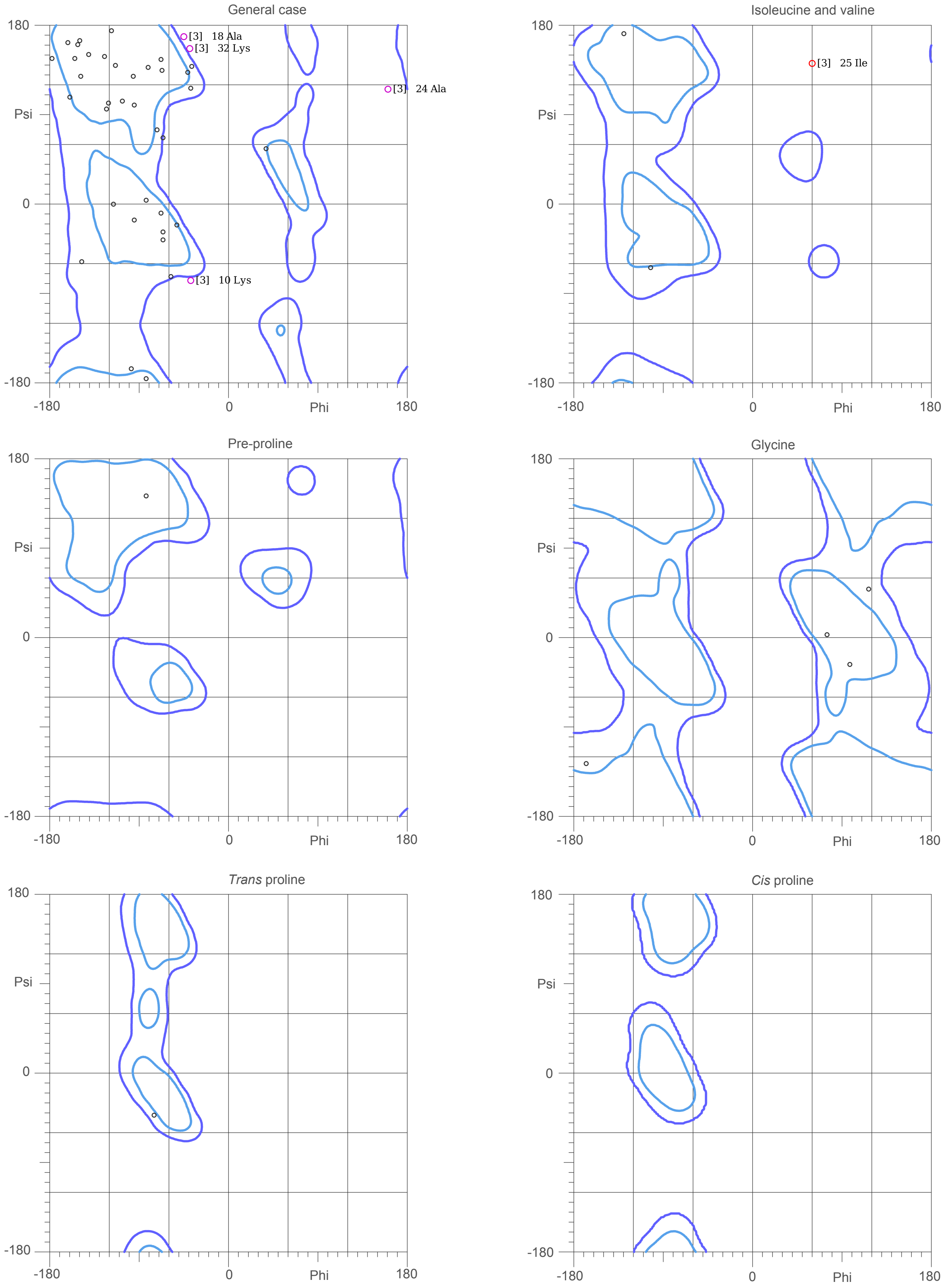

61.2% (30/49) of all residues were in favored (98%) regions.
89.8% (44/49) of all residues were in allowed (>99.8%) regions.

There were 5 outliers (phi, psi):
[3] 10 Lys (-38.7, -77.2)
[3] 18 Ala (-45.9, 169.7)
[3] 24 Ala (161.4, 117.0)
[3] 25 Ile (60.6, 142.4)
[3] 32 Lys (-39.8, 157.2)

Lovell, Davis, et al. Proteins 50:437 (2003)

MolProbity Ramachandran analysis

1afp_trimmedH.pdb, model 4

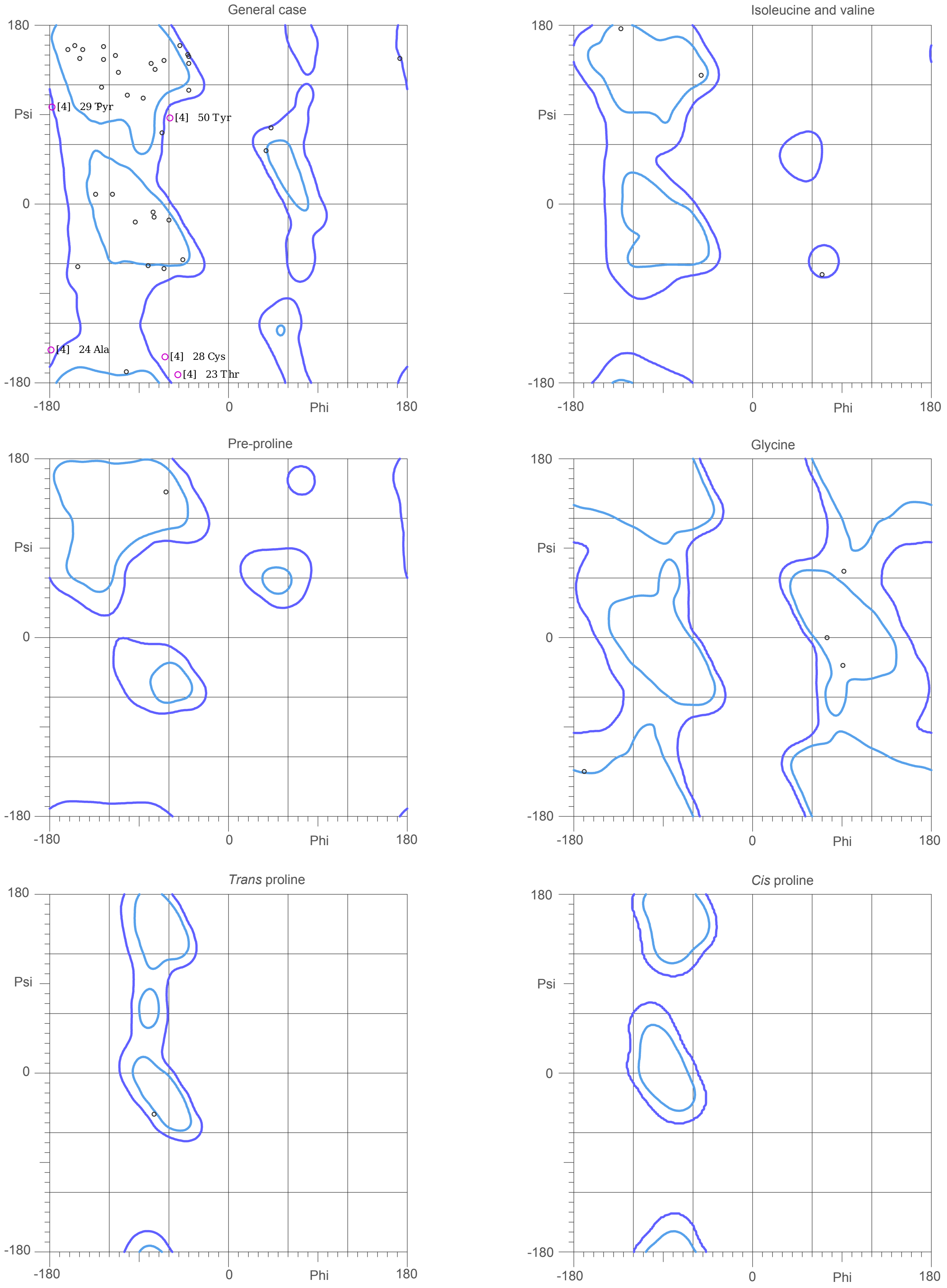

55.1% (27/49) of all residues were in favored (98%) regions.
89.8% (44/49) of all residues were in allowed (>99.8%) regions.

There were 5 outliers (phi, psi):
- [4] 23 Thr (-51.7, -172.9)
- [4] 24 Ala (-179.3, -147.8)
- [4] 28 Cys (-65.0, -154.1)
- [4] 29 Tyr (-178.2, 98.3)
- [4] 50 Tyr (-59.1, 87.5)

MolProbity Ramachandran analysis

1afp_trimmedH.pdb, model 5

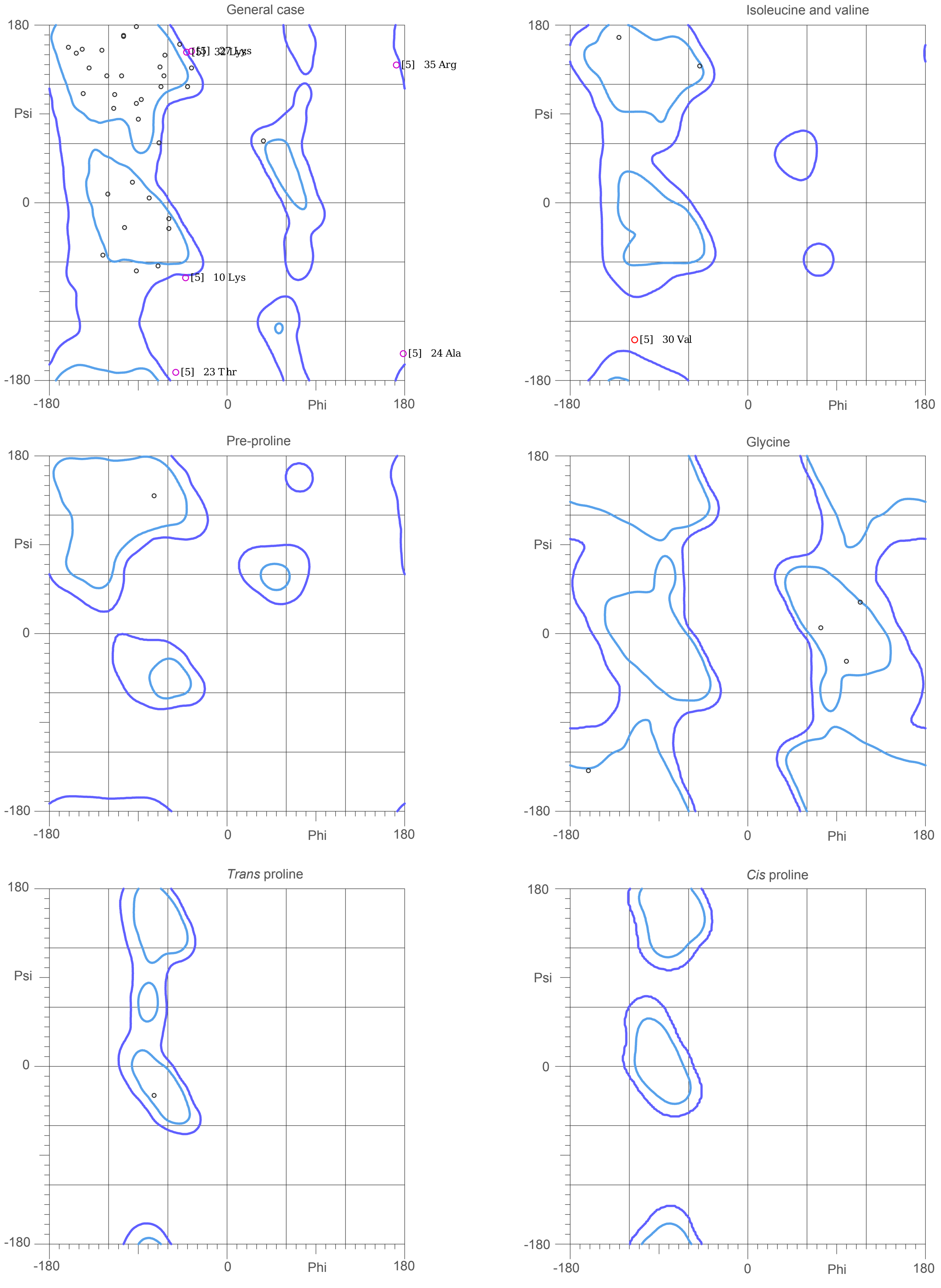

67.3% (33/49) of all residues were in favored (98%) regions.
85.7% (42/49) of all residues were in allowed (>99.8%) regions.

There were 7 outliers (phi, psi):
[5] 10 Lys (-42.2, -76.2)
[5] 23 Thr (-52.3, -172.5)
[5] 24 Ala (179.1, -153.8)
[5] 27 Lys (-36.7, 154.7)
[5] 30 Val (-115.3, -140.0)
[5] 32 Lys (-41.5, 154.0)
[5] 35 Arg (172.5, 140.8)

MolProbity Ramachandran analysis

1afp_trimmedH.pdb, model 6

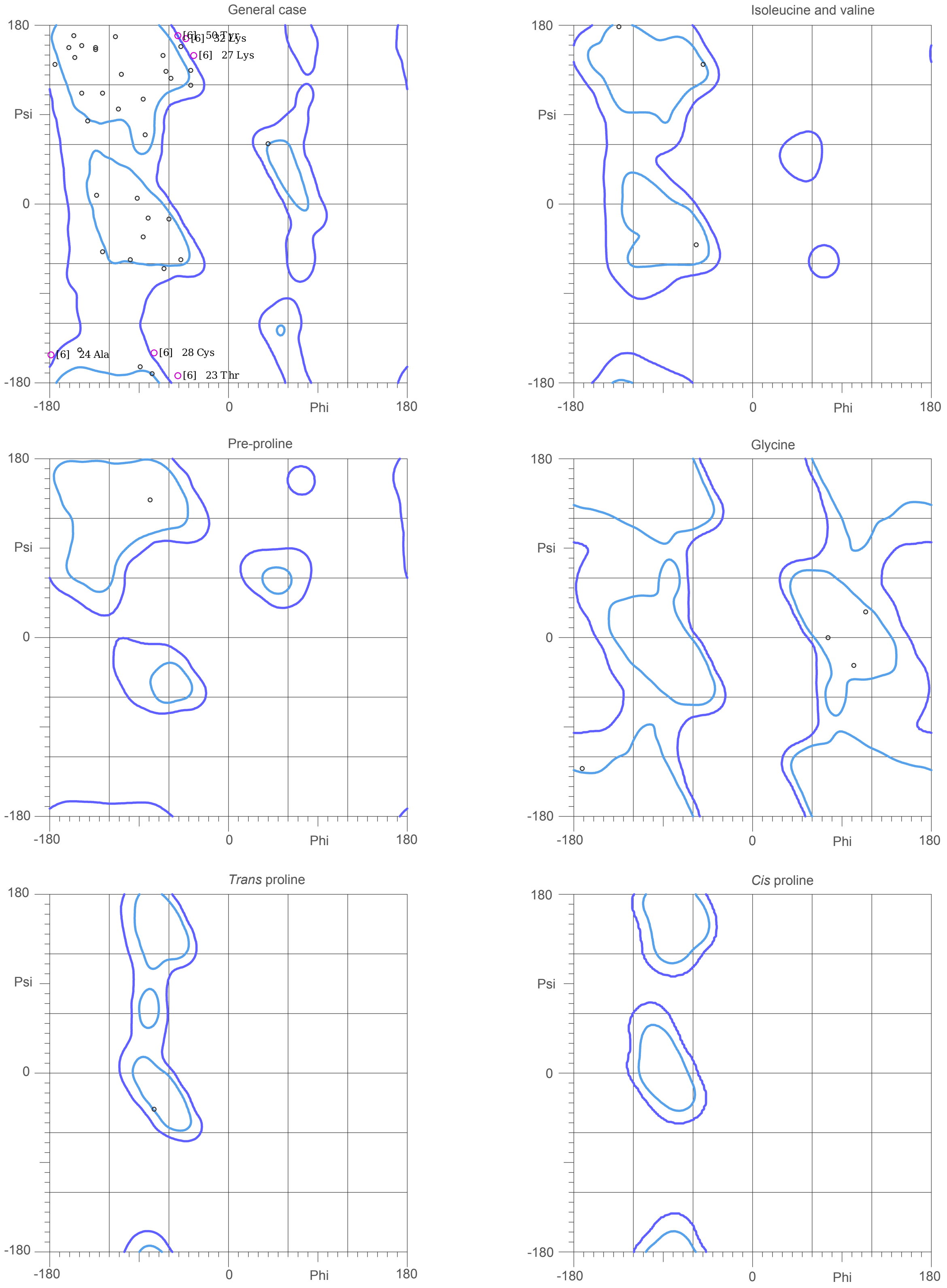

63.3% (31/49) of all residues were in favored (98%) regions.
87.8% (43/49) of all residues were in allowed (>99.8%) regions.

There were 6 outliers (phi, psi):
[6] 23 Thr (-51.8, -173.1)
[6] 24 Ala (-179.6, -152.2)
[6] 27 Lys (-35.6, 150.9)
[6] 28 Cys (-75.2, -150.8)
[6] 32 Lys (-44.0, 167.1)
[6] 50 Tyr (-51.3, 170.6)

MolProbity Ramachandran analysis

1afp_trimmedH.pdb, model 7

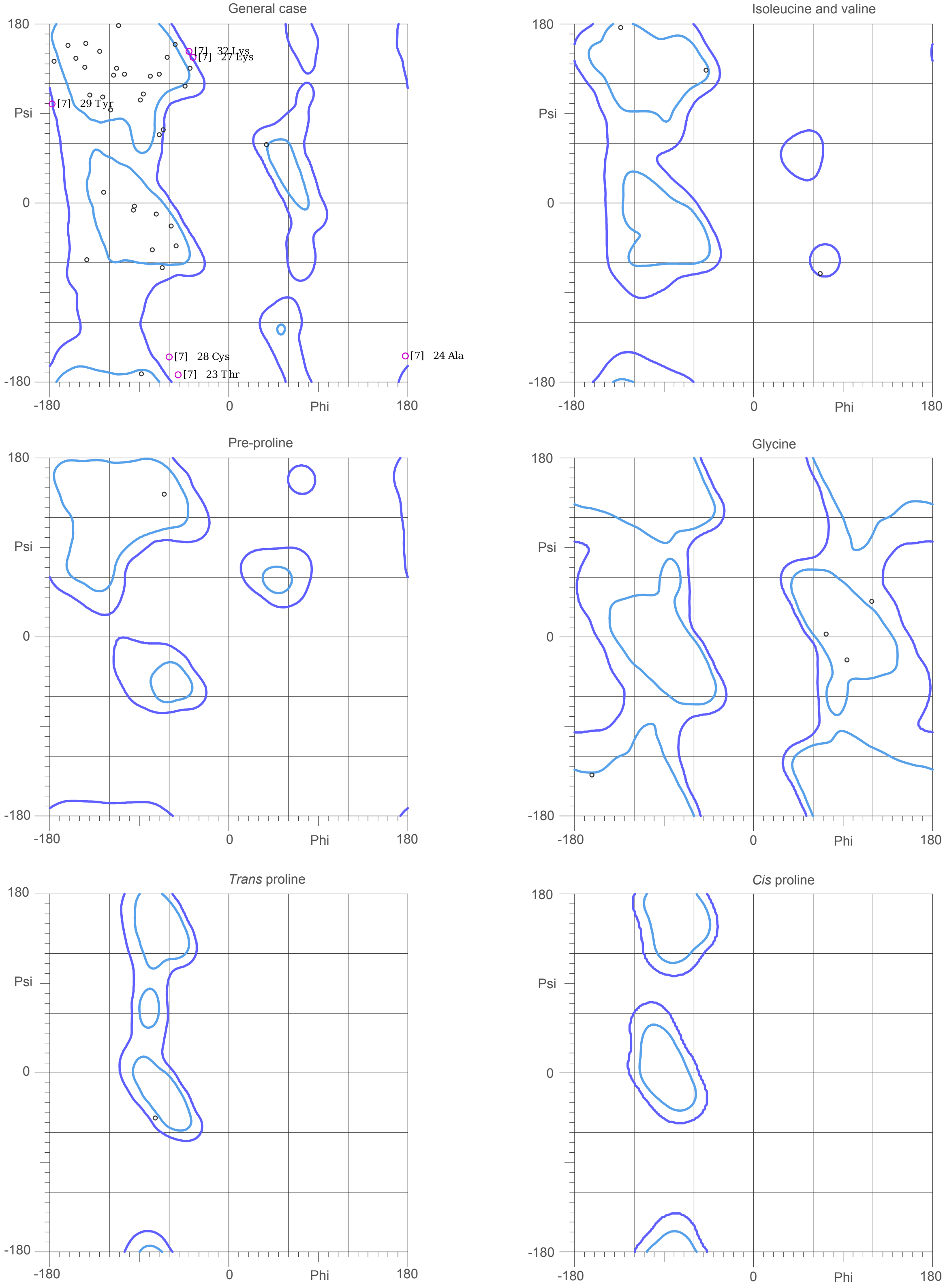

63.3% (31/49) of all residues were in favored (98%) regions.
87.8% (43/49) of all residues were in allowed (>99.8%) regions.

There were 6 outliers (phi, psi):

- [7] 23 Thr (-51.6, -173.8)
- [7] 24 Ala (178.7, -154.7)
- [7] 27 Lys (-36.5, 147.6)
- [7] 28 Cys (-60.5, -155.7)
- [7] 29 Tyr (-178.2, 100.5)
- [7] 32 Lys (-40.5, 153.8)

MolProbity Ramachandran analysis

1afp_trimmedH.pdb, model 8

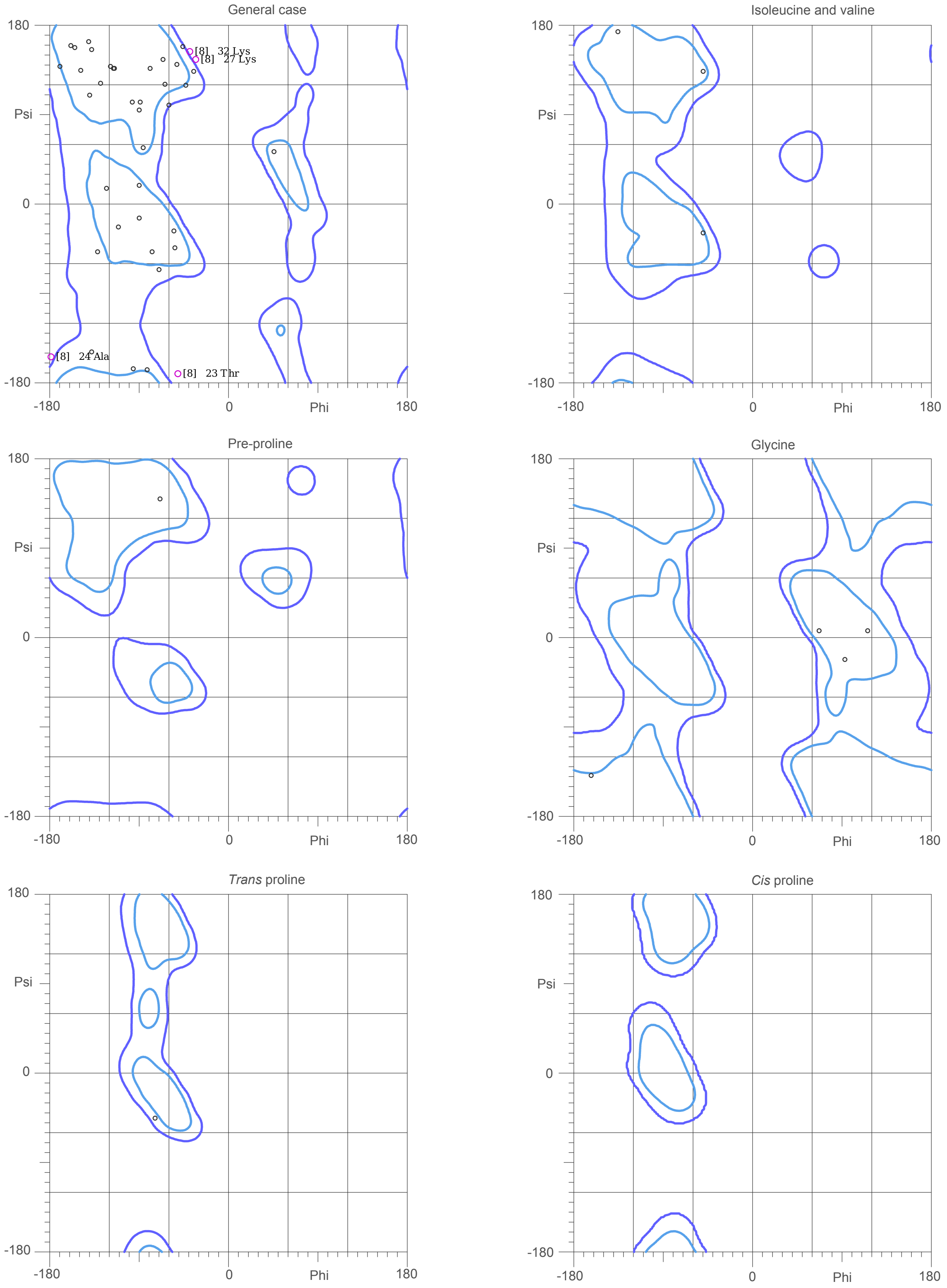

69.4% (34/49) of all residues were in favored (98%) regions.
91.8% (45/49) of all residues were in allowed (>99.8%) regions.

There were 4 outliers (phi, psi):
[8] 23 Thr (-51.8, -171.6)
[8] 24 Ala (-179.7, -154.5)
[8] 27 Lys (-33.3, 146.9)
[8] 32 Lys (-39.4, 154.3)

MolProbity Ramachandran analysis

1afp_trimmedH.pdb, model 9

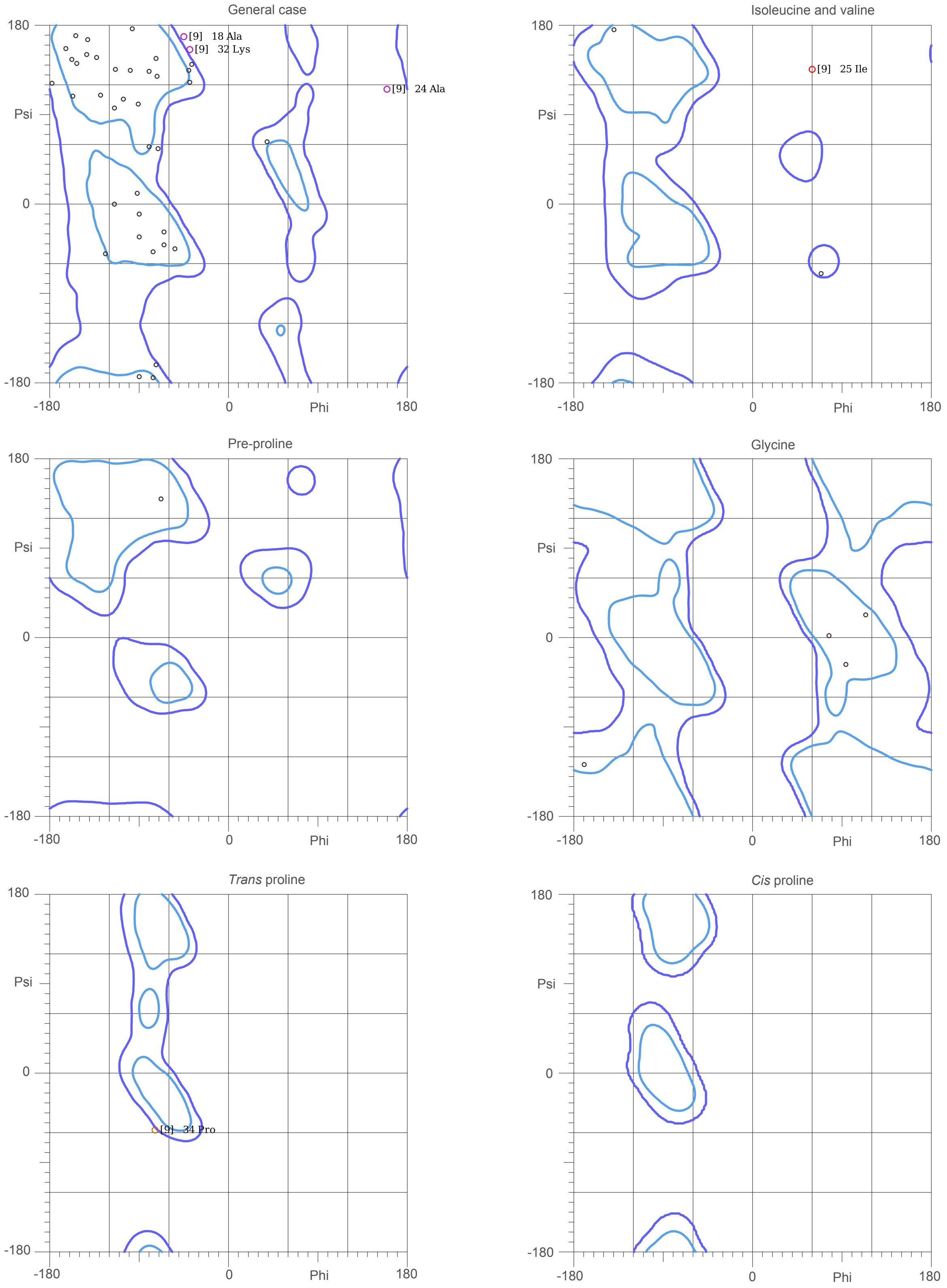

69.4% (34/49) of all residues were in favored (98%) regions.
89.8% (44/49) of all residues were in allowed (>99.8%) regions.

There were 5 outliers (phi, psi):

- [9] 18 Ala (-45.5, 169.3)
- [9] 24 Ala (160.6, 116.9)
- [9] 25 Ile (60.8, 136.2)
- [9] 32 Lys (-39.1, 156.4)
- [9] 34 Pro (-75.0, -57.4)

MolProbity Ramachandran analysis

1afp_trimmedH.pdb, model 10

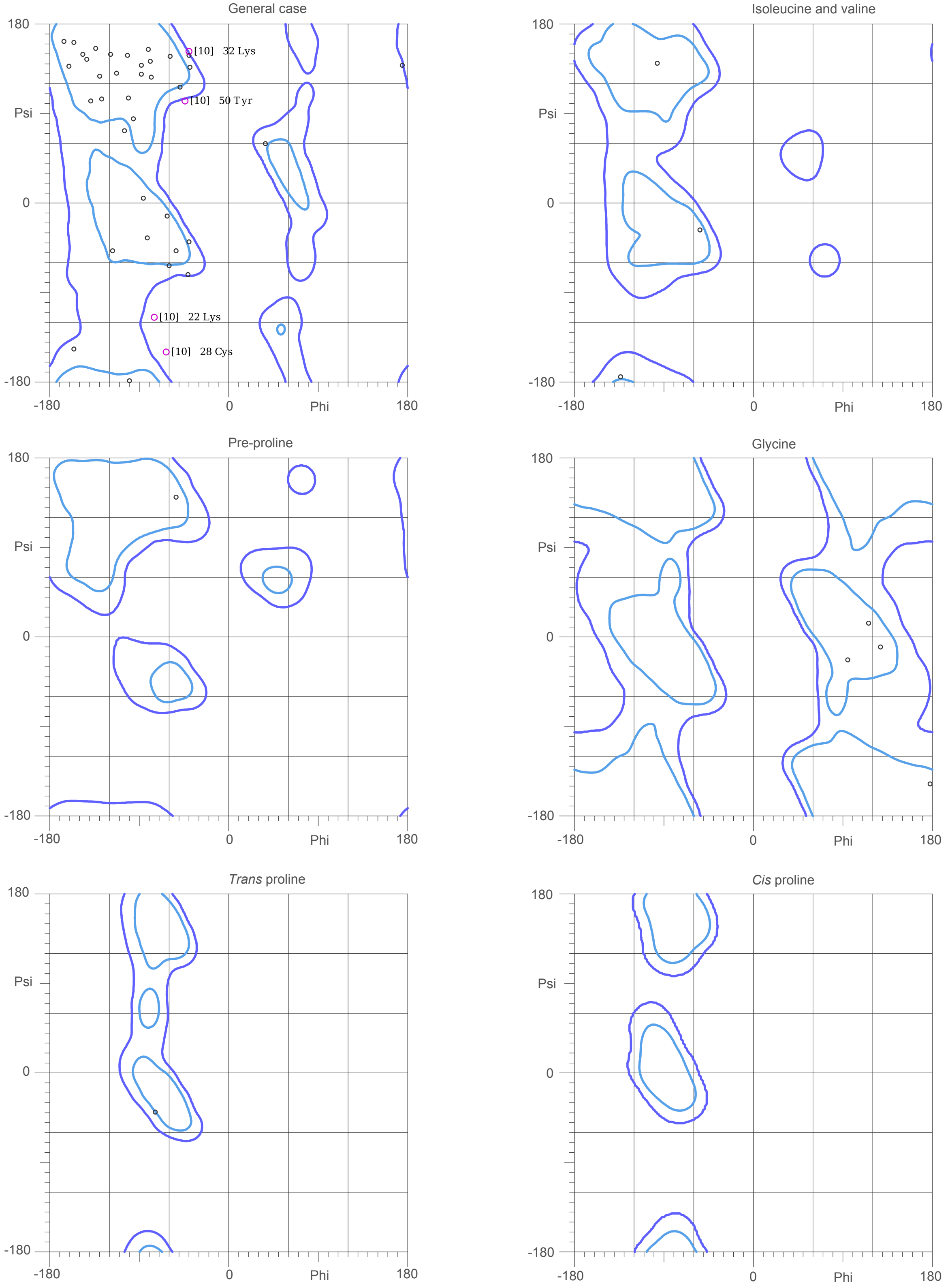

69.4% (34/49) of all residues were in favored (98%) regions.
91.8% (45/49) of all residues were in allowed (>99.8%) regions.

There were 4 outliers (phi, psi):
[10] 22 Lys (-75.9, -115.2)
[10] 28 Cys (-63.6, -150.3)
[10] 32 Lys (-40.8, 153.5)
[10] 50 Tyr (-44.6, 103.0)

MolProbity Ramachandran analysis

1afp_trimmedH.pdb, model 11

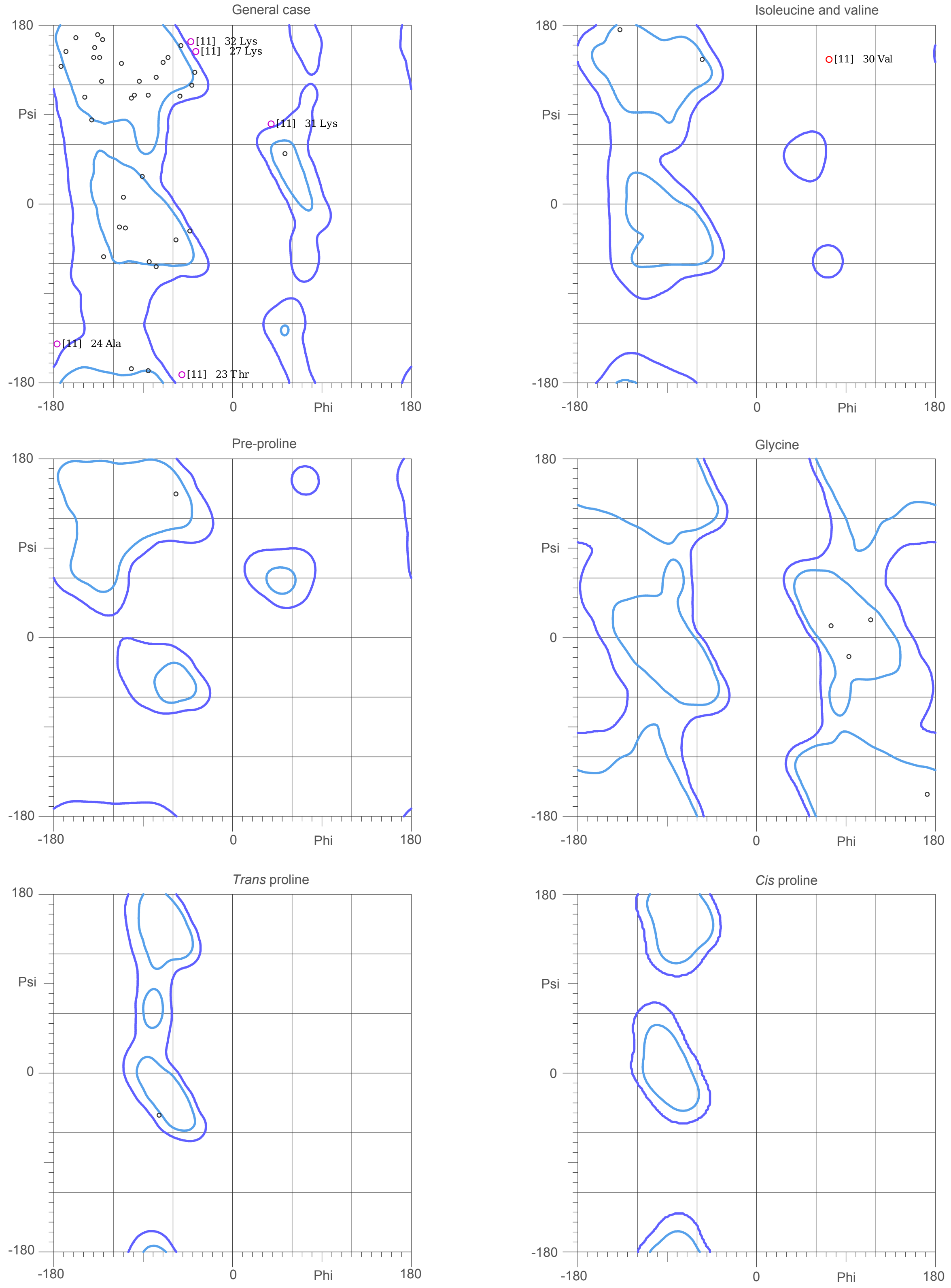

63.3% (31/49) of all residues were in favored (98%) regions.
87.8% (43/49) of all residues were in allowed (>99.8%) regions.

There were 6 outliers (phi, psi):
[11] 23 Thr (-51.6, -172.4)
[11] 24 Ala (-177.0, -141.7)
[11] 27 Lys (-37.0, 154.9)
[11] 30 Val (73.7, 146.7)
[11] 31 Lys (39.8, 81.6)
[11] 32 Lys (-43.0, 164.2)

MolProbity Ramachandran analysis

1afp_trimmedH.pdb, model 12

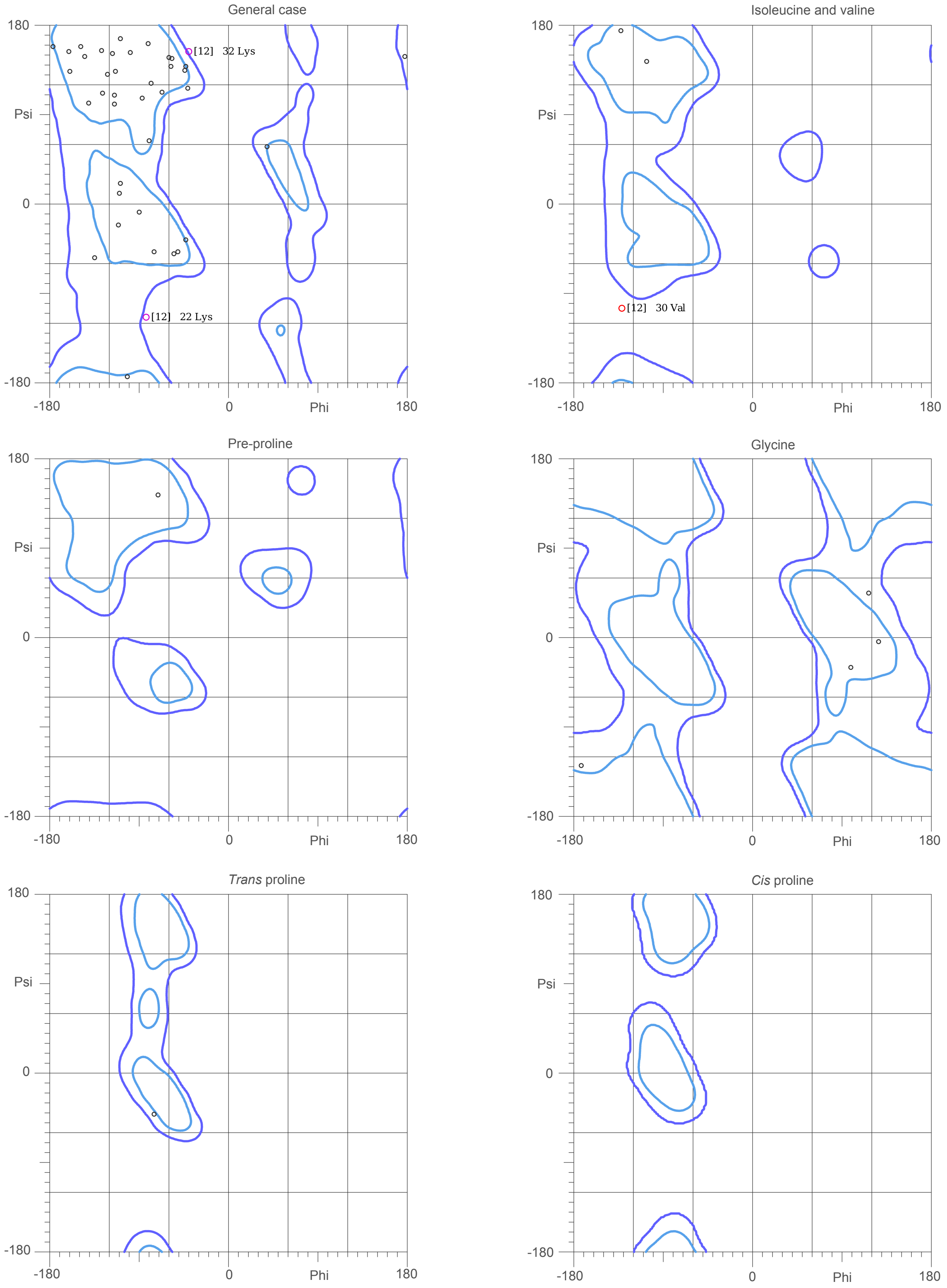

75.5% (37/49) of all residues were in favored (98%) regions.
93.9% (46/49) of all residues were in allowed (>99.8%) regions.

There were 3 outliers (phi, psi):
[12] 22 Lys (-83.6, -114.6)
[12] 30 Val (-132.1, -106.0)
[12] 32 Lys (-40.1, 154.1)

MolProbity Ramachandran analysis

1afp_trimmedH.pdb, model 13

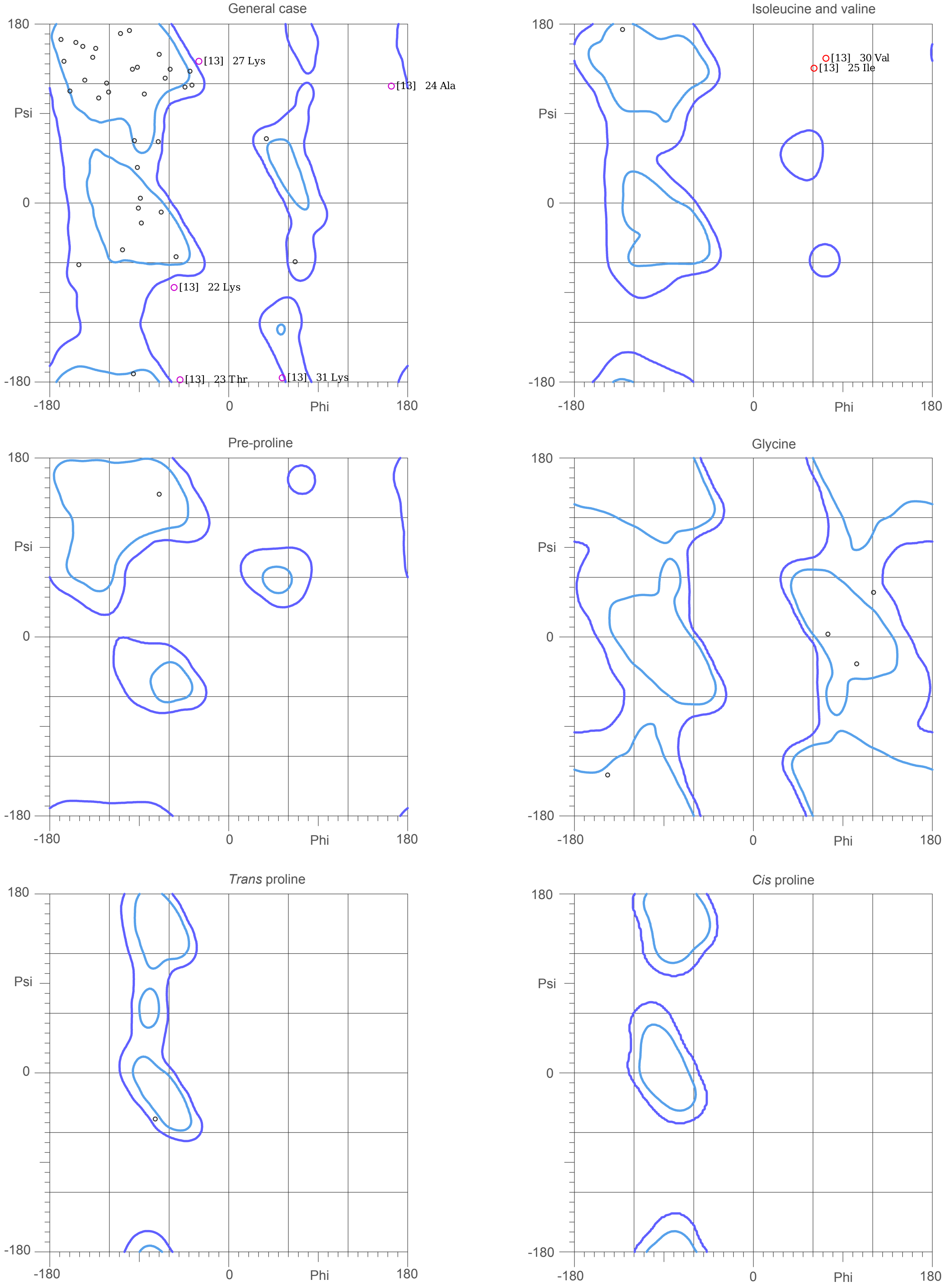

65.3% (32/49) of all residues were in favored (98%) regions.
85.7% (42/49) of all residues were in allowed (>99.8%) regions.

There were 7 outliers (phi, psi):
[13] 22 Lys (-55.8, -85.6)
[13] 23 Thr (-49.9, -178.8)
[13] 24 Ala (164.7, 118.9)
[13] 25 Ile (61.8, 136.6)
[13] 27 Lys (-31.0, 143.4)
[13] 30 Val (73.5, 146.7)
[13] 31 Lys (54.6, -176.4)

http://kinemage.biochem.duke.edu

Lovell, Davis, et al. Proteins 50:437 (2003)

MolProbity Ramachandran analysis

1afp_trimmedH.pdb, model 14

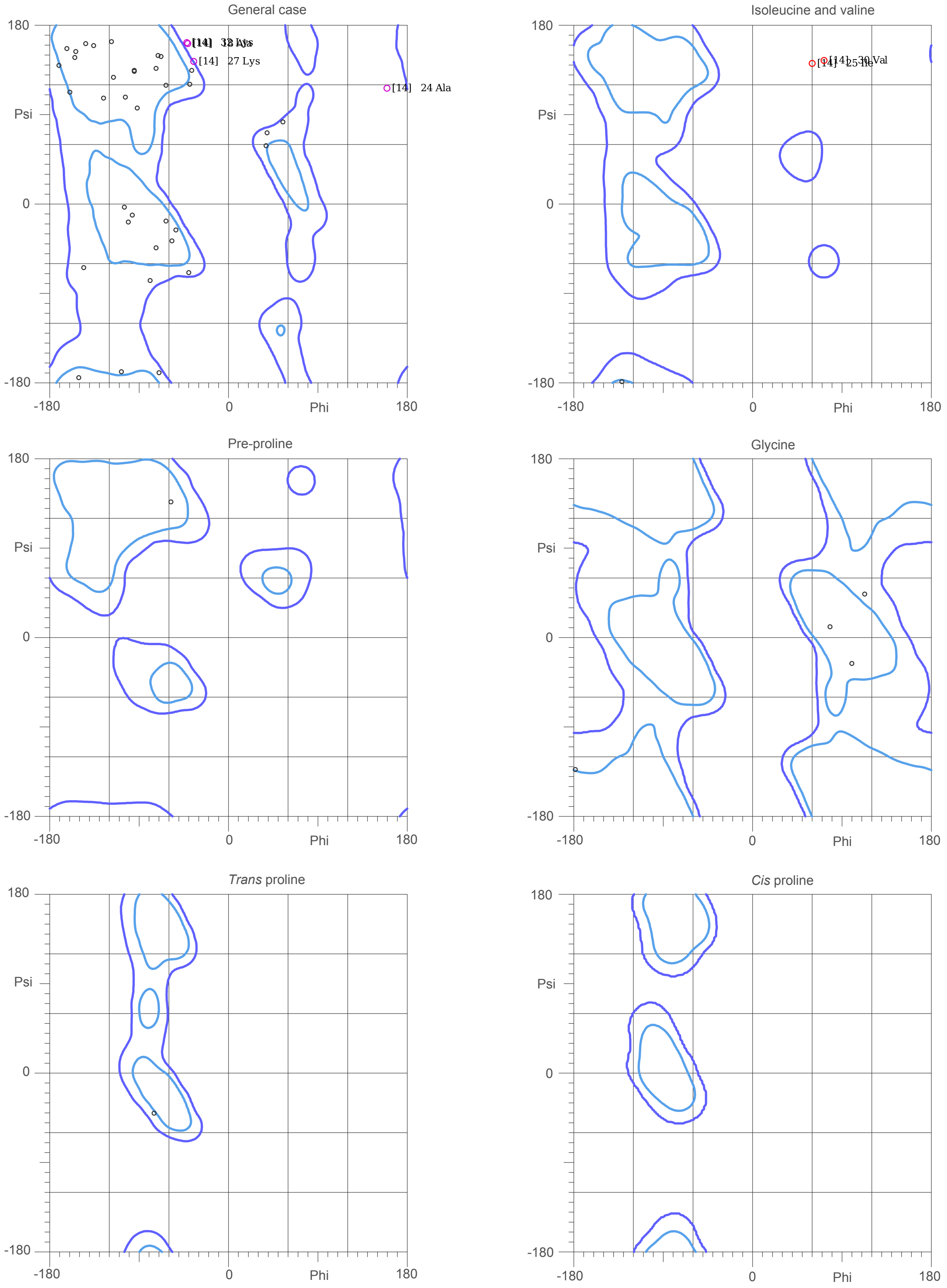

59.2% (29/49) of all residues were in favored (98%) regions.
87.8% (43/49) of all residues were in allowed (>99.8%) regions.

There were 6 outliers (phi, psi):
- [14] 18 Ala (-41.8, 162.2)
- [14] 24 Ala (160.1, 117.7)
- [14] 25 Ile (60.7, 142.8)
- [14] 27 Lys (-35.1, 144.1)
- [14] 30 Val (72.5, 145.9)
- [14] 32 Lys (-42.4, 163.2)

MolProbity Ramachandran analysis

1afp_trimmedH.pdb, model 15

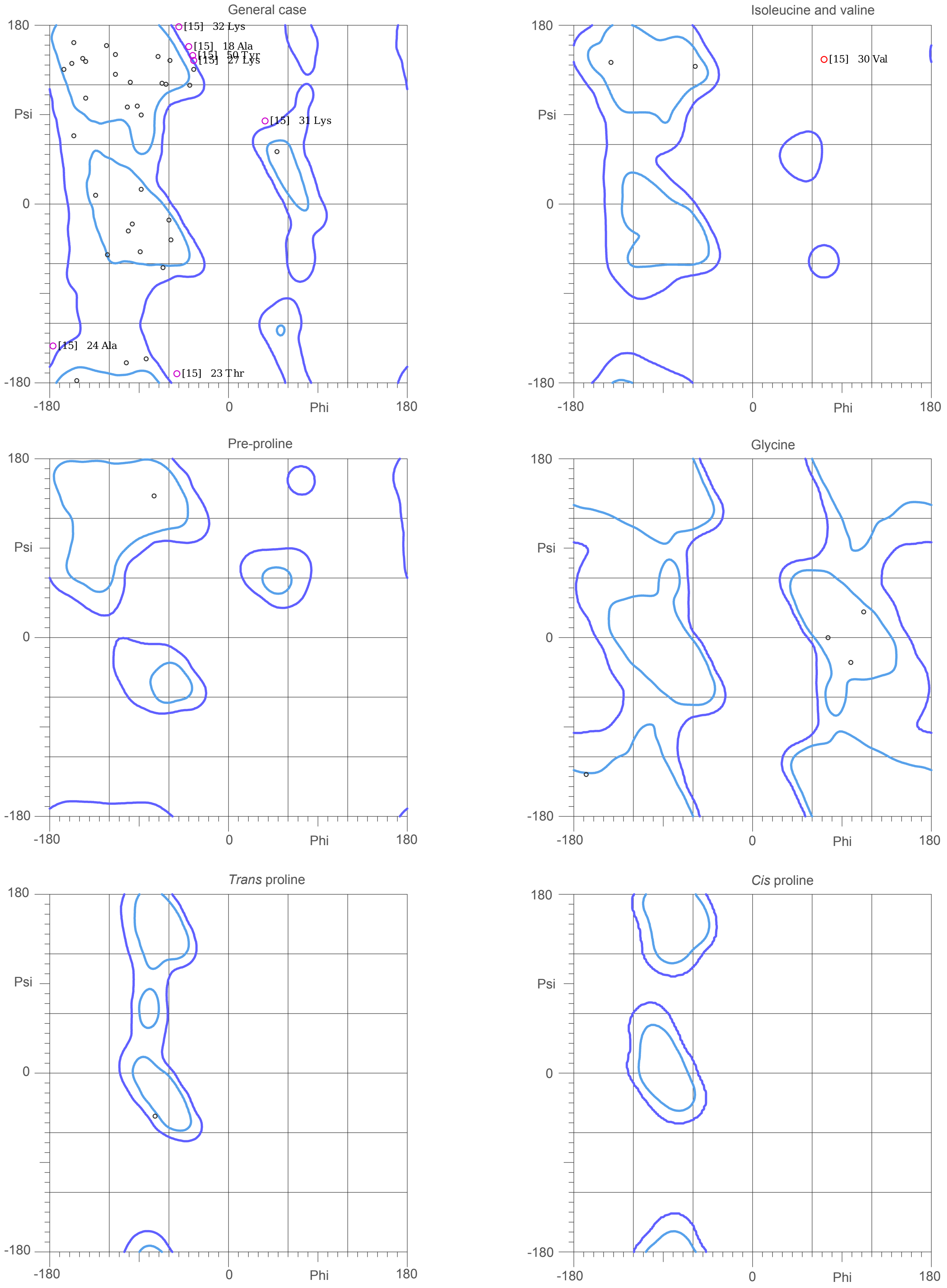

67.3% (33/49) of all residues were in favored (98%) regions.
83.7% (41/49) of all residues were in allowed (>99.8%) regions.

There were 8 outliers (phi, psi):
[15] 18 Ala (-40.8, 159.5)
[15] 23 Thr (-52.1, -171.6)
[15] 24 Ala (-177.6, -143.1)
[15] 27 Lys (-35.6, 145.4)
[15] 30 Val (72.9, 146.0)
[15] 31 Lys (37.8, 84.5)
[15] 32 Lys (-50.3, 179.2)
[15] 50 Tyr (-36.6, 150.1)

MolProbity Ramachandran analysis

1afp_trimmedH.pdb, model 16

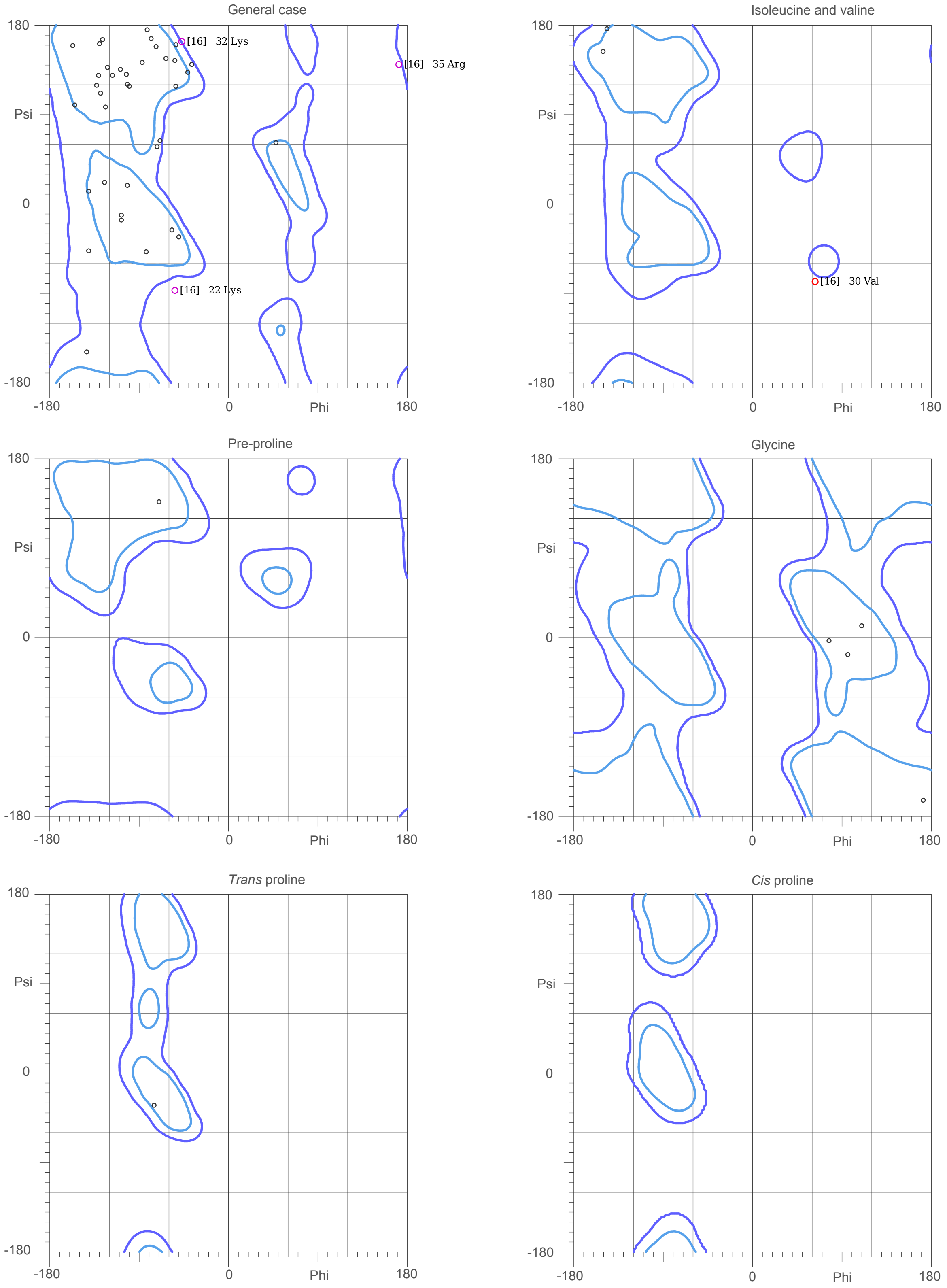

77.6% (38/49) of all residues were in favored (98%) regions.
91.8% (45/49) of all residues were in allowed (>99.8%) regions.

There were 4 outliers (phi, psi):
- [16] 22 Lys (-54.3, -87.6)
- [16] 30 Val (64.0, -78.6)
- [16] 32 Lys (-47.1, 164.1)
- [16] 35 Arg (172.6, 141.8)

MolProbity Ramachandran analysis

1afp_trimmedH.pdb, model 17

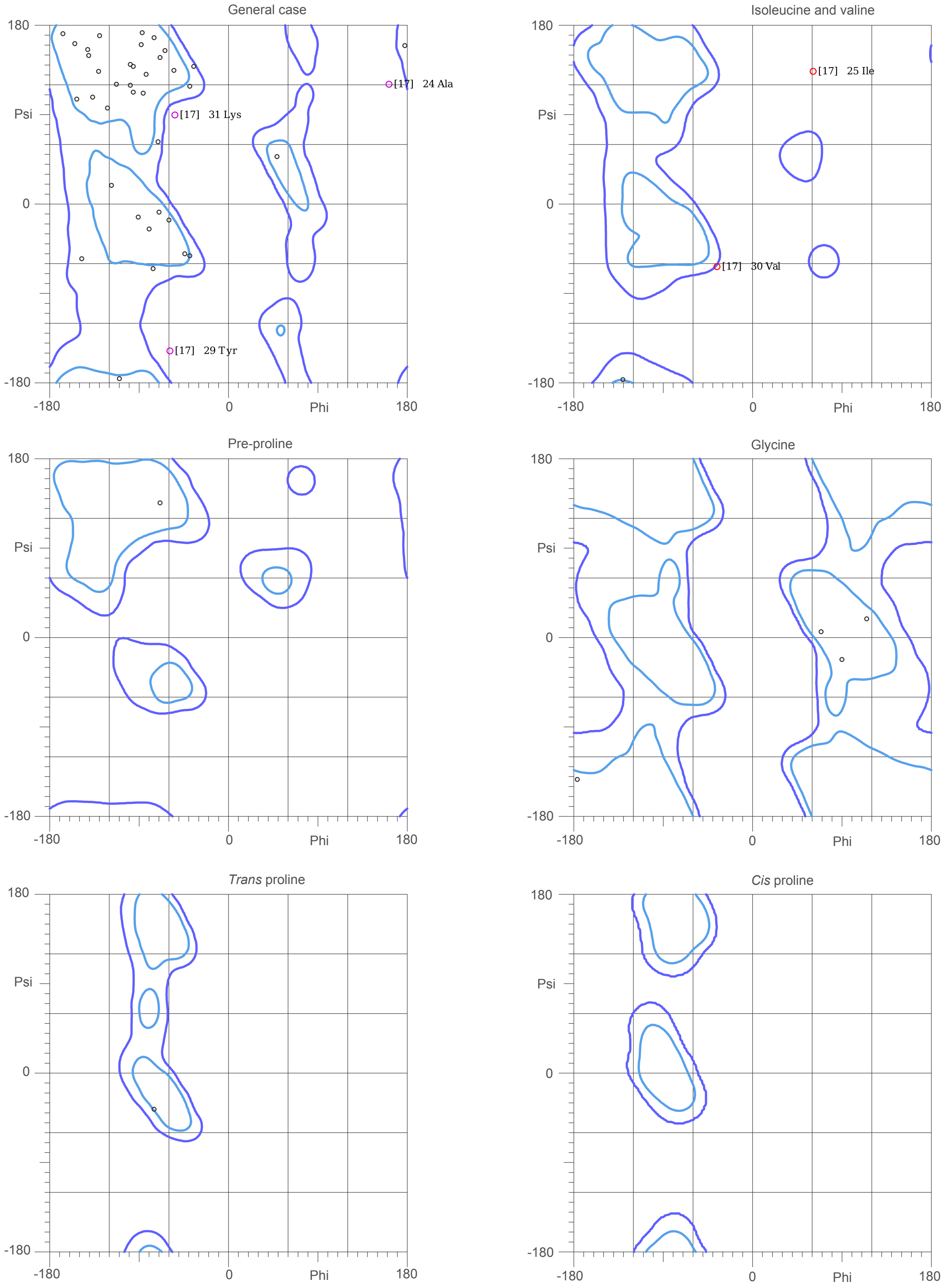

77.6% (38/49) of all residues were in favored (98%) regions.
89.8% (44/49) of all residues were in allowed (>99.8%) regions.

There were 5 outliers (phi, psi):
[17] 24 Ala (162.7, 121.1)
[17] 25 Ile (61.1, 134.6)
[17] 29 Tyr (-59.3, -148.3)
[17] 30 Val (-36.4, -63.1)
[17] 31 Lys (-54.3, 90.1)

Lovell, Davis, et al. Proteins 50:437 (2003)

MolProbity Ramachandran analysis

1afp_trimmedH.pdb, model 18

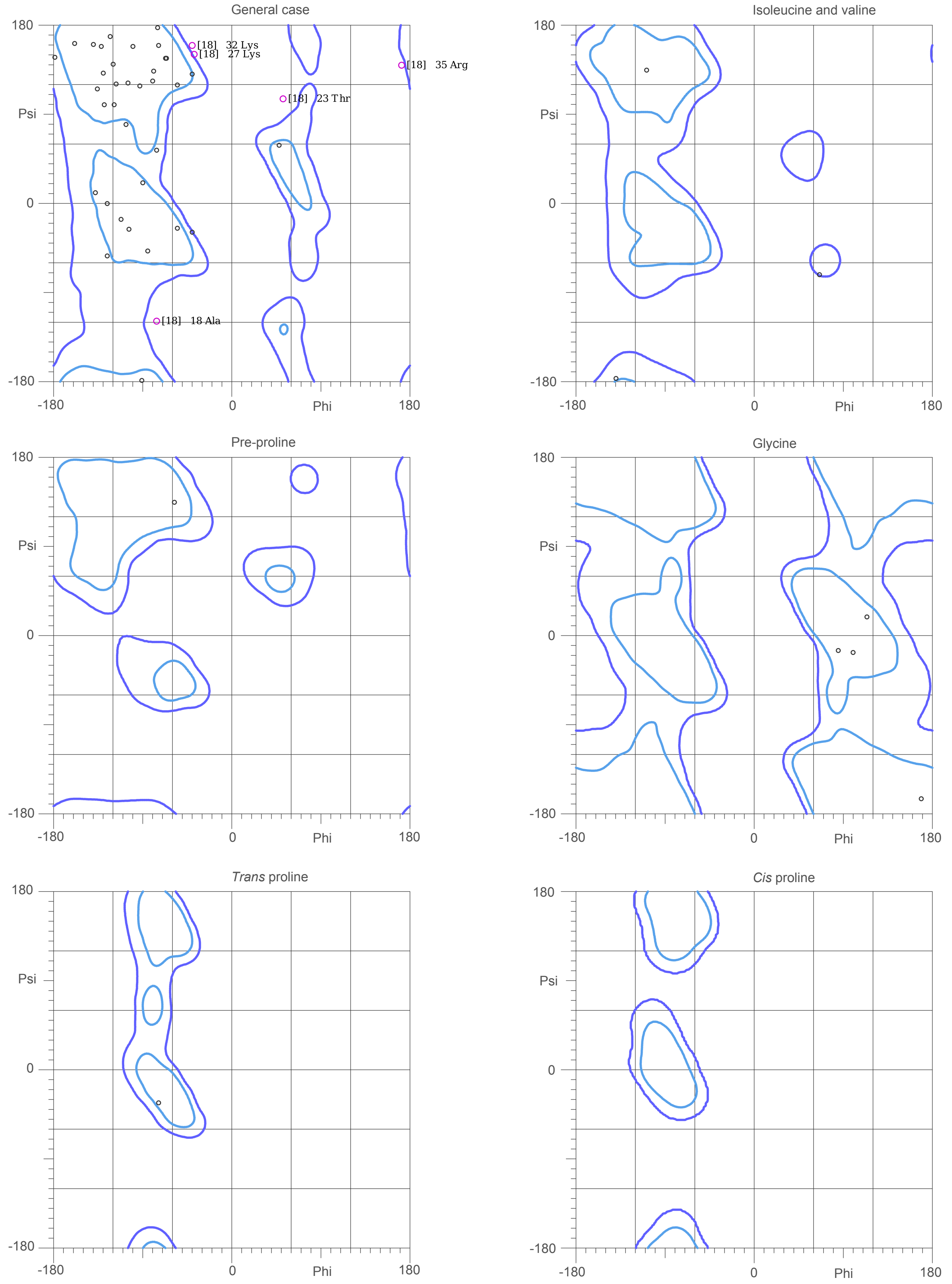

75.5% (37/49) of all residues were in favored (98%) regions.
89.8% (44/49) of all residues were in allowed (>99.8%) regions.

There were 5 outliers (phi, psi):
[18] 18 Ala (-76.7, -119.1)
[18] 23 Thr (52.9, 106.2)
[18] 27 Lys (-38.6, 151.2)
[18] 32 Lys (-40.4, 160.1)
[18] 35 Arg (172.7, 140.8)

MolProbity Ramachandran analysis

1afp_trimmedH.pdb, model 19

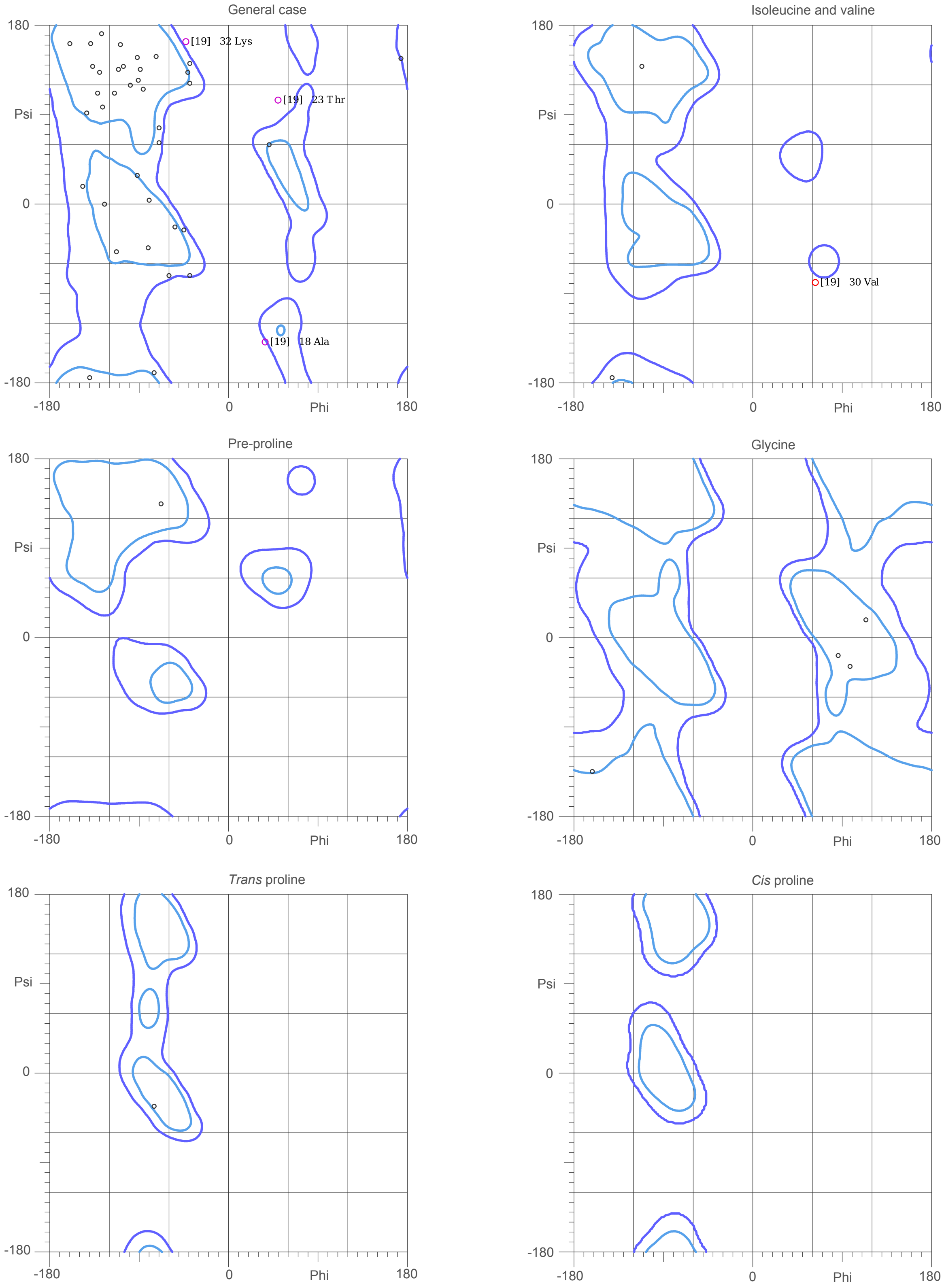

65.3% (32/49) of all residues were in favored (98%) regions.
91.8% (45/49) of all residues were in allowed (>99.8%) regions.

There were 4 outliers (phi, psi):
[19] 18 Ala (37.3, -140.0)
[19] 23 Thr (50.8, 105.8)
[19] 30 Val (63.8, -79.0)
[19] 32 Lys (-43.4, 164.8)

MolProbity Ramachandran analysis

1afp_trimmedH.pdb, model 20

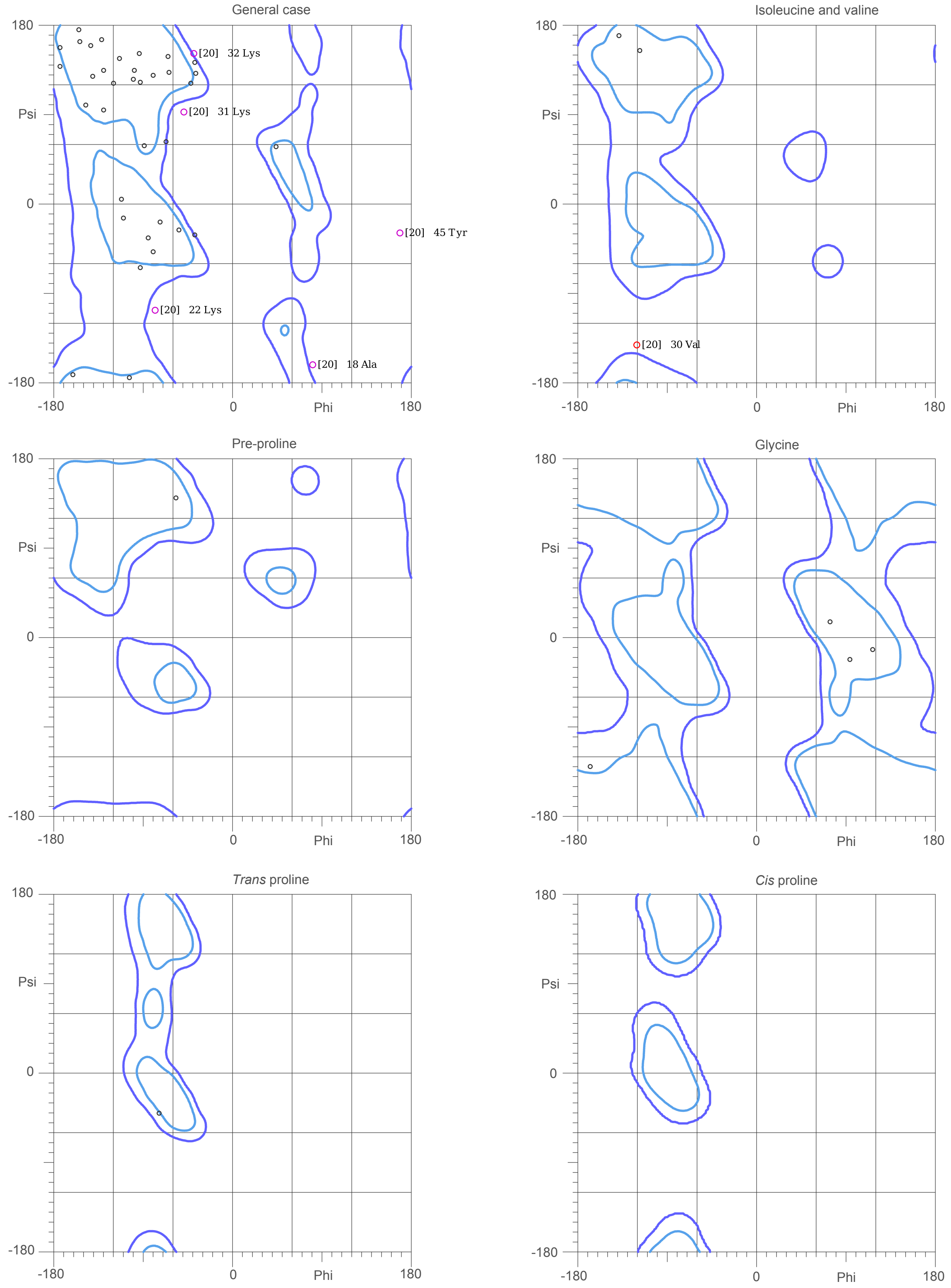

71.4% (35/49) of all residues were in favored (98%) regions.
87.8% (43/49) of all residues were in allowed (>99.8%) regions.

There were 6 outliers (phi, psi):
- [20] 18 Ala (81.5, -162.3)
- [20] 22 Lys (-79.0, -107.1)
- [20] 30 Val (-121.2, -142.4)
- [20] 31 Lys (-49.2, 93.6)
- [20] 32 Lys (-39.3, 152.2)
- [20] 45 Tyr (169.7, -29.7)

MolProbity Ramachandran analysis

1afp_trimmedH.pdb, model 21

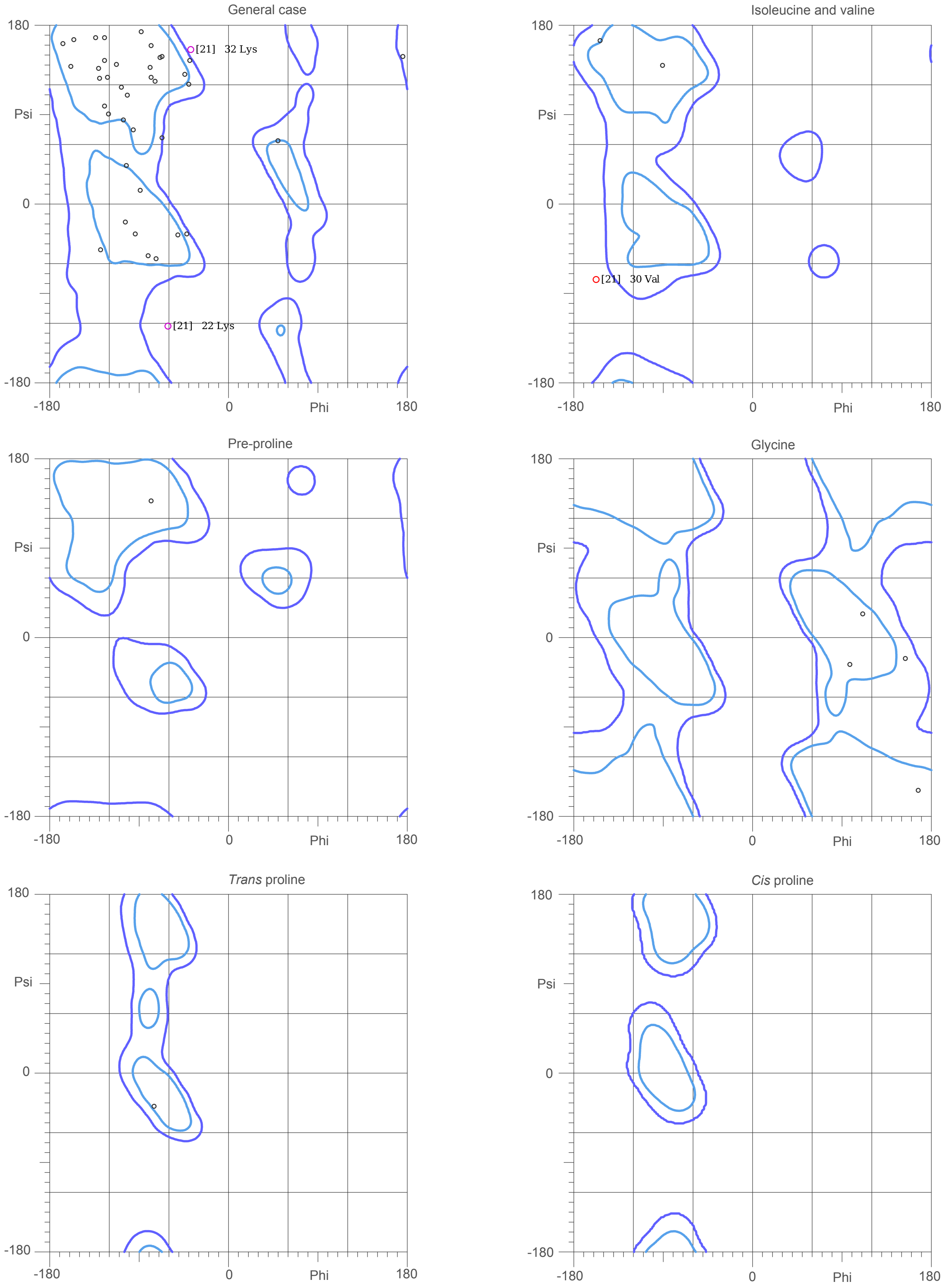

73.5% (36/49) of all residues were in favored (98%) regions.
93.9% (46/49) of all residues were in allowed (>99.8%) regions.

There were 3 outliers (phi, psi):
[21] 22 Lys (-61.6, -124.0)
[21] 30 Val (-158.2, -76.2)
[21] 32 Lys (-38.9, 156.5)

MolProbity Ramachandran analysis

1afp_trimmedH.pdb, model 22

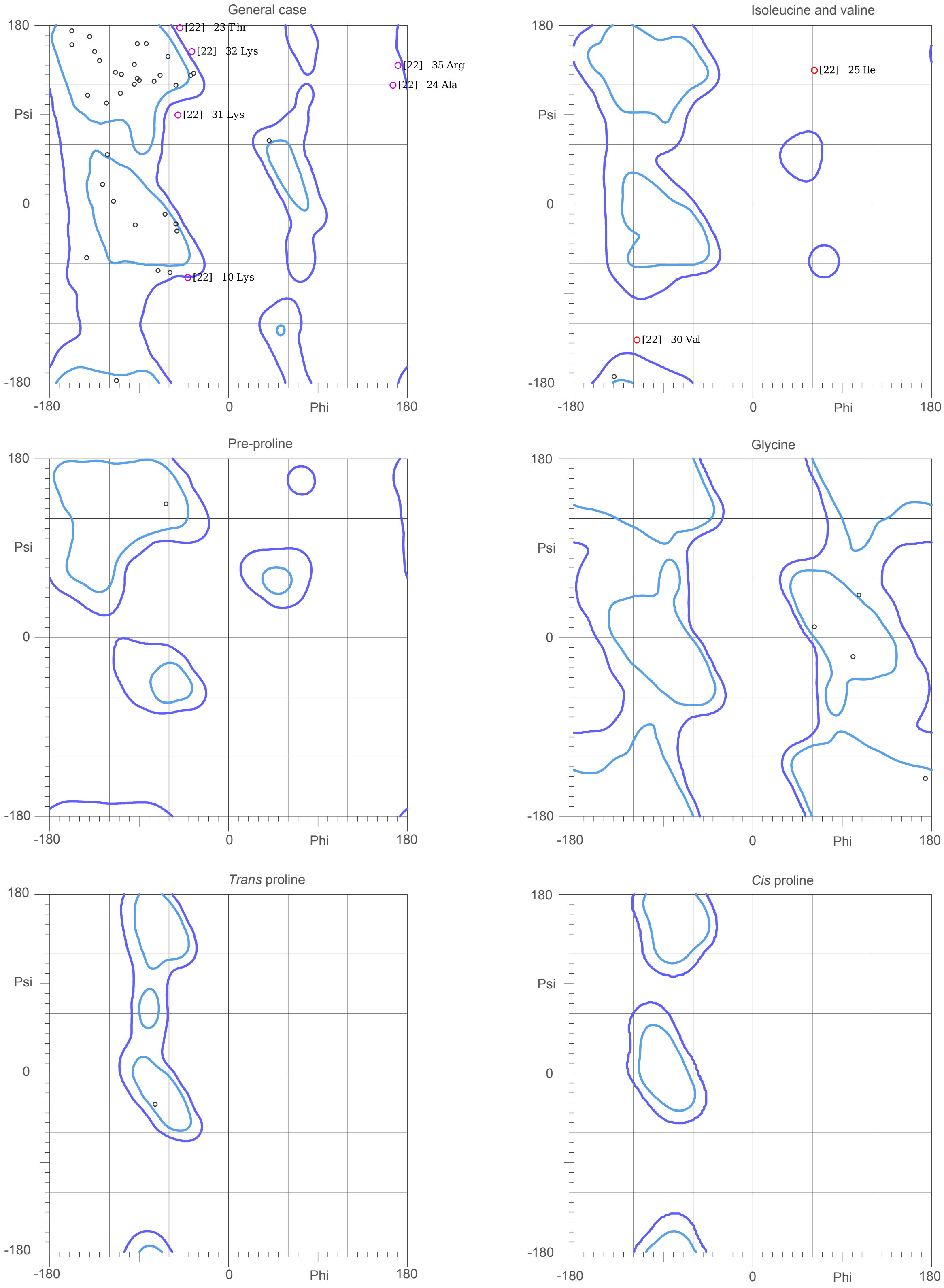

65.3% (32/49) of all residues were in favored (98%) regions.
83.7% (41/49) of all residues were in allowed (>99.8%) regions.

There were 8 outliers (phi, psi):
[22] 10 Lys (-41.4, -74.4)
[22] 23 Thr (-49.2, 178.7)
[22] 24 Ala (166.4, 120.9)
[22] 25 Ile (62.1, 135.5)
[22] 30 Val (-117.0, -137.0)
[22] 31 Lys (-51.7, 90.7)
[22] 32 Lys (-37.8, 154.7)
[22] 35 Arg (171.8, 140.5)

MolProbity Ramachandran analysis

1afp_trimmedH.pdb, model 23

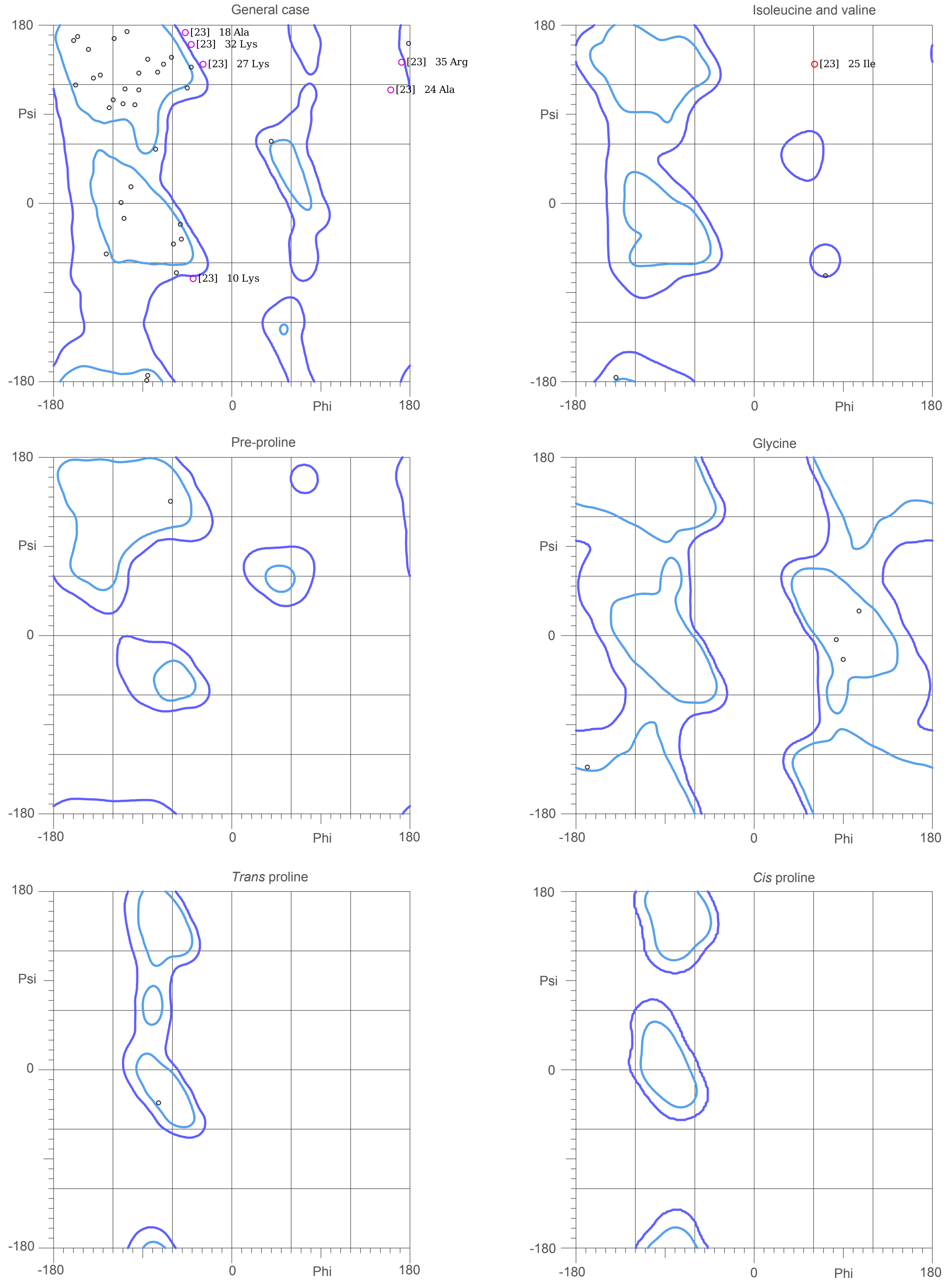

65.3% (32/49) of all residues were in favored (98%) regions.
85.7% (42/49) of all residues were in allowed (>99.8%) regions.

There were 7 outliers (phi, psi):
[23] 10 Lys (-39.6, -76.6)
[23] 18 Ala (-47.6, 173.1)
[23] 24 Ala (161.7, 115.9)
[23] 25 Ile (61.6, 141.8)
[23] 27 Lys (-29.8, 141.2)
[23] 32 Lys (-41.2, 161.3)
[23] 35 Arg (172.7, 143.5)

MolProbity Ramachandran analysis

1afp_trimmedH.pdb, model 24

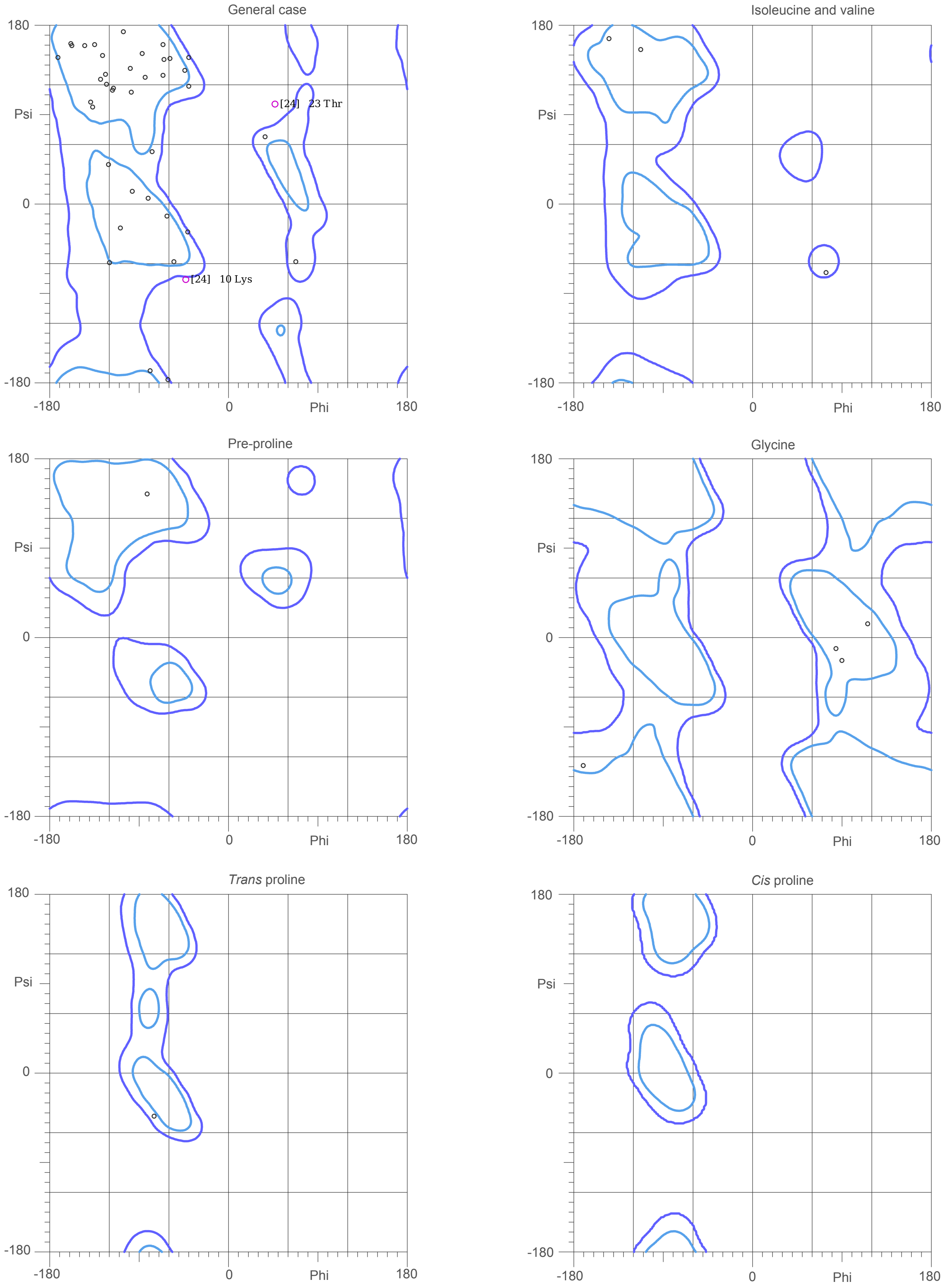

71.4% (35/49) of all residues were in favored (98%) regions.
95.9% (47/49) of all residues were in allowed (>99.8%) regions.

There were 2 outliers (phi, psi):
[24] 10 Lys (-43.3, -76.5)
[24] 23 Thr (47.9, 101.7)

MolProbity Ramachandran analysis

1afp_trimmedH.pdb, model 25

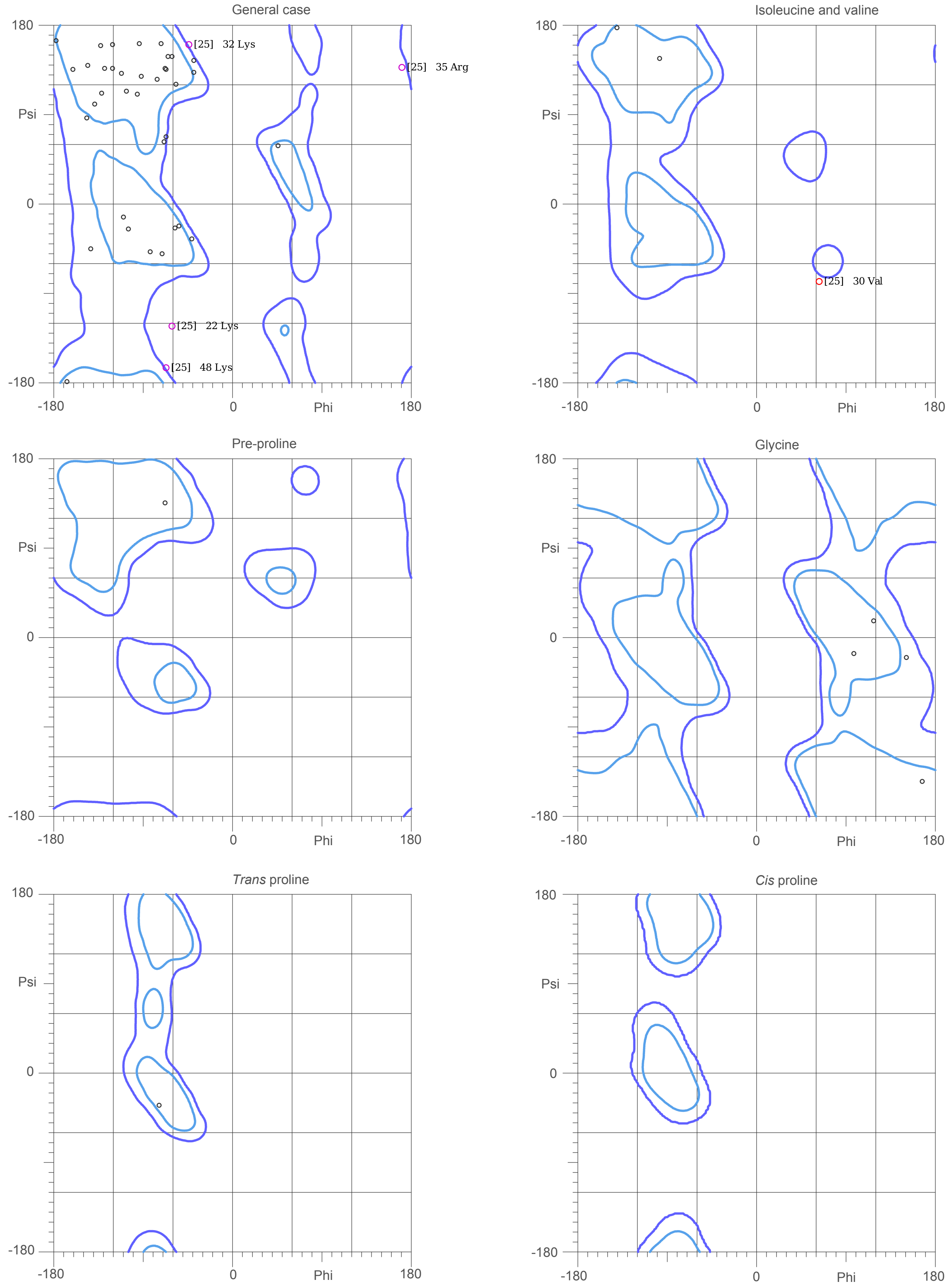

71.4% (35/49) of all residues were in favored (98%) regions.
89.8% (44/49) of all residues were in allowed (>99.8%) regions.

There were 5 outliers (phi, psi):
[25] 22 Lys (-61.3, -123.6)
[25] 30 Val (63.9, -78.2)
[25] 32 Lys (-44.5, 161.5)
[25] 35 Arg (171.8, 138.9)
[25] 48 Lys (-67.0, -165.1)

MolProbity Ramachandran analysis

1afp_trimmedH.pdb, model 26

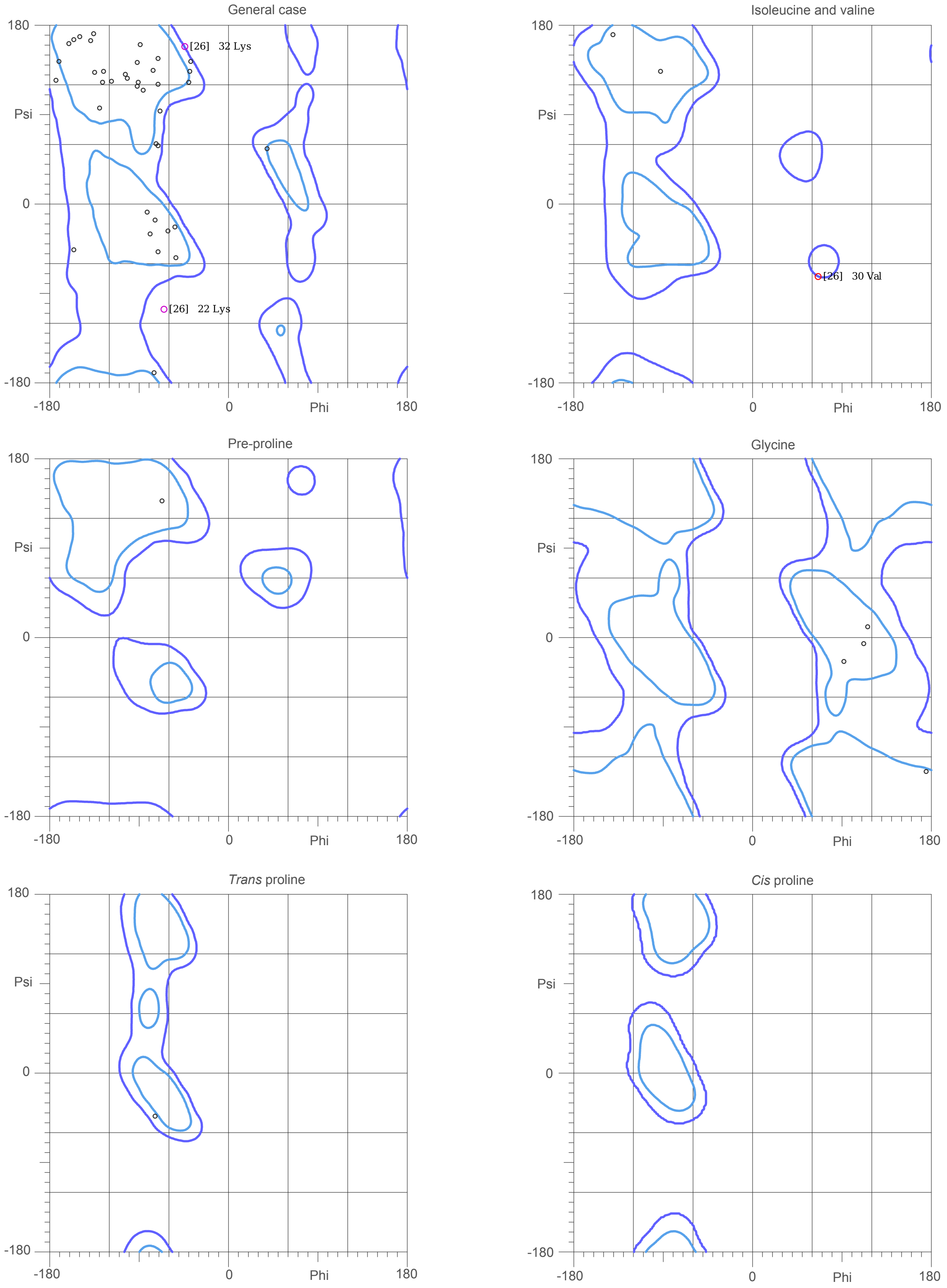

71.4% (35/49) of all residues were in favored (98%) regions.
93.9% (46/49) of all residues were in allowed (>99.8%) regions.

There were 3 outliers (phi, psi):
[26] 22 Lys (-65.1, -106.5)
[26] 30 Val (66.3, -73.3)
[26] 32 Lys (-44.5, 159.3)

MolProbity Ramachandran analysis

1afp_trimmedH.pdb, model 27

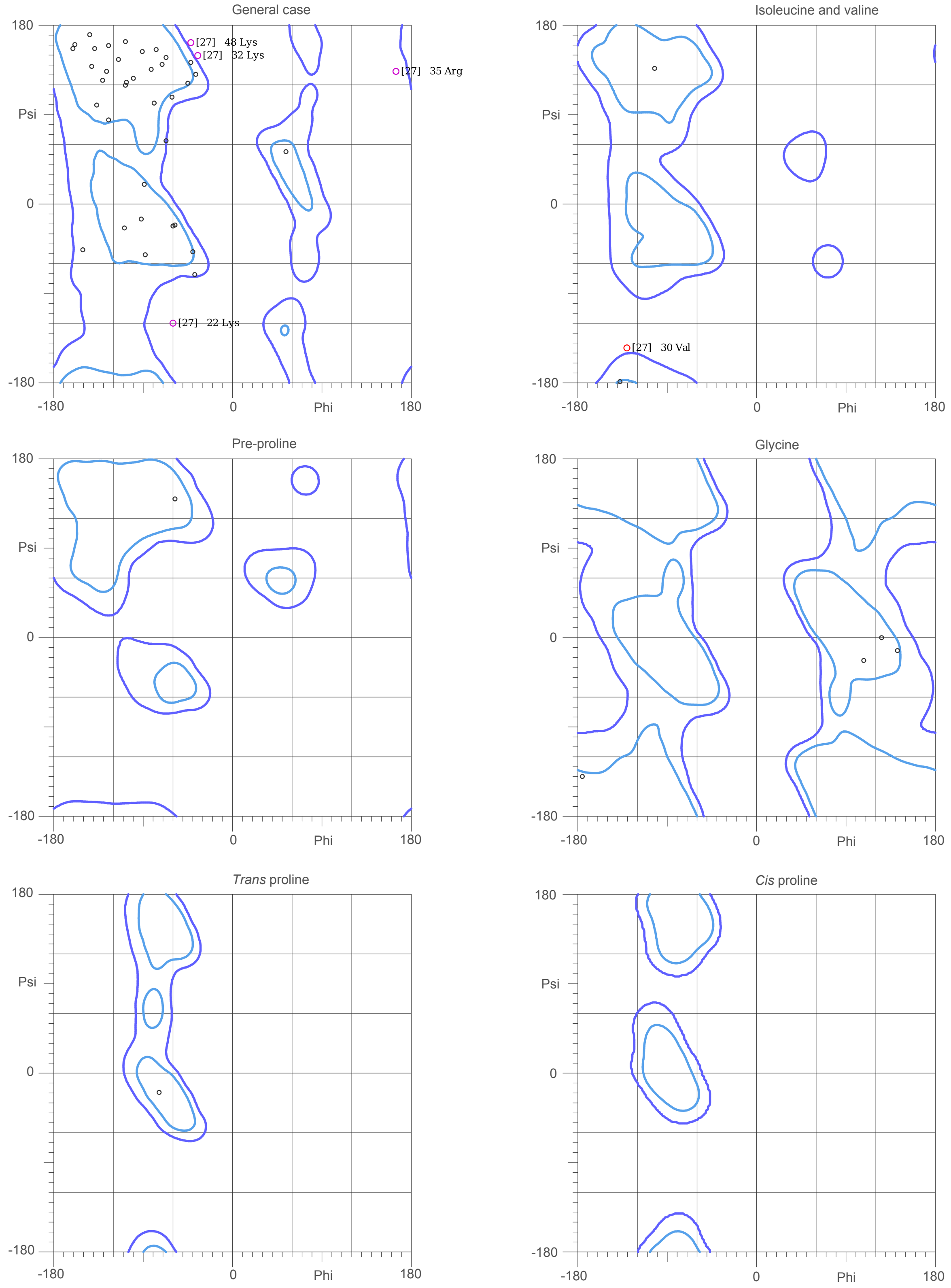

77.6% (38/49) of all residues were in favored (98%) regions.
89.8% (44/49) of all residues were in allowed (>99.8%) regions.

There were 5 outliers (phi, psi):
[27] 22 Lys (-61.0, -120.4)
[27] 30 Val (-131.4, -145.2)
[27] 32 Lys (-35.9, 150.4)
[27] 35 Arg (166.0, 134.3)
[27] 48 Lys (-42.5, 163.1)

MolProbity Ramachandran analysis

1afp_trimmedH.pdb, model 28

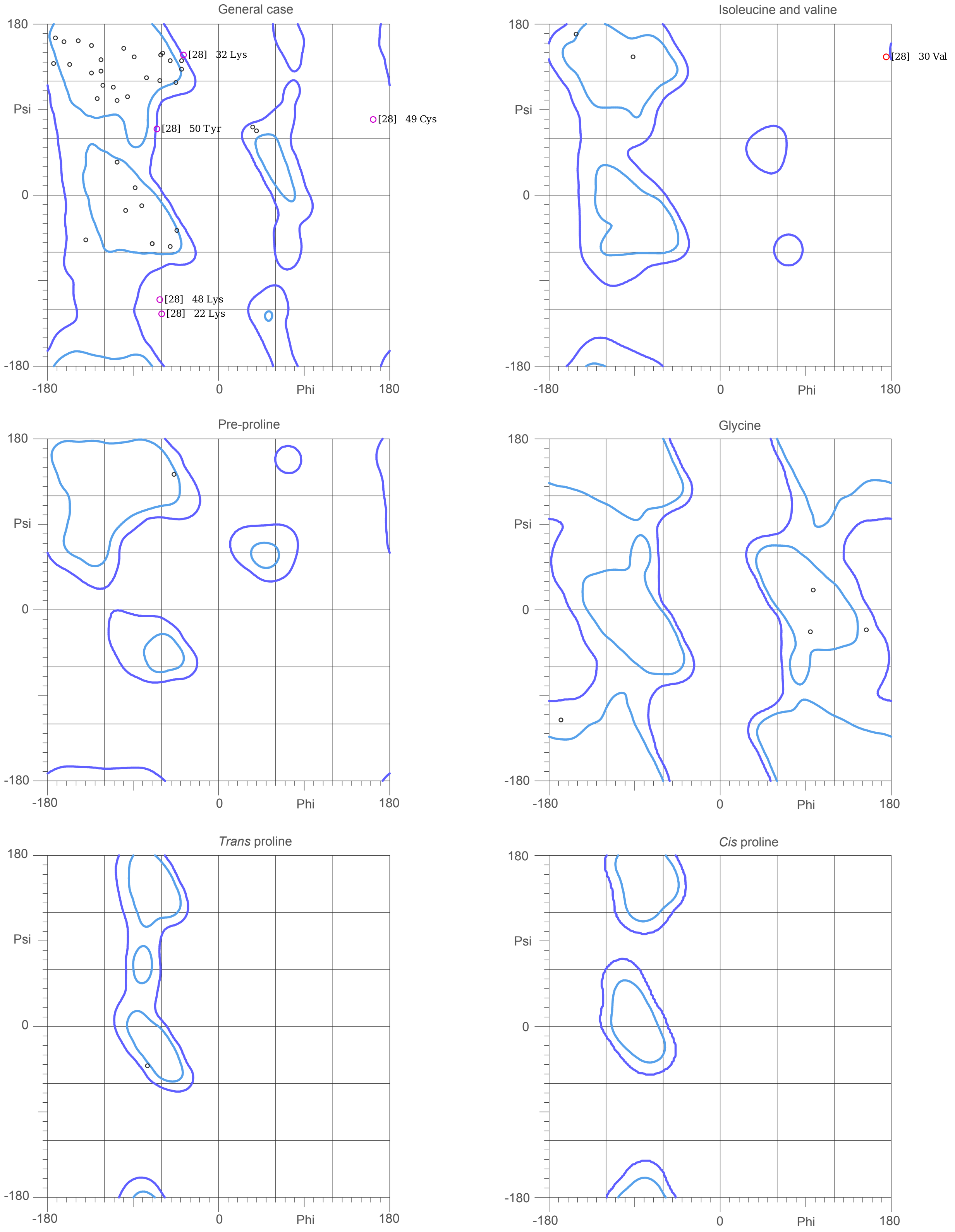

67.3% (33/49) of all residues were in favored (98%) regions.
87.8% (43/49) of all residues were in allowed (>99.8%) regions.

There were 6 outliers (phi, psi):

[28] 22 Lys (-61.0, -125.1)
[28] 30 Val (175.3, 146.0)
[28] 32 Lys (-37.5, 148.5)
[28] 48 Lys (-62.7, -110.0)
[28] 49 Cys (163.8, 80.3)
[28] 50 Tyr (-65.3, 70.8)

http://kinemage.biochem.duke.edu

Lovell, Davis, et al. Proteins 50:437 (2003)

MolProbity Ramachandran analysis

1afp_trimmedH.pdb, model 29

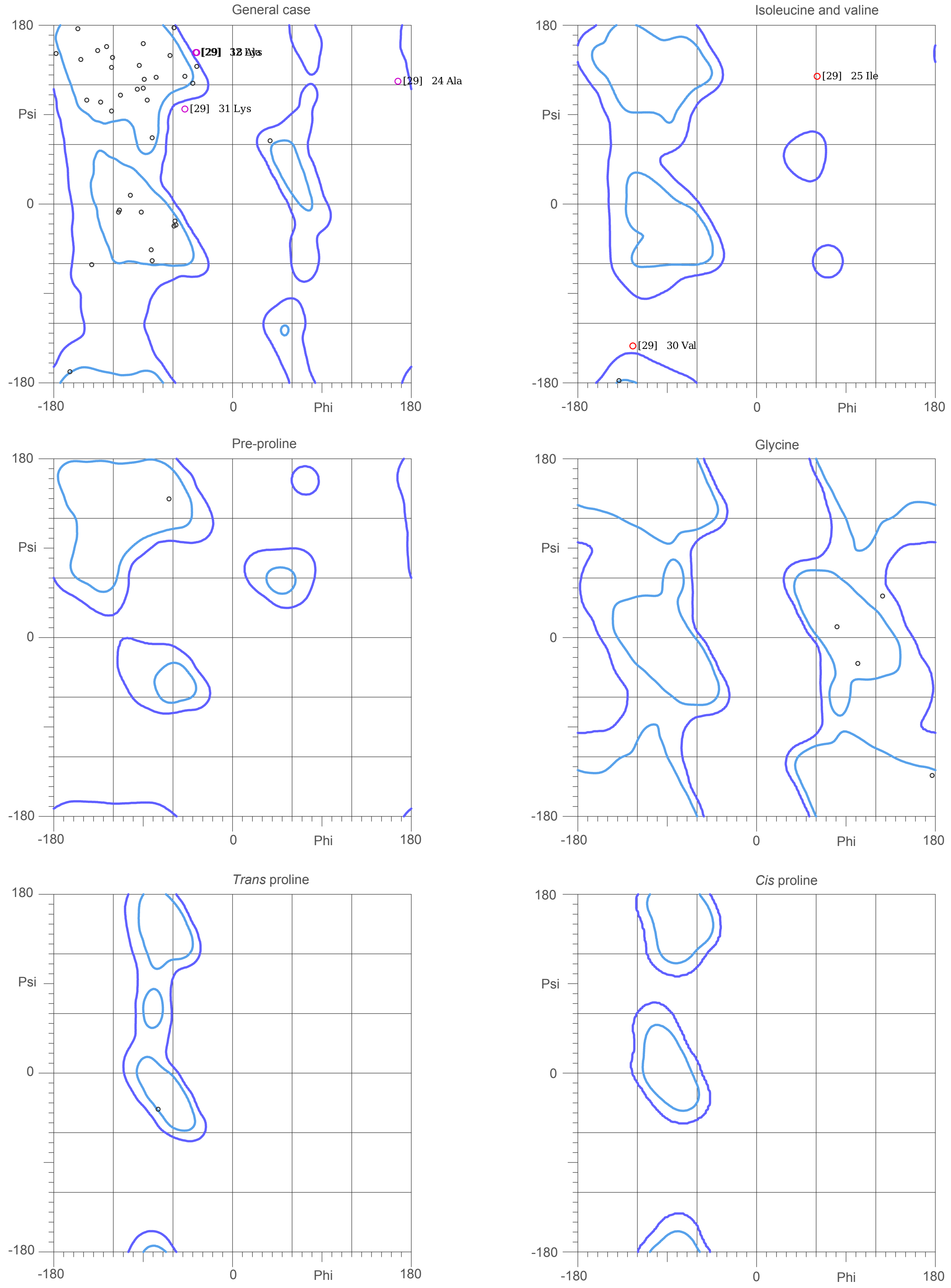

69.4% (34/49) of all residues were in favored (98%) regions.
87.8% (43/49) of all residues were in allowed (>99.8%) regions.

There were 6 outliers (phi, psi):
[29] 18 Ala (-36.4, 153.9)
[29] 24 Ala (167.3, 124.4)
[29] 25 Ile (61.8, 130.0)
[29] 30 Val (-125.8, -143.8)
[29] 31 Lys (-48.4, 96.2)
[29] 32 Lys (-37.4, 153.8)

http://kinemage.biochem.duke.edu

Lovell, Davis, et al. Proteins 50:437 (2003)

MolProbity Ramachandran analysis

1afp_trimmedH.pdb, model 30

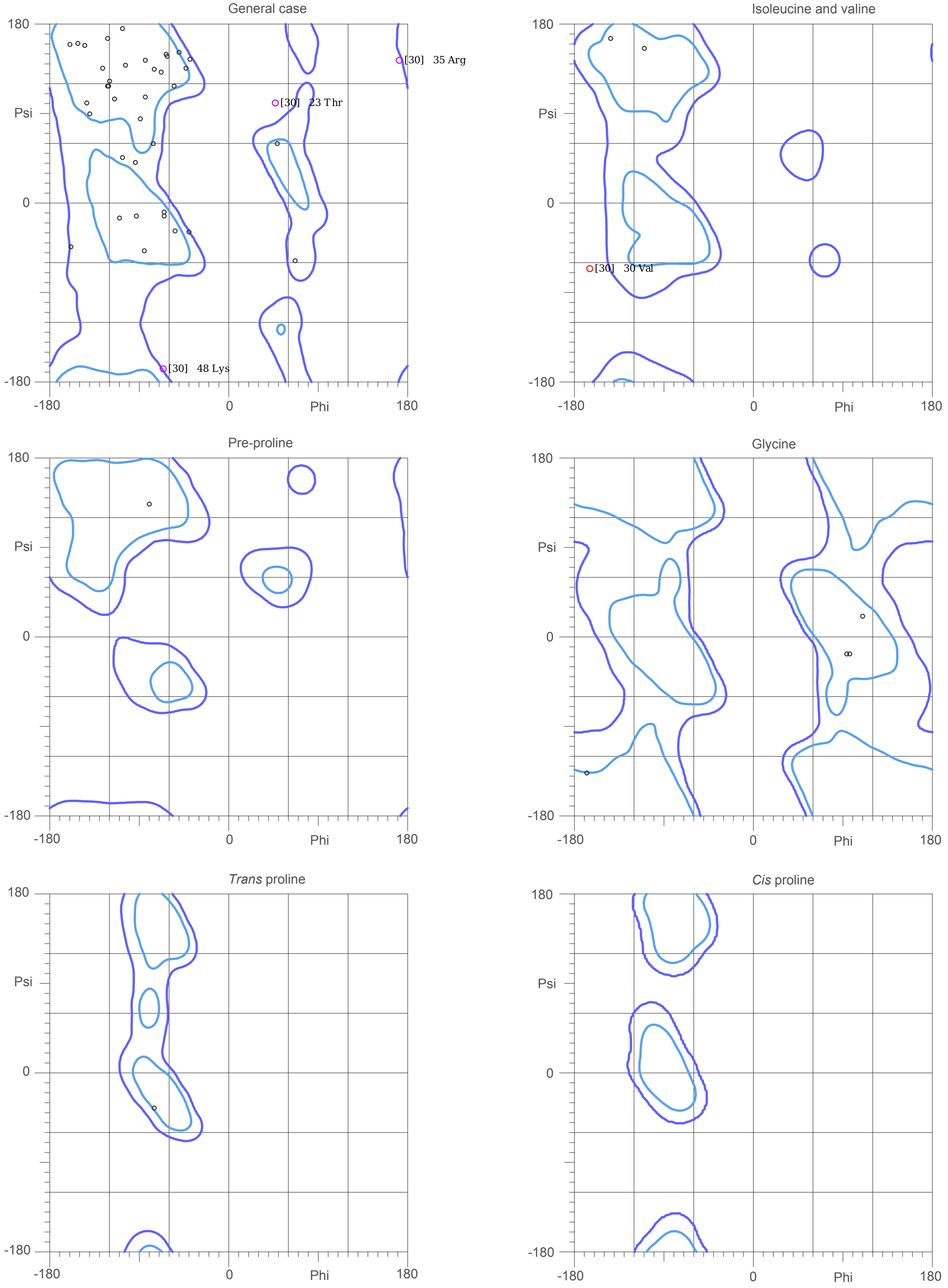

77.6% (38/49) of all residues were in favored (98%) regions.
91.8% (45/49) of all residues were in allowed (>99.8%) regions.

There were 4 outliers (phi, psi):
- [30] 23 Thr (47.1, 101.1)
- [30] 30 Val (-165.4, -66.1)
- [30] 35 Arg (172.8, 144.8)
- [30] 48 Lys (-66.4, -167.1)

MolProbity Ramachandran analysis

1afp_trimmedH.pdb, model 31

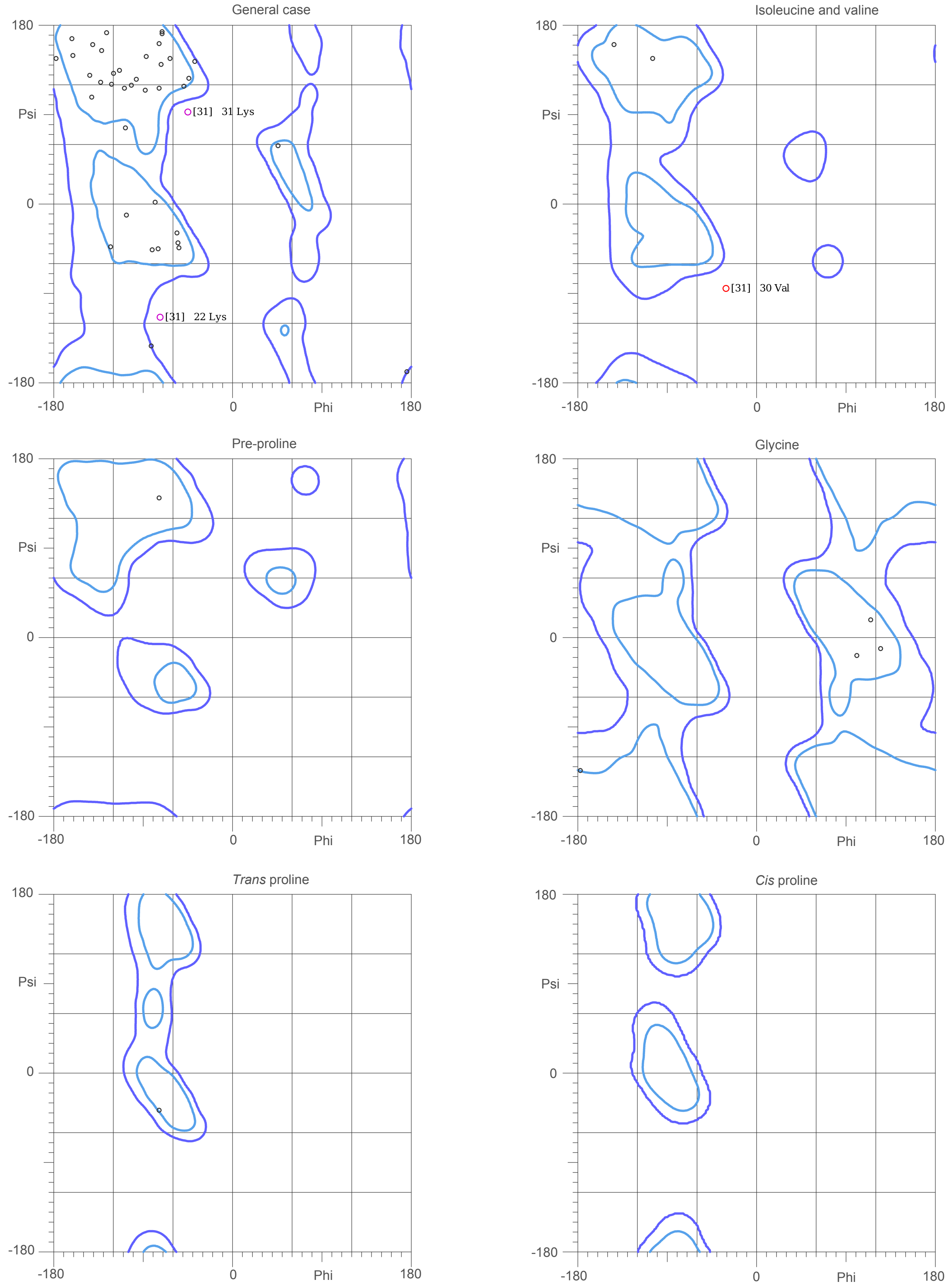

79.6% (39/49) of all residues were in favored (98%) regions.
93.9% (46/49) of all residues were in allowed (>99.8%) regions.

There were 3 outliers (phi, psi):
[31] 22 Lys (-73.3, -114.8)
[31] 30 Val (-32.0, -85.0)
[31] 31 Lys (-45.3, 93.9)

http://kinemage.biochem.duke.edu

Lovell, Davis, et al. Proteins 50:437 (2003)

MolProbity Ramachandran analysis

1afp_trimmedH.pdb, model 32

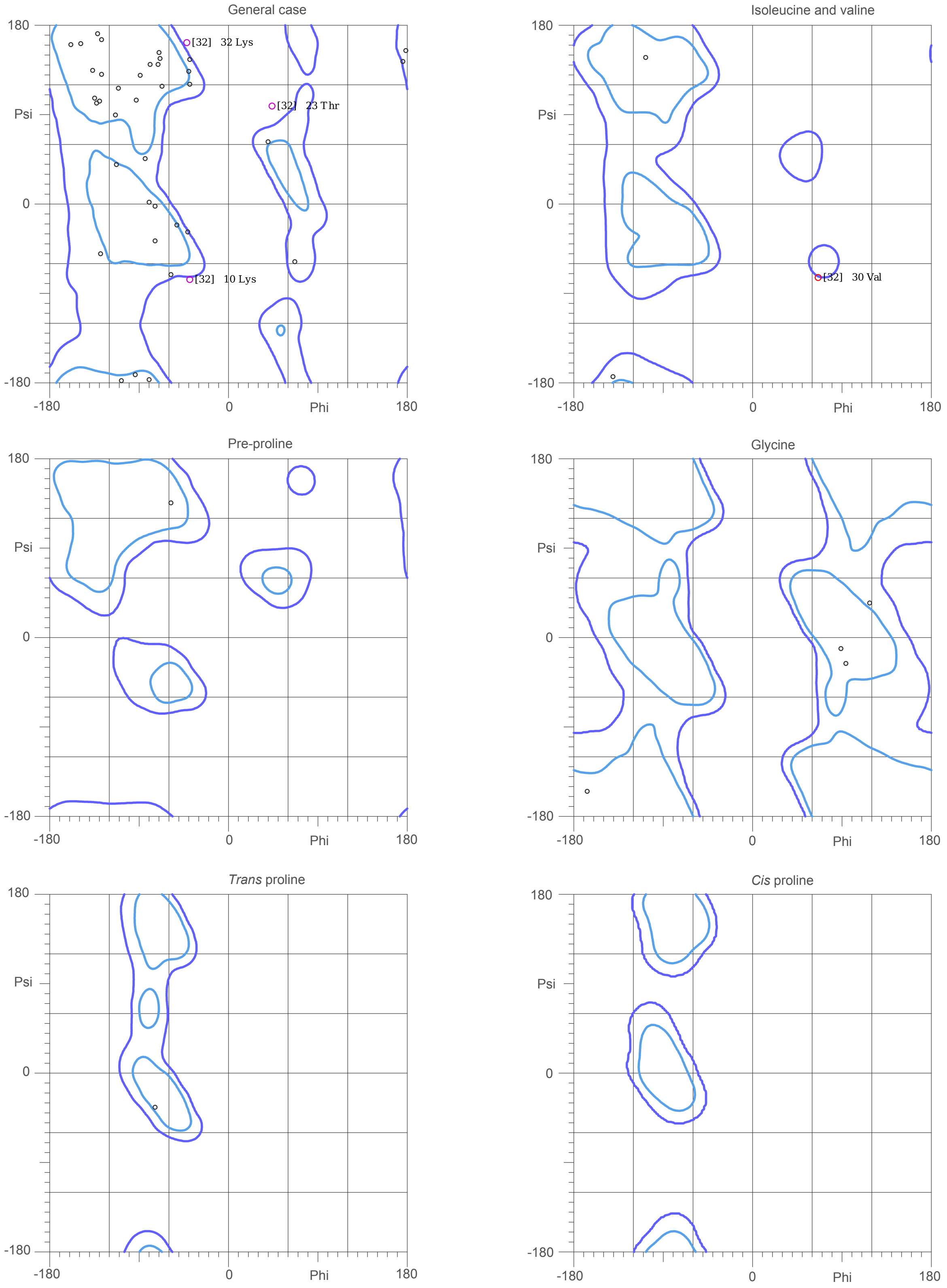

65.3% (32/49) of all residues were in favored (98%) regions.
91.8% (45/49) of all residues were in allowed (>99.8%) regions.

There were 4 outliers (phi, psi):
[32] 10 Lys (-39.4, -76.2)
[32] 23 Thr (44.9, 99.6)
[32] 30 Val (66.1, -74.5)
[32] 32 Lys (-42.7, 163.8)

MolProbity Ramachandran analysis

1afp_trimmedH.pdb, model 33

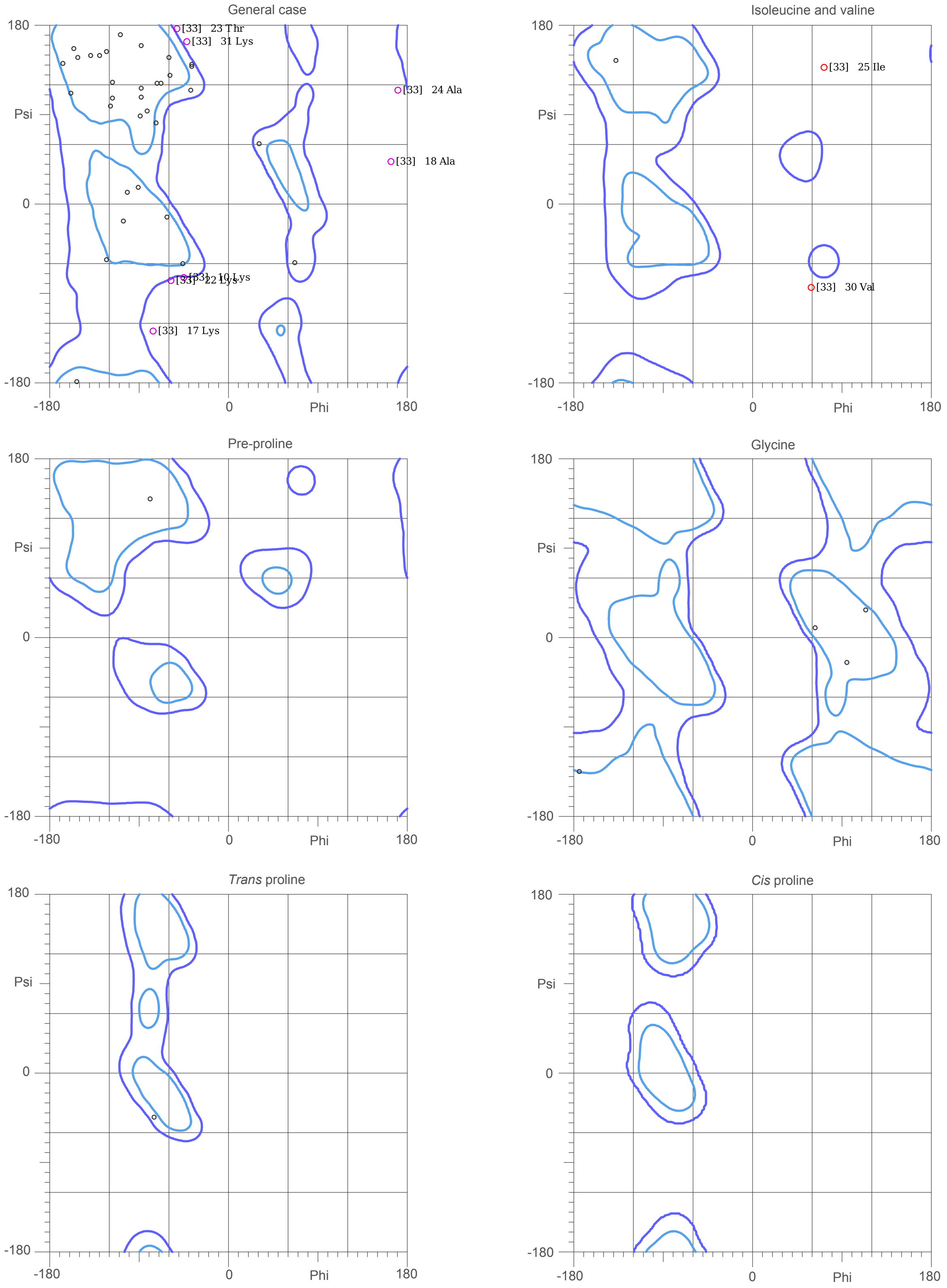

65.3% (32/49) of all residues were in favored (98%) regions.
81.6% (40/49) of all residues were in allowed (>99.8%) regions.

There were 9 outliers (phi, psi):

[33] 10 Lys (-45.1, -74.7)
[33] 17 Lys (-77.0, -128.8)
[33] 18 Ala (164.5, 43.4)
[33] 22 Lys (-58.7, -77.5)
[33] 23 Thr (-52.9, 177.7)
[33] 24 Ala (171.4, 115.7)
[33] 25 Ile (72.2, 138.2)
[33] 30 Val (59.7, -84.1)
[33] 31 Lys (-43.0, 164.3)

MolProbity Ramachandran analysis

1afp_trimmedH.pdb, model 34

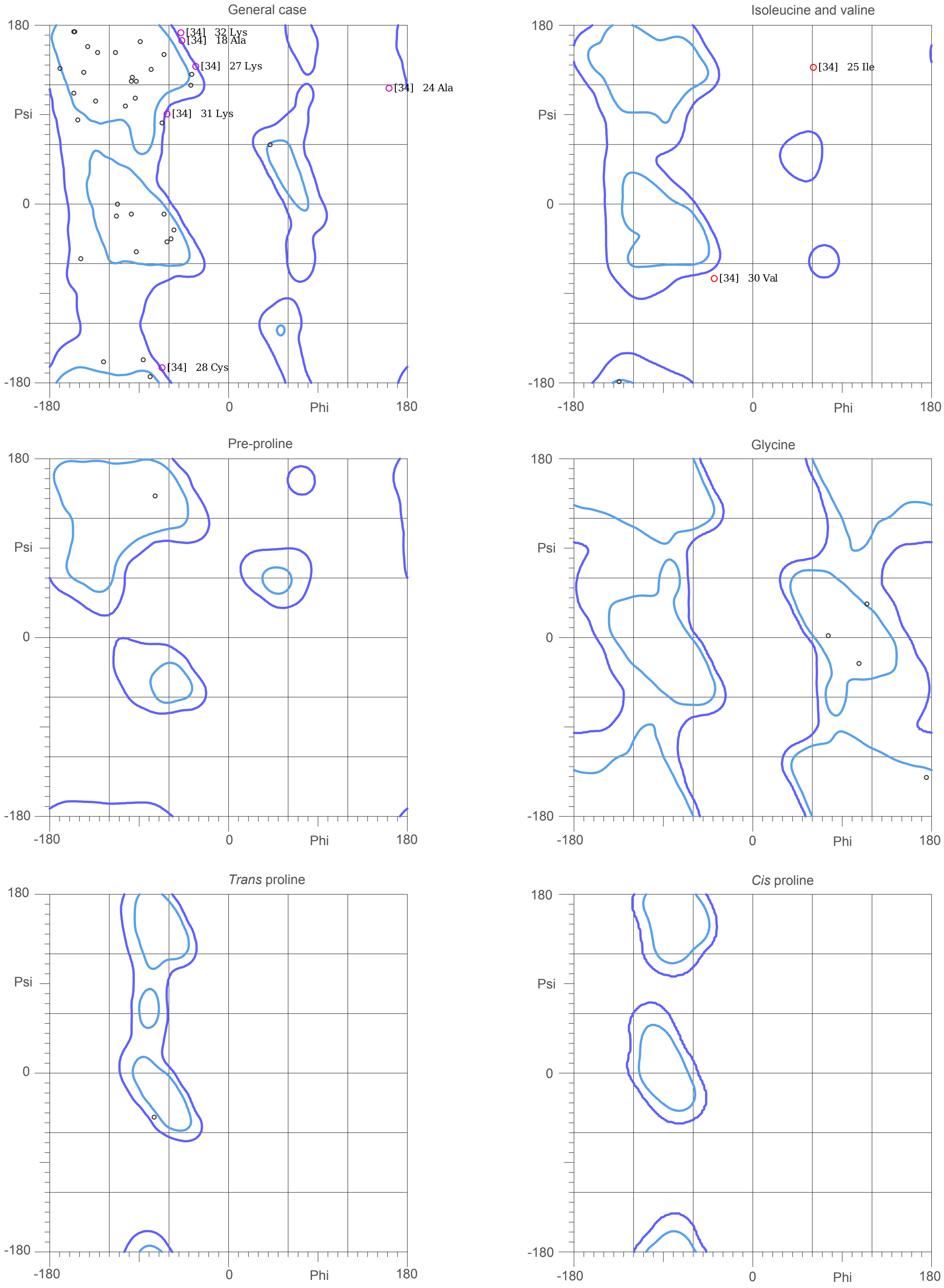

63.3% (31/49) of all residues were in favored (98%) regions.
83.7% (41/49) of all residues were in allowed (>99.8%) regions.

There were 8 outliers (phi, psi):
[34] 18 Ala (-47.1, 165.4)
[34] 24 Ala (162.2, 117.5)
[34] 25 Ile (61.1, 138.2)
[34] 27 Lys (-33.1, 140.0)
[34] 28 Cys (-67.1, -165.6)
[34] 30 Val (-39.4, -75.8)
[34] 31 Lys (-62.0, 91.9)
[34] 32 Lys (-48.6, 173.6)

MolProbity Ramachandran analysis

1afp_trimmedH.pdb, model 35

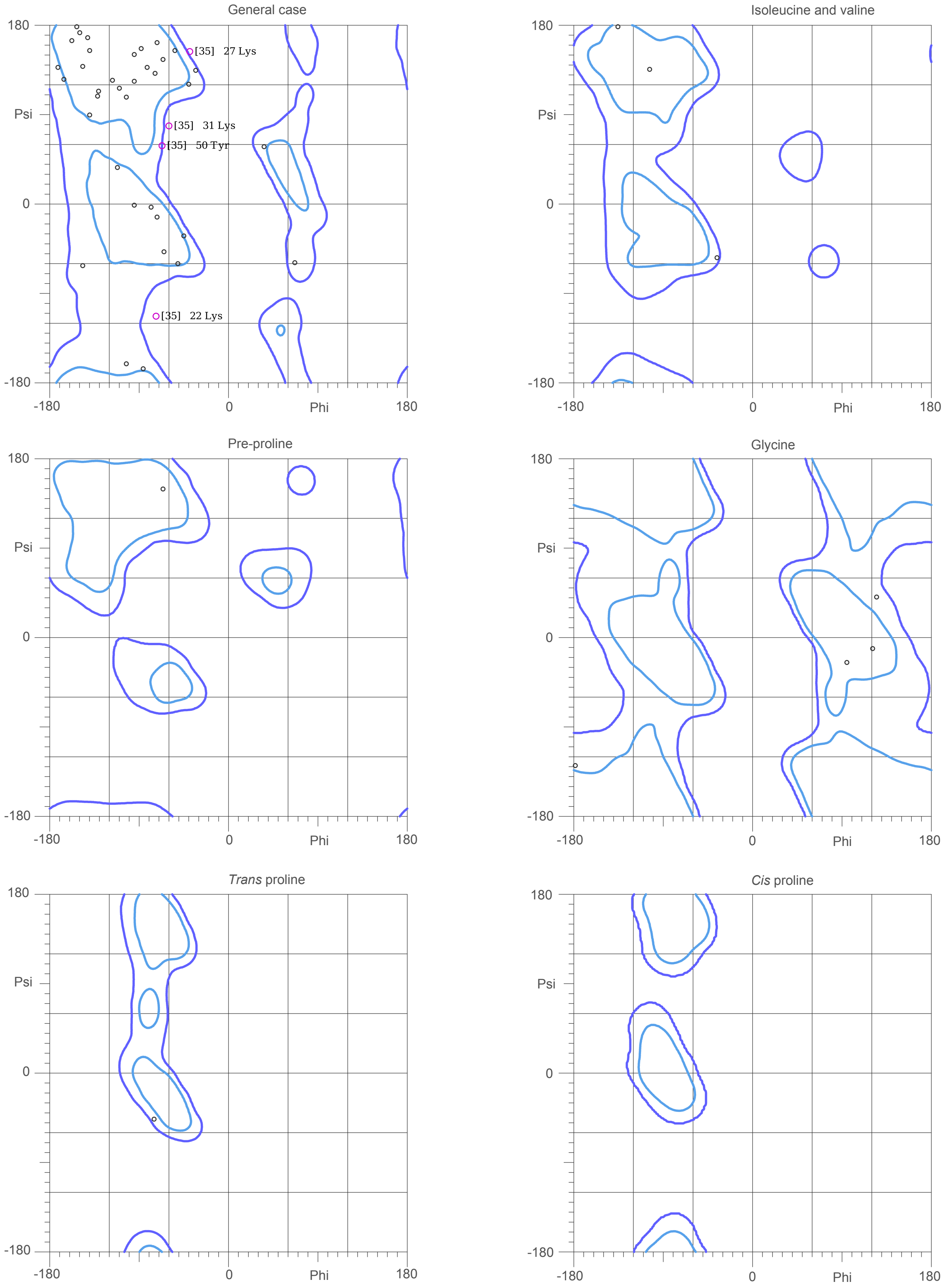

65.3% (32/49) of all residues were in favored (98%) regions.
91.8% (45/49) of all residues were in allowed (>99.8%) regions.

There were 4 outliers (phi, psi):
[35] 22 Lys (-73.8, -113.4)
[35] 27 Lys (-39.1, 155.0)
[35] 31 Lys (-60.4, 79.6)
[35] 50 Tyr (-67.1, 59.7)

MolProbity Ramachandran analysis

1afp_trimmedH.pdb, model 36

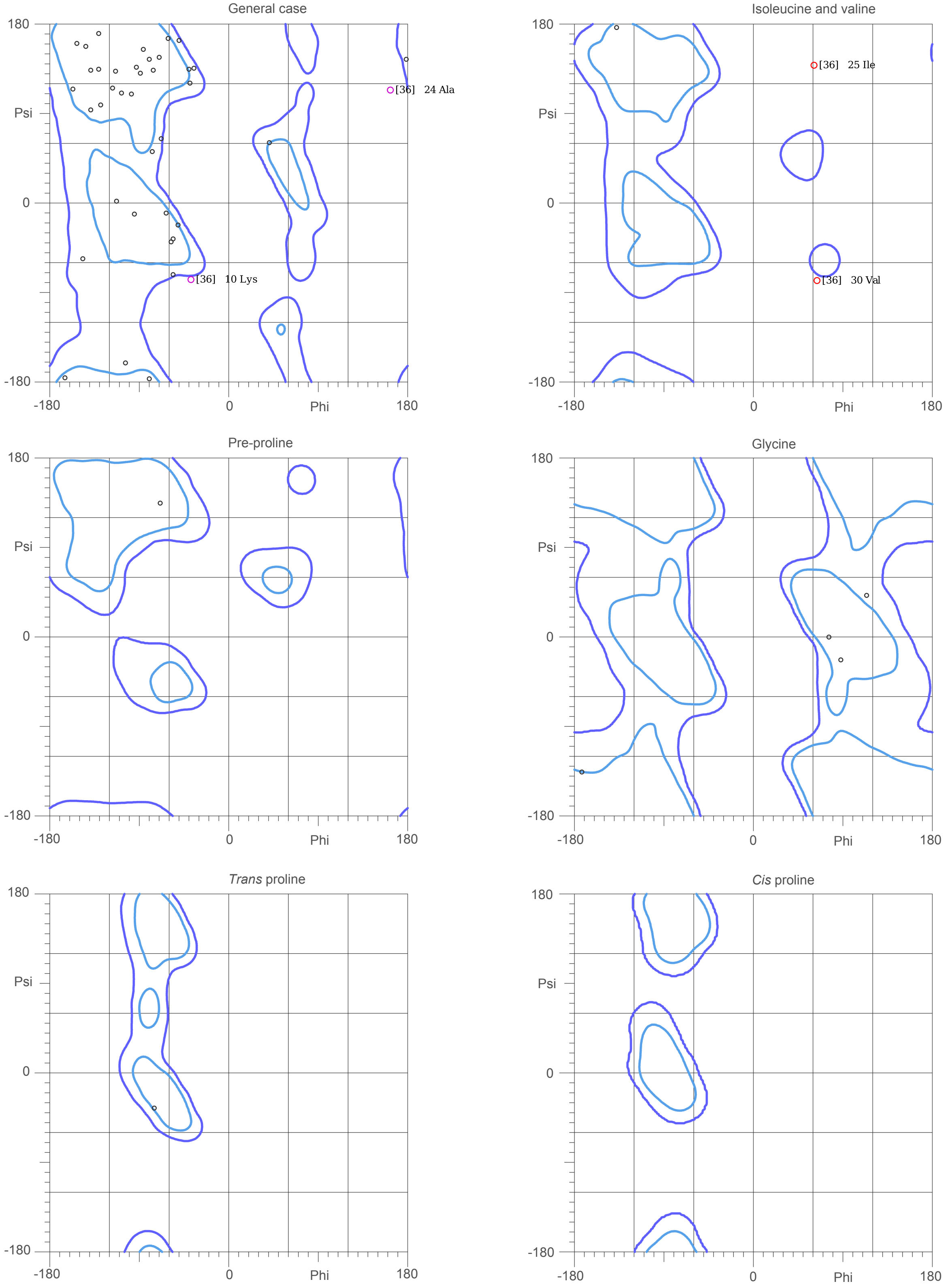

67.3% (33/49) of all residues were in favored (98%) regions.
91.8% (45/49) of all residues were in allowed (>99.8%) regions.

There were 4 outliers (phi, psi):
- [36] 10 Lys (-38.4, -77.5)
- [36] 24 Ala (163.2, 114.4)
- [36] 25 Ile (61.2, 139.9)
- [36] 30 Val (64.1, -78.4)

MolProbity Ramachandran analysis

1afp_trimmedH.pdb, model 37

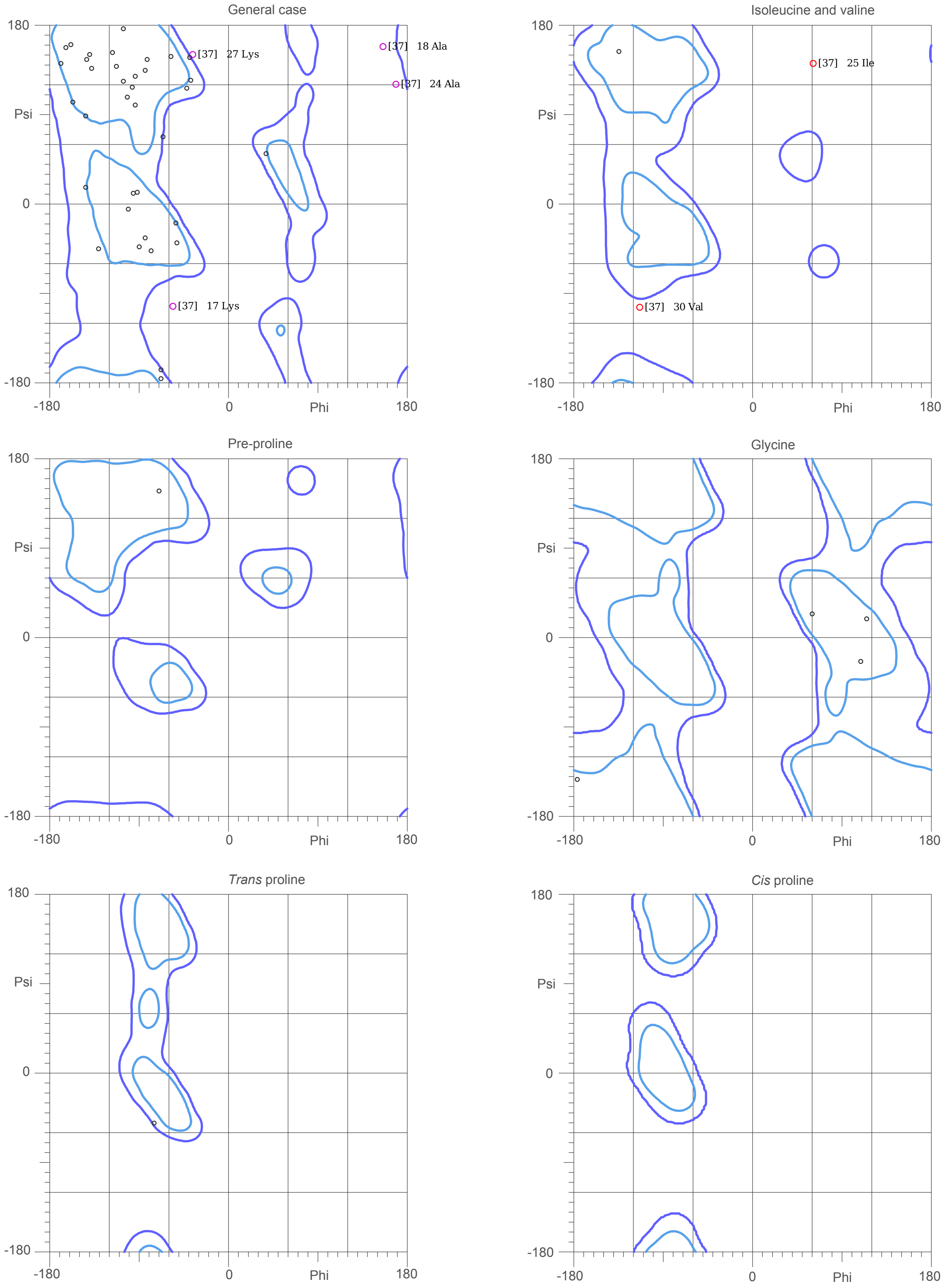

67.3% (33/49) of all residues were in favored (98%) regions.
87.8% (43/49) of all residues were in allowed (>99.8%) regions.

There were 6 outliers (phi, psi):
[37] 17 Lys (-56.6, -103.1)
[37] 18 Ala (156.8, 159.1)
[37] 24 Ala (169.7, 121.9)
[37] 25 Ile (61.2, 142.3)
[37] 27 Lys (-36.4, 151.0)
[37] 30 Val (-114.9, -104.6)

MolProbity Ramachandran analysis

1afp_trimmedH.pdb, model 38

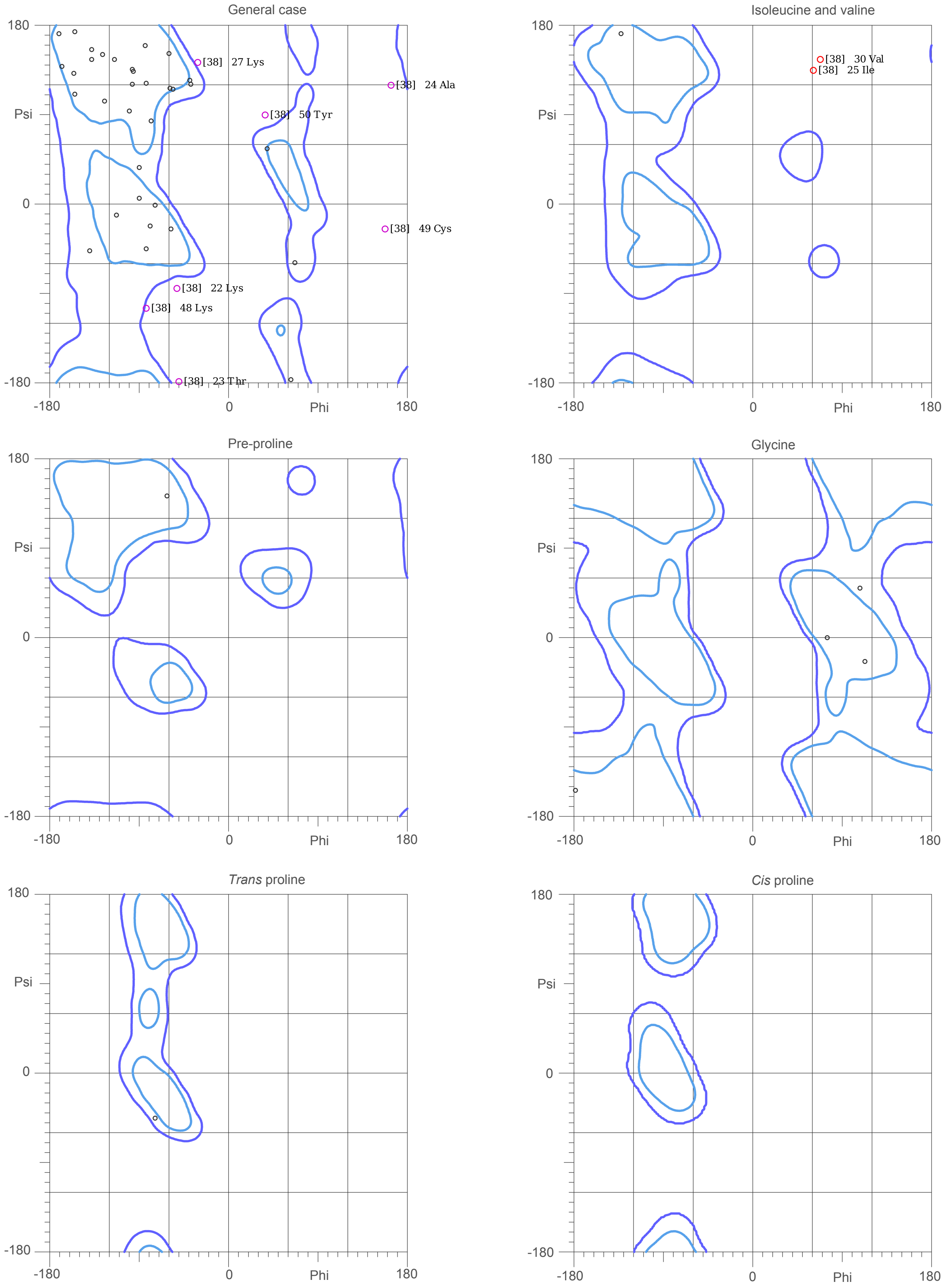

65.3% (32/49) of all residues were in favored (98%) regions.
81.6% (40/49) of all residues were in allowed (>99.8%) regions.

There were 9 outliers (phi, psi):

[38] 22 Lys (-52.1, -85.8)
[38] 23 Thr (-50.4, -179.4)
[38] 24 Ala (164.9, 120.1)
[38] 25 Ile (61.5, 135.2)
[38] 27 Lys (-31.5, 143.4)
[38] 30 Val (68.6, 146.3)
[38] 48 Lys (-83.5, -105.8)
[38] 49 Cys (158.2, -25.0)
[38] 50 Tyr (37.5, 90.6)

http://kinemage.biochem.duke.edu

Lovell, Davis, et al. Proteins 50:437 (2003)

MolProbity Ramachandran analysis

1afp_trimmedH.pdb, model 39

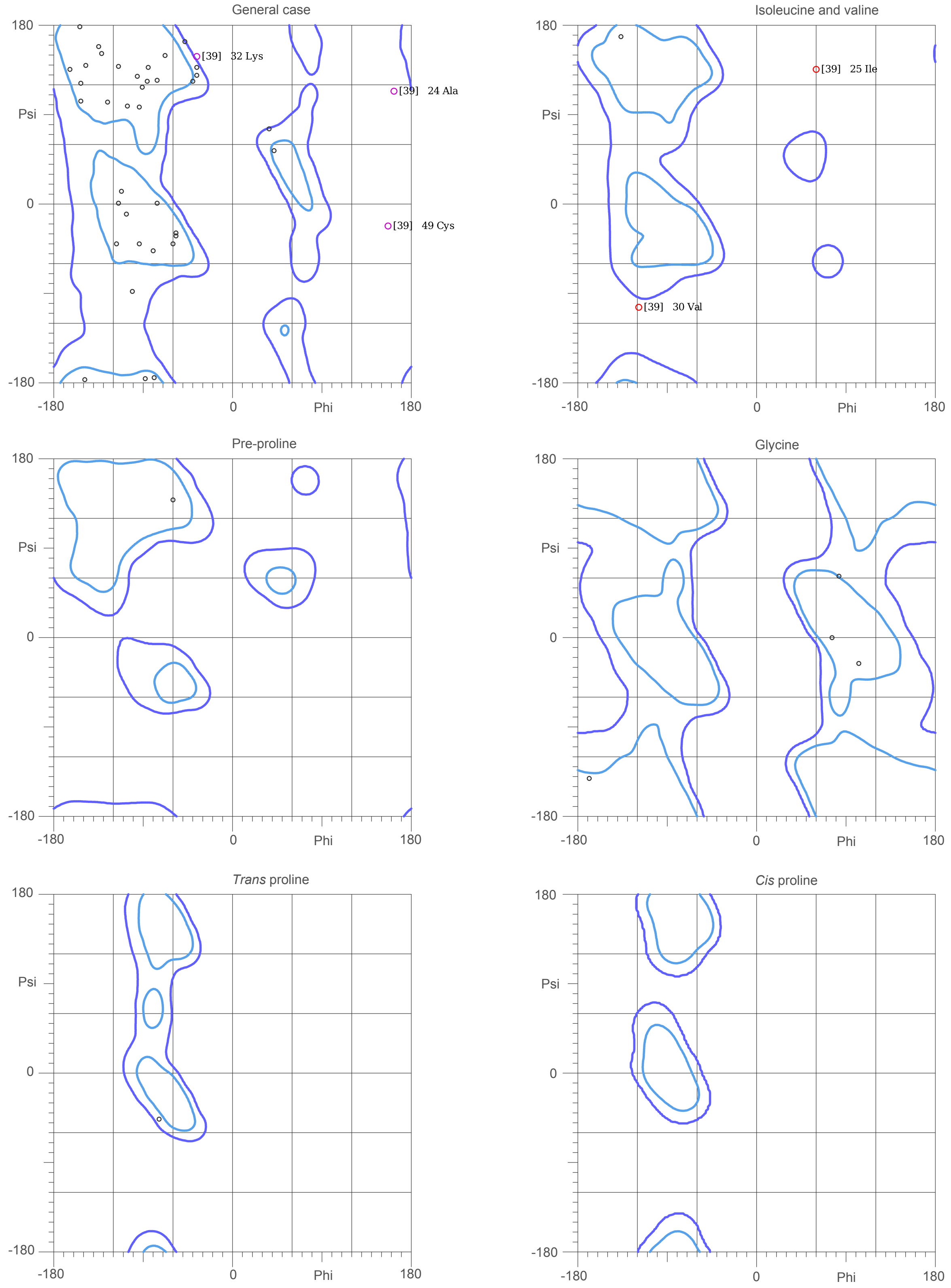

73.5% (36/49) of all residues were in favored (98%) regions.
89.8% (44/49) of all residues were in allowed (>99.8%) regions.

There were 5 outliers (phi, psi):
[39] 24 Ala (163.7, 114.7)
[39] 25 Ile (60.6, 136.2)
[39] 30 Val (-119.2, -104.6)
[39] 32 Lys (-36.6, 149.2)
[39] 49 Cys (157.3, -22.7)

MolProbity Ramachandran analysis

1afp_trimmedH.pdb, model 40

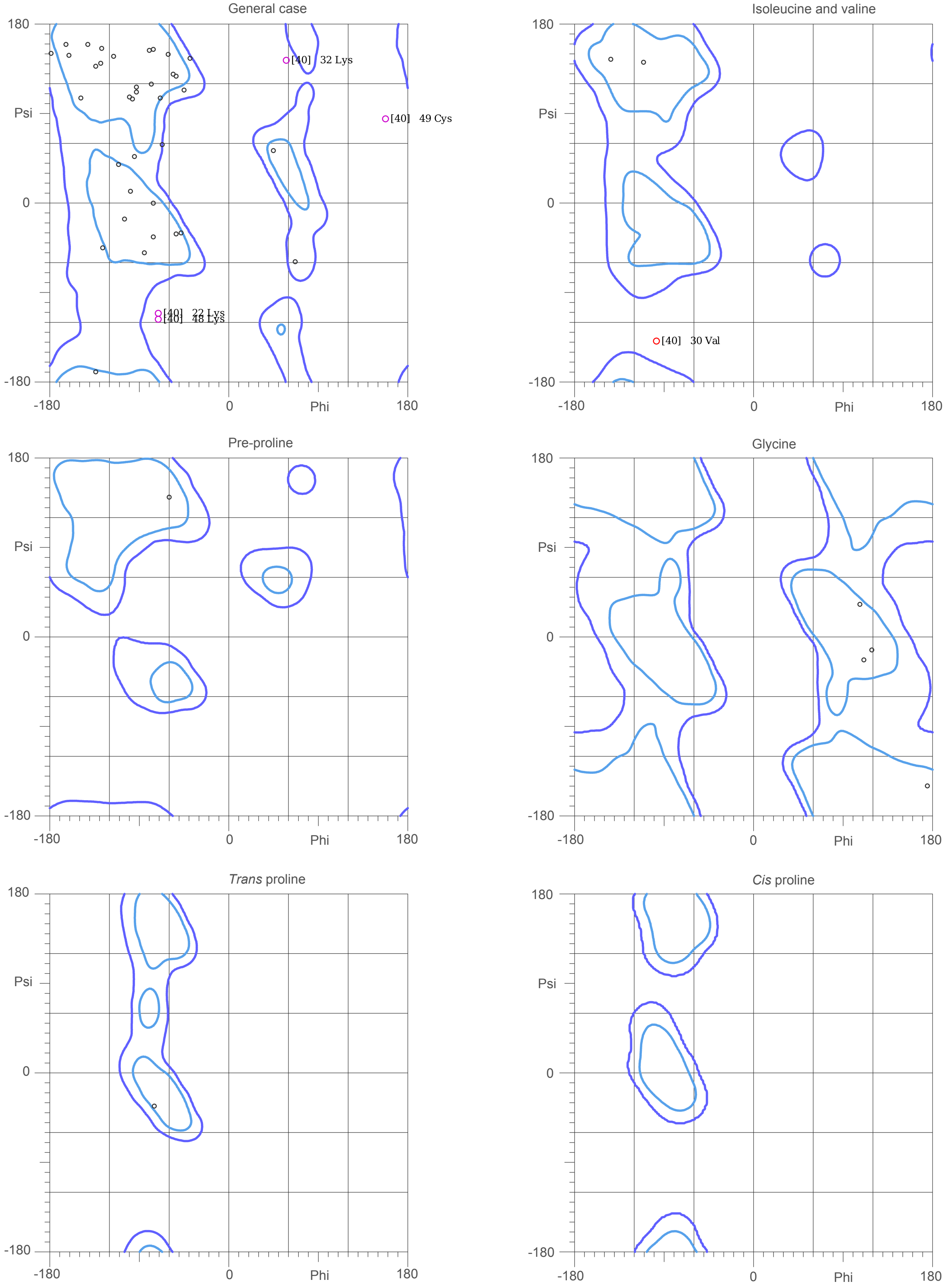

75.5% (37/49) of all residues were in favored (98%) regions.
89.8% (44/49) of all residues were in allowed (>99.8%) regions.

There were 5 outliers (phi, psi):
[40] 22 Lys (-71.0, -111.9)
[40] 30 Val (-98.2, -139.8)
[40] 32 Lys (58.6, 144.4)
[40] 48 Lys (-71.2, -117.0)
[40] 49 Cys (158.9, 85.0)
