# Supplementary material for: Small Disulfide Proteins with Antifungal Impact: NMR Experimental Structures as Compared to Models of Alphafold Versions
Source: Int J Mol Sci. 2025 Jan 31;26(3):1247. doi: 10.3390/ijms26031247 (PMC11818080; doi:10.3390/ijms26031247)
Supplement: Supplementary file 1 [file ijms-26-01247-s001.zip › Figure S7h. AF2-AFPg.pdf]

MolProbity Ramachandran analysis

AFP_0958b_unrelaxed_rank_001_alphafold2_ptm_model_5_seed_000FH.pdb, model 1

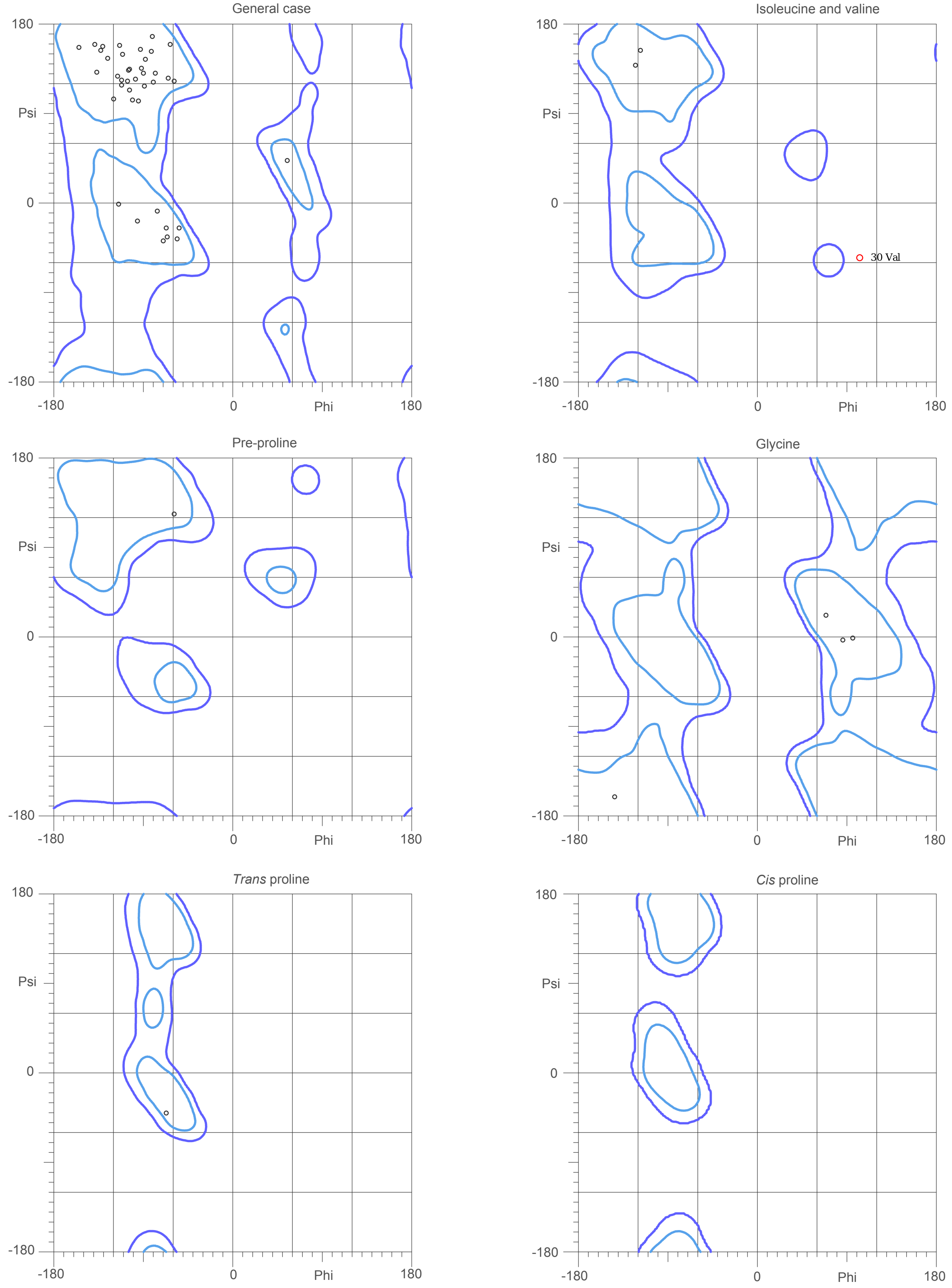

98.0% (48/49) of all residues were in favored (98%) regions.
98.0% (48/49) of all residues were in allowed (>99.8%) regions.

There were 1 outliers (phi, psi):
30 Val (103.4, -55.3)
