# Supplementary material for: Small Disulfide Proteins with Antifungal Impact: NMR Experimental Structures as Compared to Models of Alphafold Versions
Source: Int J Mol Sci. 2025 Jan 31;26(3):1247. doi: 10.3390/ijms26031247 (PMC11818080; doi:10.3390/ijms26031247)
Supplement: Supplementary file 1 [file ijms-26-01247-s001.zip › Figure S8a.AF2-PAFC.pdf]

MolProbity Ramachandran analysis

6TRM-1FH.pdb, model 1

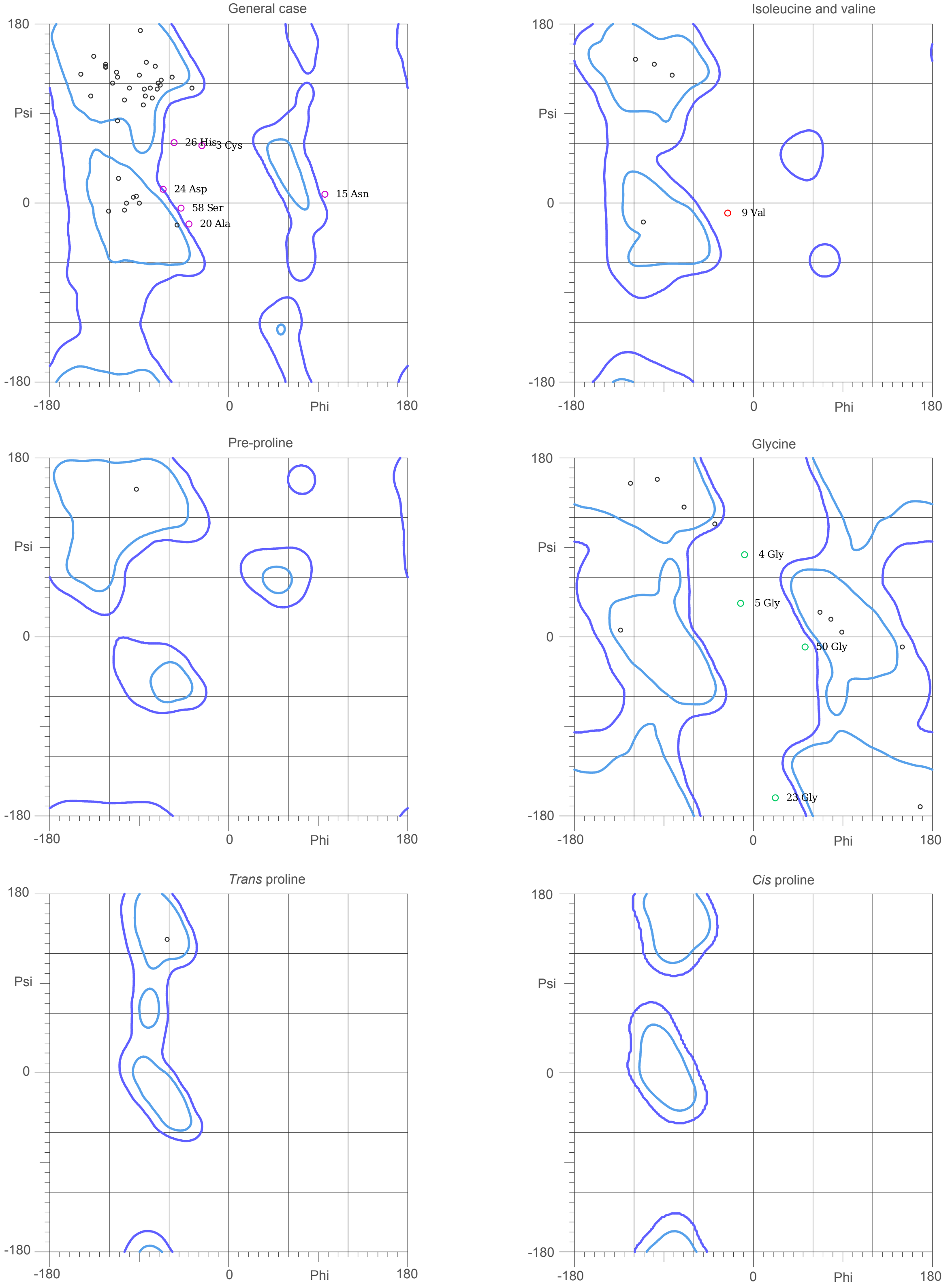

75.8% (47/62) of all residues were in favored (98%) regions.
82.3% (51/62) of all residues were in allowed (>99.8%) regions.

There were 11 outliers (phi, psi):

3 Cys (-27.5, 58.7)
4 Gly (-9.6, 83.8)
5 Gly (-13.4, 34.8)
9 Val (-26.5, -10.4)
15 Asn (97.8, 9.7)
20 Ala (-40.9, -21.2)
23 Gly (22.3, -162.0)
24 Asp (-66.6, 14.7)
26 His (-55.1, 61.6)
50 Gly (52.3, -10.2)
58 Ser (-48.8, -5.2)

http://kinemage.biochem.duke.edu

Lovell, Davis, et al. Proteins 50:437 (2003)
