# Supplementary material for: Small Disulfide Proteins with Antifungal Impact: NMR Experimental Structures as Compared to Models of Alphafold Versions
Source: Int J Mol Sci. 2025 Jan 31;26(3):1247. doi: 10.3390/ijms26031247 (PMC11818080; doi:10.3390/ijms26031247)
Supplement: Supplementary file 1 [file ijms-26-01247-s001.zip › Figure S8a.NMR-PAFC.pdf]

MolProbity Ramachandran analysis

6trmH.pdb, all models

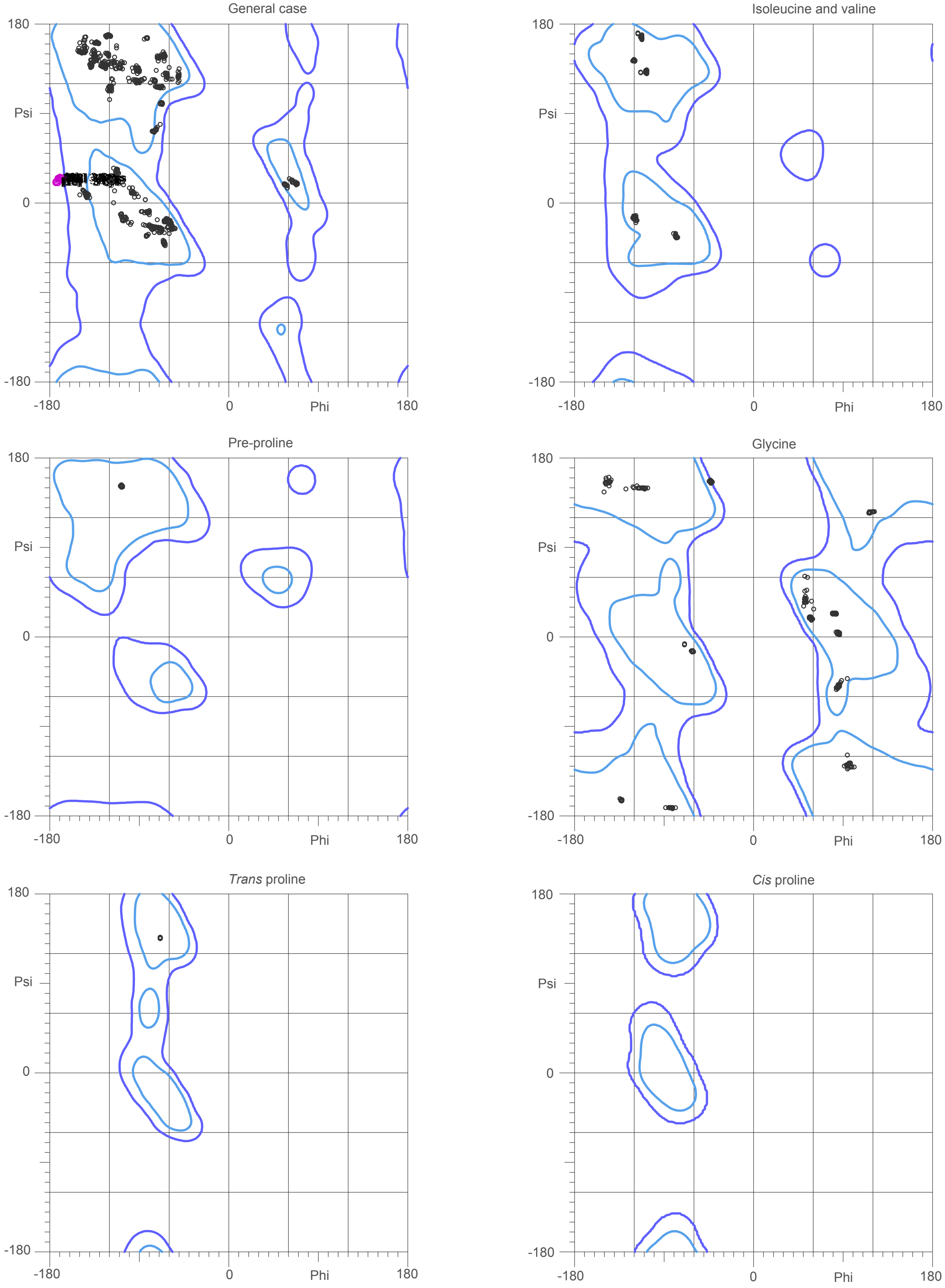

92.8% (1151/1240) of all residues were in favored (98%) regions.
98.4% (1220/1240) of all residues were in allowed (>99.8%) regions.

There were 20 outliers (phi, psi):

[1] 3 Cys (-170.4, 26.0)
[2] 3 Cys (-168.8, 25.7)
[3] 3 Cys (-170.0, 25.8)
[4] 3 Cys (-172.7, 25.4)
[5] 3 Cys (-169.1, 25.5)
[6] 3 Cys (-170.8, 26.5)
[7] 3 Cys (-168.7, 25.3)
[8] 3 Cys (-171.6, 22.4)
[9] 3 Cys (-169.4, 25.9)
[10] 3 Cys (-168.5, 25.3)
[11] 3 Cys (-167.1, 24.8)
[12] 3 Cys (-167.7, 25.0)
[13] 3 Cys (-171.7, 25.0)
[14] 3 Cys (-172.7, 25.0)
[15] 3 Cys (-169.4, 25.7)
[16] 3 Cys (-170.0, 25.7)
[17] 3 Cys (-174.2, 21.2)
[18] 3 Cys (-173.0, 20.7)
[19] 3 Cys (-173.8, 21.9)
[20] 3 Cys (-174.3, 20.8)

http://kinemage.biochem.duke.edu

Lovell, Davis, et al. Proteins 50:437 (2003)

MolProbity Ramachandran analysis

6trmH.pdb, model 1

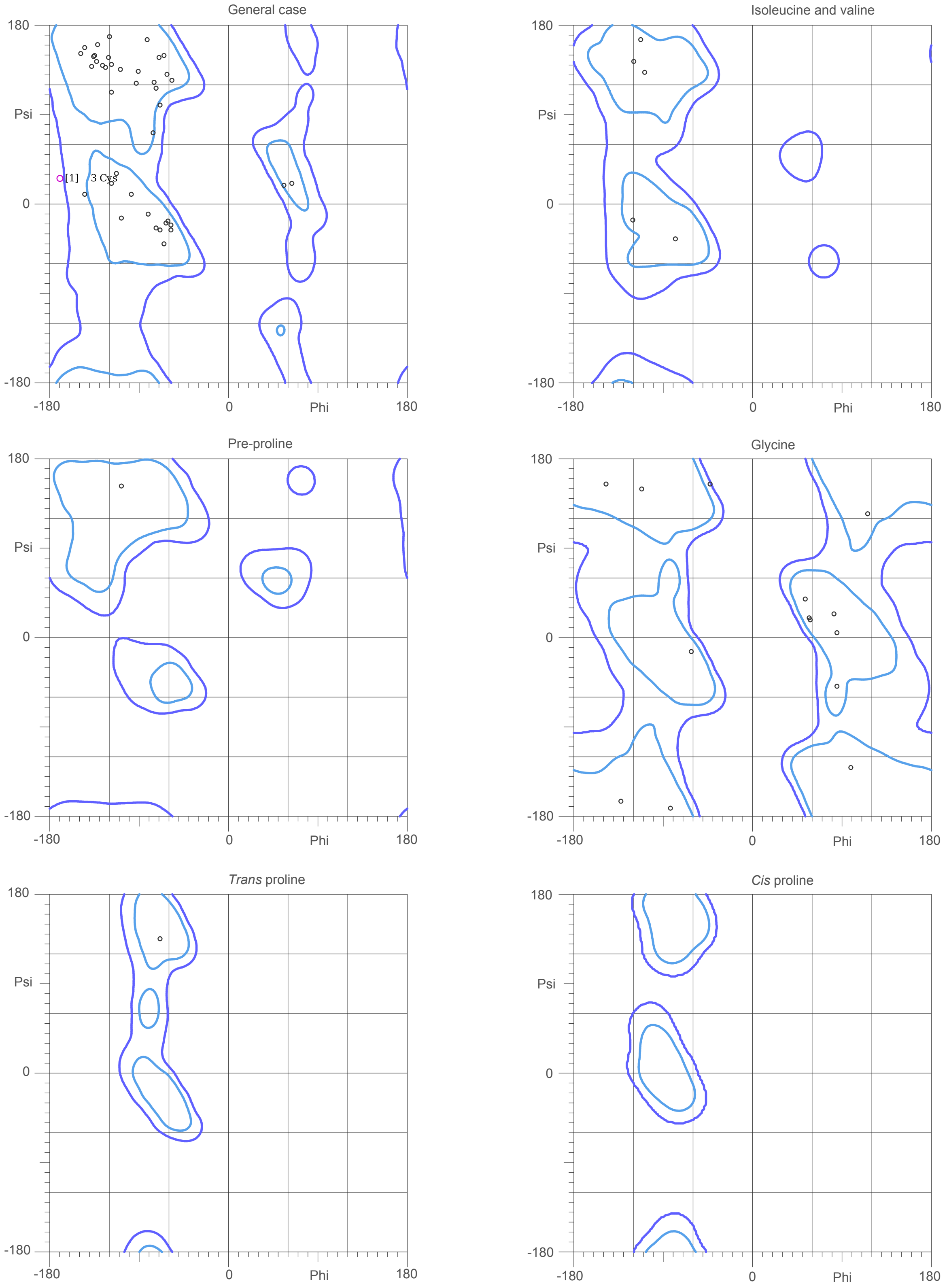

93.5% (58/62) of all residues were in favored (98%) regions.
98.4% (61/62) of all residues were in allowed (>99.8%) regions.

There were 1 outliers (phi, psi):
[1] 3 Cys (-170.4, 26.0)

MolProbity Ramachandran analysis

6trmH.pdb, model 2

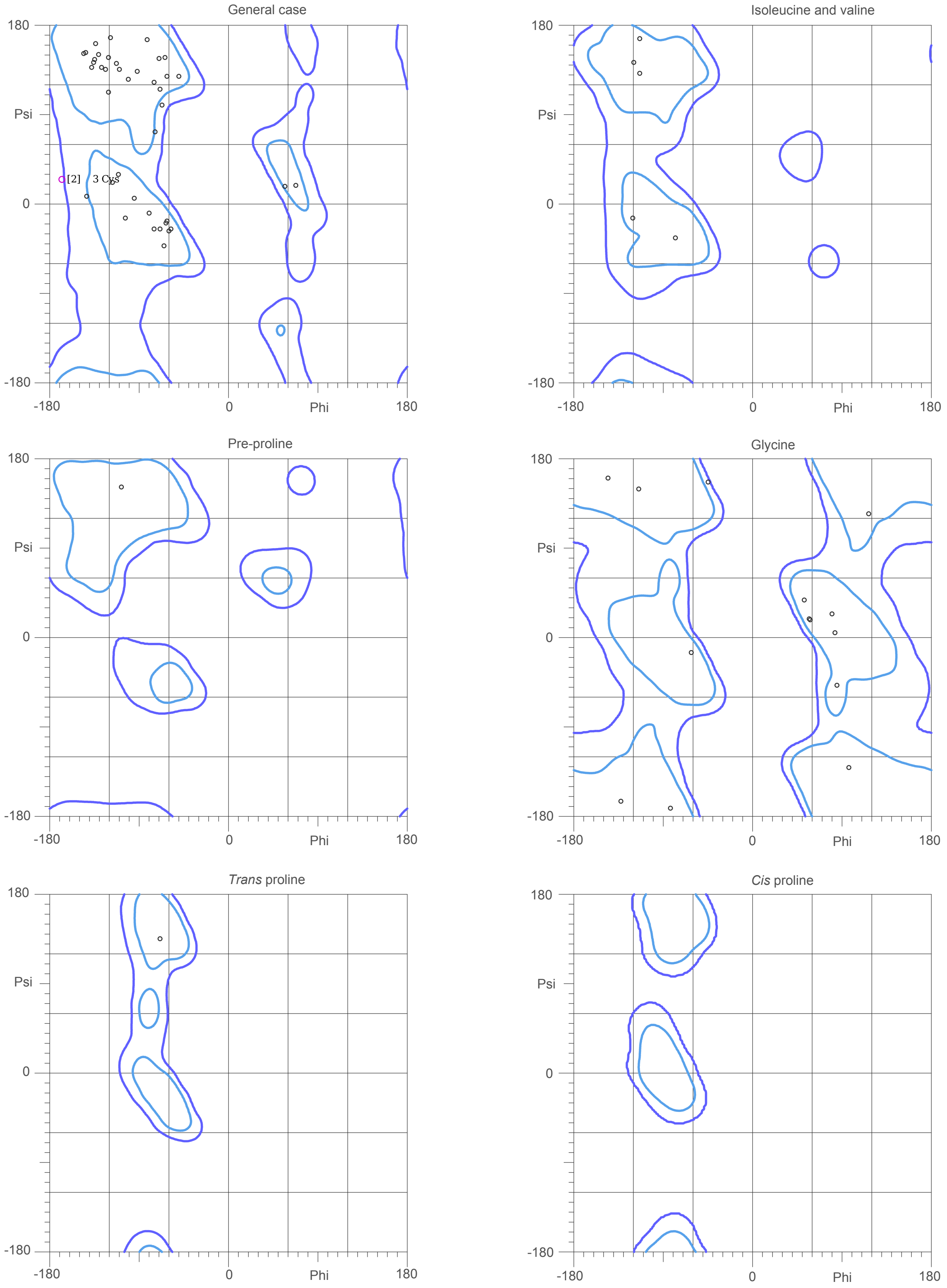

91.9% (57/62) of all residues were in favored (98%) regions.
98.4% (61/62) of all residues were in allowed (>99.8%) regions.

There were 1 outliers (phi, psi):
[2] 3 Cys (-168.8, 25.7)

MolProbity Ramachandran analysis

6trmH.pdb, model 3

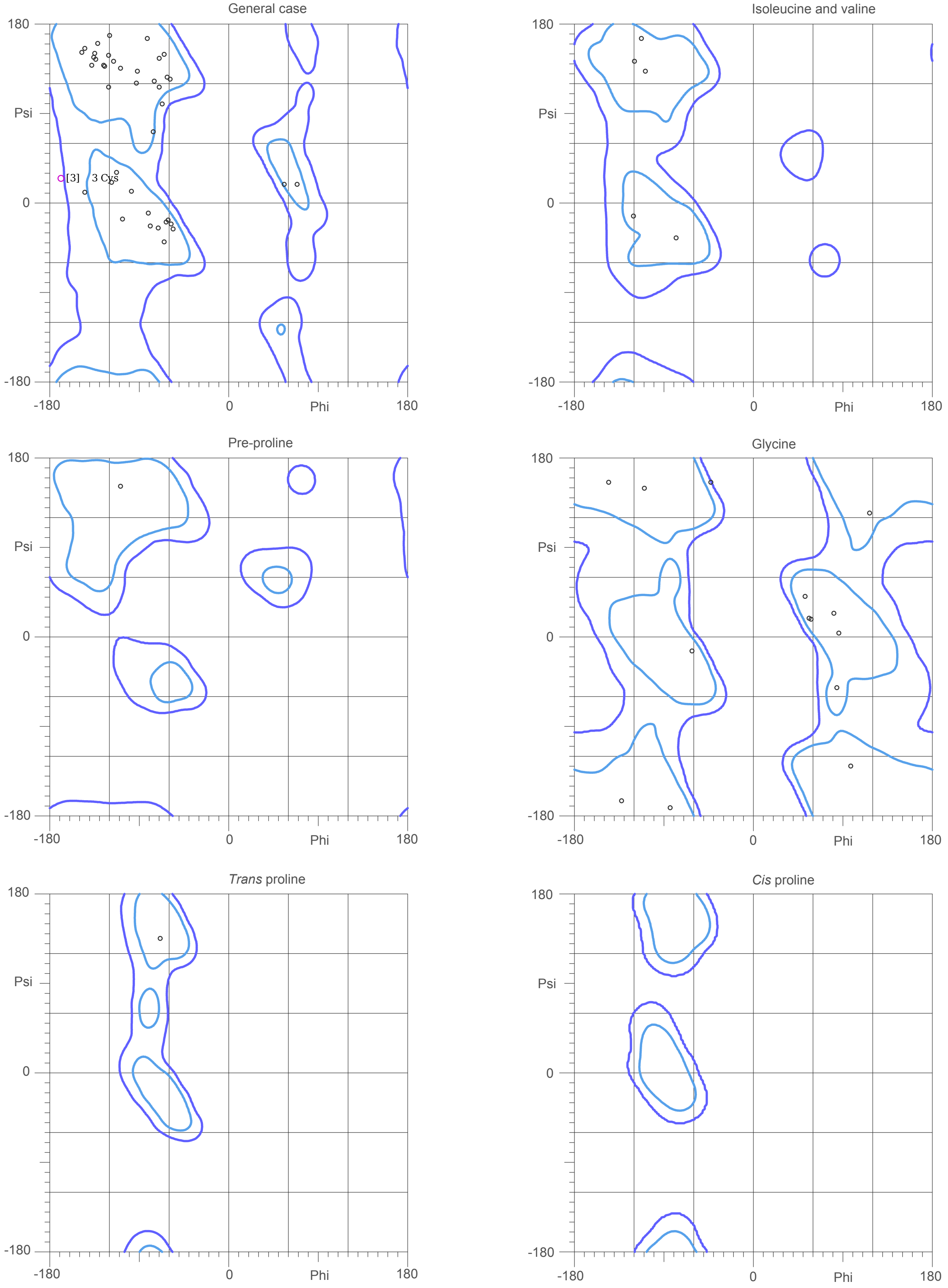

93.5% (58/62) of all residues were in favored (98%) regions.
98.4% (61/62) of all residues were in allowed (>99.8%) regions.

There were 1 outliers (phi, psi):
[3] 3 Cys (-170.0, 25.8)

Lovell, Davis, et al. Proteins 50:437 (2003)

MolProbity Ramachandran analysis

6trmH.pdb, model 4

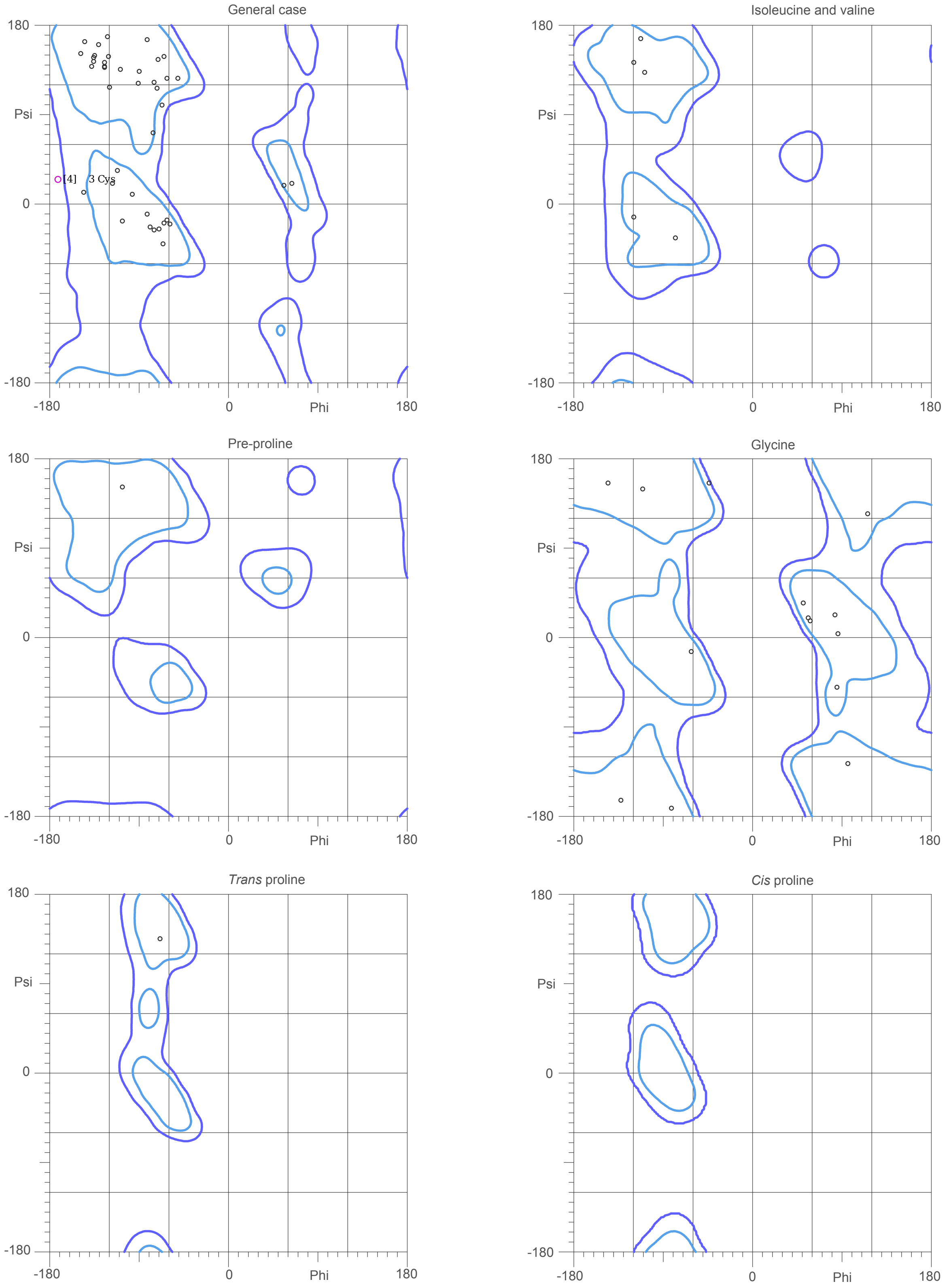

93.5% (58/62) of all residues were in favored (98%) regions.
98.4% (61/62) of all residues were in allowed (>99.8%) regions.

There were 1 outliers (phi, psi):
[4] 3 Cys (-172.7, 25.4)

MolProbity Ramachandran analysis

6trmH.pdb, model 5

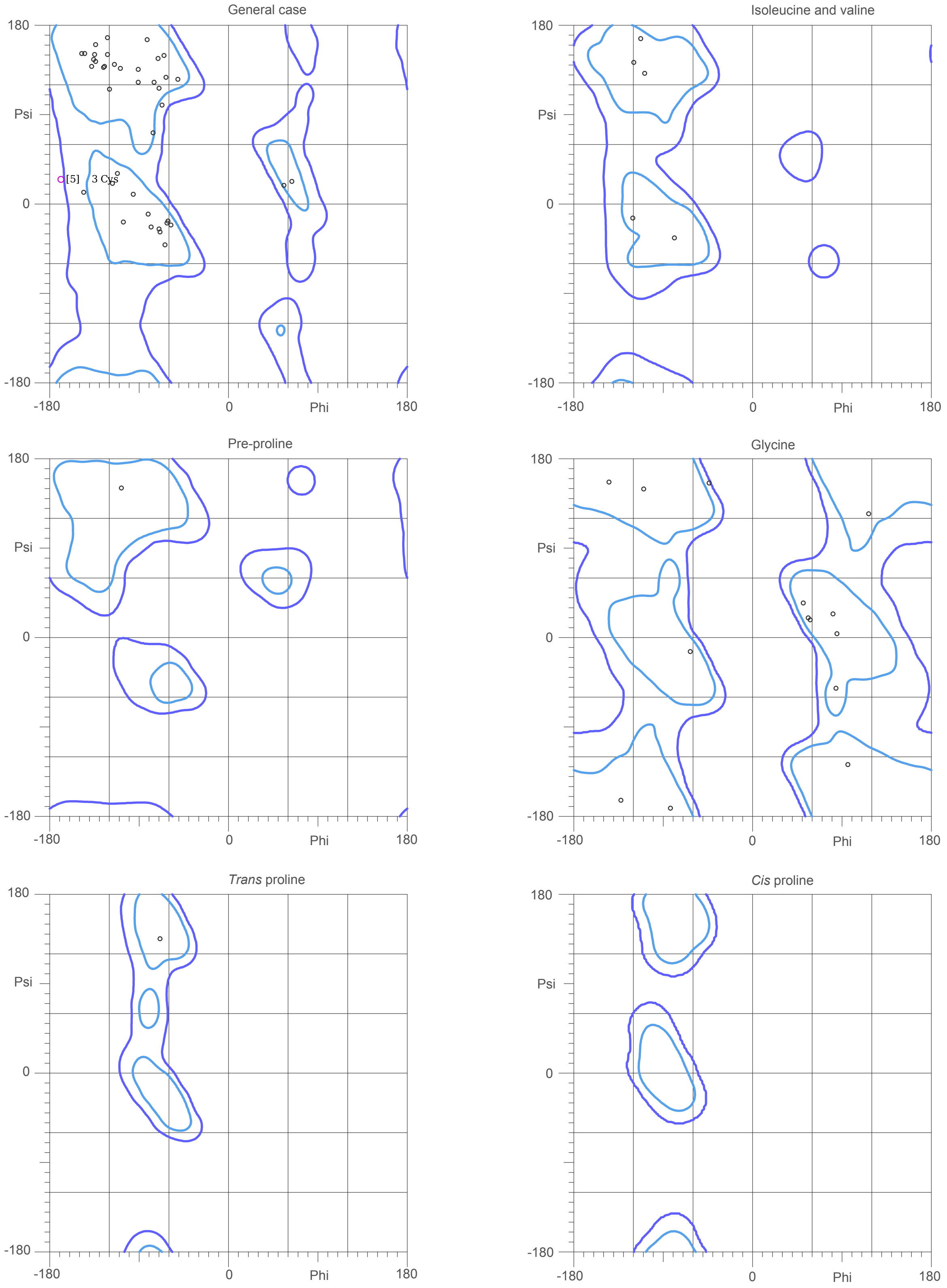

93.5% (58/62) of all residues were in favored (98%) regions.
98.4% (61/62) of all residues were in allowed (>99.8%) regions.

There were 1 outliers (phi, psi):
[5] 3 Cys (-169.1, 25.5)

MolProbity Ramachandran analysis

6trmH.pdb, model 6

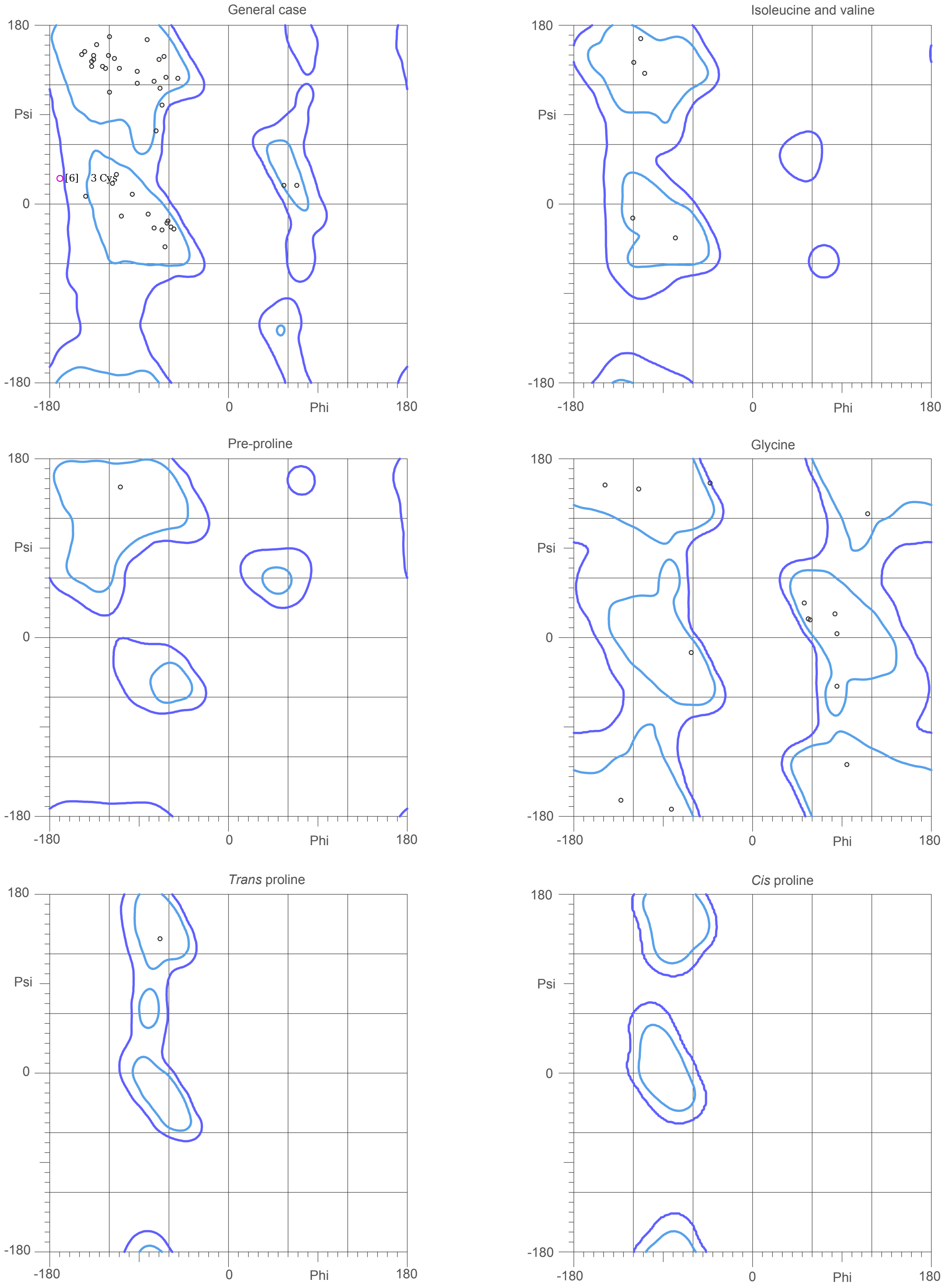

91.9% (57/62) of all residues were in favored (98%) regions.
98.4% (61/62) of all residues were in allowed (>99.8%) regions.

There were 1 outliers (phi, psi):
[6] 3 Cys (-170.8, 26.5)

MolProbity Ramachandran analysis

6trmH.pdb, model 7

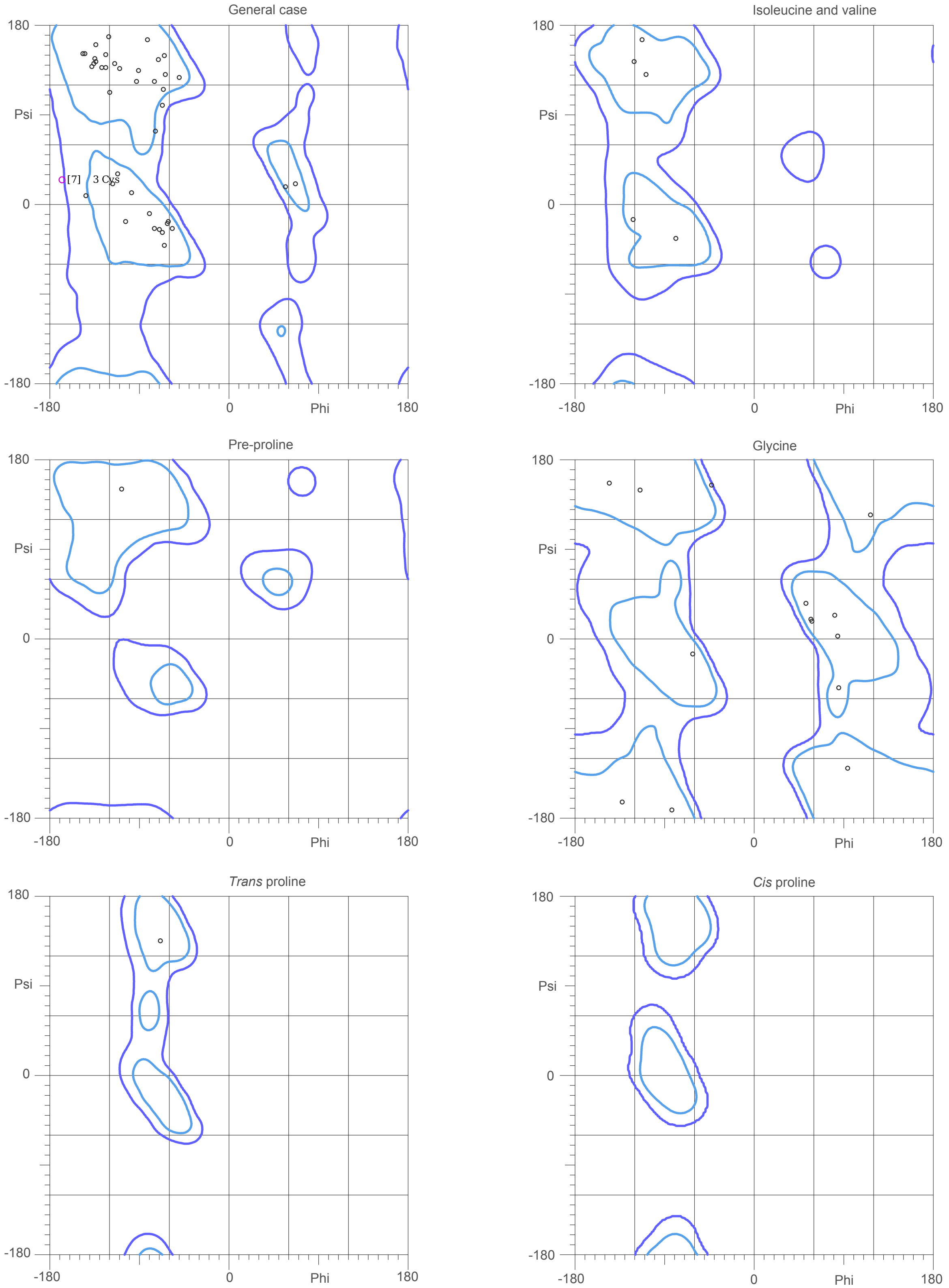

91.9% (57/62) of all residues were in favored (98%) regions.
98.4% (61/62) of all residues were in allowed (>99.8%) regions.

There were 1 outliers (phi, psi):
[7] 3 Cys (-168.7, 25.3)

MolProbity Ramachandran analysis

6trmH.pdb, model 8

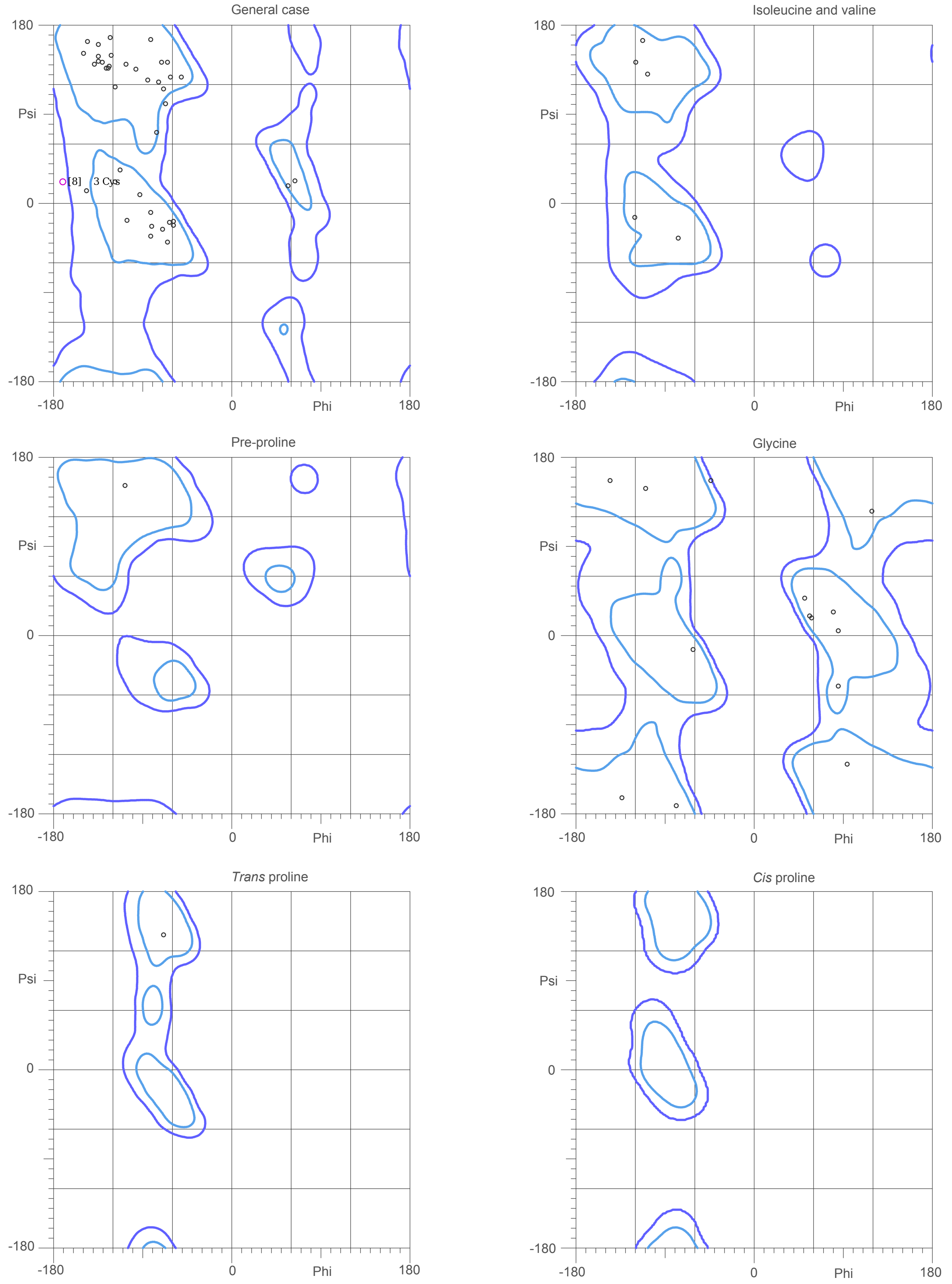

93.5% (58/62) of all residues were in favored (98%) regions.
98.4% (61/62) of all residues were in allowed (>99.8%) regions.

There were 1 outliers (phi, psi):
[8] 3 Cys (-171.6, 22.4)

MolProbity Ramachandran analysis

6trmH.pdb, model 9

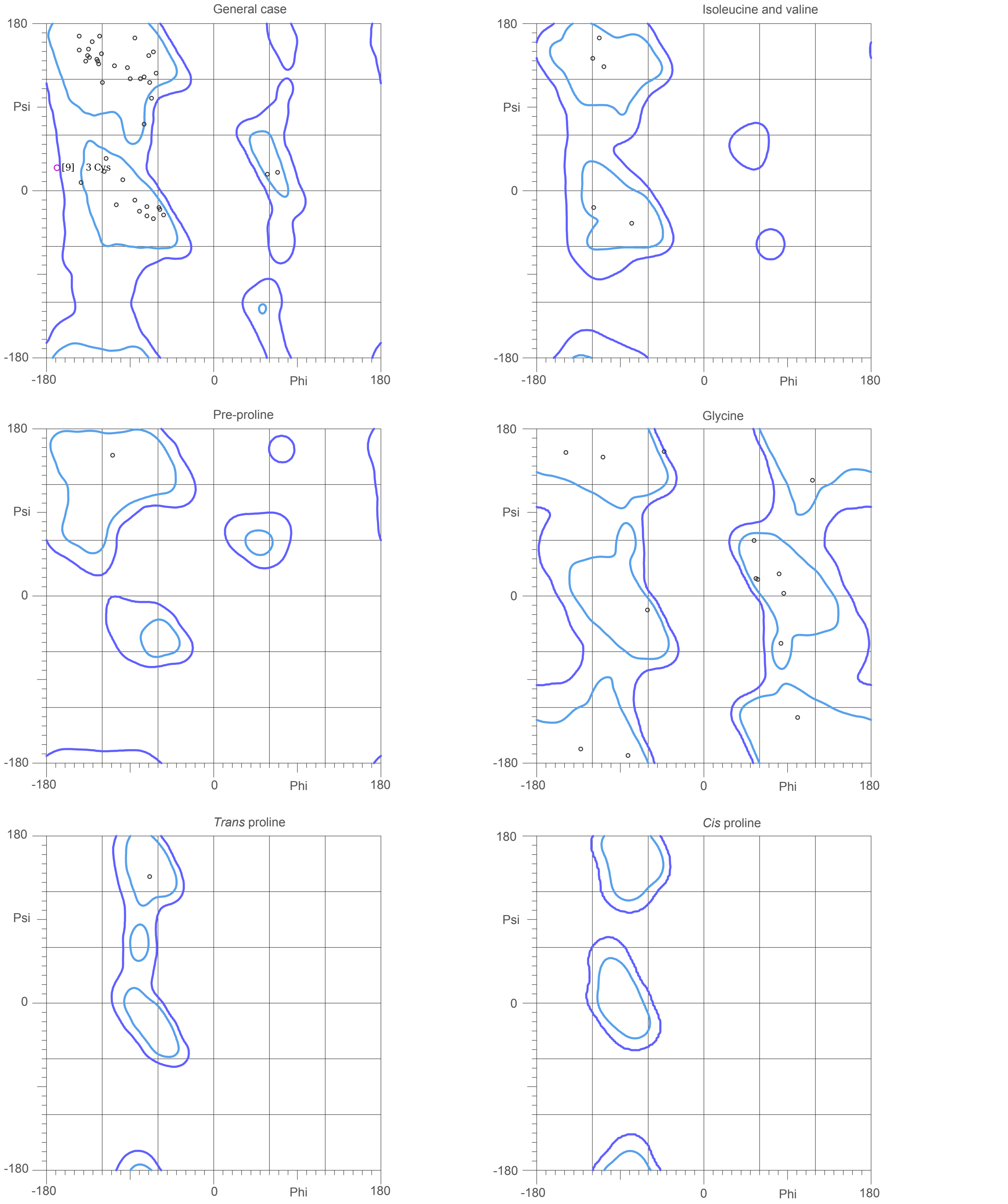

93.5% (58/62) of all residues were in favored (98%) regions.
98.4% (61/62) of all residues were in allowed (>99.8%) regions.

There were 1 outliers (phi, psi):
[9] 3 Cys (-169.4, 25.9)

MolProbity Ramachandran analysis

6trmH.pdb, model 10

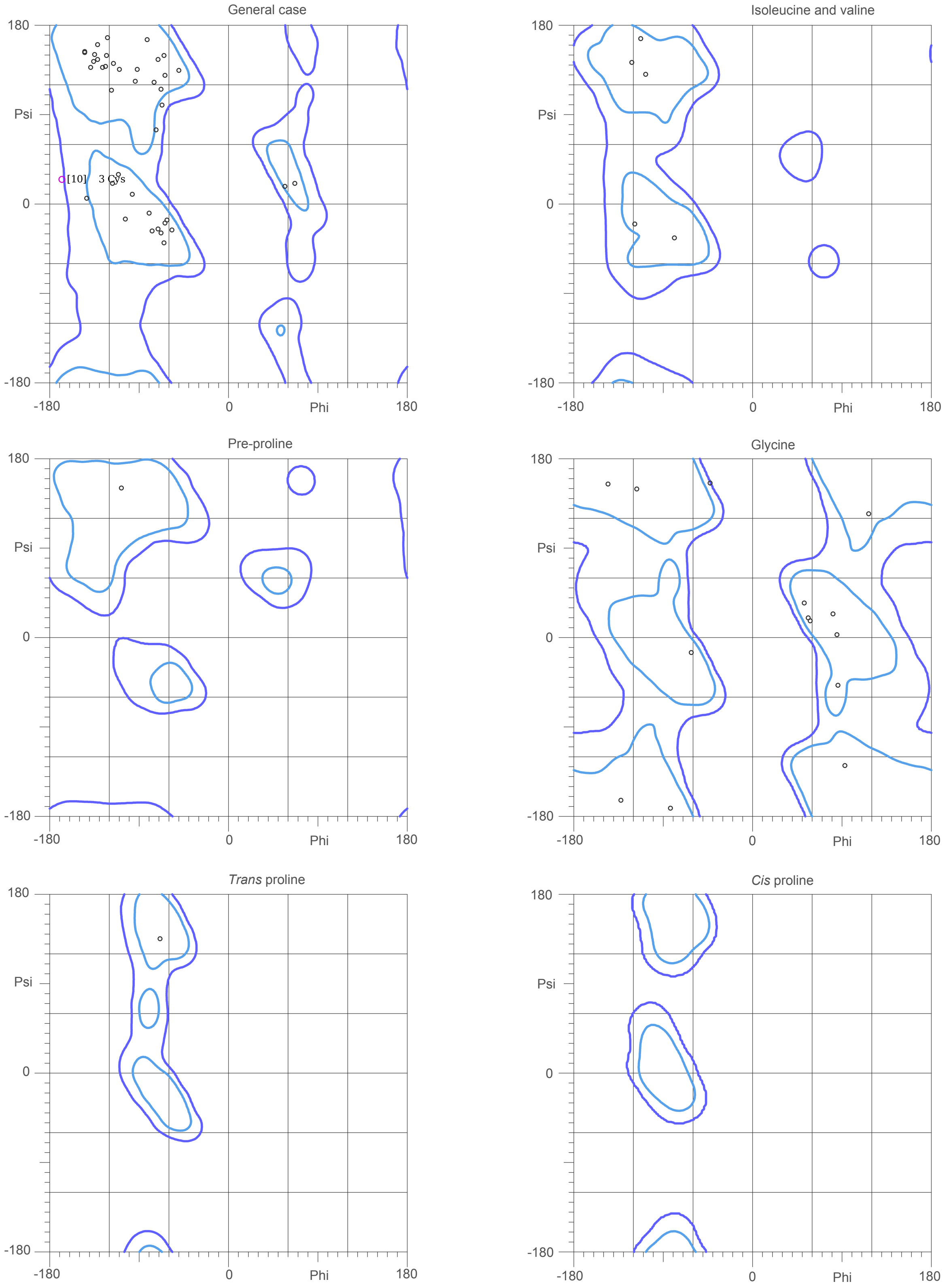

91.9% (57/62) of all residues were in favored (98%) regions.
98.4% (61/62) of all residues were in allowed (>99.8%) regions.

There were 1 outliers (phi, psi):
[10] 3 Cys (-168.5, 25.3)

MolProbity Ramachandran analysis

6trmH.pdb, model 11

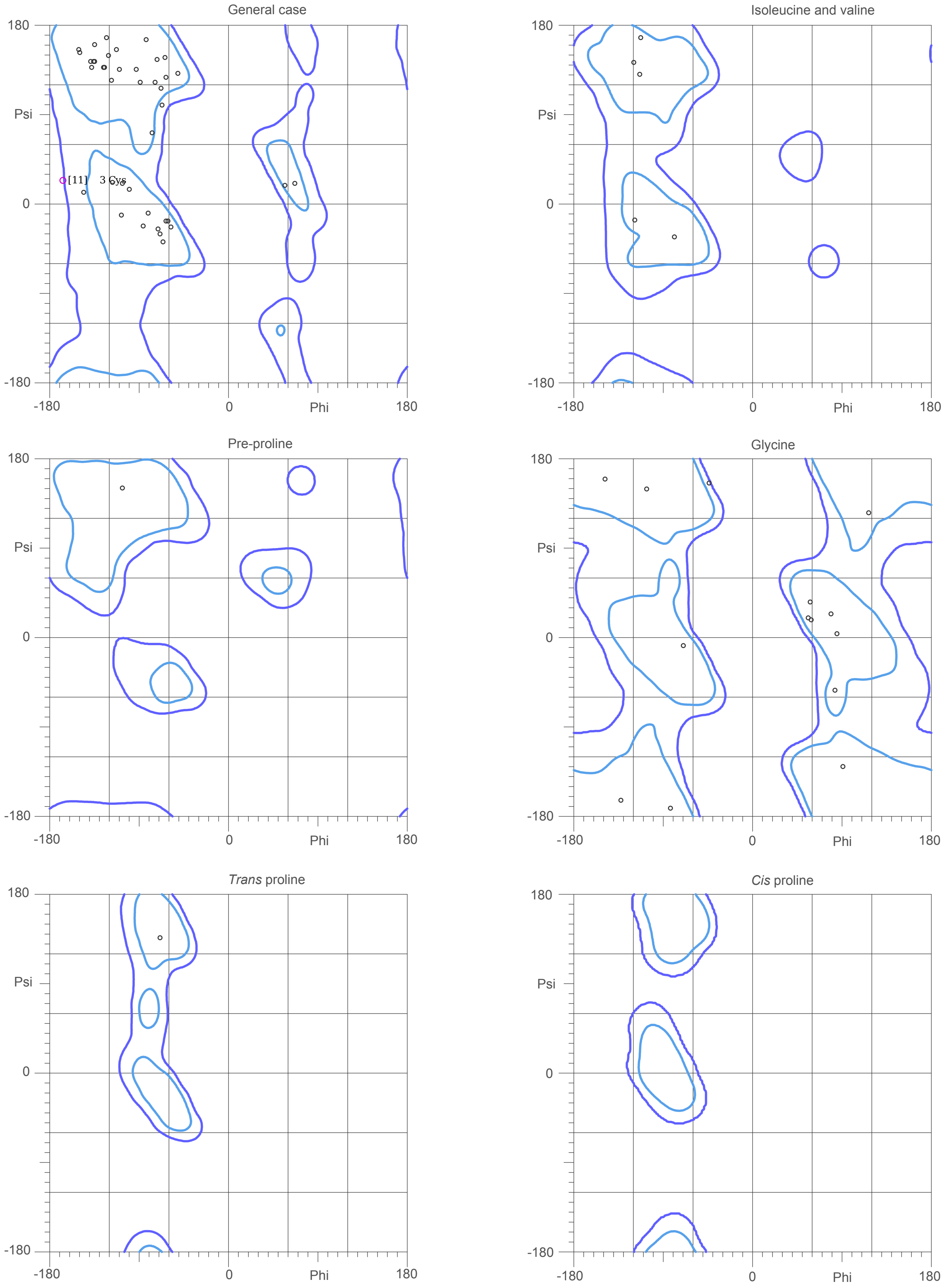

93.5% (58/62) of all residues were in favored (98%) regions.
98.4% (61/62) of all residues were in allowed (>99.8%) regions.

There were 1 outliers (phi, psi):
[11] 3 Cys (-167.1, 24.8)

MolProbity Ramachandran analysis

6trmH.pdb, model 12

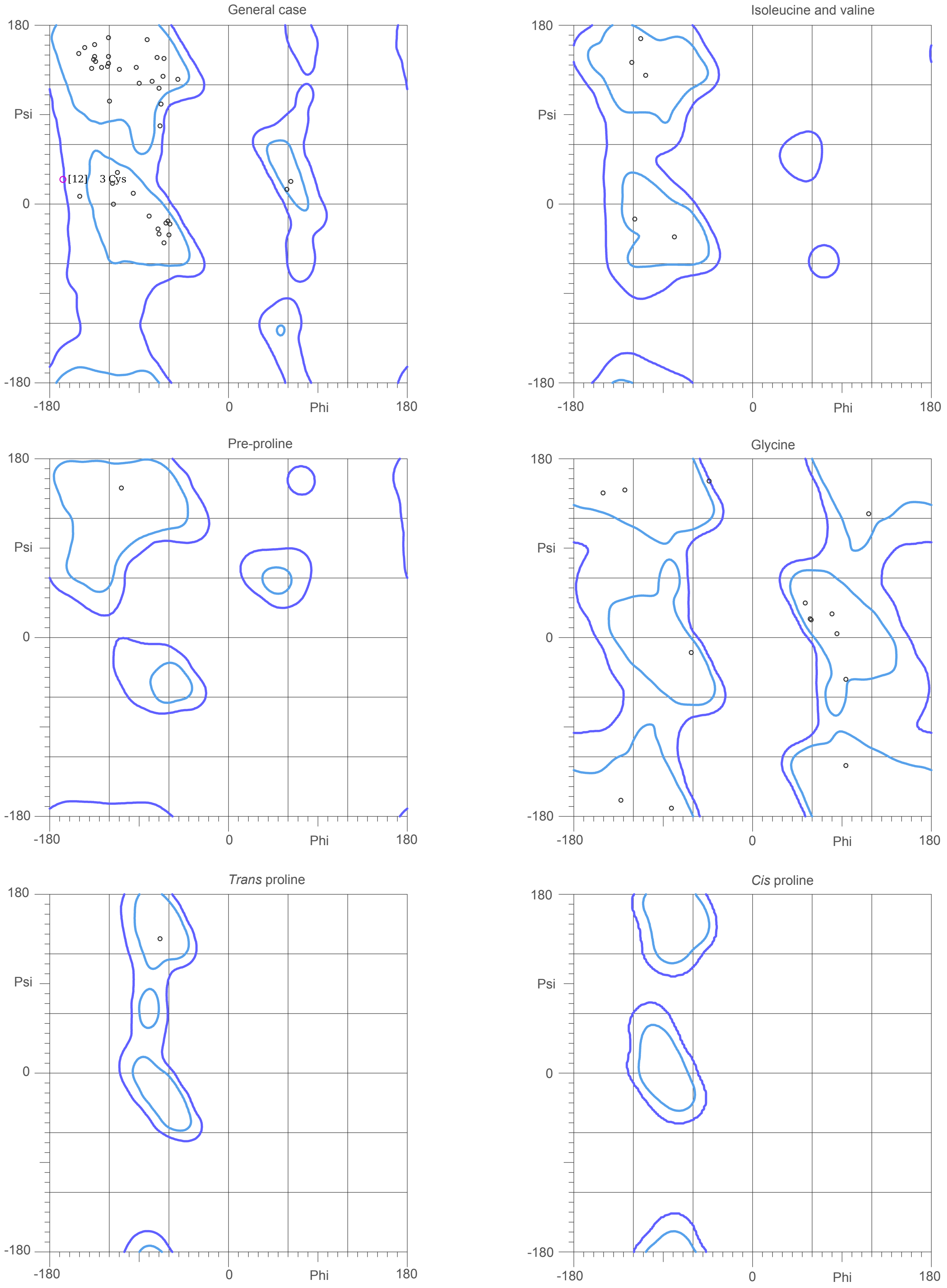

91.9% (57/62) of all residues were in favored (98%) regions.
98.4% (61/62) of all residues were in allowed (>99.8%) regions.

There were 1 outliers (phi, psi):
[12] 3 Cys (-167.7, 25.0)

MolProbity Ramachandran analysis

6trmH.pdb, model 13

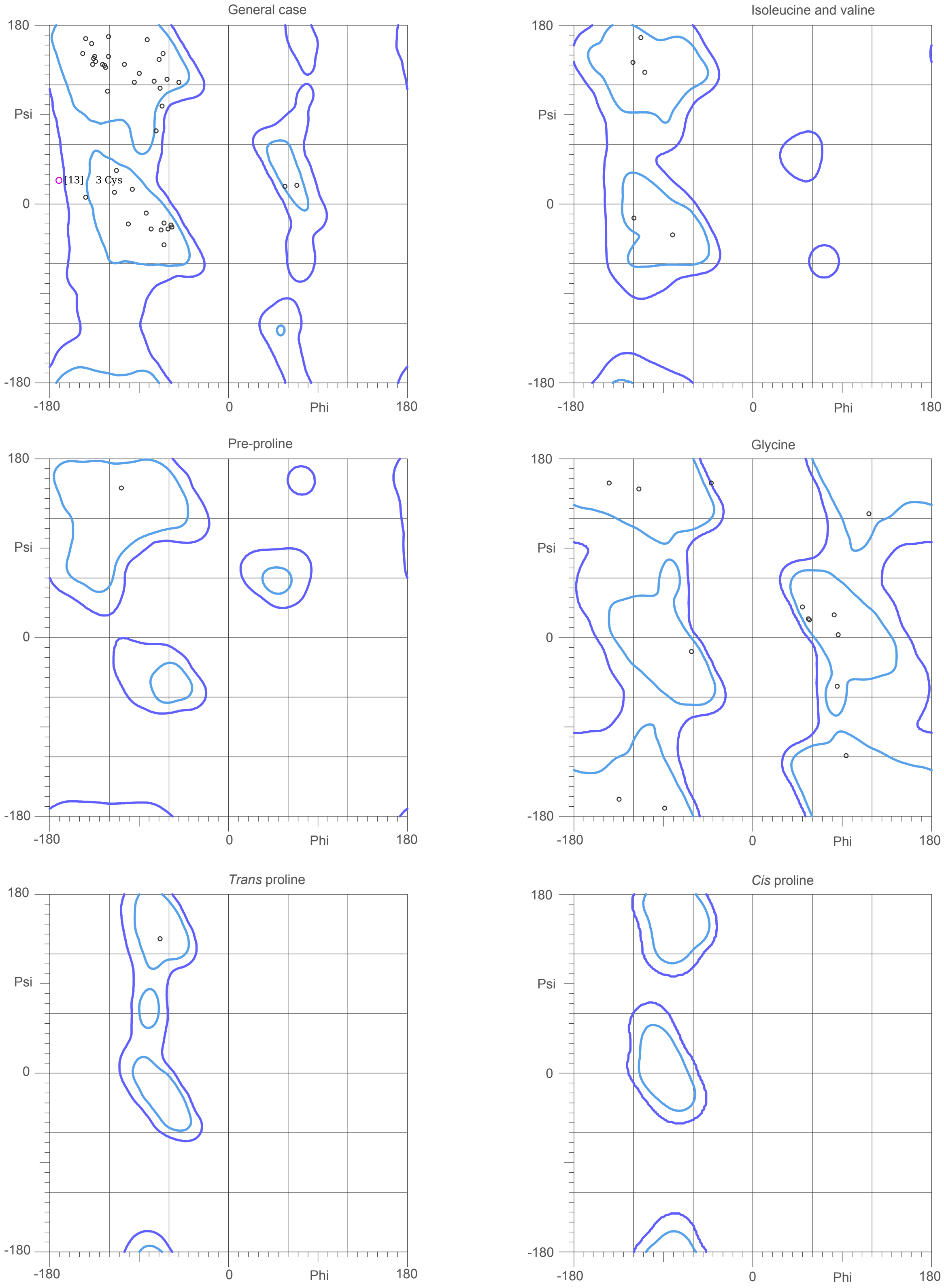

91.9% (57/62) of all residues were in favored (98%) regions.
98.4% (61/62) of all residues were in allowed (>99.8%) regions.

There were 1 outliers (phi, psi):
[13] 3 Cys (-171.7, 25.0)

MolProbity Ramachandran analysis

6trmH.pdb, model 14

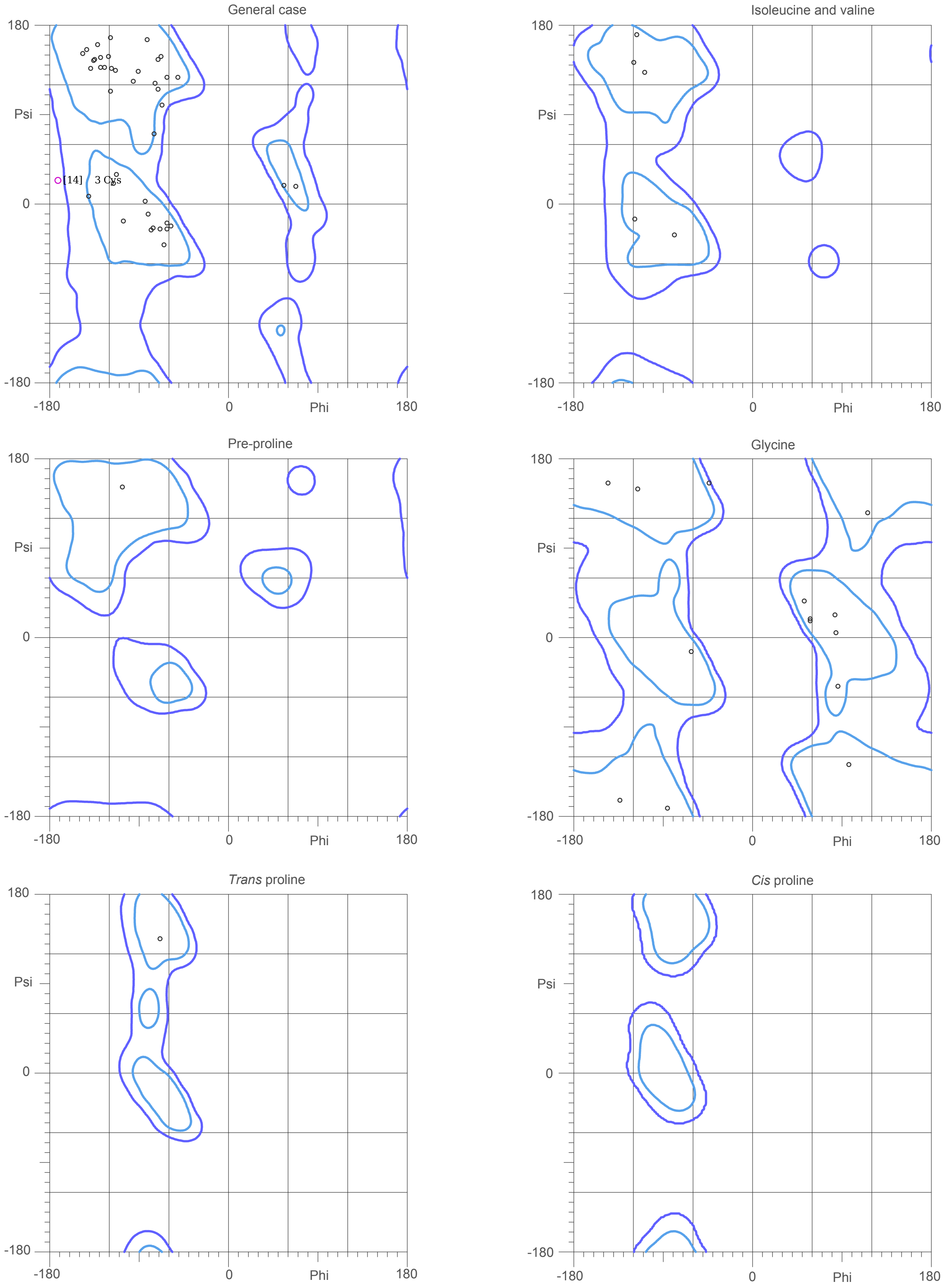

93.5% (58/62) of all residues were in favored (98%) regions.
98.4% (61/62) of all residues were in allowed (>99.8%) regions.

There were 1 outliers (phi, psi):
[14] 3 Cys (-172.7, 25.0)

MolProbity Ramachandran analysis

6trmH.pdb, model 15

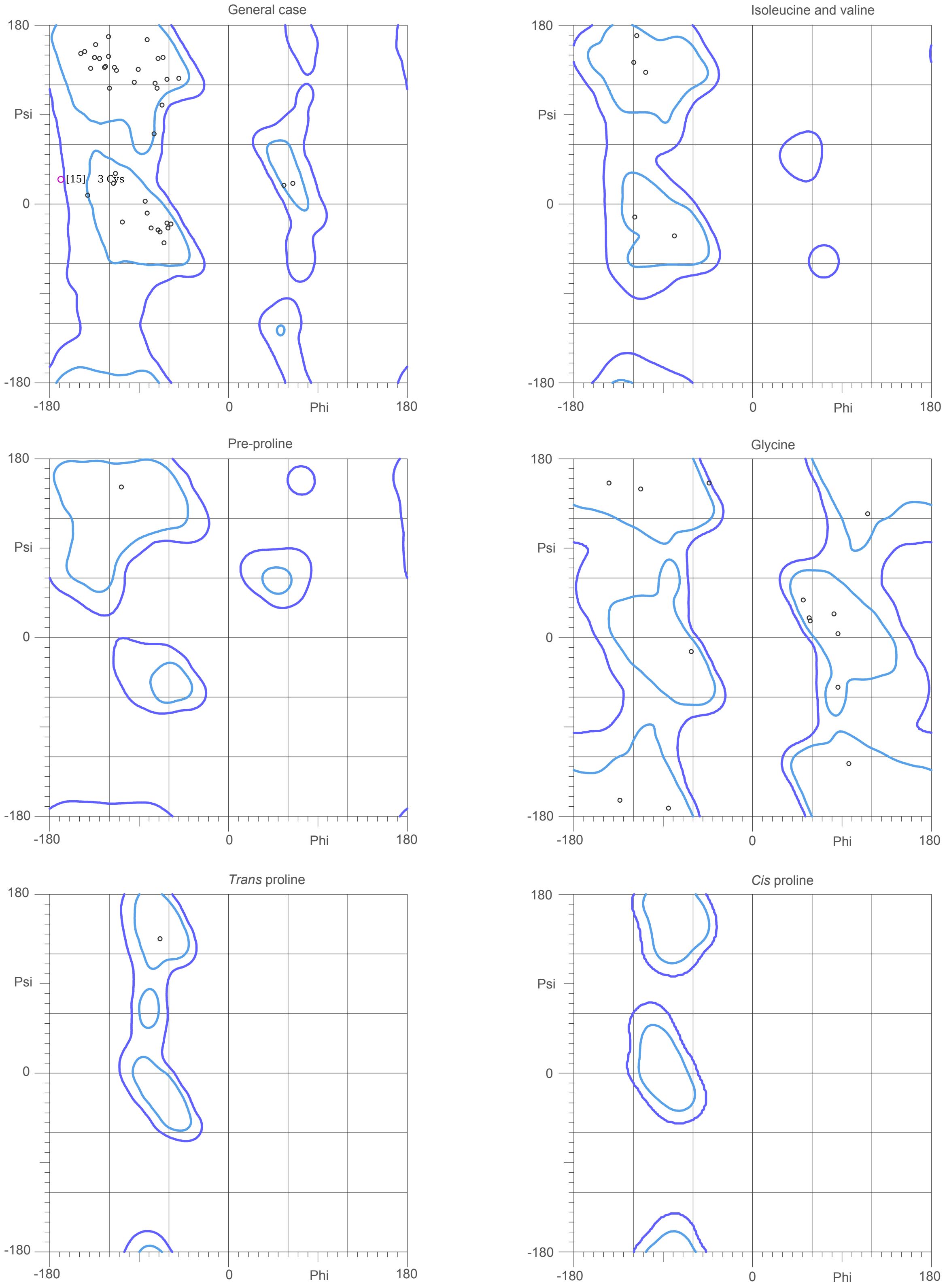

93.5% (58/62) of all residues were in favored (98%) regions.
98.4% (61/62) of all residues were in allowed (>99.8%) regions.

There were 1 outliers (phi, psi):
[15] 3 Cys (-169.4, 25.7)

MolProbity Ramachandran analysis

6trmH.pdb, model 16

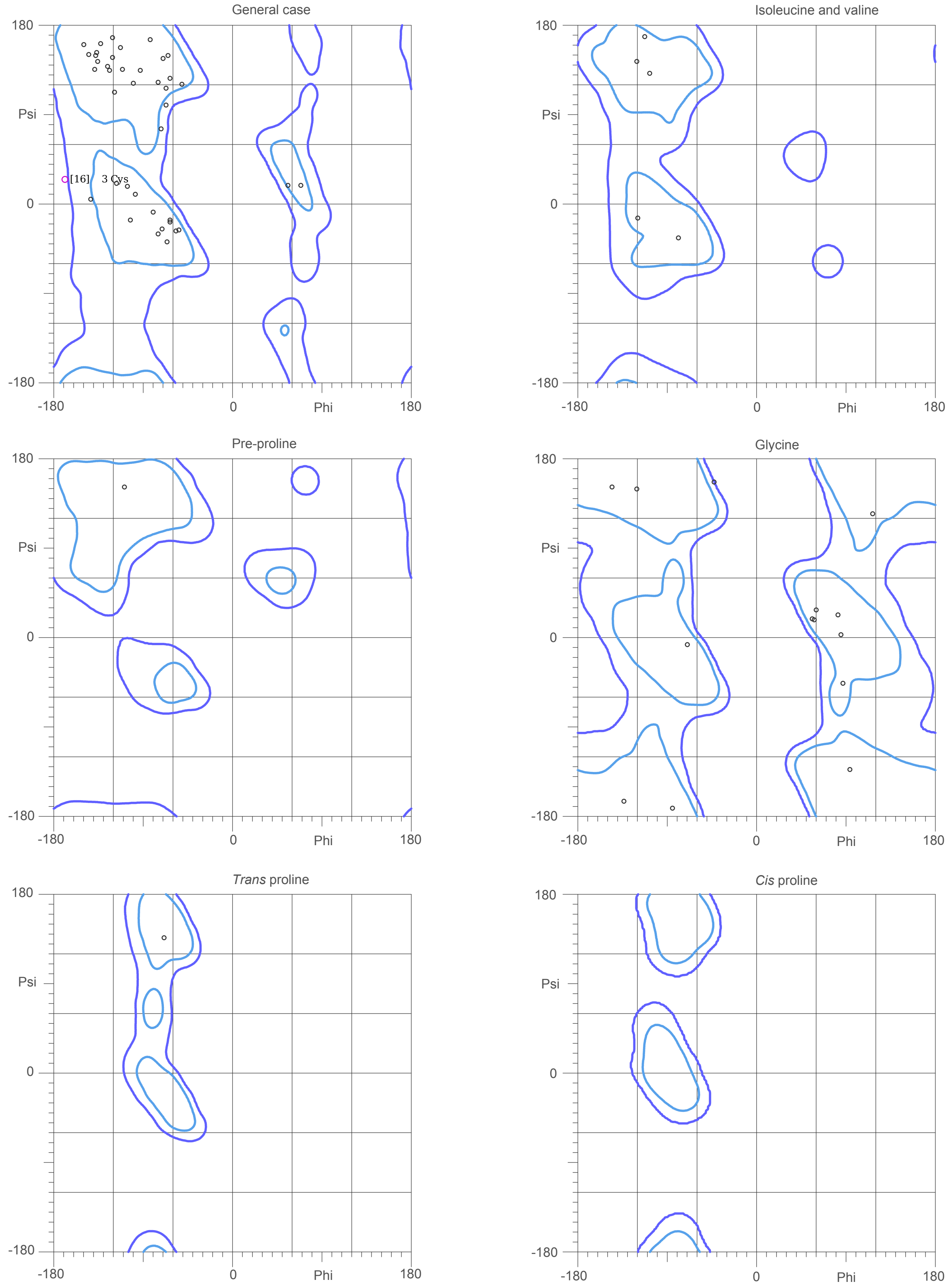

91.9% (57/62) of all residues were in favored (98%) regions.
98.4% (61/62) of all residues were in allowed (>99.8%) regions.

There were 1 outliers (phi, psi):
[16] 3 Cys (-170.0, 25.7)

MolProbity Ramachandran analysis

6trmH.pdb, model 17

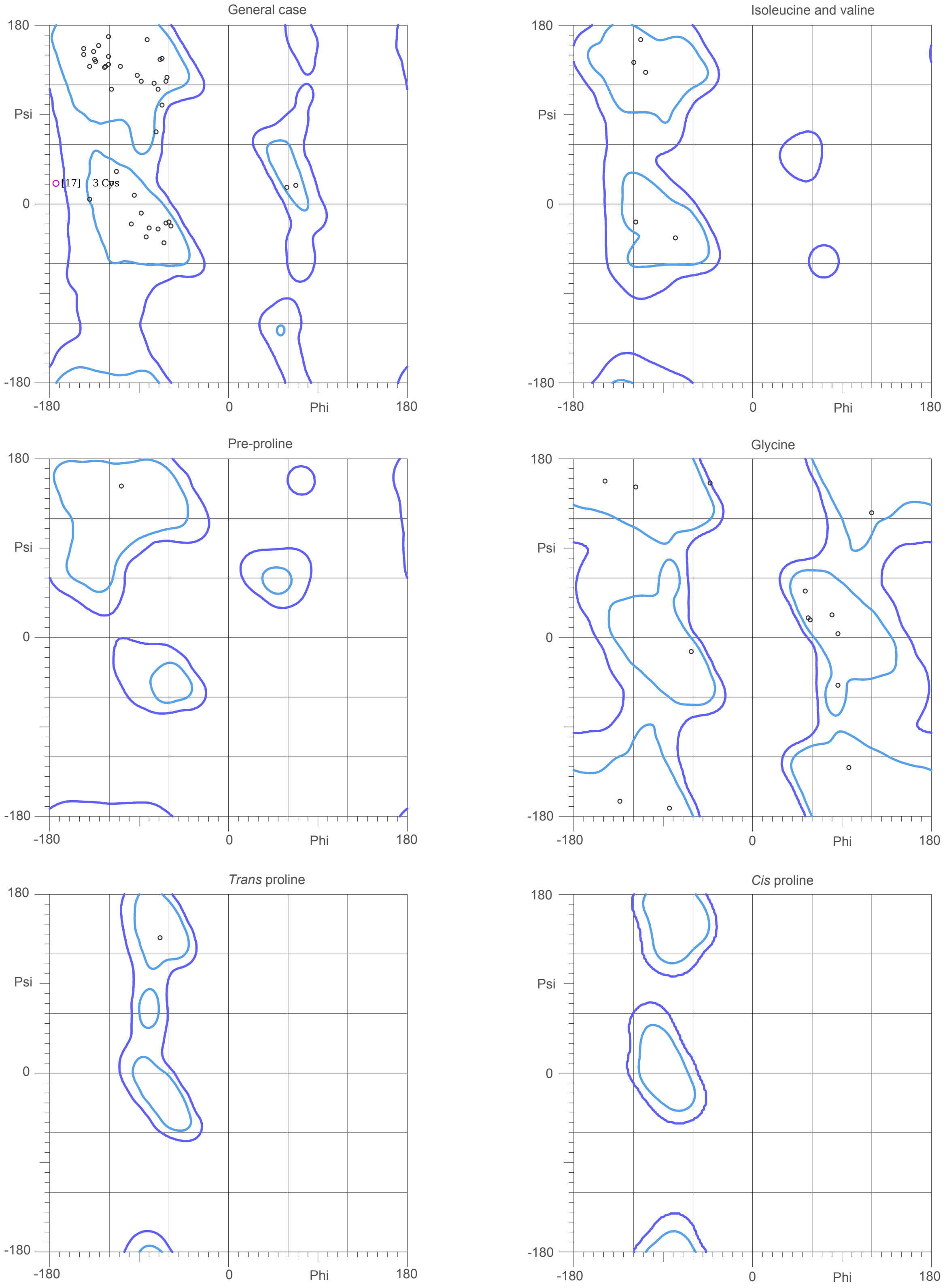

91.9% (57/62) of all residues were in favored (98%) regions.
98.4% (61/62) of all residues were in allowed (>99.8%) regions.

There were 1 outliers (phi, psi):
[17] 3 Cys (-174.2, 21.2)

MolProbity Ramachandran analysis

6trmH.pdb, model 18

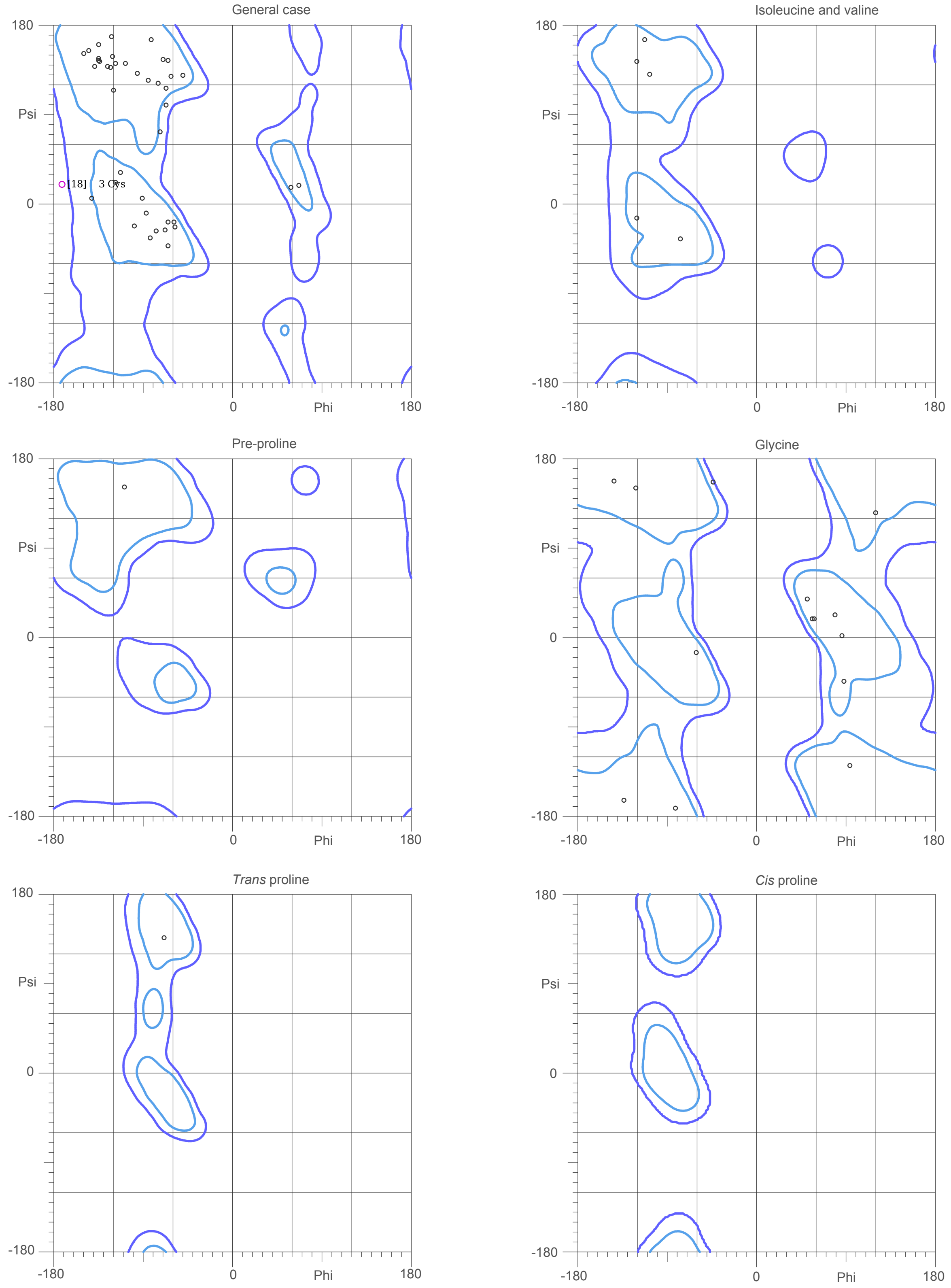

91.9% (57/62) of all residues were in favored (98%) regions.
98.4% (61/62) of all residues were in allowed (>99.8%) regions.

There were 1 outliers (phi, psi):
[18] 3 Cys (-173.0, 20.7)

MolProbity Ramachandran analysis

6trmH.pdb, model 19

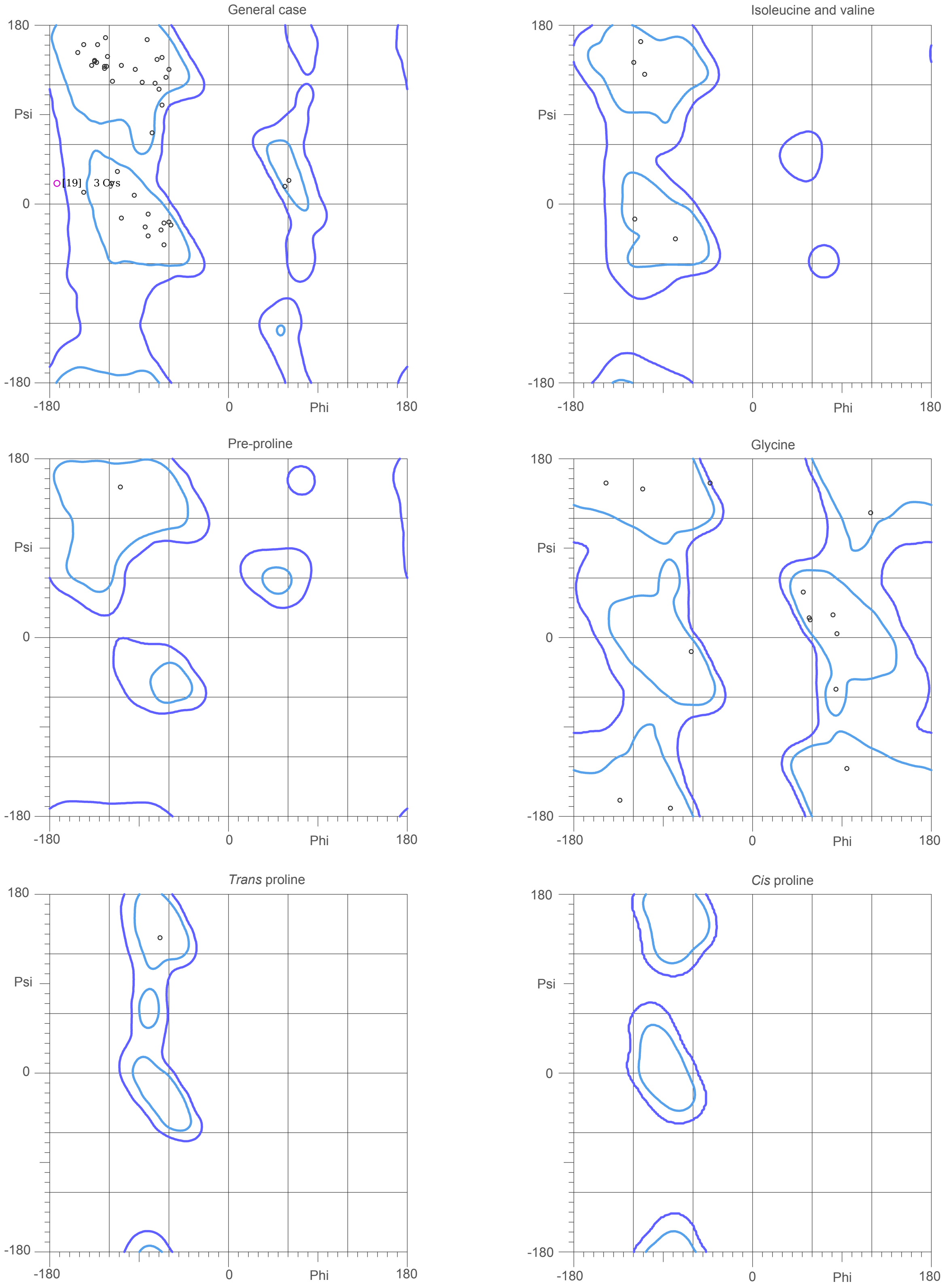

93.5% (58/62) of all residues were in favored (98%) regions.
98.4% (61/62) of all residues were in allowed (>99.8%) regions.

There were 1 outliers (phi, psi):
[19] 3 Cys (-173.8, 21.9)

MolProbity Ramachandran analysis

6trmH.pdb, model 20

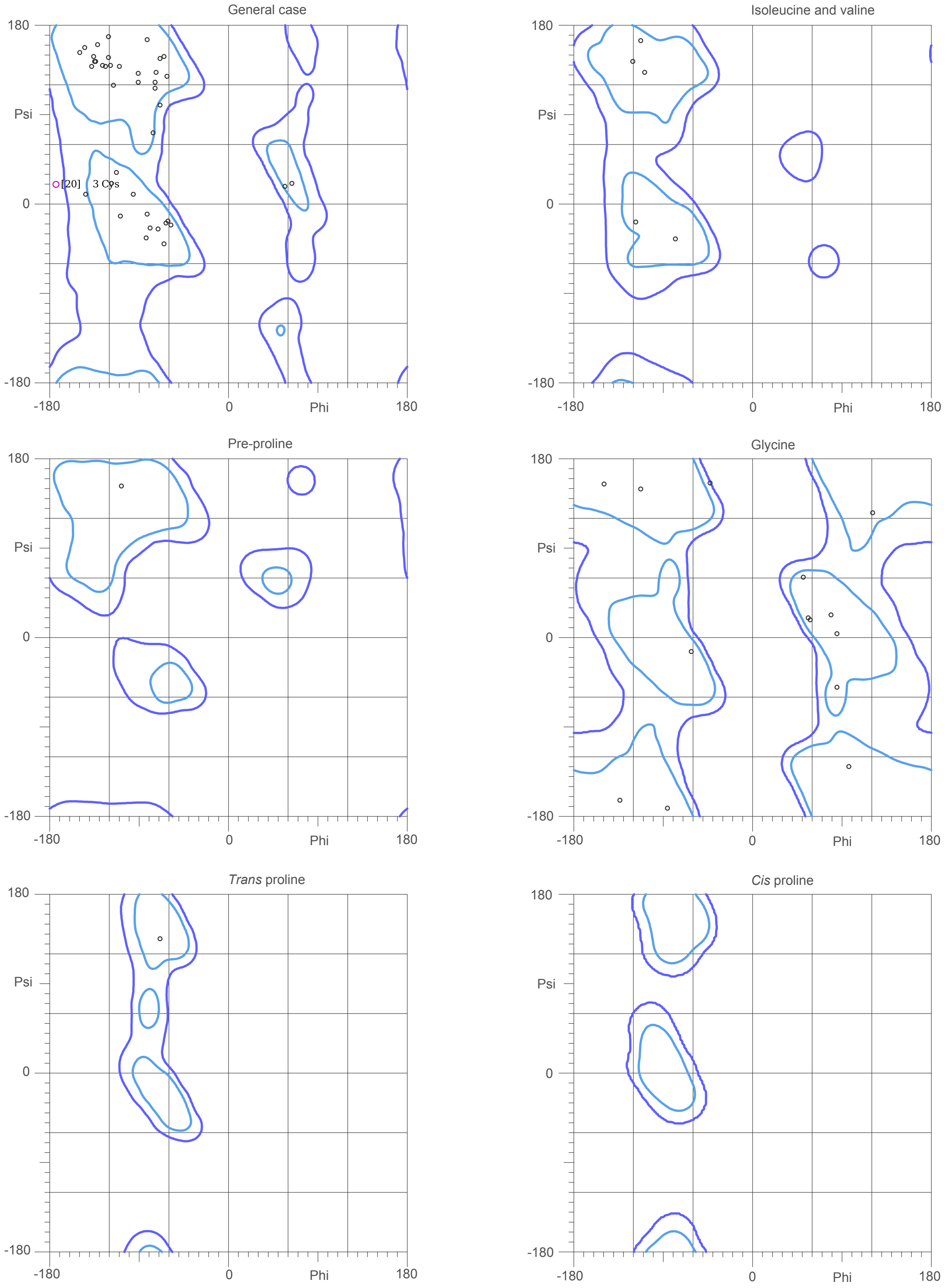

93.5% (58/62) of all residues were in favored (98%) regions.
98.4% (61/62) of all residues were in allowed (>99.8%) regions.

There were 1 outliers (phi, psi):
[20] 3 Cys (-174.3, 20.8)
