# Supplementary material for: Small Disulfide Proteins with Antifungal Impact: NMR Experimental Structures as Compared to Models of Alphafold Versions
Source: Int J Mol Sci. 2025 Jan 31;26(3):1247. doi: 10.3390/ijms26031247 (PMC11818080; doi:10.3390/ijms26031247)
Supplement: Supplementary file 1 [file ijms-26-01247-s001.zip › ijms-3414871-supplementary.pdf]

Supplementary Data

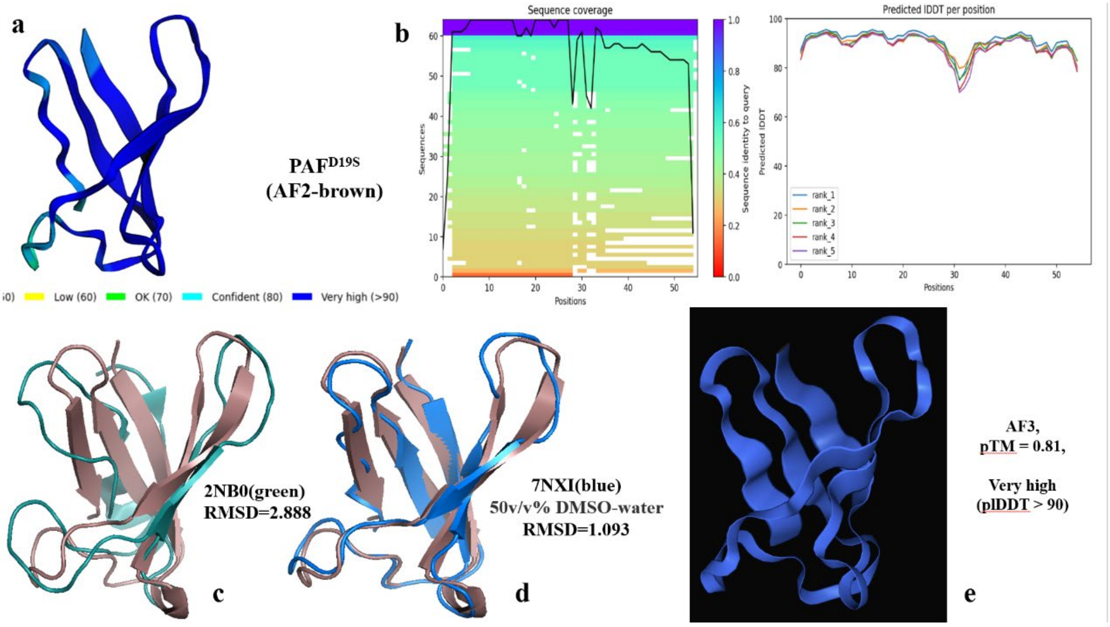

Figure S1. Comparison of AF2 predicted structure with NMR structure of PAFD19S. a. AF2 predicted confidence scores. b. Predicted sequence coverage and residue scores. c, d. Comparison of AF2 model with NMR structure in different solvents. e. AF3 predicted structure.

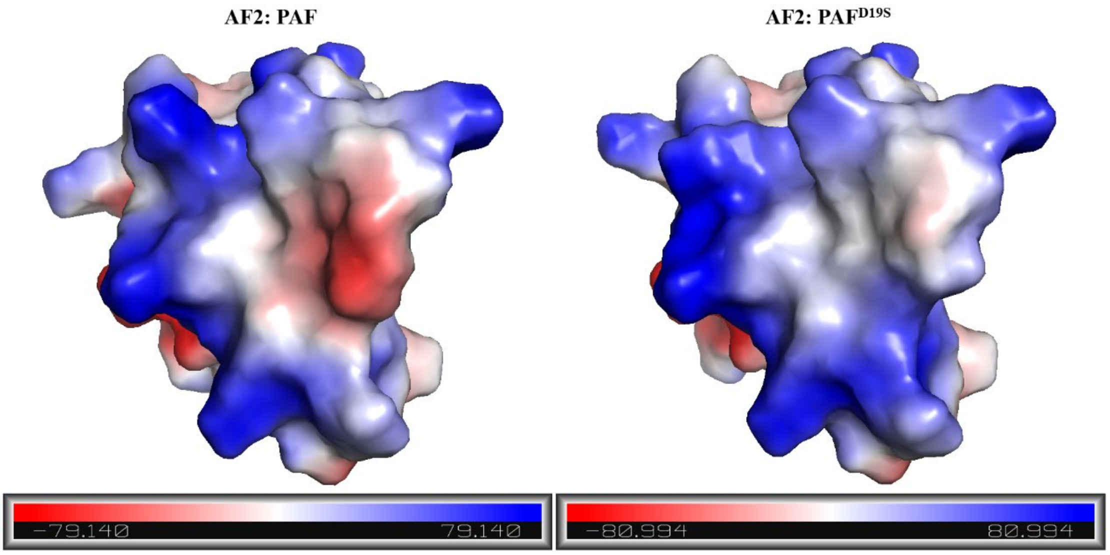

Figure S2. Surface electrostatic potential of AF2 structures of PAF and PAF^{D19S}. After site-directed mutagenesis, the charge distribution surrounding the 19S region in PAF^{D19S} transitions from negative to neutral.

Table S1. Evaluation Standards for MolProbity Parameters

**Category**	Validation	Good	Caution	Warning
**All-Atom contacts**	Clashscore, all atoms:	Percentile ≥ 66	66 > Percentile ≥ 33	Percentile < 33
**Protein Geometry**	Poor rotamers:	Outliers ≤ 0.3%	0.3% < Outliers ≤ 1.5%	Outliers > 1.5%
	Favored rotamers:	Favored ≥ 98%	98% > Favored ≥ 95%	Favored < 95%
	Ramachandran outliers:	Outliers ≤ 0.05%	0.05% < Outliers ≤ 0.5% or Outliers > 0.5% and Outlier count = 1	Outliers > 0.5% And Outlier count ≥ 2
	Ramachandran Favored:	Favored ≥ 98%	98% > Favored ≥ 95%	Favored < 95%
	Ramachandran Z-score:	abs(Z-score) ≤ 2	2 < abs(Z-score) ≤ 3	abs(Z-score) > 3%
	MolProbity score:	Percentile ≥ 66	66 > Percentile ≥ 33	Percentile < 33
	Cβ deviations >0.25A:	Outlier count = 0	0 < Outliers < 5%	Outliers ≥ 5%
	Bad bonds	Outlier bonds < 0.01%	0.01% ≤ Outlier bonds < 0.2%	Outlier bonds ≥ 0.2%
	Bad angles:	Outlier angles < 0.1%	0.1% ≤ Outlier angles < 0.5%	Outlier angles ≥ 0.5%
**Peptide Omegas**	Cis Prolines:	No suitable	universal cutoffs	for cisProline
	Cis nonProlines:	Peptides ≤ 0.05% Cis	0.05% < Cis Peptides ≤ 0.1%	Peptides > 0.1% Cis
	Twisted Peptides:	Twisted peptide count = 0	0 < Twisted peptides ≤ 0.1%	Peptides > 0.1% twisted
**Nucleic Acid Geometry**	Probably wrong sugar puckers:	Outlier count = 0	0 < Outliers ≤ 5%	Outliers > 5%
	Bad backbone Conformations:	Outliers ≤ 5%	5% < Outliers ≤ 15%	Outliers > 15%
	Bad bonds:	Outlier bonds < 0.01%	0.01% ≤ Outlier bonds < 0.2%	Outlier bonds ≥ 0.2%
	Bad angles:	Outlier angles < 0.1%	0.1% ≤ Outlier angles < 0.5%	Outlier angles ≥ 0.5%
**Low-resolution Criteria**	CaBLAM ouliers:	Outliers ≤ 1%	1% < Outliers ≤ 5%	Outliers > 5%
	CA Geometry Outliers:	Outliers ≤ 0.5%	0.5% < Outliers < 1%	Outliers > 1%

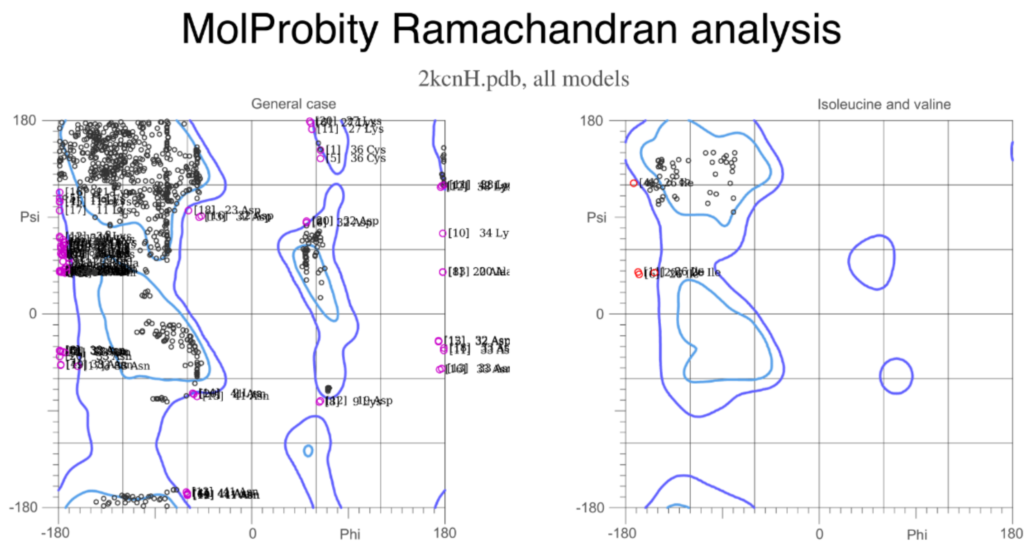

Fig S3a. PAF-NMR, PDB ID: 2KCN

72.4% (767/1060) of all residues were in favored (98%) regions.
92.2% (977/1060) of all residues were in allowed (>99.8%) regions.
This list is truncated; use the MolProbity multi-chart.html for complete list.
There were 83 outliers (phi, psi):

[1] 20 Ala (-174.0, 44.8)
[1] 34 Lys (-179.4, 71.2)
[1] 36 Cys (64.0, 153.3)
[2] 20 Ala (-170.2, 52.1)
[2] 33 Asn (-177.0, -35.9)
[2] 34 Lys (-175.7, 58.0)
[3] 20 Ala (-175.3, 42.3)
[3] 33 Asn (-179.9, -34.7)
[3] 34 Lys (-175.6, 60.3)
[4] 11 Lys (-179.7, 105.3)
[4] 20 Ala (-177.1, 39.9)
[4] 26 Ile (-173.9, 122.5)
[4] 33 Asn (-178.4, -47.4)
[4] 41 Asn (-60.8, -167.2)
[5] 11 Lys (-179.4, 108.7)
[5] 20 Ala (-173.8, 40.9)
[5] 33 Asn (-179.4, -34.8)
[5] 34 Lys (-174.8, 56.2)
[5] 36 Cys (64.5, 145.0)
[6] 20 Ala (-178.4, 40.0)
[6] 26 Ile (-169.0, 37.8)
[6] 27 Lys (55.1, 178.2)
[6] 32 Asp (51.5, 85.5)
[6] 33 Asn (-179.9, -34.9)
[6] 34 Lys (-175.3, 67.8)
[6] 41 Asn (-60.9, -168.4)
[7] 20 Ala (-175.7, 40.0)
[7] 33 Asn (-178.4, -35.5)
[7] 34 Lys (-175.5, 58.6)
[8] 9 Lys (63.9, -82.7)
[8] 20 Ala (178.6, 39.9)
[8] 33 Asn (-174.5, -36.6)
[8] 34 Lys (-176.3, 64.9)
[9] 20 Ala (-176.4, 49.3)
[9] 34 Lys (-177.0, 55.0)
[10] 20 Ala (-178.2, 61.0)
[10] 34 Lys (178.2, 75.5)
[11] 20 Ala (-171.5, 41.5)
[11] 26 Ile (-169.8, 39.7)
[11] 27 Lys (56.6, 172.5)
[11] 33 Asn (179.8, -34.7)
[11] 38 Lys (179.0, 120.9)
[12] 19 Asp (64.1, -81.7)
[12] 20 Ala (-170.8, 47.8)
[12] 32 Asp (174.7, -25.9)
[12] 34 Lys (-179.6, 72.7)
[12] 37 Thr (-174.8, 38.9)
[12] 38 Lys (176.9, 118.0)
[13] 20 Ala (178.9, 39.9)
[13] 32 Asp (-49.1, 90.8)
[13] 33 Asn (177.6, -51.6)
[13] 41 Asn (-61.1, -166.8)
[14] 9 Lys (-55.1, -74.7)
[14] 20 Ala (-179.2, 39.9)
[14] 34 Lys (-175.6, 64.1)
[14] 41 Asn (-60.2, -169.5)
[15] 11 Lys (-179.5, 105.0)
[15] 20 Ala (-178.2, 40.1)
[15] 32 Asp (174.9, -26.2)
[15] 34 Lys (-176.0, 69.8)
[15] 41 Asn (-51.4, -77.4)
[16] 11 Lys (-179.9, 113.3)
[16] 20 Ala (-175.5, 41.3)
[16] 32 Asp (-47.0, 91.3)

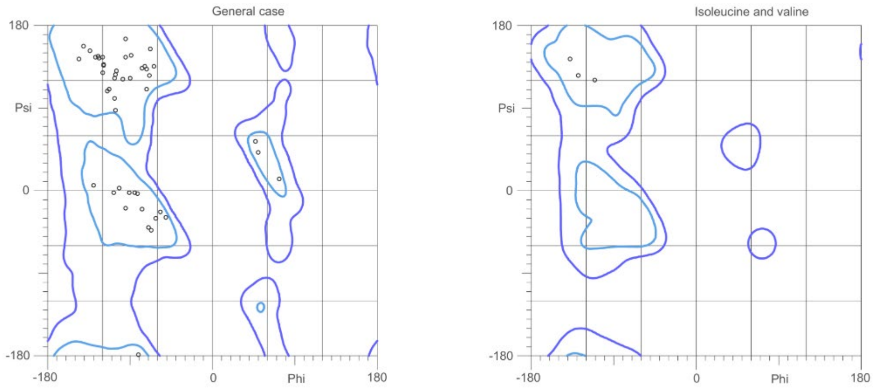

Fig S3b. PAF-AF2. 100.0% (53/53) of all residues were in favored (98%) regions. 100.0% (53/53) of all residues were in allowed (>99.8%) regions. There were no outliers.

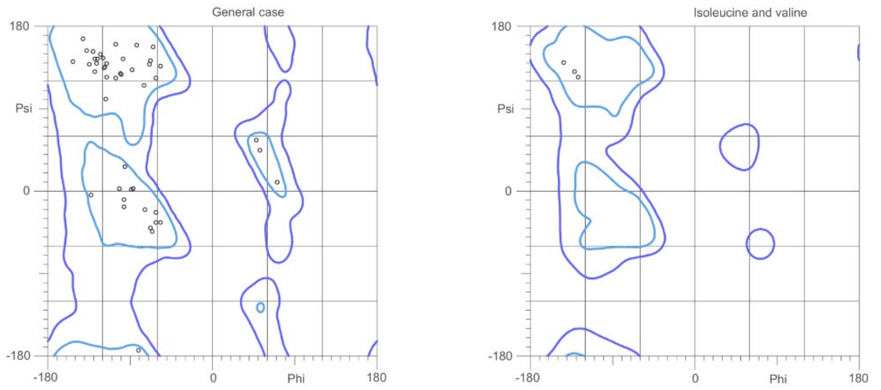

Fig S3c. PAF-AF3. 100.0% (53/53) of all residues were in favored (98%) regions. 100.0% (53/53) of all residues were in allowed (>99.8%) regions. There were no outliers.

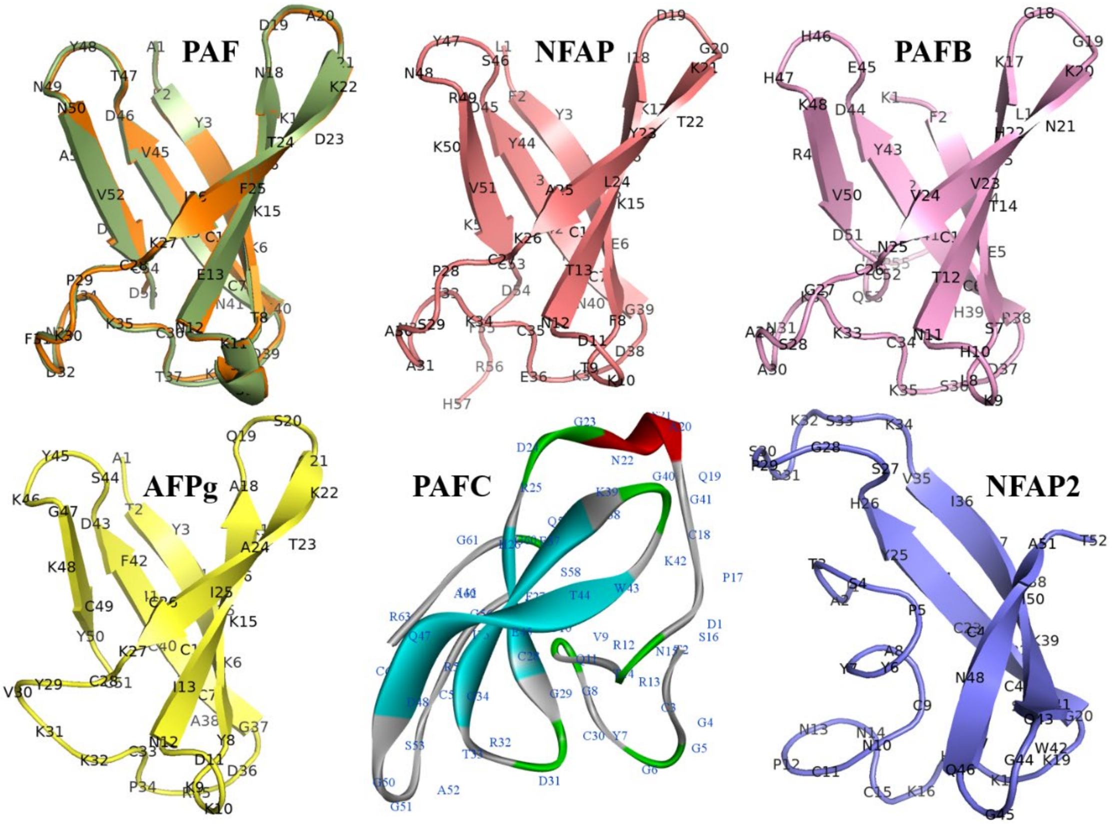

Figure S4. Predicted Structures of AFPs from AF Models. The predicted structures include PAF-AF2 (orange) and PAF-AF3 (green), the AF3 structures of NFAP, PAFB, AFPg, PAFC and NFAP2.

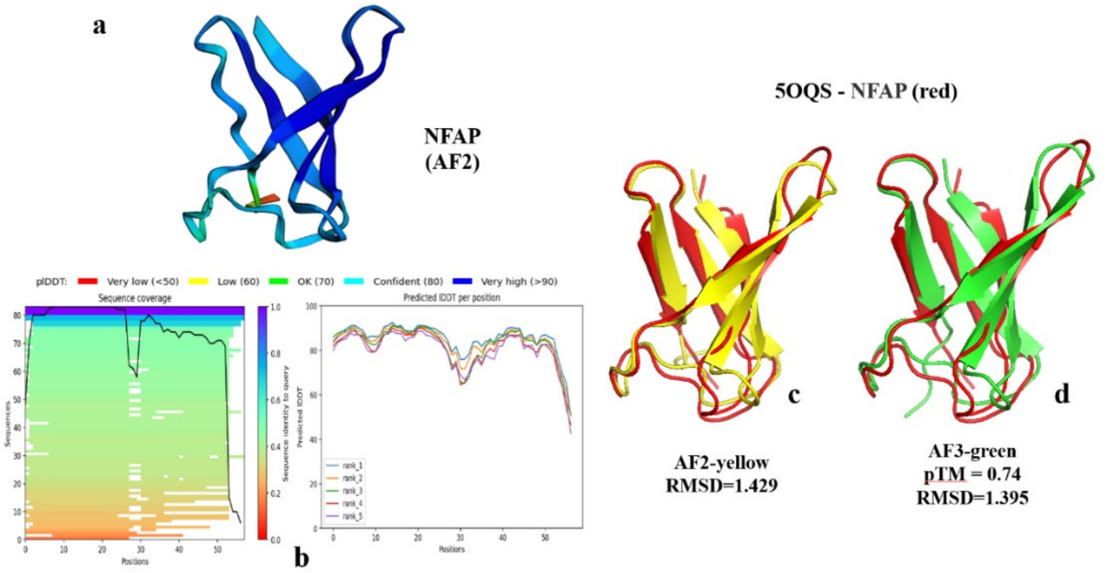

Figure S5a. Comparison of AF2 predicted structure with NMR structure of NFAP. a. AF2 predicted confidence scores. b. Predicted sequence coverage and residue scores. c. Comparison of AF2 model with NMR structure. e. Comparison of AF2 model with NMR structure.

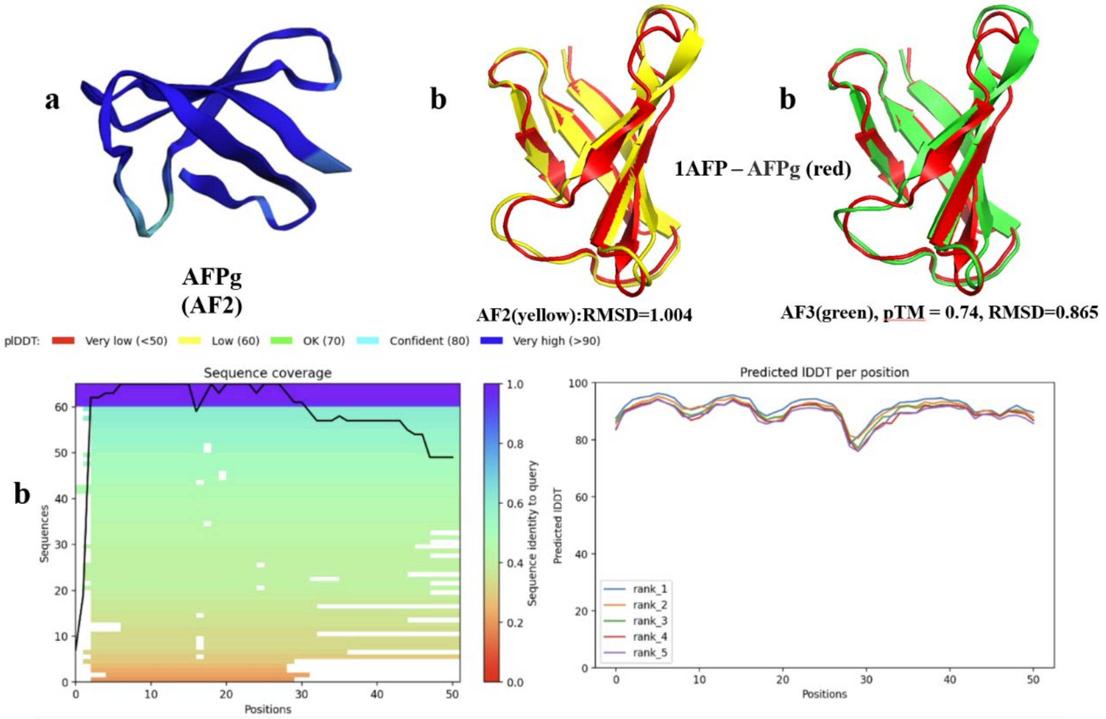

Figure S5b. Comparison of AF2 predicted structure with NMR structure of AFPg. a. AF2 predicted confidence scores. b. Predicted sequence coverage and residue scores. c. Comparison of AF2 model with NMR structure. e. Comparison of AF3 model with NMR structure.

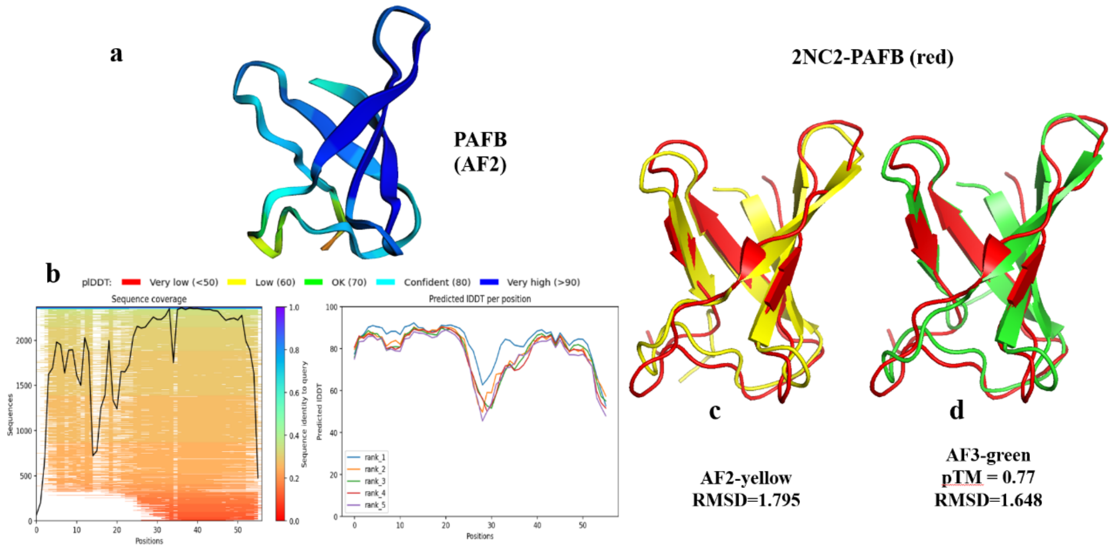

Figure S5c. Comparison of AF2 predicted structure with NMR structure of PAFB. a. AF2 predicted confidence scores. b. Predicted sequence coverage and residue scores. c. Comparison of AF2 model with NMR structure. e. Comparison of AF3 model with NMR structure.

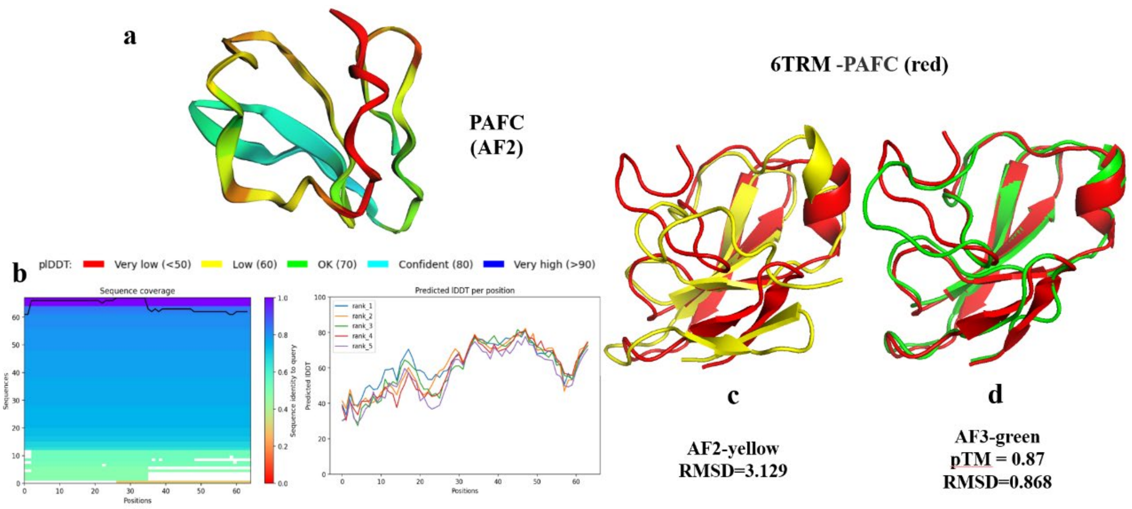

Figure S5d. Comparison of AF2 predicted structure with NMR structure of PAFC. a. AF2 predicted confidence scores. b. Predicted sequence coverage and residue scores. c. Comparison of AF2 model with NMR structure. e. Comparison of AF3 model with NMR structure.

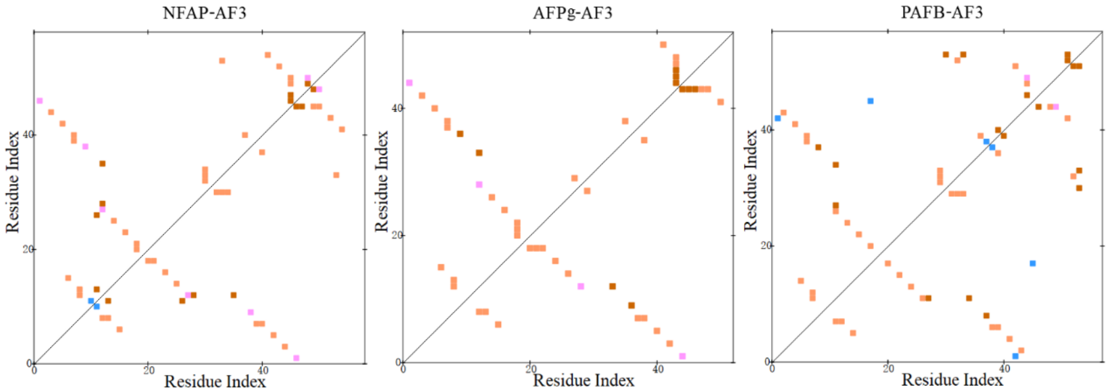

Figure S6. AF3 structure and hydrogen bond interaction distribution of NFAP, AFPg and PAFB. Hydrogen bonds are categorized based on their formation sites: main chain to main chain (orange), main chain to side chain (red), side chain to side chain (blue), multiple (pink).

MolProbity Ramachandran analysis

5oqsH.pdb, all models

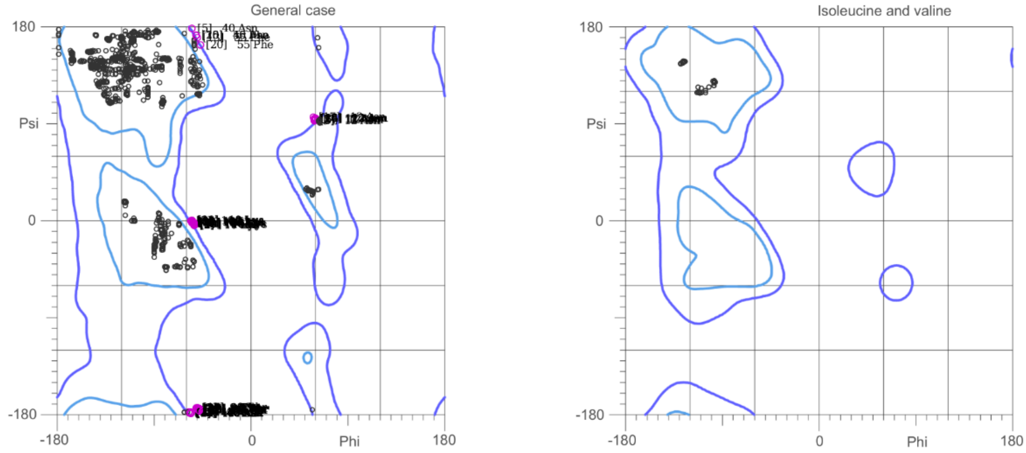

Fig S7a. NFAP-NMR, PDB ID: 5OQS

89.2% (981/1100) of all residues were in favored (98%) regions.
93.6% (1030/1100) of all residues were in allowed (>99.8%) regions.
This list is truncated; use the MolProbity multi-chart.html for complete list.
There were 70 outliers (phi, psi):

[1] 9 Thr (-49.0, -175.4)
[1] 10 Lys (-53.8, -3.0)
[1] 12 Asn (59.8, 95.2)
[2] 9 Thr (-51.2, -174.6)
[2] 10 Lys (-53.4, -4.2)
[2] 12 Asn (59.5, 94.7)
[2] 40 Asn (-56.2, -179.4)
[3] 9 Thr (-50.5, -175.1)
[3] 10 Lys (-53.9, -3.4)
[3] 12 Asn (60.7, 95.6)
[3] 40 Asn (-56.3, -179.5)
[4] 9 Thr (-49.7, -176.6)
[4] 10 Lys (-55.7, -1.5)
[4] 40 Asn (-56.4, -179.2)
[5] 9 Thr (-48.7, -177.2)
[5] 10 Lys (-56.1, -0.4)
[5] 40 Asn (-55.5, 179.7)
[6] 9 Thr (-49.8, -176.7)
[6] 10 Lys (-55.6, -1.7)
[6] 40 Asn (-56.7, -178.8)
[7] 9 Thr (-48.9, -176.0)
[7] 10 Lys (-54.4, -2.2)
[7] 12 Asn (62.2, 94.9)
[8] 9 Thr (-50.3, -174.1)
[8] 10 Lys (-53.4, -2.9)
[8] 12 Asn (59.7, 93.6)
[8] 40 Asn (-56.3, -179.7)
[9] 9 Thr (-49.9, -175.7)
[9] 10 Lys (-55.0, -1.9)
[9] 40 Asn (-56.3, -179.3)
[10] 9 Thr (-49.0, -175.3)
[10] 10 Lys (-53.7, -3.0)
[10] 12 Asn (59.6, 95.2)
[10] 40 Asn (-58.2, -178.5)
[11] 9 Thr (-49.3, -176.4)
[11] 10 Lys (-55.6, -0.8)
[11] 40 Asn (-56.2, -179.6)
[12] 9 Thr (-49.7, -175.0)
[12] 10 Lys (-53.5, -3.4)
[12] 12 Asn (60.1, 95.8)
[12] 40 Asn (-56.2, -179.5)
[13] 9 Thr (-48.6, -176.3)
[13] 10 Lys (-55.3, -1.0)
[13] 40 Asn (-57.6, -179.3)
[13] 55 Phe (-50.4, 170.7)
[14] 9 Thr (-48.6, -175.7)
[14] 10 Lys (-53.9, -2.7)
[14] 12 Asn (60.2, 95.1)
[15] 9 Thr (-48.7, -175.7)
[15] 10 Lys (-54.1, -2.7)
[15] 12 Asn (60.8, 95.1)
[15] 40 Asn (-56.7, -179.6)
[15] 55 Phe (-51.5, 172.1)
[16] 9 Thr (-49.8, -176.5)
[16] 10 Lys (-55.3, -1.6)
[16] 40 Asn (-56.3, -179.3)
[17] 9 Thr (-50.0, -174.4)
[17] 10 Lys (-52.5, -4.4)
[17] 12 Asn (58.2, 96.8)
[17] 40 Asn (-56.4, -179.2)
[18] 9 Thr (-49.9, -176.2)
[18] 10 Lys (-55.2, -2.1)
[18] 40 Asn (-56.3, -179.2)
[19] 9 Thr (-50.6, -175.8)

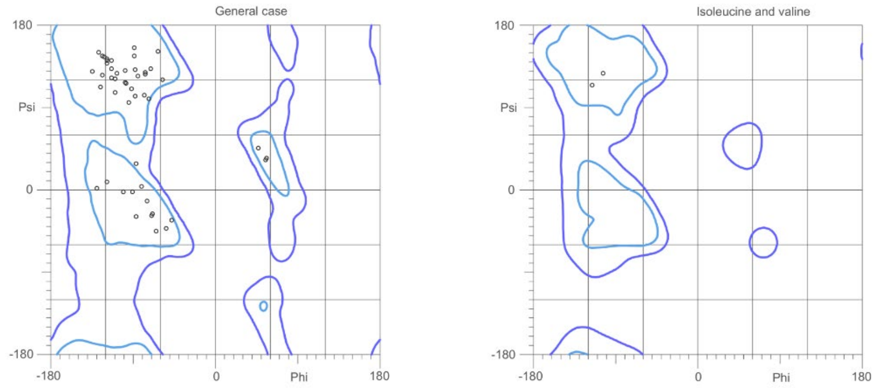

Fig S7b. NFAP-AF2. 98.2% (54/55) of all residues were in favored (98%) regions. 100.0% (55/55) of all residues were in allowed (>99.8%) regions. There were no outliers.

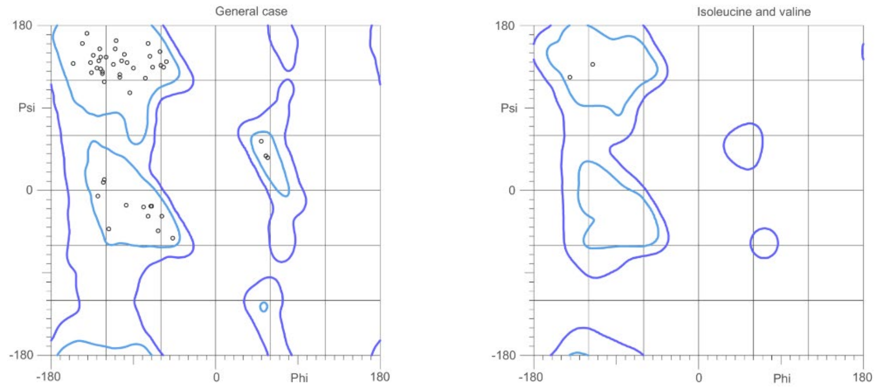

Fig S7c. NFAP-AF3. 100.0% (55/55) of all residues were in favored (98%) regions. 100.0% (55/55) of all residues were in allowed (>99.8%) regions. There were no outliers.

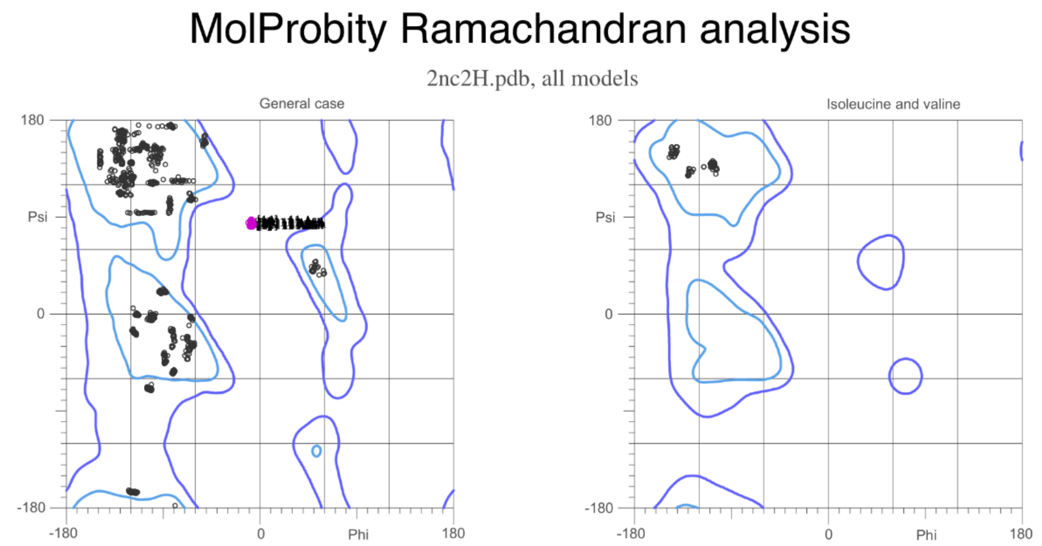

Fig S7d. PAFB-NMR, PDB ID: 2CN2

86.3% (932/1080) of all residues were in favored (98%) regions.
97.2% (1050/1080) of all residues were in allowed (>99.8%) regions.

There were 30 outliers (phi, psi):

[1] 11 Asn (-8.2, 83.2)
[2] 11 Asn (-8.0, 84.5)
[3] 11 Asn (-8.0, 85.8)
[4] 11 Asn (-9.0, 82.6)
[5] 11 Asn (-10.1, 85.1)
[6] 11 Asn (-8.6, 83.6)
[7] 3 Gly (-29.6, 135.2)
[7] 11 Asn (-9.0, 82.2)
[8] 3 Gly (-29.8, 134.6)
[8] 11 Asn (-8.5, 84.6)
[9] 3 Gly (-29.4, 135.1)
[9] 11 Asn (-7.8, 83.0)
[10] 3 Gly (-29.2, 135.1)
[10] 11 Asn (-8.0, 85.5)
[11] 3 Gly (-29.4, 135.3)
[11] 11 Asn (-8.3, 84.7)
[12] 11 Asn (-8.6, 83.0)
[13] 11 Asn (-7.7, 84.3)
[14] 3 Gly (-29.6, 135.5)
[14] 11 Asn (-8.9, 87.2)
[15] 3 Gly (-29.3, 135.2)
[15] 11 Asn (-8.3, 85.7)
[16] 11 Asn (-8.6, 83.5)
[17] 3 Gly (-29.2, 135.1)
[17] 11 Asn (-8.4, 84.0)
[18] 11 Asn (-6.9, 83.3)
[19] 3 Gly (-28.6, 134.7)
[19] 11 Asn (-8.4, 86.2)
[20] 3 Gly (-29.5, 135.4)
[20] 11 Asn (-7.2, 84.6)

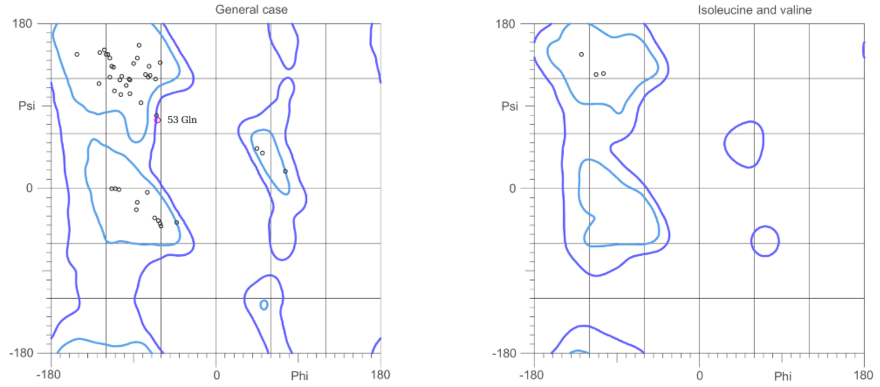

Fig S7e. PAFB-AF2. 90.7% (49/54) of all residues were in favored (98%) regions. 94.4% (51/54) of all residues were in allowed (>99.8%) regions. There were 3 outliers (phi, psi): 27 Gly (-1.6, 170.0), 53 Gln (-64.1, 75.4), 55 Pro (-46.9, 88.5)

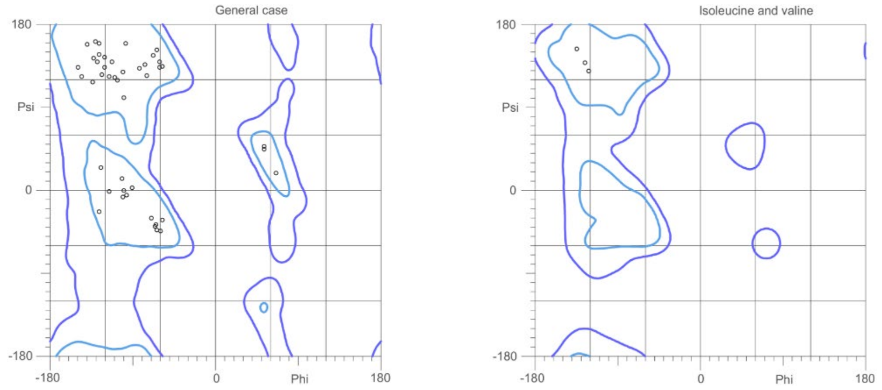

Fig S7f. PAFB-AF3. 100.0% (54/54) of all residues were in favored (98%) regions. 100.0% (54/54) of all residues were in allowed (>99.8%) regions. There were no outliers.

MolProbity Ramachandran analysis

1afp_trimmedH.pdb, all models

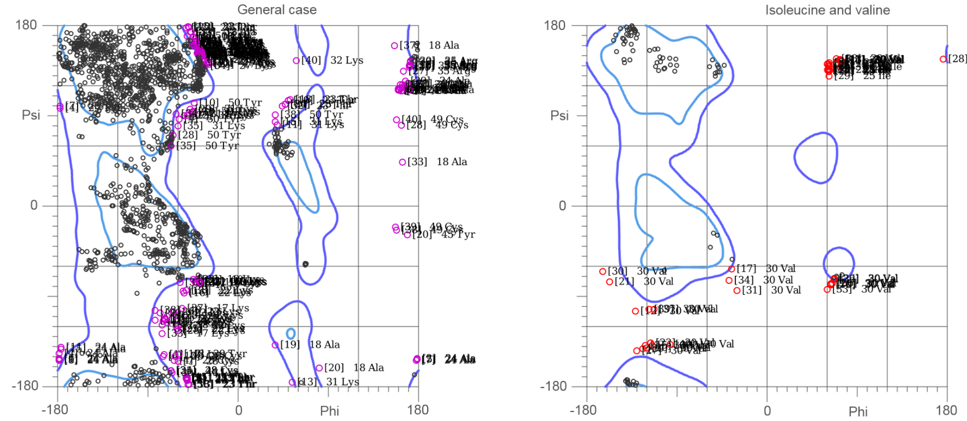

Fig S7g. AFPg-NMR, PDB ID: 1AFP

68.5% (1343/1960) of all residues were in favored (98%) regions.
89.1% (1747/1960) of all residues were in allowed (>99.8%) regions.
This list is truncated; use the MolProbity multi-chart.html for complete list.
There were 213 outliers (phi, psi):

[1] 23 Thr (-51.3, -173.0)
[1] 24 Ala (-179.1, -153.8)
[1] 30 Val (74.5, 146.9)
[1] 32 Lys (-40.7, 160.2)
[1] 48 Lys (-74.2, -148.9)
[1] 50 Tyr (-45.9, 95.1)
[2] 23 Thr (-51.6, -174.1)
[2] 24 Ala (179.3, -152.0)
[2] 27 Lys (-37.8, 155.7)
[2] 30 Val (-122.3, -139.4)
[2] 32 Lys (-39.8, 154.2)
[3] 10 Lys (-38.7, -77.2)
[3] 18 Ala (-45.9, 169.7)
[3] 24 Ala (161.4, 117.0)
[3] 25 Ile (60.6, 142.4)
[3] 32 Lys (-39.8, 157.2)
[4] 23 Thr (-51.7, -172.9)
[4] 24 Ala (-179.3, -147.8)
[4] 28 Cys (-65.0, -154.1)
[4] 29 Tyr (-178.2, 98.3)
[4] 50 Tyr (-59.1, 87.5)
[5] 10 Lys (-42.2, -76.2)
[5] 23 Thr (-52.3, -172.5)
[5] 24 Ala (179.1, -153.8)
[5] 27 Lys (-36.7, 154.7)
[5] 30 Val (-115.3, -140.0)
[5] 32 Lys (-41.5, 154.0)
[5] 35 Arg (172.5, 140.8)
[6] 23 Thr (-51.8, -173.1)
[6] 24 Ala (-179.6, -152.2)
[6] 27 Lys (-35.6, 150.9)
[6] 28 Cys (-75.2, -150.8)
[6] 32 Lys (-44.0, 167.1)
[6] 50 Tyr (-51.3, 170.6)
[7] 23 Thr (-51.6, -173.8)
[7] 24 Ala (178.7, -154.7)
[7] 27 Lys (-36.5, 147.6)
[7] 28 Cys (-60.5, -155.7)
[7] 29 Tyr (-178.2, 100.5)
[7] 32 Lys (-40.5, 153.8)
[8] 23 Thr (-51.8, -171.6)
[8] 24 Ala (-179.7, -154.5)
[8] 27 Lys (-33.3, 146.9)
[8] 32 Lys (-39.4, 154.3)
[9] 18 Ala (-45.5, 169.3)
[9] 24 Ala (160.6, 116.9)
[9] 25 Ile (60.8, 136.2)
[9] 32 Lys (-39.1, 156.4)
[9] 34 Pro (-75.0, -57.4)
[10] 22 Lys (-75.9, -115.2)
[10] 28 Cys (-63.6, -150.3)
[10] 32 Lys (-40.8, 153.5)
[10] 50 Tyr (-44.6, 103.0)
[11] 23 Thr (-51.6, -172.4)
[11] 24 Ala (-177.0, -141.7)
[11] 27 Lys (-37.0, 154.9)
[11] 30 Val (73.7, 146.7)
[11] 31 Lys (39.8, 81.6)
[11] 32 Lys (-43.0, 164.2)
[12] 22 Lys (-83.6, -114.6)
[12] 30 Val (-132.1, -106.0)
[12] 32 Lys (-40.1, 154.1)
[13] 22 Lys (-55.8, -85.6)
[13] 23 Thr (-49.9, -178.8)

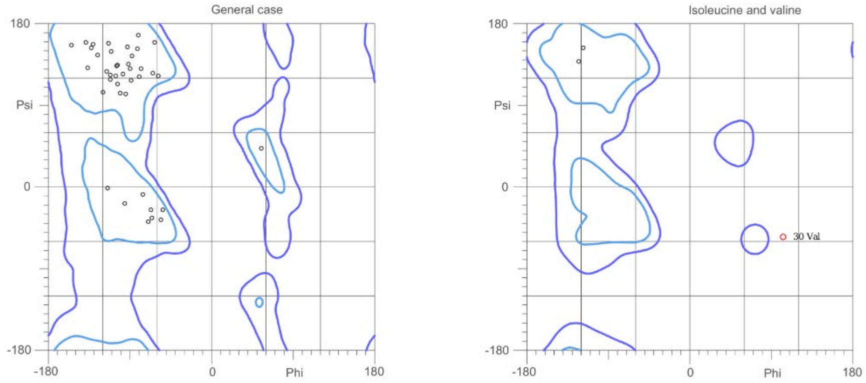

Fig S7h. AFPg-AF2. 98.0% (48/49) of all residues were in favored (98%) regions. 98.0% (48/49) of all residues were in allowed (>99.8%) regions. There were 1 outliers (phi, psi): 30 Val (103.4, -55.3)

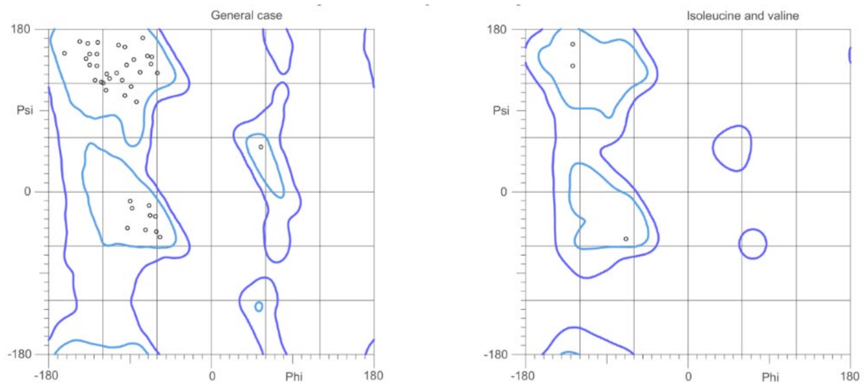

Fig S7i. AFPg-AF3. 100.0% (49/49) of all residues were in favored (98%) regions；100.0% (49/49) of all residues were in allowed (>99.8%) regions；There were no outliers.

Table S2a. MolProbity Evaluation of NMR Structure of AFPg.

All-Atom Contacts	Clashscore, all atoms:	102.98		0th percentile* (N=1784, all resolutions)
	Clashscore is the number of serious steric overlaps (> 0.4 Å) per 1000 atoms.			
Protein Geometry	Poor rotamers	15	34.88%	Goal: <0.3%
	Favored rotamers	14	32.56%	Goal: >98%
	Ramachandran outliers	6	12.24%	Goal: <0.05%
	Ramachandran favored	30	61.22%	Goal: >98%
	Rama distribution Z-score	-6.74 ± 0.86		Goal: abs(Z score) < 2
	MolProbity score^	4.56		0th percentile* (N=27675, 0Å - 99Å)
	Cβ deviations >0.25Å	0	0.00%	Goal: 0
	Bad bonds:	0 / 407	0.00%	Goal: 0%
	Bad angles:	0 / 538	0.00%	Goal: <0.1%
Peptide Omegas	Cis Prolines:	0 / 1	0.00%	Expected: ≤1 per chain, or ≤5%
Low-resolution Criteria	CaBLAM outliers	6	12.8%	Goal: <1.0%
	CA Geometry outliers	1	2.13%	Goal: <0.5%
Additional validations	Chiral volume outliers	0/52		
	Waters with clashes	0/0	0.00%	See UnDowser table for details

In the two column results, the left column gives the raw count, right column gives the percentage.

* 100th percentile is the best among structures of comparable resolution; 0th percentile is the worst. For clashscore the comparative set of structures was selected in 2004, for MolProbity score in 2006.

^ MolProbity score combines the clashscore, rotamer, and Ramachandran evaluations into a single score, normalized to be on the same scale as X-ray resolution.

Key to table colors and cutoffs here:

Live sorting requires JavaScript enabled and Safari 2, Firefox 1.5, or IE 6

#	Alt	Res	High B	Clash > 0.4Å	Ramachandran	Rotamer	Cβ deviation	CaBLAM	Bond lengths	Bond angles	Cis Peptides
			Avg: 2.58	Clashscore: 102.98	Outliers: 6 of 49	Poor rotamers: 15 of 43	Outliers: 0 of 47	Outliers: 7 of 47	Outliers: 0 of 51	Outliers: 0 of 51	Non-Trans: 0 of 50
A 1		ALA	0.97	0.66Å<br>HB3 with A 44 SER OG	-	-	0.05Å	-	-	-	-
A 2		THR	0.86	0.71Å<br>HG22 with A 41 GLU HB3	Favored (58.2%)<br>General / -64.1,143.0	Allowed (1.2%) *m*<br>chi angles: 320.2	0.12Å	-	-	-	-
A 3		TYR	2.28	0.52Å<br>CZ with A 19 GLN HB2	Allowed (1.08%)<br>General / -97.8,-163.8	Favored (3.5%) *m-10*<br>chi angles: 307,346.7	0.16Å	Favored (7.437%)	-	-	-
A 4		ASN	2.56	0.46Å<br>ND2 with A 3 TYR O	Favored (17.96%)<br>General / -155.6,141.3	Favored (6.5%) *m-40*<br>chi angles: 278.1,258	0.08Å	CA Geom Outlier (0.188%)	-	-	-
A 5		GLY	0.42	0.42Å<br>C with A 6 LYS HG3	Allowed (1.8%)<br>Glycine / -166.8,-135.1	-	-	Favored (17.133%)<br>beta sheet	-	-	-
A 6		LYS	2.54	0.42Å<br>HG3 with A 5 GLY C	Favored (42.87%)<br>General / -147.0,160.0	Favored (6%) *mttt*<br>chi angles: 305.4,178.8,179.6,227.8	0.09Å	Favored (15.041%)<br>beta sheet	-	-	-
A 7		CYS	0.98	0.46Å<br>C with A 33 CYS HG	Favored (52.07%)<br>General / -134.9,153.4	Favored (2.1%) *p*<br>chi angles: 45.4	0.04Å	Favored (56.409%)<br>beta sheet	-	-	-
A 8		TYR	1.87	0.55Å<br>CD2 with A 13 ILE HD11	Favored (55.15%)<br>General / -114.8,134.4	Favored (17.3%) *m-10*<br>chi angles: 298.6,346.7	0.16Å	Favored (63.44%)	-	-	-
A 9		LYS	2.15	0.49Å<br>N with A 33 CYS SG	Favored (23.38%)<br>General / -75.4,-48.4	OUTLIER (0%)<br>chi angles: 63.2,87.3,91.6,126.9	0.09Å	Favored (16.586%)	-	-	-
A 10		LYS	3.54	0.46Å<br>N with A 9 LYS CD	Favored (73.29%)<br>General / -54.8,-47.9	Allowed (0.6%) *ttmt*<br>chi angles: 217.5,217.6,280.4,162.1	0.09Å	Favored (64.289%)<br>alpha helix	-	-	-
A 11		ASP	2.44	0.47Å<br>O with A 12 ASN C	Favored (56.24%)<br>General / -89.8,-6.1	Favored (65%) *m-30*<br>chi angles: 285.9,149.3	0.08Å	Favored (16.554%)	-	-	-
A 12		ASN	2.41	0.56Å<br>CG with A 12 ASN O	Allowed (1.25%)<br>General / 38.7,61.1	Allowed (1.7%) *t0*<br>chi angles: 165.8,238.3	0.08Å	Favored (11.194%)	-	-	-
A 13		ILE	1.32	0.83Å<br>HG22 with A 27 LYS HA	Favored (16.08%)<br>Ile or Val / -142.9,165.3	Allowed (0.4%) *pp*<br>chi angles: 38.8,74.2	0.11Å	Favored (30.032%)<br>beta sheet	-	-	-
A 14		CYS	1.27	0.53Å<br>HB2 with A 51 CYS SG	Favored (15.55%)<br>General / -123.5,110.8	Allowed (1.1%) *t*<br>chi angles: 162	0.04Å	Favored (32.811%)<br>beta sheet	-	-	-
A 15		LYS	2.84	0.68Å<br>CG with A 25 ILE HG23	Favored (46.02%)<br>General / -104.7,135.5	OUTLIER (0.2%)<br>chi angles: 311.2,147.4,69.7,265.4	0.09Å	Favored (20.085%)<br>beta sheet	-	-	-
A 16		TYR	1.06	0.91Å<br>CE2 with A 23 THR HG21	Favored (17.6%)<br>General / -164.2,157.2	Allowed (0.9%) *p90*<br>chi angles: 53.6,293.9	0.16Å	Favored (17.801%)<br>beta sheet	-	-	-
A 17		LYS	4.53	-	Favored (18.65%)<br>General / -86.0,109.3	OUTLIER (0%)<br>chi angles: 240.6,276.2,143.6,149	0.09Å	Favored (20.473%)	-	-	-
A 18		ALA	1.92	0.72Å<br>HB2 with A 23 THR CB	Allowed (0.81%)<br>General / -52.3,160.3	-	0.05Å	Favored (8.343%)	-	-	-
A 19		GLN	4.2	0.52Å<br>HB2 with A 3 TYR CZ	Favored (24.03%)<br>General / -57.1,-20.2	Allowed (0.7%) *tm130*<br>chi angles: 199.6,271.1,109.7	0.03Å	Favored (9.318%)	-	-	-
A 20		SER	2.6	-	Favored (5.03%)<br>General / -131.4,14.5	OUTLIER (0.1%)<br>chi angles: 26.9	0.01Å	Favored (11.804%)	-	-	-
A 22		LYS	6.89	0.44Å<br>O with A 23 THR O	Allowed (0.52%)<br>General / -68.2,-66.8	OUTLIER (0%)<br>chi angles: 192.5,270.3,245.4,222.7	0.09Å	CaBLAM Disfavored (3.918%)	-	-	-
A 23		THR	5.52	0.91Å<br>HG21 with A 16 TYR CE2	OUTLIER (0)%)<br>General / -51.3,-173.0	OUTLIER (0%)<br>chi angles: 226.8	0.12Å	CaBLAM Disfavored (2.274%)	-	-	-
A 24		ALA	2.32	0.75Å<br>N with A 23 THR HG23	OUTLIER (0.03)%)<br>General / -179.1,-153.8	-	0.05Å	CaBLAM Disfavored (4.721%)	-	-	-
A 25		ILE	1.94	0.68Å<br>HG23 with A 15 LYS CG	Allowed (0.56%)<br>Ile or Val / -43.7,141.5	Allowed (0.3%) *mp*<br>chi angles: 321.6,100	0.11Å	CaBLAM Outlier (0.851%)	-	-	-
A 26		CYS	1.39	0.53Å<br>O with A 27 LYS C	Favored (4.42%)<br>General / -146.9,109.0	Favored (52.8%) *t*<br>chi angles: 184.1	0.04Å	CaBLAM Disfavored (3.661%)	-	-	-
A 27		LYS	4.99	0.83Å<br>HA with A 13 ILE HG22	Allowed (0.06%)<br>General / -34.0,140.8	OUTLIER (0%)<br>chi angles: 29.3,259.9,126.7,139.7	0.09Å	CaBLAM Disfavored (2.628%)<br>try beta sheet	-	-	-
A 28		CYS	1.66	0.43Å<br>CB with A 12 ASN ND2	Favored (3.24%)<br>General / -76.5,77.2	OUTLIER (0.1%)<br>chi angles: 250.4	0.04Å	Favored (33.383%)	-	-	-
A 29		TYR	5.02	0.83Å<br>O with A 30 VAL HG22	Favored (3.56%)<br>General / -62.7,114.4	Favored (5.3%) *t80*<br>chi angles: 193.2,38.9	0.16Å	Favored (5.849%)	-	-	-
A 30		VAL	3.64	0.83Å<br>HG22 with A 29 TYR O	OUTLIER (0.01)%)<br>Ile or Val / 74.5,146.9	OUTLIER (0.1%)<br>chi angles: 333.8	0.09Å	CaBLAM Disfavored (3.771%)	-	-	-
A 31		LYS	3.29	0.46Å<br>C with A 30 VAL O	Allowed (0.09%)<br>General / 39.4,74.8	OUTLIER (0.2%)<br>chi angles: 207.4,181.2,250.9,95.7	0.09Å	CaBLAM Outlier (0.201%)	-	-	-
A 32		LYS	4.69	0.44Å<br>CG with A 33 CYS N	OUTLIER (0.01)%)<br>General / -40.7,160.2	Allowed (0.4%) *ttmt*<br>chi angles: 148.2,182.6,321.1,203	0.09Å	CaBLAM Disfavored (3.313%)	-	-	-
A 33		CYS	1.32	0.49Å<br>SG with A 9 LYS N	Favored (64.73%)<br>Pre-Pro / -56.6,144.3	Allowed (0.4%) *t*<br>chi angles: 156	0.04Å	Favored (15.087%)	-	-	-
A 34		PRO	0.78	0.47Å<br>HD2 with A 38 ALA CB	Allowed (0.3%)<br>Trans-Pro / -75.0,-45.0	Favored (18.4%) *Cg_endo*<br>chi angles: 18.6,346,3.8	0.07Å	CaBLAM Disfavored (2.85%)	-	-	-
A 35		ARG	4.11	0.49Å<br>O with A 36 ASP C	Favored (2.87%)<br>General / -167.5,137.8	OUTLIER (0.1%)<br>chi angles: 52,128.6,160.8,198.4	0.03Å	CaBLAM Outlier (0.379%)	-	-	-
A 36		ASP	2.45	0.49Å<br>HA with A 8 TYR HA	Allowed (0.82%)<br>General / -37.4,132.0	OUTLIER (0%)<br>chi angles: 150.7,301.4	0.08Å	Favored (7.107%)	-	-	-
A 38		ALA	0.45	0.47Å<br>CB with A 34 PRO HD2	Allowed (1.43%)<br>General / -44.4,118.0	-	0.05Å	Favored (26.376%)	-	-	-
A 39		LYS	3.53	0.44Å<br>C with A 39 LYS CE	Favored (50.87%)<br>General / -62.7,147.0	OUTLIER (0.2%)<br>chi angles: 183.2,247.6,57.6,184.1	0.09Å	Favored (15.604%)<br>beta sheet	-	-	-
A 40		CYS	2.21	0.50Å<br>CB with A 51 CYS SG	Favored (26.79%)<br>General / -157.5,153.2	Allowed (0.3%) *p*<br>chi angles: 33.5	0.04Å	Favored (45.686%)<br>beta sheet	-	-	-
A 41		GLU	3.87	0.71Å<br>HB3 with A 2 THR HG22	Favored (37.58%)<br>General / -116.8,150.9	Favored (9.8%) *mt-10*<br>chi angles: 309.2,196,109.5	0.03Å	Favored (45.214%)<br>beta sheet	-	-	-
A 42		PHE	1.04	0.56Å<br>CD1 with A 16 TYR OH	Favored (8.96%)<br>General / -106.5,99.4	Favored (77.6%) *t80*<br>chi angles: 173.9,71.8	0.14Å	Favored (39.322%)<br>beta sheet	-	-	-
A 43		ASP	2.69	0.64Å<br>CG with A 50 TYR CE1	Favored (15.27%)<br>General / -62.3,122.3	Allowed (0.9%) *m-30*<br>chi angles: 273.4,39	0.08Å	Favored (32.173%)<br>beta sheet	-	-	-
A 44		SER	1.32	0.66Å<br>OG with A 1 ALA HB3	Favored (2.62%)<br>General / -90.8,24.7	Favored (9.8%) *p*<br>chi angles: 82.4	0.01Å	CaBLAM Disfavored (2.112%)	-	-	-
A 46		LYS	3.95	-	Allowed (0.97%)<br>General / -131.9,-51.7	Allowed (0.3%) *mtmm*<br>chi angles: 318.7,210.8,244.8,289.8	0.09Å	CaBLAM Outlier (0.093%)	-	-	-
A 48		LYS	3.15	-	OUTLIER (0.02)%)<br>General / -74.2,-148.9	OUTLIER (0%)<br>chi angles: 222.8,85,54,134.7	0.09Å	CaBLAM Outlier (0.044%)	-	-	-
A 49		CYS	2.63	0.41Å<br>O with A 51 CYS SG	Allowed (0.49%)<br>General / -159.2,70.9	OUTLIER (0%)<br>chi angles: 356.9	0.04Å	CaBLAM Outlier (0.104%)	-	-	-
A 50		TYR	1.51	0.64Å<br>CE1 with A 43 ASP CG	OUTLIER (0.01)%)<br>General / -45.9,95.1	Favored (10.4%) *m-80*<br>chi angles: 271.3,66	0.16Å	-	-	-	-
A 51		CYS	2.42	0.53Å<br>SG with A 14 CYS HB2	-	Favored (30%) *m*<br>chi angles: 306.8	0.04Å	-	-	-	-

Table S2b. MolProbity Evaluation of AF3 Structure of AFPg.

All-Atom Contacts	Clashscore, all atoms:	3.75		96th percentile* (N=1784, all resolutions)
	Clashscore is the number of serious steric overlaps (> 0.4 Å) per 1000 atoms.			
Protein Geometry	Poor rotamers	0	0.00%	Goal: <0.3%
	Favored rotamers	43	100.00%	Goal: >98%
	Ramachandran outliers	0	0.00%	Goal: <0.05%
	Ramachandran favored	49	100.00%	Goal: >98%
	Rama distribution Z-score	0.42 ± 1.27		Goal: abs(Z score) < 2
	MolProbity score^	1.16		99th percentile* (N=27675, 0Å - 99Å)
	Cβ deviations >0.25Å	0	0.00%	Goal: 0
	Bad bonds:	0 / 408	0.00%	Goal: 0%
	Bad angles:	0 / 540	0.00%	Goal: <0.1%
Peptide Omegas	Cis Prolines:	0 / 1	0.00%	Expected: ≤1 per chain, or ≤5%
Low-resolution Criteria	CaBLAM outliers	0	0.0%	Goal: <1.0%
	CA Geometry outliers	0	0.00%	Goal: <0.5%
Additional validations	Chiral volume outliers	0/52		
	Waters with clashes	0/0	0.00%	See UnDowser table for details

In the two column results, the left column gives the raw count, right column gives the percentage.
* 100th percentile is the best among structures of comparable resolution; 0th percentile is the worst. For clashscore the comparative set of structures was selected in 2004, for MolProbity score in 2006.
^ MolProbity score combines the clashscore, rotamer, and Ramachandran evaluations into a single score, normalized to be on the same scale as X-ray resolution.
Key to table colors and cutoffs here:

Live sorting requires JavaScript enabled and Safari 2, Firefox 1.5, or IE 6

#	Alt	Res	High B	Clash > 0.4Å	Ramachandran	Rotamer	Cβ deviation	CaBLAM	Bond lengths	Bond angles	Cis Peptides
			Avg: 95.54	Clashscore: 3.75	Outliers: 0 of 49	Poor rotamers: 0 of 43	Outliers: 0 of 47	Outliers: 0 of 47	Outliers: 0 of 51	Outliers: 0 of 51	Non-Trans: 0 of 50
A 1		ALA	93.01	-	-	-	0.03Å	-	-	-	-
A 2		THR	95.95	-	Favored (56.88%)<br>General / -116.1,131.7	Favored (83.8%) *m*<br>chi angles: 301.9	0.04Å	-	-	-	-
A 3		TYR	96.88	-	Favored (56.04%)<br>General / -113.0,126.9	Favored (66.7%) *m-80*<br>chi angles: 305,281.1	0.03Å	Favored (63.097%)	-	-	-
A 4		ASN	97.18	-	Favored (25.9%)<br>General / -86.5,147.6	Favored (28%) *m110*<br>chi angles: 296.4,107.3	0.03Å	Favored (6.197%)<br>beta sheet	-	-	-
A 5		GLY	97.88	-	Favored (13.99%)<br>Glycine / -161.8,-163.1	-	-	Favored (43.405%)<br>beta sheet	-	-	-
A 6		LYS	98.46	-	Favored (51.99%)<br>General / -127.7,140.6	Favored (98.7%) *mttt*<br>chi angles: 293.4,180.3,179.7,179.1	0.05Å	Favored (18.141%)<br>beta sheet	-	-	-
A 7		CYS	98.69	-	Favored (12.24%)<br>General / -103.9,163.6	Favored (27.9%) *p*<br>chi angles: 67.3	0.02Å	Favored (34.27%)<br>beta sheet	-	-	-
A 8		TYR	98.03	0.46Å<br>CE1 with A 15 LYS HD3	Favored (34.57%)<br>General / -129.3,124.5	Favored (79.4%) *m-80*<br>chi angles: 295.8,81.9	0.03Å	Favored (53.589%)	-	-	-
A 9		LYS	96.57	-	Favored (96.26%)<br>General / -61.5,-44.9	Favored (86.2%) *tttt*<br>chi angles: 185.6,178.8,180.2,180.6	0.02Å	Favored (50.572%)	-	-	-
A 10		LYS	94.93	-	Favored (71.99%)<br>General / -57.6,-50.8	Favored (85.8%) *tttt*<br>chi angles: 187.4,175.7,181.7,178.8	0.03Å	Favored (55.542%)<br>alpha helix	-	-	-
A 11		ASP	95.46	-	Favored (41.19%)<br>General / -90.8,-10.9	Favored (75.6%) *m-30*<br>chi angles: 297.7,333.4	0.02Å	Favored (35.437%)	-	-	-
A 12		ASN	96.42	-	Favored (14.95%)<br>General / 55.3,50.5	Favored (62%) *t0*<br>chi angles: 197.4,40.2	0.01Å	Favored (20.939%)	-	-	-
A 13		ILE	98.07	-	Favored (29.53%)<br>Ile or Val / -128.7,164.9	Favored (35.6%) *pt*<br>chi angles: 58.9,175.5	0.04Å	Favored (29.905%)<br>beta sheet	-	-	-
A 14		CYS	98.47	0.70Å<br>HB3 with A 49 CYS SG	Favored (38.48%)<br>General / -122.5,122.2	Favored (36.6%) *t*<br>chi angles: 174.7	0.04Å	Favored (53.755%)<br>beta sheet	-	-	-
A 15		LYS	98.49	0.46Å<br>HD3 with A 8 TYR CE1	Favored (44.65%)<br>General / -99.6,124.3	Favored (52.7%) *mtpt*<br>chi angles: 294.3,178.9,65.7,177	0.05Å	Favored (52.651%)<br>beta sheet	-	-	-
A 16		TYR	98.25	-	Favored (26.22%)<br>General / -137.8,165.5	Favored (49.6%) *p90*<br>chi angles: 62,276.1	0.03Å	Favored (36.021%)<br>beta sheet	-	-	-
A 17		LYS	96.83	-	Favored (28.16%)<br>General / -89.0,117.6	Favored (98.8%) *mttt*<br>chi angles: 293.7,180.9,179.9,179.2	0.03Å	Favored (32.57%)	-	-	-
A 18		ALA	94.95	-	Favored (14.38%)<br>General / -76.6,171.6	-	0.03Å	Favored (32.184%)	-	-	-
A 19		GLN	91.58	-	Favored (68.88%)<br>General / -62.5,-27.1	Favored (56.6%) *tt0*<br>chi angles: 190.7,172.3,8.9	0.01Å	Favored (51.564%)	-	-	-
A 20		SER	92.05	-	Favored (63.09%)<br>General / -70.0,-15.8	Favored (95.3%) *p*<br>chi angles: 63.9	0.04Å	Favored (29.91%)	-	-	-
A 21		GLY	93.2	-	Favored (63.61%)<br>Glycine / 97.4,-6.9	-	-	Favored (76.249%)	-	-	-
A 22		LYS	96.11	-	Favored (14.02%)<br>General / -96.1,161.3	Favored (70.3%) *mmtt*<br>chi angles: 302.2,300.8,179.6,182.2	0.03Å	Favored (24.65%)	-	-	-
A 23		THR	97.19	-	Favored (36.55%)<br>General / -79.4,133.4	Favored (84.6%) *m*<br>chi angles: 301.8	0.03Å	Favored (32.864%)<br>beta sheet	-	-	-
A 24		ALA	97.93	-	Favored (17.69%)<br>General / -126.1,166.1	-	0.03Å	Favored (47.676%)<br>beta sheet	-	-	-
A 25		ILE	97.64	-	Favored (48.46%)<br>Ile or Val / -128.8,140.7	Favored (75.6%) *mt*<br>chi angles: 300,174.5	0.02Å	Favored (54.812%)<br>beta sheet	-	-	-
A 26		CYS	97.77	-	Favored (51.77%)<br>General / -135.2,153.9	Favored (15.4%) *p*<br>chi angles: 56	0.02Å	Favored (50.343%)<br>beta sheet	-	-	-
A 27		LYS	96.74	-	Favored (28.92%)<br>General / -94.3,141.1	Favored (41.1%) *tttt*<br>chi angles: 191.3,154.4,186.1,171.4	0.02Å	Favored (39.844%)<br>beta sheet	-	-	-
A 28		CYS	95.96	-	Favored (10.35%)<br>General / -83.2,100.1	Favored (18.6%) *m*<br>chi angles: 308.4	0.06Å	Favored (28.727%)<br>beta sheet	-	-	-
A 29		TYR	88.47	-	Favored (10.48%)<br>General / -93.5,-40.2	Favored (45.8%) *m-80*<br>chi angles: 286.5,294	0.01Å	Favored (12.003%)	-	-	-
A 30		VAL	80.86	-	Favored (29.23%)<br>Ile or Val / -69.8,-52.7	Favored (45.3%) *t*<br>chi angles: 182.4	0.02Å	Favored (32.647%)<br>alpha helix	-	-	-
A 31		LYS	85.29	-	Favored (46.08%)<br>General / -135.1,141.6	Favored (97%) *mttt*<br>chi angles: 290.4,184.4,178.2,180.6	0.04Å	Favored (21.068%)	-	-	-
A 32		LYS	94.03	-	Favored (44.13%)<br>General / -71.0,151.9	Favored (98%) *mttt*<br>chi angles: 292.3,183.3,178.7,180.1	0.03Å	Favored (39.828%)	-	-	-
A 33		CYS	97.17	-	Favored (87.07%)<br>Pre-Pro / -64.0,125.0	Favored (55.8%) *t*<br>chi angles: 181.7	0.05Å	Favored (34.271%)	-	-	-
A 34		PRO	96.12	-	Favored (40.7%)<br>Trans-Pro / -65.6,-34.9	Favored (75.6%) *Cg_endo*<br>chi angles: 28.8,321.3,33.1	0.02Å	Favored (10.227%)	-	-	-
A 35		ARG	97.24	-	Favored (16.38%)<br>General / -163.6,154.6	Favored (17.6%) *ptm160*<br>chi angles: 57.3,179.8,293.3,174.9	0.03Å	Favored (7.476%)	-	-	-
A 36		ASP	97.42	-	Favored (53.72%)<br>General / -60.2,132.4	Favored (93.9%) *m-30*<br>chi angles: 292.2,340.4	0.04Å	Favored (28.265%)	-	-	-
A 37		GLY	97.63	-	Favored (86.17%)<br>Glycine / 87.3,-1.5	-	-	Favored (75.485%)	-	-	-
A 38		ALA	98.26	-	Favored (49.31%)<br>General / -66.1,150.1	-	0.03Å	Favored (20.51%)	-	-	-
A 39		LYS	97.87	-	Favored (56.46%)<br>General / -67.5,142.9	Favored (98.7%) *mttt*<br>chi angles: 293.7,181.7,179.6,179.5	0.02Å	Favored (24.549%)<br>beta sheet	-	-	-
A 40		CYS	98.25	-	Favored (22.35%)<br>General / -146.1,167.9	Favored (25.1%) *p*<br>chi angles: 59.5	0.03Å	Favored (50.282%)<br>beta sheet	-	-	-
A 41		GLU	97.07	-	Favored (42.83%)<br>General / -139.6,148.7	Favored (89.5%) *mt-10*<br>chi angles: 300.5,175.6,359.2	0.06Å	Favored (62.181%)<br>beta sheet	-	-	-
A 42		PHE	96.55	-	Favored (37.45%)<br>General / -120.6,121.0	Favored (68.3%) *t80*<br>chi angles: 173.2,69.1	0.05Å	Favored (62.727%)<br>beta sheet	-	-	-
A 43		ASP	95.43	-	Favored (20.49%)<br>General / -96.9,107.9	Favored (42.3%) *t0*<br>chi angles: 188.3,327.3	0.05Å	Favored (71.529%)<br>beta sheet	-	-	-
A 44		SER	94.23	-	Favored (65.35%)<br>General / -68.5,-26.2	Favored (97.5%) *p*<br>chi angles: 63.5	0.01Å	Favored (52.273%)	-	-	-
A 45		TYR	93.96	-	Favored (62.35%)<br>General / -73.5,-42.0	Favored (20%) *m-10*<br>chi angles: 291.3,340.9	0.01Å	Favored (56.487%)<br>alpha helix	-	-	-
A 46		LYS	93.14	-	Favored (28.08%)<br>General / -88.0,-18.9	Favored (97.9%) *mttt*<br>chi angles: 296.1,181.2,179.8,179.5	0.02Å	Favored (36.575%)	-	-	-
A 47		GLY	92.54	-	Favored (78.3%)<br>Glycine / 67.8,32.8	-	-	Favored (64.828%)	-	-	-
A 48		LYS	94.43	-	Favored (46.01%)<br>General / -127.1,153.5	Favored (95.3%) *mttt*<br>chi angles: 291.1,186.1,177.3,181.7	0.02Å	Favored (29.61%)<br>beta sheet	-	-	-
A 49		CYS	95.57	0.70Å<br>SG with A 14 CYS HB3	Favored (51.69%)<br>General / -105.6,132.5	Favored (2.5%) *t*<br>chi angles: 203.1	0.09Å	Favored (60.101%)	-	-	-
A 50		TYR	96.13	-	Favored (21.82%)<br>General / -117.8,113.5	Favored (90.8%) *m-80*<br>chi angles: 299.4,281	0.04Å	-	-	-	-
A 51		CYS	94.29	-	-	Favored (14.8%) *m*<br>chi angles: 309.9	0.03Å	-	-	-	-

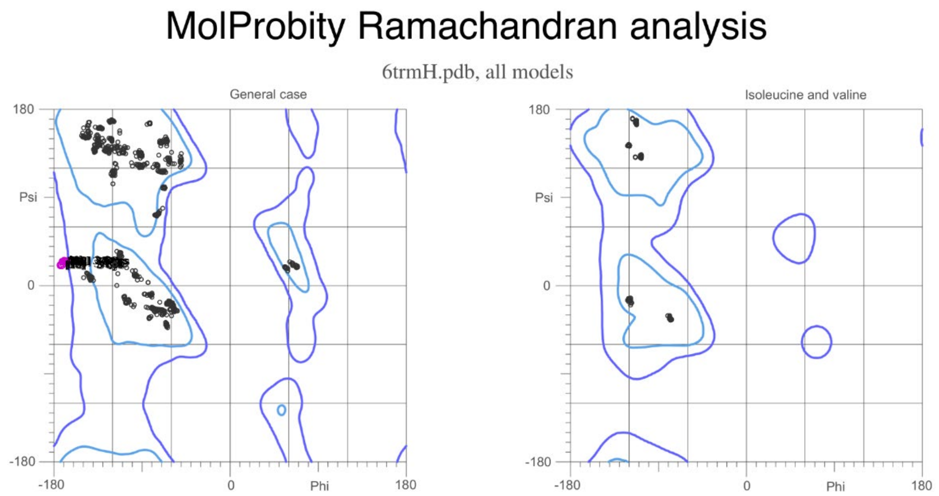

Figure S8a. PAFC-NMR, PDB ID: 6TRM

92.8% (1151/1240) of all residues were in favored (98%) regions.
98.4% (1220/1240) of all residues were in allowed (>99.8%) regions.

There were 20 outliers (phi, psi):

[1] 3 Cys (-170.4, 26.0)
[2] 3 Cys (-168.8, 25.7)
[3] 3 Cys (-170.0, 25.8)
[4] 3 Cys (-172.7, 25.4)
[5] 3 Cys (-169.1, 25.5)
[6] 3 Cys (-170.8, 26.5)
[7] 3 Cys (-168.7, 25.3)
[8] 3 Cys (-171.6, 22.4)
[9] 3 Cys (-169.4, 25.9)
[10] 3 Cys (-168.5, 25.3)
[11] 3 Cys (-167.1, 24.8)
[12] 3 Cys (-167.7, 25.0)
[13] 3 Cys (-171.7, 25.0)
[14] 3 Cys (-172.7, 25.0)
[15] 3 Cys (-169.4, 25.7)
[16] 3 Cys (-170.0, 25.7)
[17] 3 Cys (-174.2, 21.2)
[18] 3 Cys (-173.0, 20.7)
[19] 3 Cys (-173.8, 21.9)
[20] 3 Cys (-174.3, 20.8)

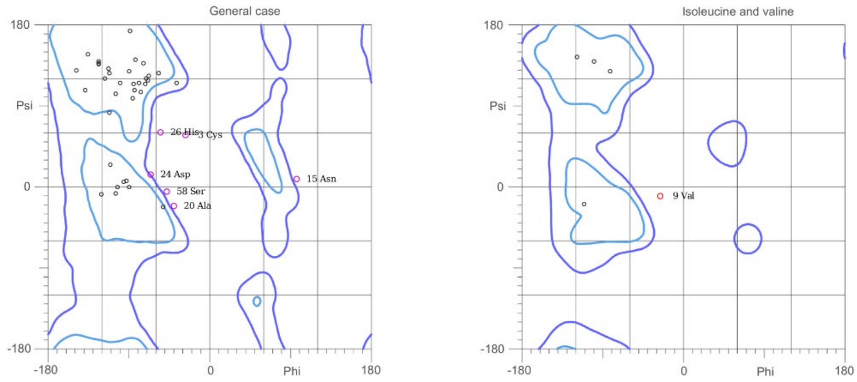

Figure S8a. PAFC-AF2. 75.8% (47/62) of all residues were in favored (98%) regions. 82.3% (51/62) of all residues were in allowed (>99.8%) regions. There were 11 outliers (phi, psi): 3 Cys (-27.5, 58.7), 4 Gly (-9.6, 83.8), 5 Gly (-13.4, 34.8), 9 Val (-26.5, -10.4), 15 Asn (97.8, 9.7), 20 Ala (-40.9, -21.2), 23 Gly (22.3, -162.0), 24 Asp (-66.6, 14.7).

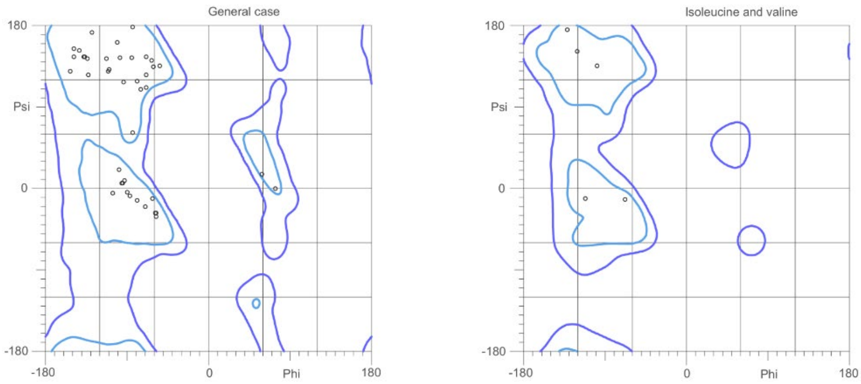

Figure S8a. PAFC-AF3. 100.0% (62/62) of all residues were in favored (98%) regions. 100.0% (62/62) of all residues were in allowed (>99.8%) regions. There were no outliers.

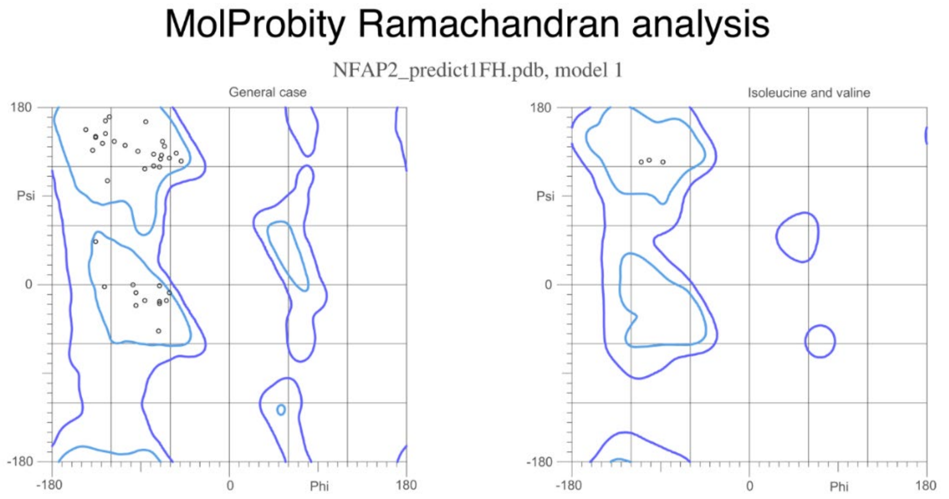

Figure S8b. NFAP2-AF2. 100.0% (50/50) of all residues were in favored (98%) regions. 100.0% (50/50) of all residues were in allowed (>99.8%) regions. There were no outliers.

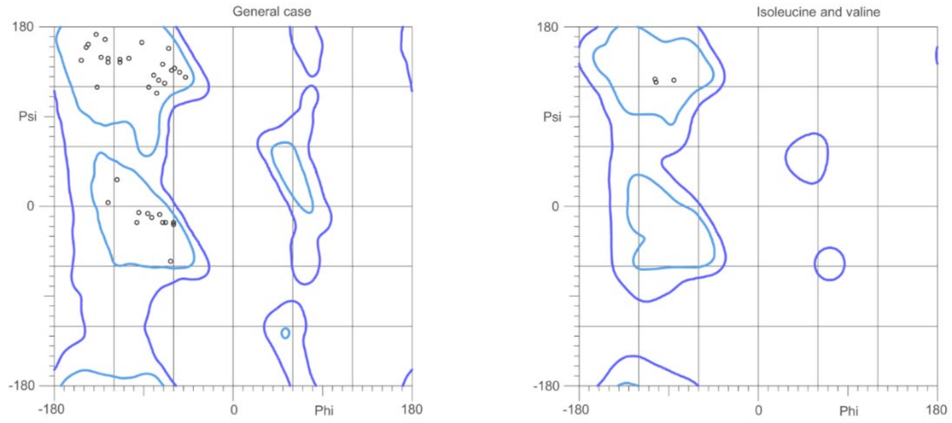

Figure S8b. NFAP2-AF3. 100.0% (50/50) of all residues were in favored (98%) regions. 100.0% (50/50) of all residues were in allowed (>99.8%) regions. There were no outliers.

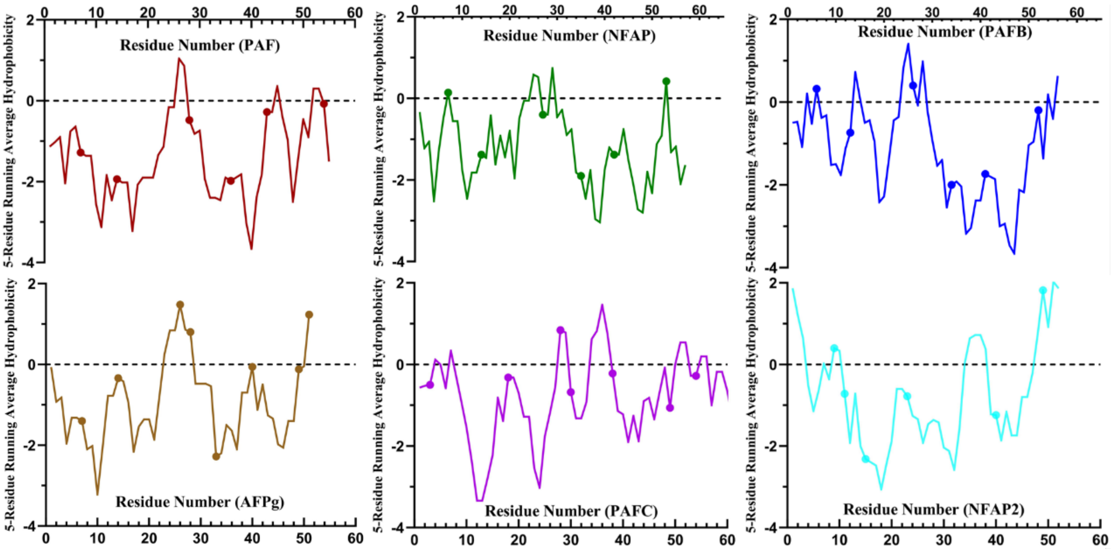

Figure S9. Hydrophilicity of the AFPs AF3 Structure. This figure illustrates the hydrophilicity distribution of AFPs in the AF3-predicted structure. Cysteine residues are highlighted to indicate their specific positions within the protein structure.
