# Supplementary figures and images for: Small Disulfide Proteins with Antifungal Impact: NMR Experimental Structures as Compared to Models of Alphafold Versions
Source: Int J Mol Sci. 2025 Jan 31;26(3):1247. doi: 10.3390/ijms26031247 (PMC11818080; doi:10.3390/ijms26031247)

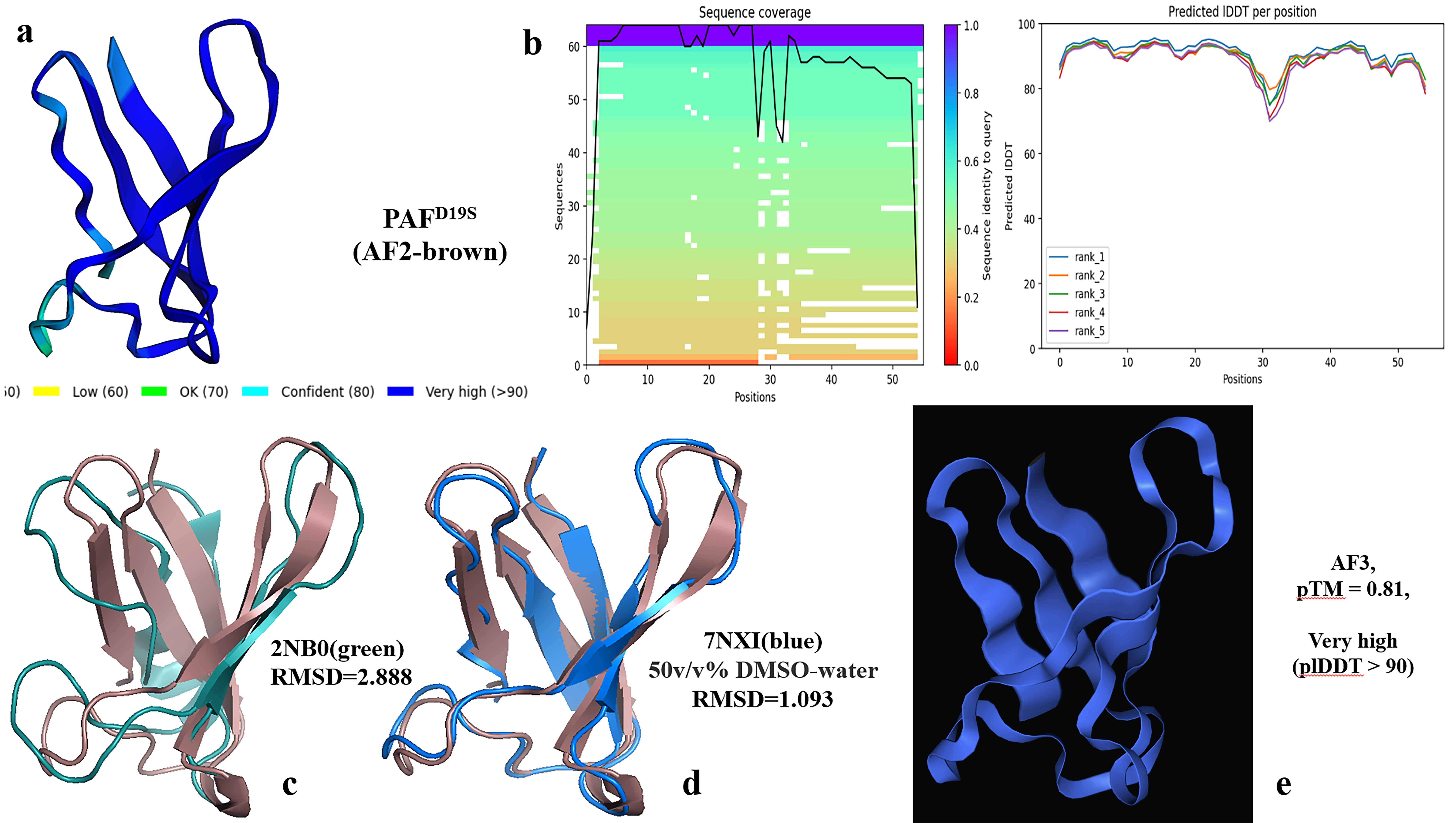

Supplement: Supplementary file 1 [file ijms-26-01247-s001.zip › Figure S1.png]

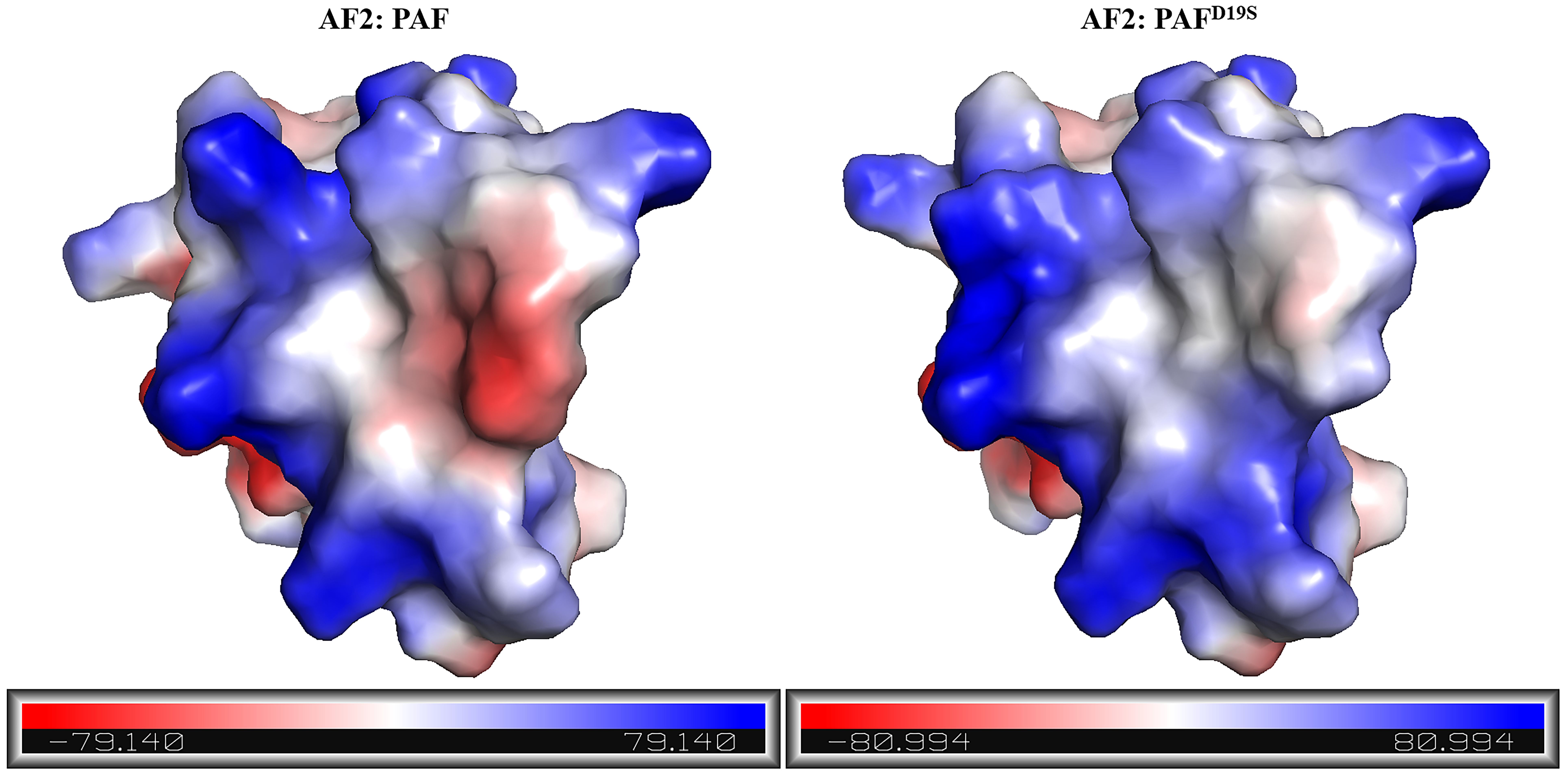

Supplement: Supplementary file 1 [file ijms-26-01247-s001.zip › Figure S2.png]

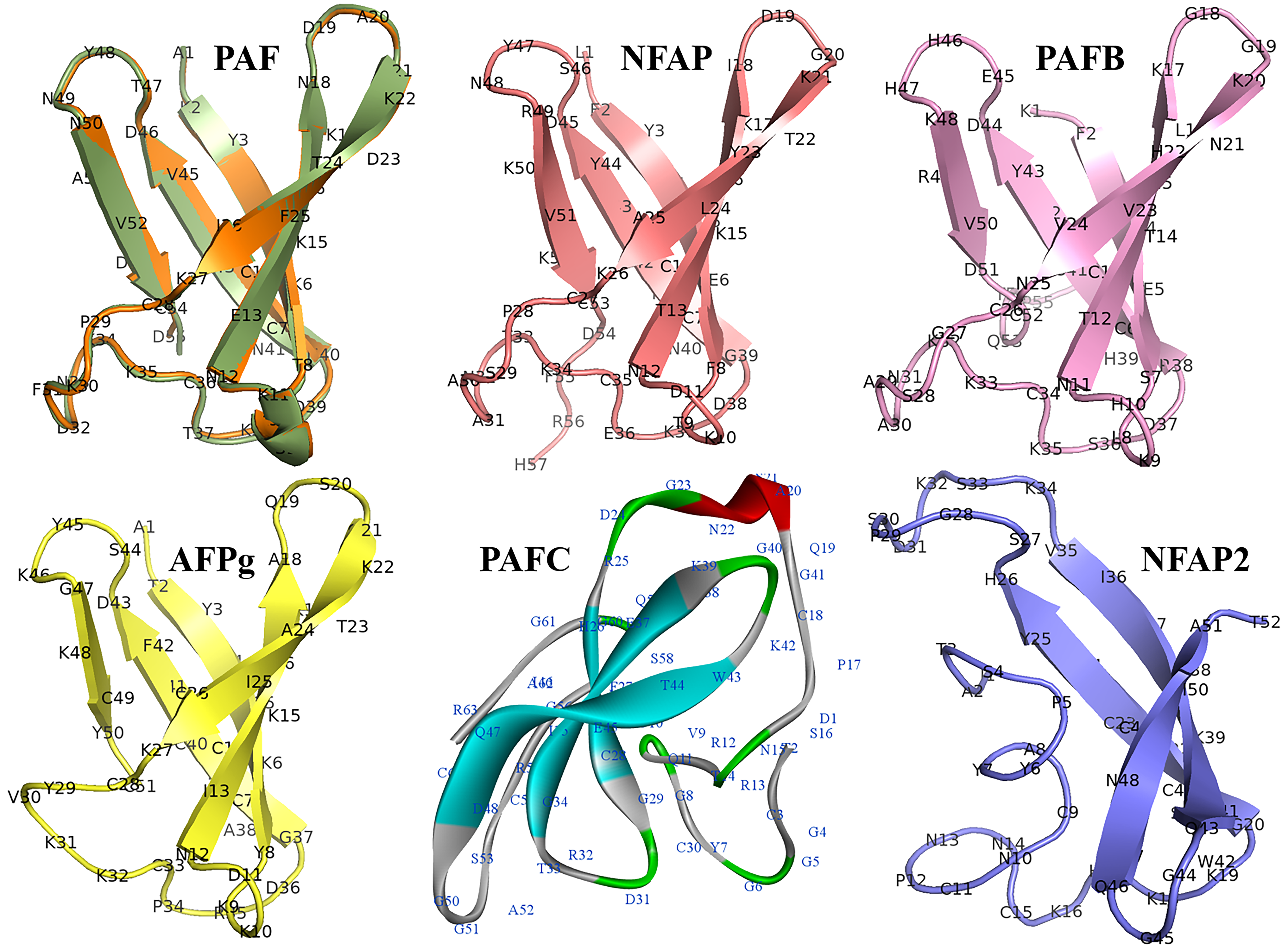

Supplement: Supplementary file 1 [file ijms-26-01247-s001.zip › Figure S4.png]

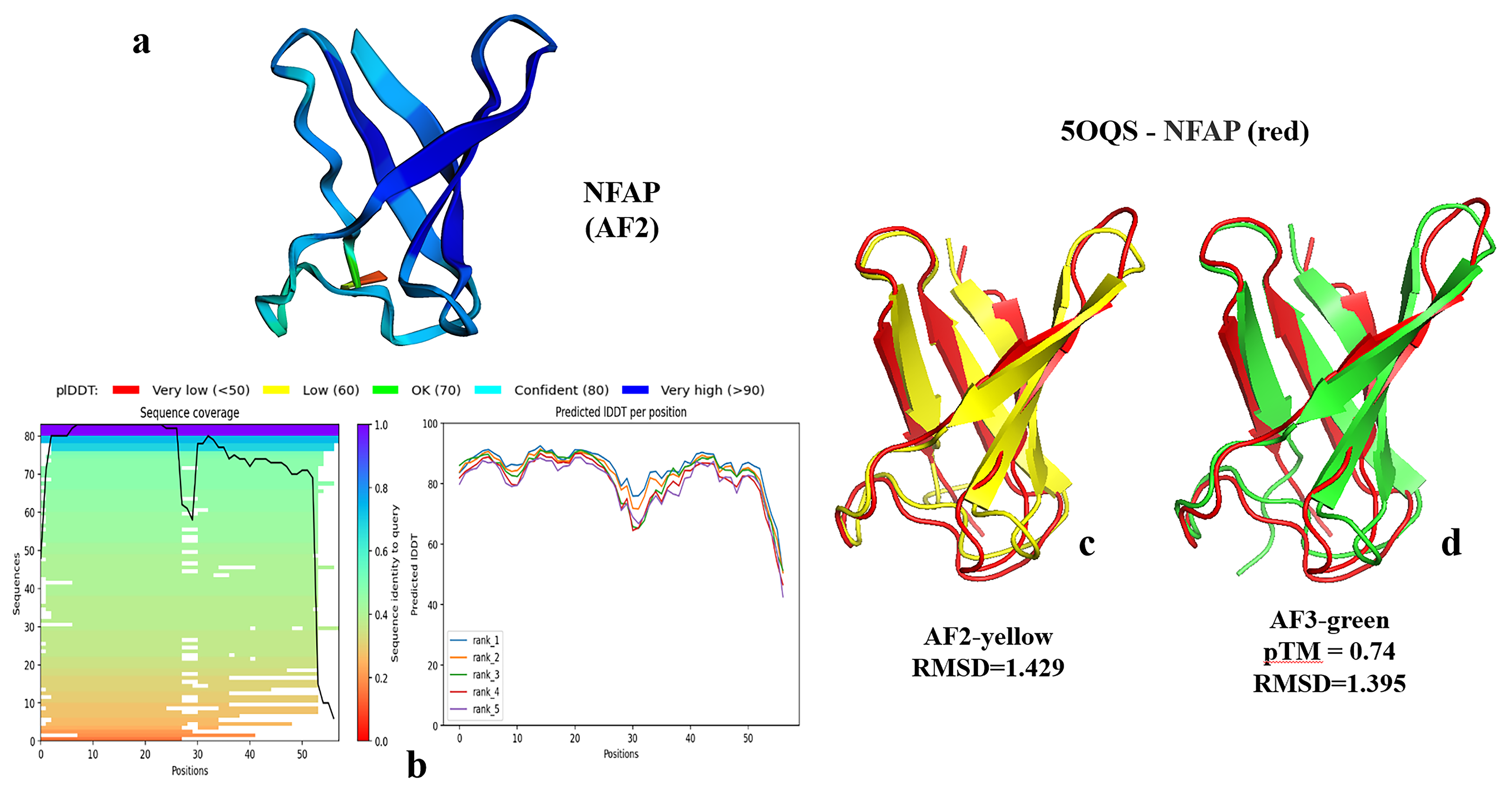

Supplement: Supplementary file 1 [file ijms-26-01247-s001.zip › Figure S5a.png]

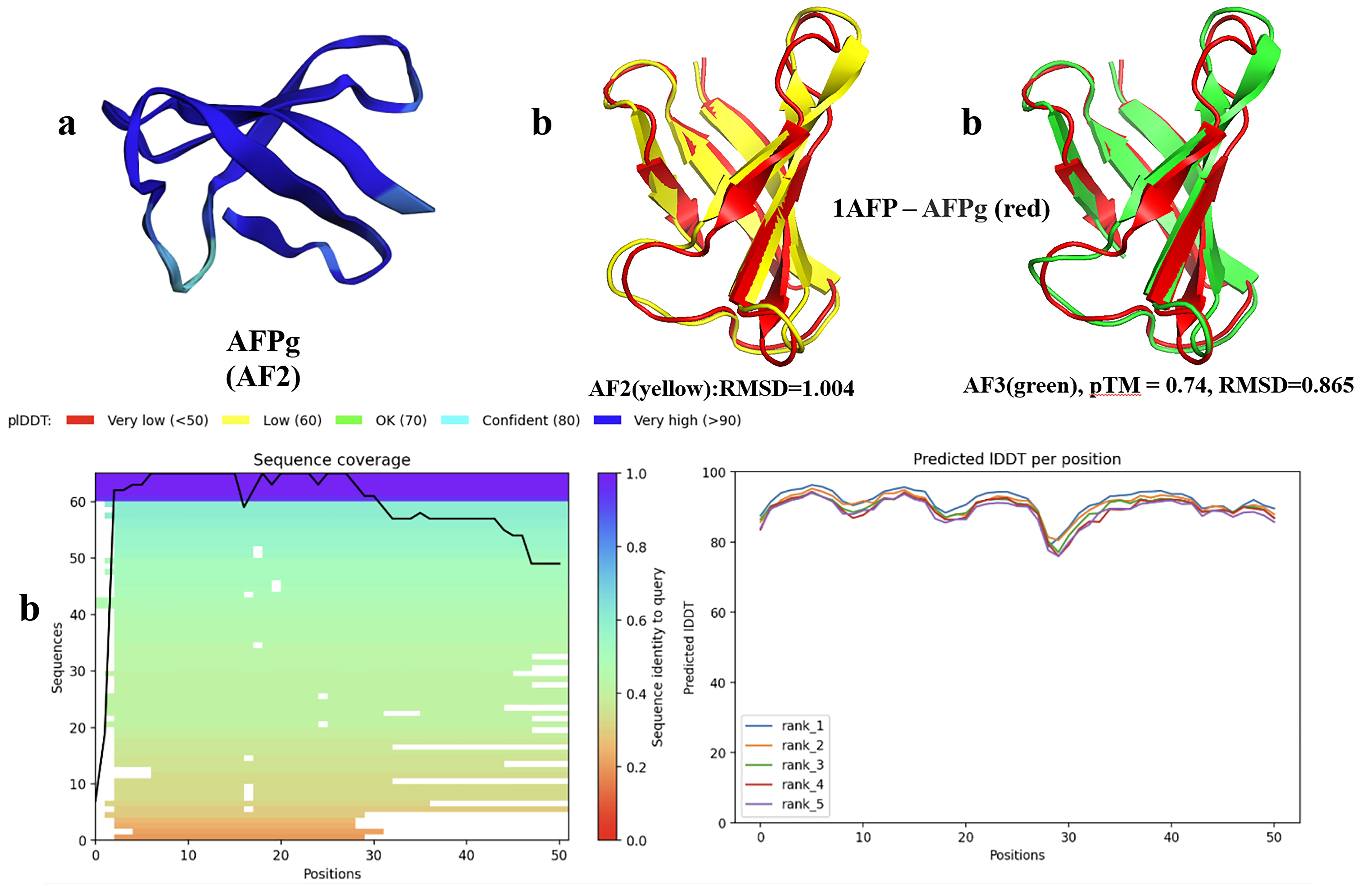

Supplement: Supplementary file 1 [file ijms-26-01247-s001.zip › Figure S5b.png]

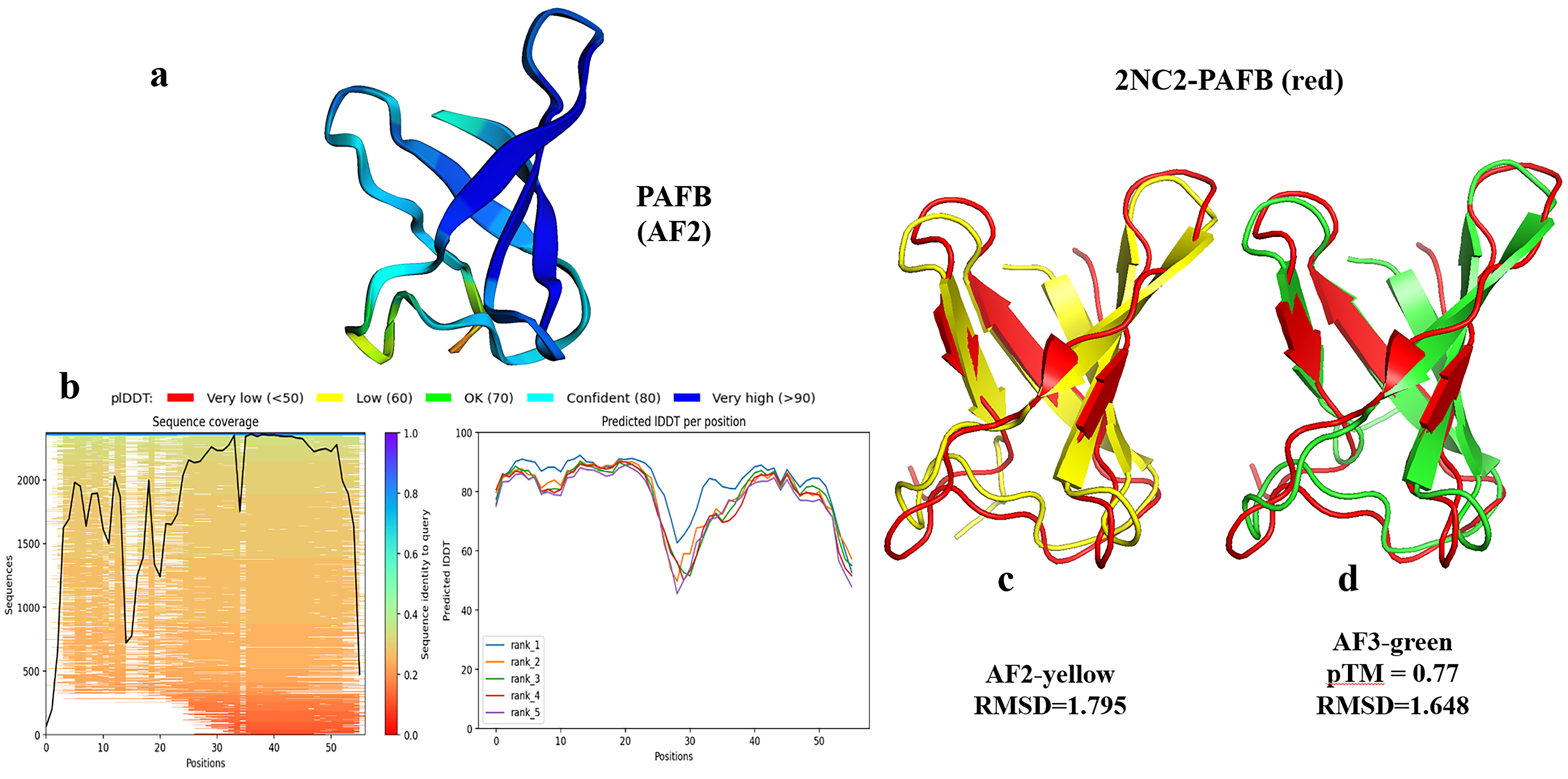

Supplement: Supplementary file 1 [file ijms-26-01247-s001.zip › Figure S5c.png]

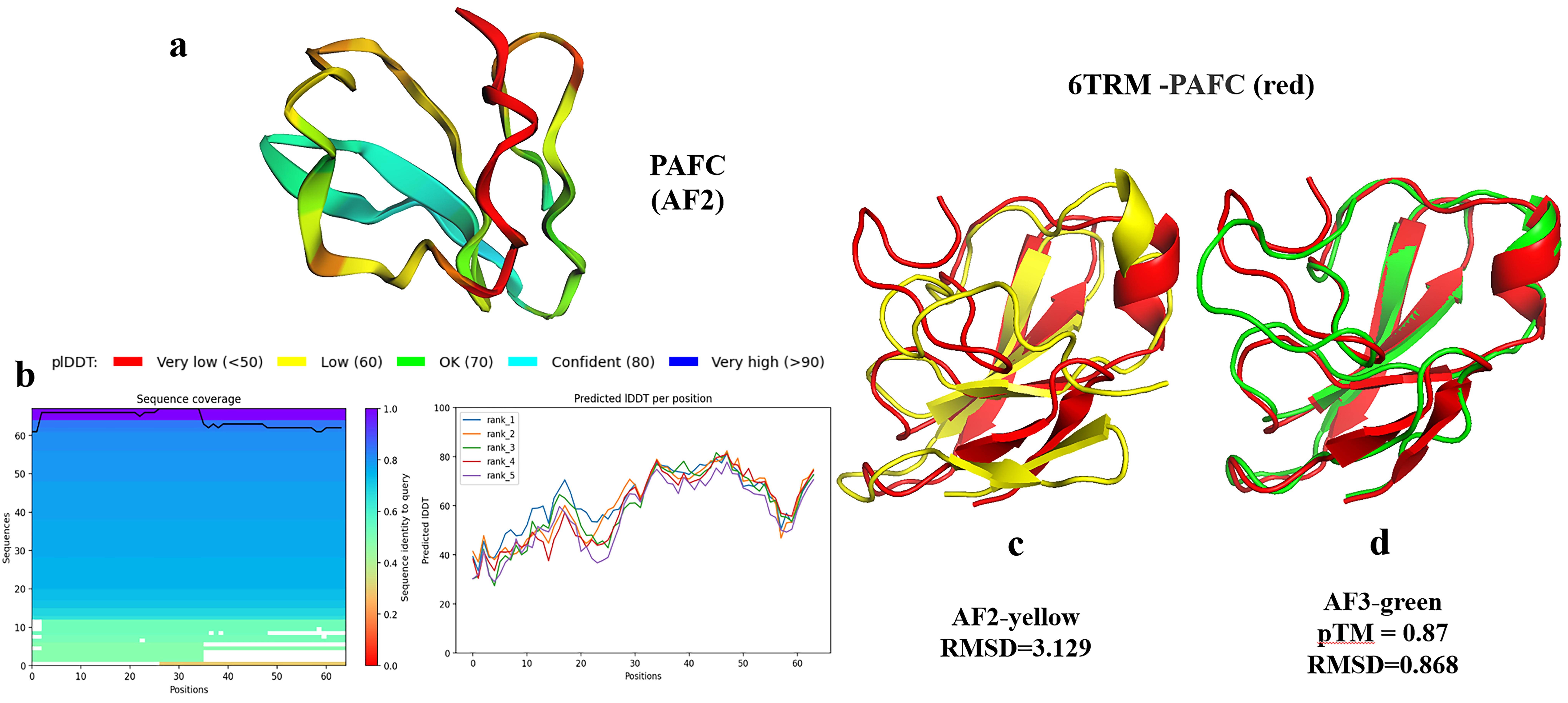

Supplement: Supplementary file 1 [file ijms-26-01247-s001.zip › Figure S5d.png]

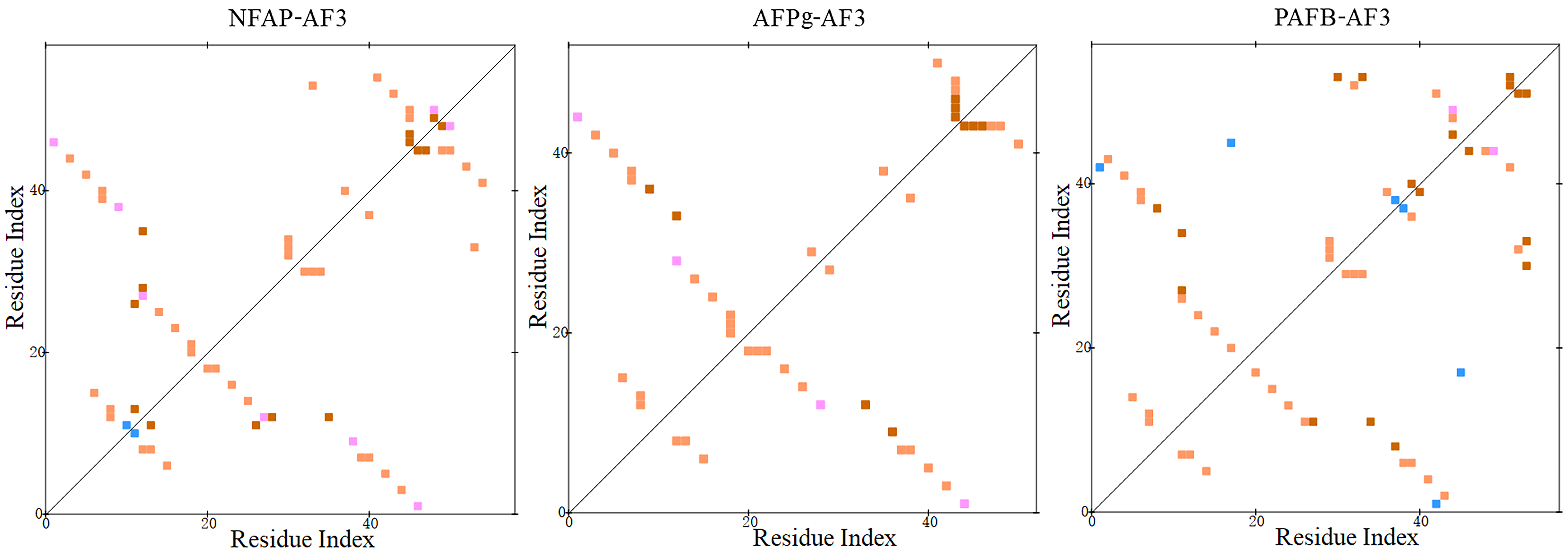

Supplement: Supplementary file 1 [file ijms-26-01247-s001.zip › Figure S6.png]

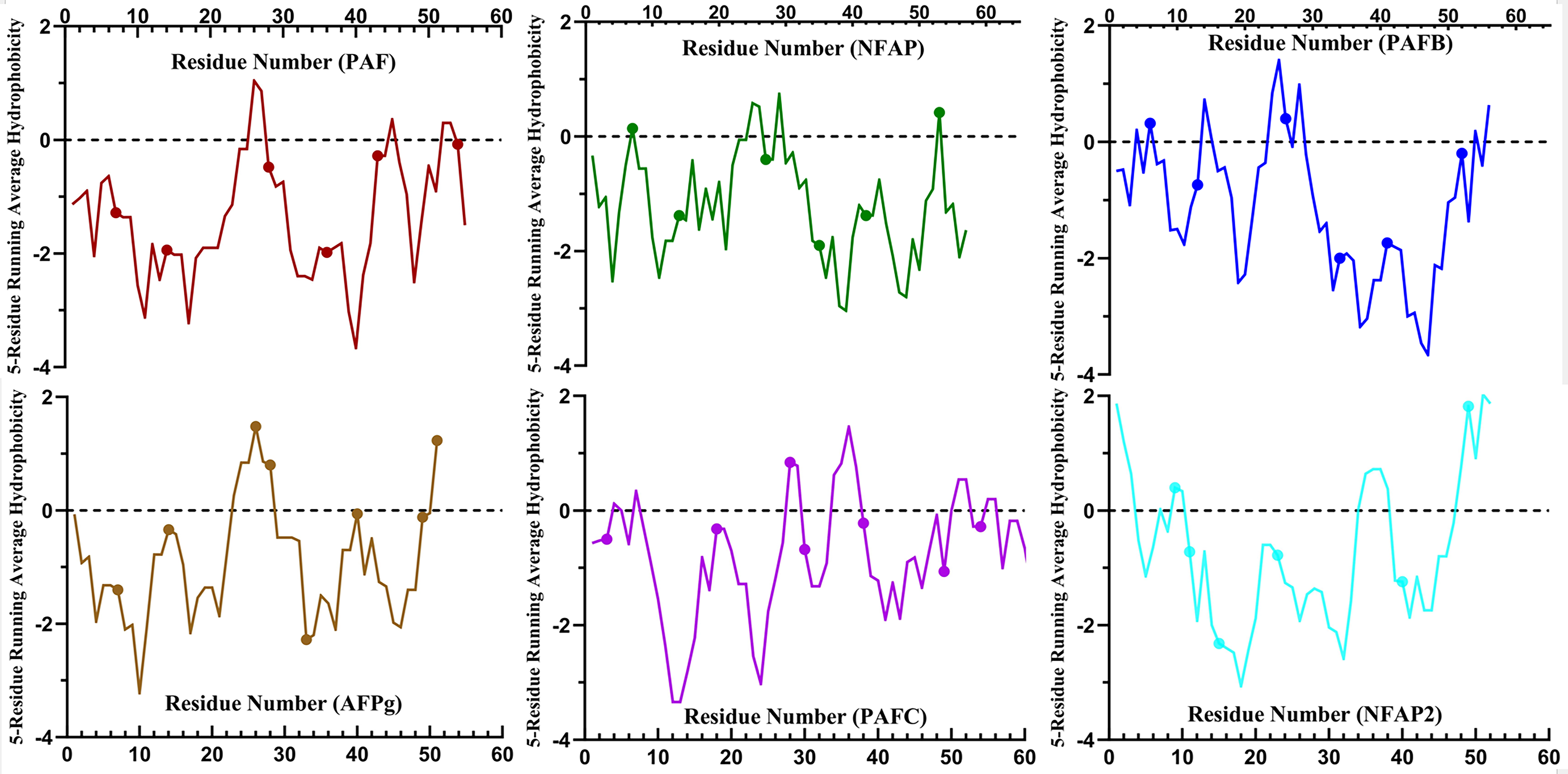

Supplement: Supplementary file 1 [file ijms-26-01247-s001.zip › Figure S9.png]
